# CD73 restrains mutant **β**-catenin oncogenic activity in endometrial carcinomas

**DOI:** 10.1172/jci.insight.189510

**Published:** 2026-01-23

**Authors:** Rebecca M. Hirsch, Gaith Droby, Sunthoshini Premsankar, Molly L. Parrish, Katherine C. Kurnit, Lilly F. Chiou, Emily M. Rabjohns, Hannah N. Lee, Russell R. Broaddus, Cyrus Vaziri, Jessica L. Bowser

**Affiliations:** 1 Department of Pathology and Laboratory Medicine,; 2 Curriculum in Cell Biology and Physiology,; 3 Curriculum in Genetics and Molecular Biology,; 4 Chancellor’s Science Scholars Program, and; 5 Curriculum in Pathobiology and Translational Science, University of North Carolina, Chapel Hill, North Carolina, USA.; 6 Department of Obstetrics and Gynecology, University of Chicago, Chicago, Illinois, USA.; 7 UNC Lineberger Comprehensive Cancer Center, University of North Carolina, Chapel Hill, North Carolina, USA.

**Keywords:** Cell biology, Oncology, Cancer, Obstetrics/gynecology, Oncogenes

## Abstract

Approximately 30% of patients with endometrial carcinomas (ECs) with exon 3 *CTNNB1* (β-catenin) mutations experience disease recurrence, whereas others with the same mutations remain recurrence-free. The molecular factors driving mutant β-catenin’s oncogenic and clinical variability are unknown. Here we show that CD73 restrains the oncogenic activity of exon 3 β-catenin mutants, and CD73 loss is associated with recurrence. Using 7 patient-specific β-catenin mutants, together with genetic deletion or ectopic expression of CD73, we demonstrate that CD73 loss increases β-catenin–TCF/LEF transcriptional activity. In CD73-deficient cells, membrane levels of mutant β-catenin decreased, which corresponded with increased levels of nuclear and chromatin-bound mutant β-catenin. These results suggest that CD73 sequesters mutant β-catenin to the membrane to limit its oncogenic activity. Adenosine A1 receptor deletion phenocopied the effects of CD73 loss, implicating adenosine receptor signaling in this regulation. Ectopic CD73 expression suppressed the invasiveness and stemness capacity of β-catenin–mutant EC cells. TCGA analyses, GeoMx digital spatial profiling, and functional analyses showed that CD73 loss drives distinct Wnt–TCF/LEF–dependent gene expression programs linked to cancer cell stemness. These findings identify CD73 as a key regulator of mutant β-catenin, providing mechanistic insight into the variability of recurrence in *CTNNB1*-mutant EC.

## Abstract 2

CD73 regulates the oncogenic activity of mutant b-catenin. This finding may help improve predicting which endometrial cancer patients are at a higher risk of recurrence.

## Graphical abstract

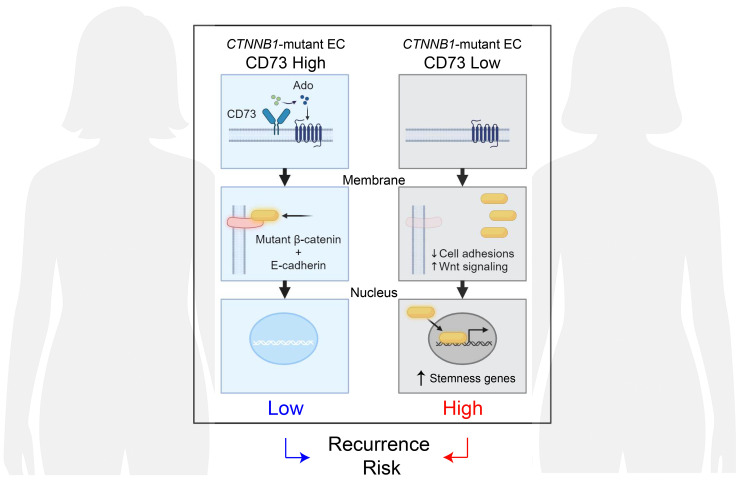

## Introduction

β-Catenin, encoded by the gene *CTNNB1*, is an essential component of cell-cell adhesions (1–5) and a transcriptional coactivator of Wnt signaling (6–10). β-Catenin is a crucial oncogene in various types of cancer and is often activated by somatic missense mutations in exon 3 of *CTNNB1* (11–15). In endometrial carcinomas (ECs), exon 3 *CTNNB1* mutations occur frequently (~20%–30% on average), especially in low-grade, early-stage endometrioid EC (EEC) (16–19). Exon 3 mutations prevent degradation of β-catenin protein, leading to its cytoplasmic accumulation and subsequent nuclear translocation and oncogenic transcriptional activity (13, 14, 20). Several studies implicate *CTNNB1* mutations as oncogenic drivers in EC. For instance, *CTNNB1* mutations are seen as early as atypical hyperplasia, and expression of a dominant stabilized *CTNNB1* exon 3 in murine studies results in endometrial hyperplasia (21–23). Many more studies report that high expression of β-catenin or *CTNNB1* mutations was associated with recurrence, worse recurrence-free survival, or overall survival (16, 17, 24–28) and that *CTNNB1* mutation is a greater risk factor for recurrence than other aggressive clinical features, such as myometrial invasion and lymphovascular space invasion (17).

Despite a close association with recurrence, *CTNNB1* status has been challenging to use clinically for identifying patients at high risk for poor outcomes. *CTNNB1* mutation alone has poor sensitivity and specificity in predicting disease recurrence. Some patients with *CTNNB1*-mutant tumors will have disease recurrence (~30%), yet many others with genomically identical tumors never have recurrence (28). Additionally, studies assessing the nuclear localization of β-catenin by immunohistochemistry as a possible approach for predicting recurrence have shown that the percentage of β-catenin nuclear expression in *CTNNB1*-mutant EC (29) and/or endometrial tumors with aberrant β-catenin expression (30) is widely variable (~5%–60%) (29), with no clear correlation with outcomes. The variability in outcomes has suggested that mutant β-catenin oncogenic activity is modified by other factors. These putative factors have yet to be identified, and this lack of knowledge represents a critical barrier to accurate risk stratification and the development of targeted interventions for patients with *CTNNB1*-mutant EC.

The purpose of this study was to identify molecular determinants that modulate β-catenin–mutant oncogenic activity in EC. Remarkably, we discovered that *NT5E*, the gene encoding the cell surface enzyme CD73, is downregulated in nearly all mutant β-catenin–expressing tumors from patients with recurrent disease. The role of CD73 in EC is atypical because its pathological downregulation (as opposed to its upregulation, as seen in other tumors) is causally linked to tumor aggressiveness and poor survival (31, 32). CD73 is a cell surface enzyme that generates adenosine for adenosine receptor signaling.

We previously showed that CD73-mediated adenosine signaling functions as a homeostatic sensor in endometrial tissue and suppresses EC cell invasiveness in low-grade tumors by promoting cell-cell adhesion through redistributing wild-type (WT) β-catenin to the membrane (31). In EEC, the majority of mutations in *CTNNB1* cluster in exon 3 (88.7%; The Cancer Genome Atlas [TCGA]) in a stretch of 14 amino acids (codons 32–45) (16), which is outside the region that encodes the E-cadherin–binding domain and is essential for β-catenin’s membrane localization (33–36). We hypothesized that CD73 may similarly sequester mutant β-catenin at the membrane, and that CD73 loss would unleash its oncogenic potential, driving tumor aggressiveness.

While CD73 localizes WT β-catenin to the membrane (31), there is no existing paradigm to suggest that mechanisms controlling WT β-catenin would also regulate mutant β-catenin in human tumors. The same principle applies to other major oncogenes; for example, regulators of WT Ras signaling do not always constrain oncogenic Ras mutants (37). Additionally, mechanisms responsible for gain-of-function activities of mutant p53 are not necessarily the same for WT p53 (38). Therefore, it is critical to investigate whether CD73 is a major molecular determinant controlling mutant β-catenin, as doing so could reveal fundamental mechanisms that govern this devastating oncogene and explain, for the first time, its variable aggressiveness in EC. Given that CD73’s role in EC is different from its role in other cancers (31, 32, 39, 40), revealing such biology from studies in other tumors would be challenging if not impossible.

To test our hypothesis, we engineered 7 patient-specific β-catenin mutants in CD73-isogenic EC models and corroborated our findings through integrated analyses of datasets from TCGA for Uterine Cancer and GeoMx spatial profiling of exon 3 *CTNNB1*-mutant endometrial tumors. Here we show that CD73 critically restrains the oncogenic activity and EC cell aggressiveness of exon 3 β-catenin mutants and reveal that CD73 loss in β-catenin–mutant EC induces distinct Wnt–TCF/LEF–driven gene expression programs linked to cancer cell stemness — a feature essential for disease recurrence. We also provide evidence for CD73 as a potential predictive biomarker for recurrence. Altogether, our findings offer a mechanistic explanation for the long-standing clinical question of the variable recurrence observed in patients with *CTNNB1*-mutant EC.

## Results

### Low CD73 expression associates with recurrence in patients with endometrial cancer with exon 3 CTNNB1 mutations.

**Figure 1:**
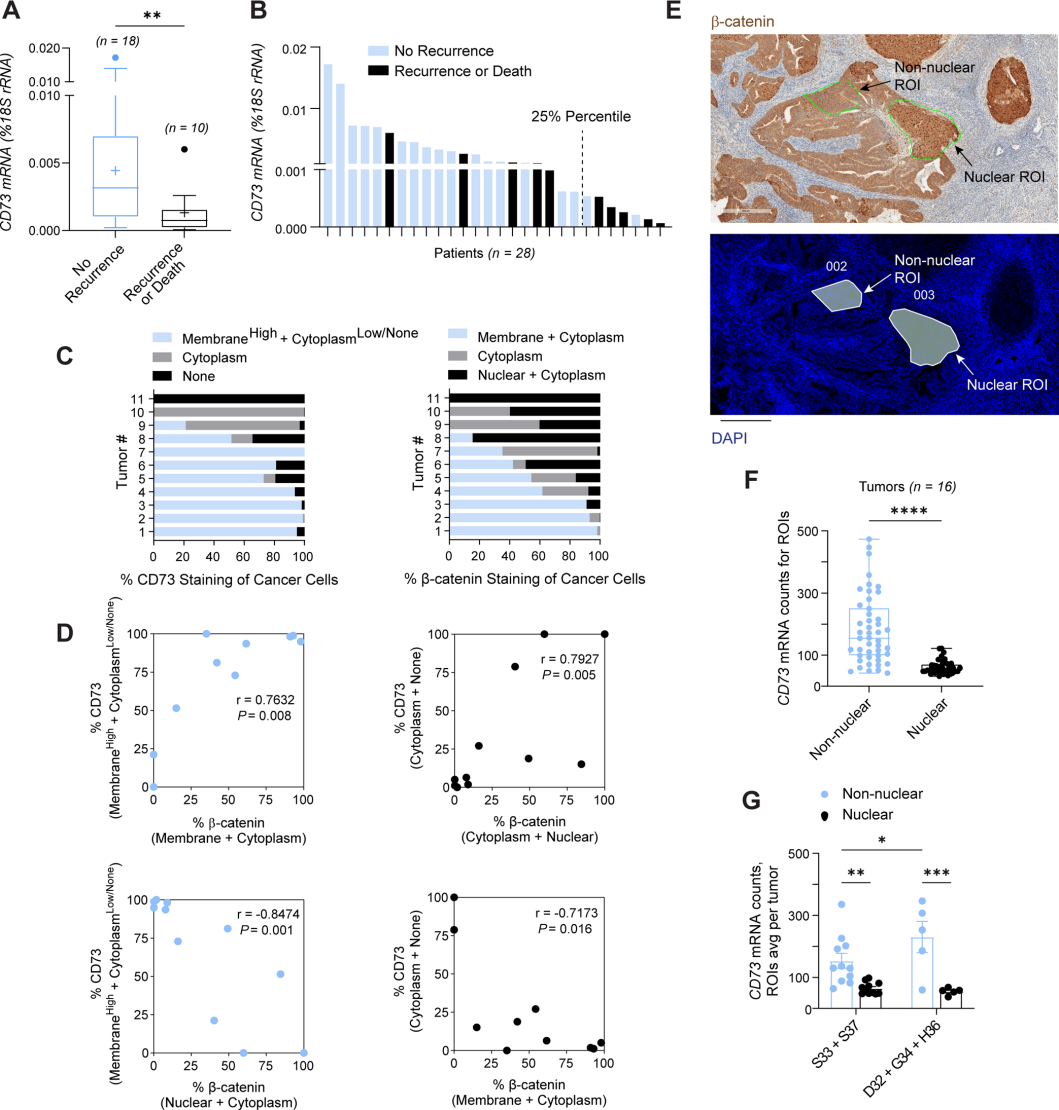
Loss of CD73 associates with recurrence and nuclear β-catenin in exon 3 *CTNNB1*-mutant EC. (**A**) *CD73* mRNA in exon 3 *CTNNB1*-mutant tumors, stratified by recurrence (*n* = 28). Box plots show median, IQR, mean (cross), whiskers (±1.5 × IQR), and outliers (circles). Values are molecules of *CD73* transcripts/molecules of 18S rRNA. (**B**) Distribution of *CD73* mRNA expression in individual tumors; 5 of 7 recurrences fall below the 25th percentile. (**C**) Quantification of CD73 and β-catenin staining in *n* = 11 tumors (cancer cells stained/total cancer cell area imaged). (**D**) Spearman’s correlations of **C**. (**E**–**G**) *CD73* expression from a publicly available GeoMx DSP whole-transcriptome dataset from *n* = 16 exon 3 *CTNNB1*-mutant tumors. (**E**) Representative regions of interest (ROIs) of nuclear versus non-nuclear β-catenin from a tumor of the dataset. Scale bars: 300 μm. (**F**) *CD73* mRNA by ROI type. (**G**) *CD73* mRNA in phosphorylation (S33, S37) versus non-phosphorylation (D32, G34, H36) *CTNNB1*-mutant tumors. **P < 0.01, Mann-Whitney (**A**); *P < 0.05, **P < 0.005, ***P < 0.0005, ****P < 0.0001, Mann-Whitney (**F**) or 2-way ANOVA with Fisher’s least significant difference (**G**).

We first assessed whether CD73 was associated with disease recurrence in β-catenin–mutant EC. *CD73* expression was measured in a published cohort of endometrial tumors verified by next-generation sequencing (NGS) to have exon 3 *CTNNB1* mutations (17). Clinical details for the tumors used in this study are provided in Supplemental Table 1 (supplemental material available online with this article; https://doi.org/10.1172/jci.insight.189510DS1). Tumors were then stratified by patient recurrence or death. *CD73* expression was significantly lower in patients with recurrence or who died of their disease compared with patients with no recurrence (Figure 1A). *CD73* expression was not significantly different when the tumors were stratified by surgical stage (Supplemental Figure 1A) or lymphovascular space invasion (Supplemental Figure 1B), indicating that *CD73* expression associates with recurrence. *CD73* expression plotted for individual patients revealed that the lower quartile contained most of the recurrences (Figure 1B). Of these patients, 71% had recurrence or death. Notably, the lower quartile value (0.0055 molecules of *CD73*) is similar to values we previously reported to be associated with poor patient outcomes in a more diverse group of endometrial tumors (31). Because of the small cohort size, we were not statistically powered for performing survival analyses.

### Loss of CD73 associates with β-catenin nuclear localization in exon 3 CTNNB1-mutant tumors.

We found a strong correlation between CD73 expression and β-catenin localization in exon 3 *CTNNB1*-mutant tumors, supporting our hypothesis that CD73 may control mutant β-catenin. Immunohistochemical staining for CD73 and β-catenin was performed on *n* = 11 exon 3 *CTNNB1*-mutant EECs. Staining patterns for CD73 and β-catenin for each tumor are shown in Figure 1C, and correlations in Figure 1D. These data show that CD73 and β-catenin correlations varied depending on subcellular localization of the two proteins. A negative correlation between normal CD73 expression (depicted as Membrane^high^ + Cytoplasm^low/none^) and β-catenin was observed when β-catenin was predominantly nuclear, whereas a positive correlation was seen between normal CD73 expression and membrane/cytoplasm-bound β-catenin. Similarly, with CD73 loss (depicted as Cytoplasmic + None) less β-catenin was found at the cell membrane (depicted as Membrane + Cytoplasm; a negative correlation) and more β-catenin was found in the nucleus (a positive correlation). Together, these data suggest that CD73 presence or absence may influence β-catenin localization in *CTNNB1*-mutant ECs.

To directly assess the relationship between CD73 expression and β-catenin localization, we analyzed publicly available GeoMx digital spatial profiling (DSP) transcriptomic data from a cohort of *n* = 16 NGS-confirmed exon 3 *CTNNB1*-mutant ECs. From each tumor, 6 regions of interest (ROIs) were included in the dataset: 3 characterized by non-nuclear β-catenin and 3 by nuclear β-catenin expression. Non-nuclear ROIs represented areas with membrane and cytoplasmic β-catenin staining, consistent with the pattern shown in Figure 1E. Analysis of the GeoMx Human Whole Transcriptome profiles for these ROIs demonstrated significantly higher *CD73* mRNA levels in non-nuclear compared with nuclear ROIs (Figure 1F). This relationship was consistently observed across multiple tumors (Supplemental Figure 1C), despite the limited number of ROIs per group, underscoring the robustness of the association between CD73 and β-catenin localization. We further compared phosphorylation-site versus non-phosphorylation-site mutants (Figure 1G). Both showed elevated *CD73* expression in non-nuclear ROIs relative to nuclear ROIs, with slightly higher levels in non-phosphorylation-site mutants. Together, these findings provide strong evidence that CD73 expression is directly linked to β-catenin localization. These data prompted us to pursue in vitro experiments to determine whether CD73 may control the localization and oncogenic activity of mutant β-catenin in EC.

### Validation of the TOPFlash reporter assay for measuring mutant β-catenin activity in EC cells.

**Figure 2:**
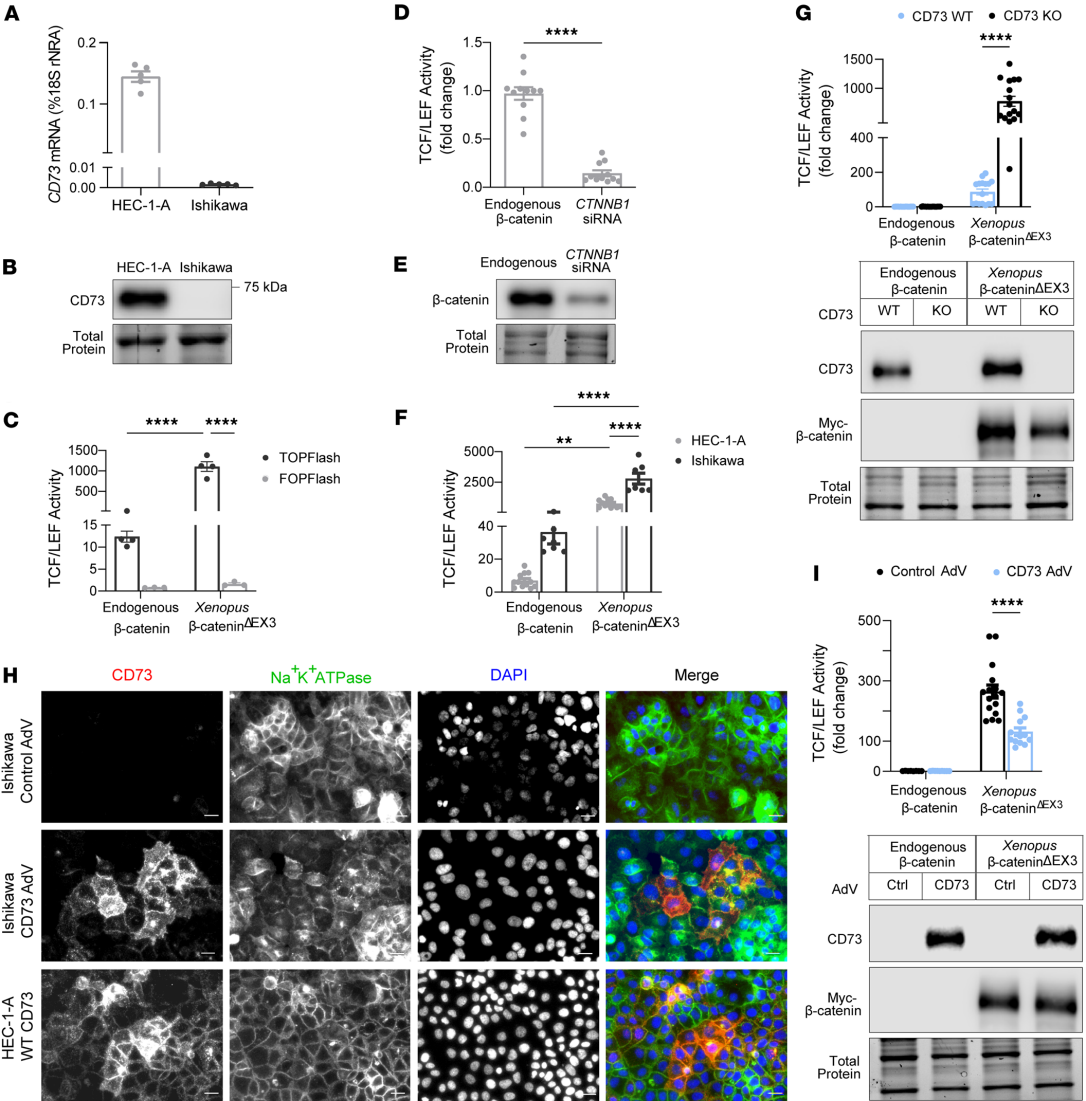
CD73 restrains transcriptional activity of *Xenopus* exon 3 mutant β-catenin. (**A** and **B**) CD73 mRNA and protein in EC cell lines. (**C**) Validation of TCF/LEF–luciferase reporter with TOPFlash/FOPFlash for endogenous and *Xenopus* exon 3 mutant β-catenin (β-catenin^ΔEX3^). (**D** and **E**) Reporter activity and immunoblot after *CTNNB1* siRNA knockdown in HEC-1-A cells. (**F** and **G**) Reporter activity in WT HEC-1-A or Ishikawa cells or CD73-WT versus CD73-KO HEC-1-A cells with and without β-catenin^ΔEX3^. (**G**) Immunoblot: validation of *CD73* deletion and β-catenin^ΔEX3^ expression. (**H**) Immunofluorescence: CD73 localized to membrane in AdV-CD73–transduced Ishikawa cells. HEC-1-A cells were used as positive control. Cropped images are shown (original magnification, ×20; scale bars: 20 μm); originals are in Supplemental Figure 2. (**I**) Reporter activity in Ishikawa cells with and without β-catenin^ΔEX3^ and AdV-CD73 and validation immunoblots. (**C**, **D**, **F**, **G**, and **I**) Mean ± SEM from *n* = 2–3 independent experiments. **P = 0.008, ****P < 0.0001, Mann-Whitney (**D**) or 2-way ANOVA with Šidák’s post hoc test (**C**, **F**, **G**, and **I**).

β-Catenin is a transcriptional cofactor and often complexes with proteins of the T cell factor/lymphoid enhancer factor (TCF/LEF) family of transcription factors to activate the transcription of Wnt target genes (8–10, 13, 41). Accordingly, we performed experiments testing a TOPFlash reporter as a readout of mutant β-catenin transcriptional activity in EC cells (41). The TOPFlash construct contained 8 TCF/LEF binding sites upstream of a luciferase gene promoter (42). HEC-1-A and Ishikawa cells are EC cell lines commonly used to model low-grade EC and express high or low/no levels of CD73, respectively (Figure 2, A and B) (31). We also used a multi-site exon 3 *CTNNB1* *Xenopus* mutant (*Xenopus* β-catenin^ΔEX3^) to test the induction of TCF/LEF reporter activity (43). *Xenopus* *CTNNB1* is 97% homologous to the human *CTNNB1* gene and 100% homologous in the exon 3 region (UniProt, P35222 and P26233). Our validation experiments showed that both endogenous β-catenin and *Xenopus* β-catenin^ΔEX3^ induced TCF/LEF reporter activity in HEC-1-A and Ishikawa cells (Figure 2, C and F), with *Xenopus* β-catenin^ΔEX3^ showing a greater induction (Figure 2, C and F). TCF/LEF activity was not induced in cells transfected with FOPFlash, a construct with mutated TCF/LEF sites (Figure 2C) (42), and was reduced by *CTNNB1* siRNA (Figure 2, D and E), demonstrating that the luciferase signals of the TOPFlash reporter were specific to β-catenin.

### CD73 restrains exon 3 Xenopus β-catenin mutant transcriptional activity.

With the successful validation of the TCF/LEF reporter assay in EC cells, we next examined whether CD73 loss alters the transcriptional activity of mutant β-catenin. We measured *Xenopus* β-catenin^ΔEX3^ reporter gene activity in CD73-WT and CD73-knockout (CD73-KO) HEC-1-A cells. The CD73-KO HEC-1-A cell line was generated by CRISPR/Cas9–mediated editing of *NT5E*, the gene encoding CD73. Ectopically expressed *Xenopus* β-catenin^ΔEX3^ induced approximately 10-fold higher levels of TCF/LEF reporter activity in CD73-KO cells when compared with CD73-WT cells (Figure 2G), indicating that CD73 limits transcriptional activity of exon 3 mutant β-catenin. CD73 status and equivalent expression of *Xenopus* β-catenin^ΔEX3^ in CD73-WT and CD73-KO cells were confirmed by immunoblotting (Figure 2G).

Next, we tested the effect of ectopically expressed CD73 on β-catenin–driven TCF/LEF activity in Ishikawa cells (which lack endogenous CD73). We used a CD73 adenoviral vector (AdV) to reconstitute CD73 expression in Ishikawa cells. We ensured that Ishikawa cells were reconstituted with a level of CD73 expression that was equivalent to endogenous CD73 levels in HEC-1-A CD73-WT cells (Supplemental Figure 2A). As shown in Figure 2H, Ishikawa cells transduced with CD73 AdV showed a pattern of membrane-localized CD73, which was similar to the distribution of endogenous CD73 in HEC-1-A CD73-WT cells (Supplemental Figure 2C).

We measured the effect of reconstituted CD73 on *Xenopus* β-catenin^ΔEX3^–dependent transcriptional activity of the TCF/LEF reporter construct. Consistent with a role of CD73 in restraining mutant β-catenin activity, *Xenopus* β-catenin^ΔEX3^–driven TCF/LEF reporter activity was reduced 2-fold in Ishikawa cells reconstituted with CD73 when compared with control cultures (Figure 2I). Reconstitution of CD73 protein levels and equivalent expression of *Xenopus* β-catenin^ΔEX3^ expression in control and CD73-complemented cells was confirmed by immunoblotting (Figure 2I and Supplemental Figure 2B). Taken together, these data suggest a critical role for CD73 in controlling mutant β-catenin transcriptional activity in EC.

### Selection of patient-specific β-catenin mutants from public databases for study in EC cells.

**Figure 3:**
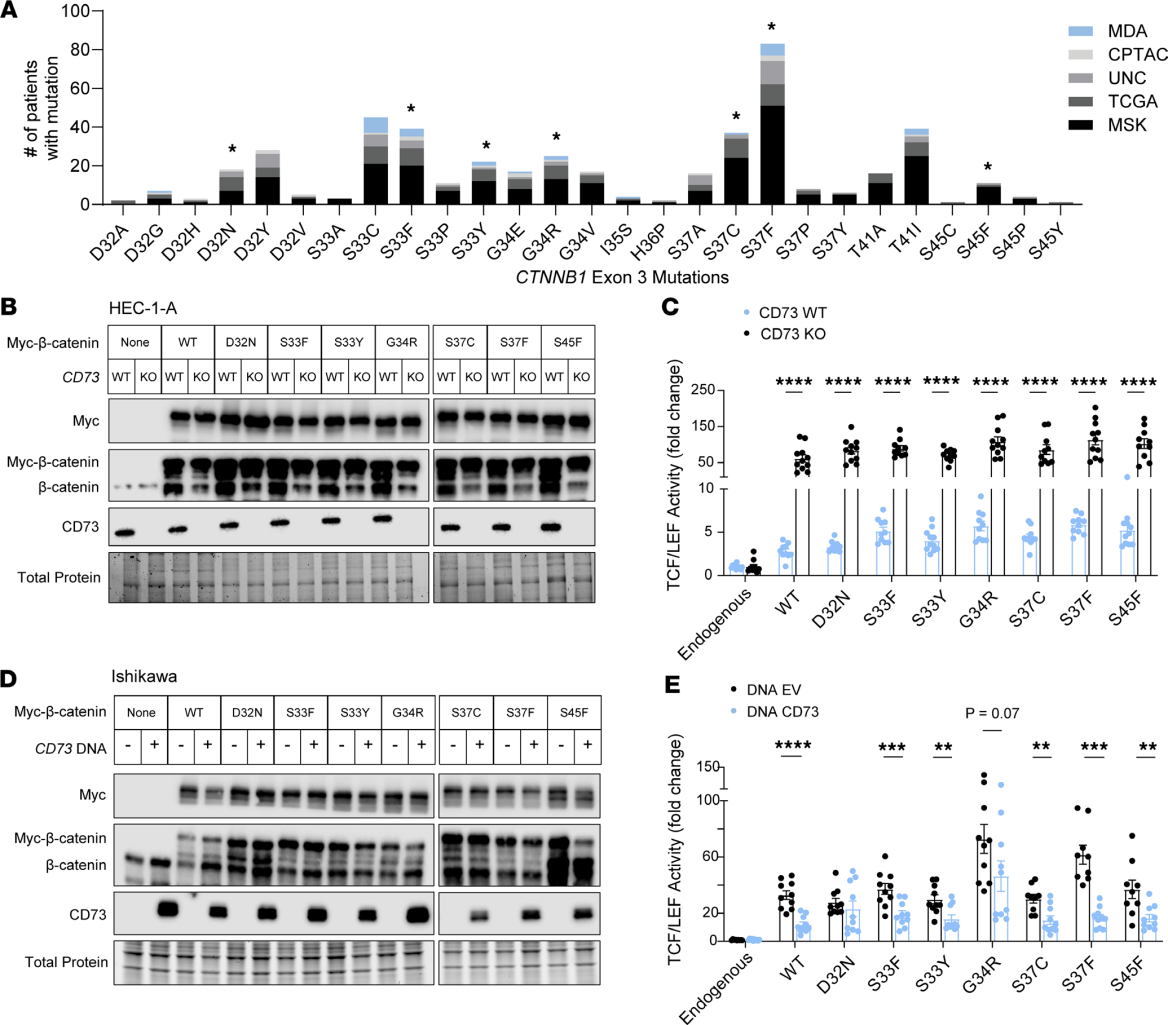
CD73 restrains transcriptional activity of patient-specific β-catenin mutants. (**A**) Variety and frequency of exon 3 *CTNNB1* missense mutations across 5 EC cohorts: The Cancer Genome Atlas (TCGA), Memorial Sloan Kettering Cancer Center (MSK), the Clinical Proteomic Tumor Analysis Consortium (CPTAC), the University of North Carolina (UNC), and the University of Texas MD Anderson Cancer Center (MDA). Asterisks indicate patient-specific *CTNNB1* mutations for which expression vectors were developed and used in our studies. (**B** and **D**) Immunoblots of Myc-tagged WT and patient-specific β-catenin mutants with and without CD73 in HEC-1-A and Ishikawa cells. (**C** and **E**) TCF/LEF reporter activity of WT and patient-specific β-catenin mutants in HEC-1-A and Ishikawa cells; Ishikawa cells were transfected with AdV-CD73 or empty vector. (**B** and **D**) Representative of 2 experiments. (**C** and **E**) Pooled data from *n* = 2 independent experiments (5–6 replicates each). Mean ± SEM. **P < 0.01, ***P < 0.0005, ****P < 0.0001, multiple Mann-Whitney tests.

Having demonstrated that CD73 can restrain the transcriptional activity of *Xenopus* β-catenin^ΔEX3^, a 4-residue mutant, we asked whether CD73 restrains the activity of exon 3 *CTNNB1* mutants that are found in human EC with only 1 mutated codon. An additional consideration was the wide variety of missense mutations in exon 3 of *CTNNB1* reported for EC (16, 17, 44, 45). To select the most patient-relevant mutants to develop expression constructs, mutational data were pooled from 5 patient cohorts, and variety and frequency of the mutations were graphed (Figure 3A). As expected, the most commonly mutated codons were S37 and S33, which are residues that when phosphorylated by glycogen synthase kinase-3β initiate the degradation of β-catenin (43, 46–49). Non-serine/threonine residues D32 and G34 were also highly mutated. Mutations at these sites interfere with β-catenin ubiquitination and subsequent degradation by reducing both β-catenin binding to E3 ligase β-TrCP and β-TrCP–mediated ubiquitination of β-catenin (50, 51). We selected 7 mutants to test for negative regulation by CD73: phosphorylation-site mutants S33F, S33Y, S37C, S37F, and S45F and non-phosphorylation-site mutants D32N and G34R. We additionally included WT *CTNNB1*, as overexpression of β-catenin is oncogenic and is found in EC (16, 52–54).

### CD73 restrains the transcriptional activity of patient-specific exon 3 β-catenin mutants.

Using plasmid transfection, we successfully expressed C-terminal Myc-tagged forms of all 7 patient-specific β-catenin mutants (and WT β-catenin) in HEC-1-A CD73-WT and CD73-KO cells (Figure 3B). Similar to our data with *Xenopus* β-catenin^ΔEX3^, CD73 loss led to approximately 20-fold increased TCF/LEF reporter activity in response to WT β-catenin and all 7 patient-specific β-catenin mutants (Figure 3C). Thus, CD73 downregulation not only increases mutant β-catenin activity in *CTNNB1*-mutant tumors but potentially promotes the oncogenic activity of WT β-catenin in EC.

In a reciprocal experiment, ectopic expression of CD73 in Ishikawa cells (which lack endogenous CD73; Figure 3D) led to the 2- to 4-fold repression of β-catenin–driven TCF/LEF activity with most patient-relevant mutants, as well as WT β-catenin (Figure 3E). Restraining of WT β-catenin transcriptional activity by CD73 is consistent with our previous studies showing that CD73 can control the localization of WT β-catenin to the cell membrane (31). Thus, CD73 likely limits the oncogenic activity of both aberrantly expressed WT β-catenin and exon 3 β-catenin in EC. G34R exhibited the most variability (Figure 3E). Additional independent experiments confirmed that ectopic CD73 suppressed its TCF/LEF activity (Supplemental Figure 3).

### CD73 sequesters exon 3 mutant β-catenin to the cell membrane.

**Figure 4:**
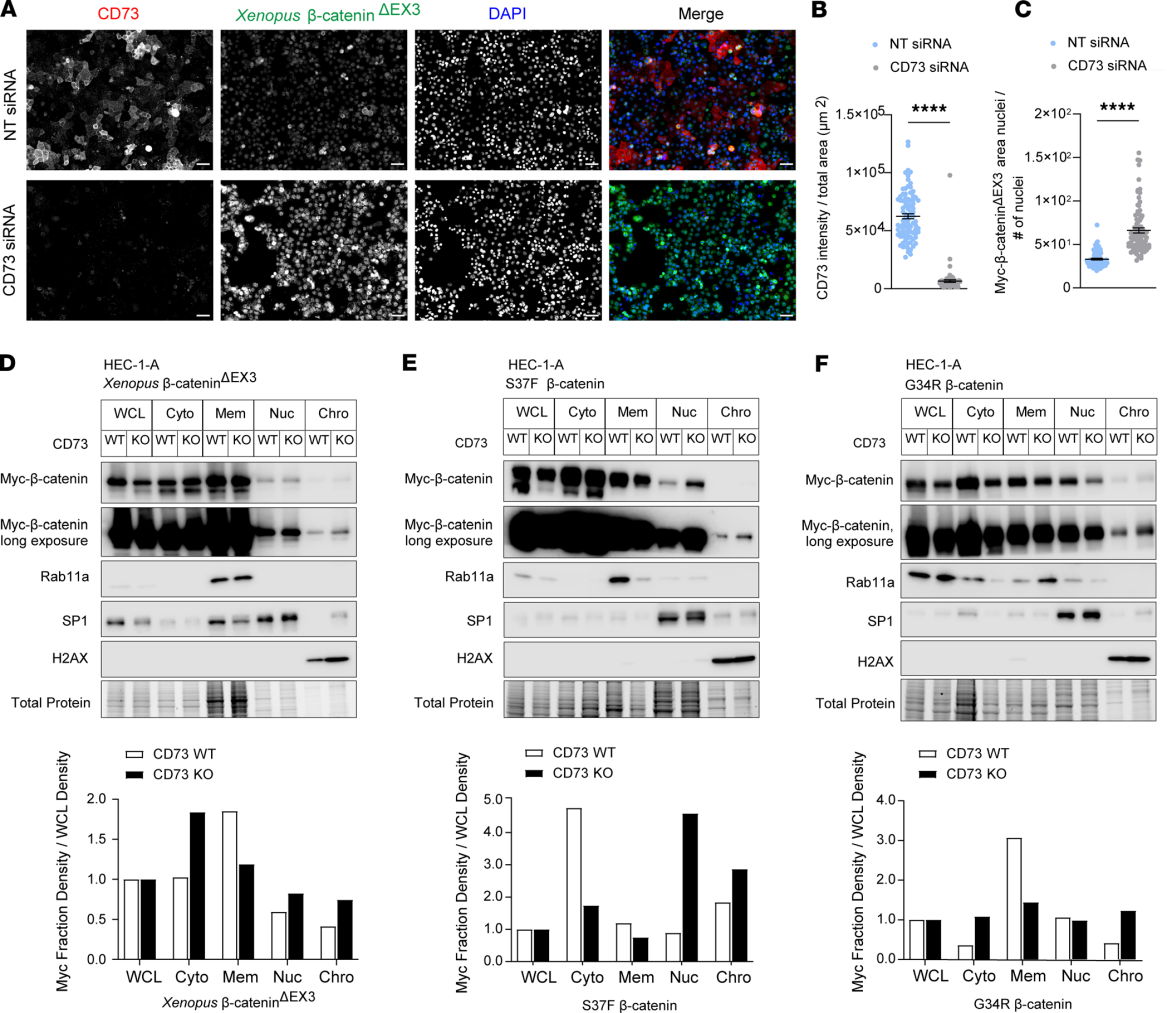
CD73 sequesters exon 3 mutant β-catenin to the cell membrane. (**A**–**C**) HEC-1-A cells were transfected with β-catenin^ΔEX3^ and non-targeting (NT) or CD73 siRNA under 1% O_2_/5% CO_2_ for 48 hours. (**A**) Immunofluorescence of nuclear β-catenin^ΔEX3^ (scale bars: 50 μm), quantified with BZ-X800 Analyzer Macro Cell Count software (Keyence) as shown in **C**. (**B**) CD73 knockdown validation. (**C**) Nuclear fluorescence intensity of Myc-tagged β-catenin^ΔEX3^. (**D**–**F**) Representative immunoblots of *n* = 2–4 independent cellular fractionation experiments with CD73-WT versus -KO HEC-1-A cells transfected with β-catenin^ΔEX3^ (**D**), S37F (**E**), or G34R (**F**) mutants. Densitometry normalized to Myc–β-catenin mutant expression in whole-cell lysate (WCL) and total protein; fractionation markers: Rab11a (membrane), SP1 (nuclear), H2AX (chromatin). (**Β** and **C**) Mean ± SEM. ****P < 0.0001, Mann-Whitney.

Given that exon 3 encodes a region on the N-terminus of β-catenin, which is separate from the region where β-catenin binds with E-cadherin (33–36), we hypothesized that CD73 may restrain exon 3 β-catenin–mutant transcriptional activity by sequestering it to the cell membrane. As proof of principle, we first assessed the nuclear localization of *Xenopus* β-catenin^ΔEX3^ in HEC-1-A cells treated with CD73-directed siRNA (or control non-targeting siRNA). Consistent with our hypothesis, knockdown of CD73 led to increased nuclear *Xenopus* β-catenin^ΔEX3^ (Figure 4, A–C), as detected by immunofluorescence microscopy. To further test our hypothesis, cellular fractionations and immunoblotting were performed to compare the levels of mutant β-catenin in the different cellular compartments of CD73-WT and -KO HEC-1-A cells. Fully consistent with the results of our immunofluorescence microscopy experiments, we observed that in CD73-KO HEC-1-A cells, mutant β-catenin membrane levels were decreased up to 2-fold, whereas nuclear and chromatin-bound levels were increased, compared with those in CD73-WT HEC-1-A cells (Figure 4, D–F, and Supplemental Figure 4, A–E).

A 2-fold increase in nuclear mutant β-catenin and a 3-fold increase in chromatin-bound mutant β-catenin were seen with *Xenopus* β-catenin^ΔEX3^ (Figure 4D and Supplemental Figure 4A) and patient-specific β-catenin mutants, S37F (Figure 4E and Supplemental Figure 4B) and G34R (Figure 4F and Supplemental Figure 4, C–E). We accounted for the random variability in mutant β-catenin expression in CD73-KO versus -WT cells (Figure 4, D–F, and Supplemental Figure 4, A–E) in our calculations by normalizing the expression of mutant β-catenin in each cell compartment to mutant β-catenin expression in the whole-cell lysate for each cell type. Subtle variations in fraction densities were observed for G34R. However, similarly to luminometry results, these effects did not persist across replicates (Supplemental Figure 4, C–E). Altogether, these data suggest that CD73 restrains mutant β-catenin activity by sequestering it to the cell membrane.

Consistent with this interpretation, we show in Supplemental Figure 5A that patient-specific mutant β-catenin did bind with E-cadherin in CD73-KO and -WT HEC-1-A cells. Here, we observed that total and phosphorylated E-cadherin levels were noticeably lower in CD73-KO cells than in CD73-WT cells (Supplemental Figure 5B). Phosphorylated E-cadherin, particularly its cytoplasmic region, is important for the increased affinity for β-catenin binding and subsequent prevention of the binding of TCF transcription factors (2, 55). The difference in E-cadherin levels between CD73-KO and -WT HEC-1-A cells prompted us to investigate the expression of other cell-cell adhesion genes. CD73-KO versus -WT cells showed decreased expression of several genes that assemble and stabilize cell-cell adhesions, including α-catenin (Supplemental Figure 5C), which stabilizes E-cadherin–β-catenin complexes at the cell membrane (56–58). Thus, the ability of CD73 to globally promote epithelial integrity likely partially explains the role of CD73 in restraining mutant β-catenin oncogenic activity.

### CD73 restrains mutant β-catenin transcriptional activity through adenosine A1 receptor activity.

**Figure 5:**
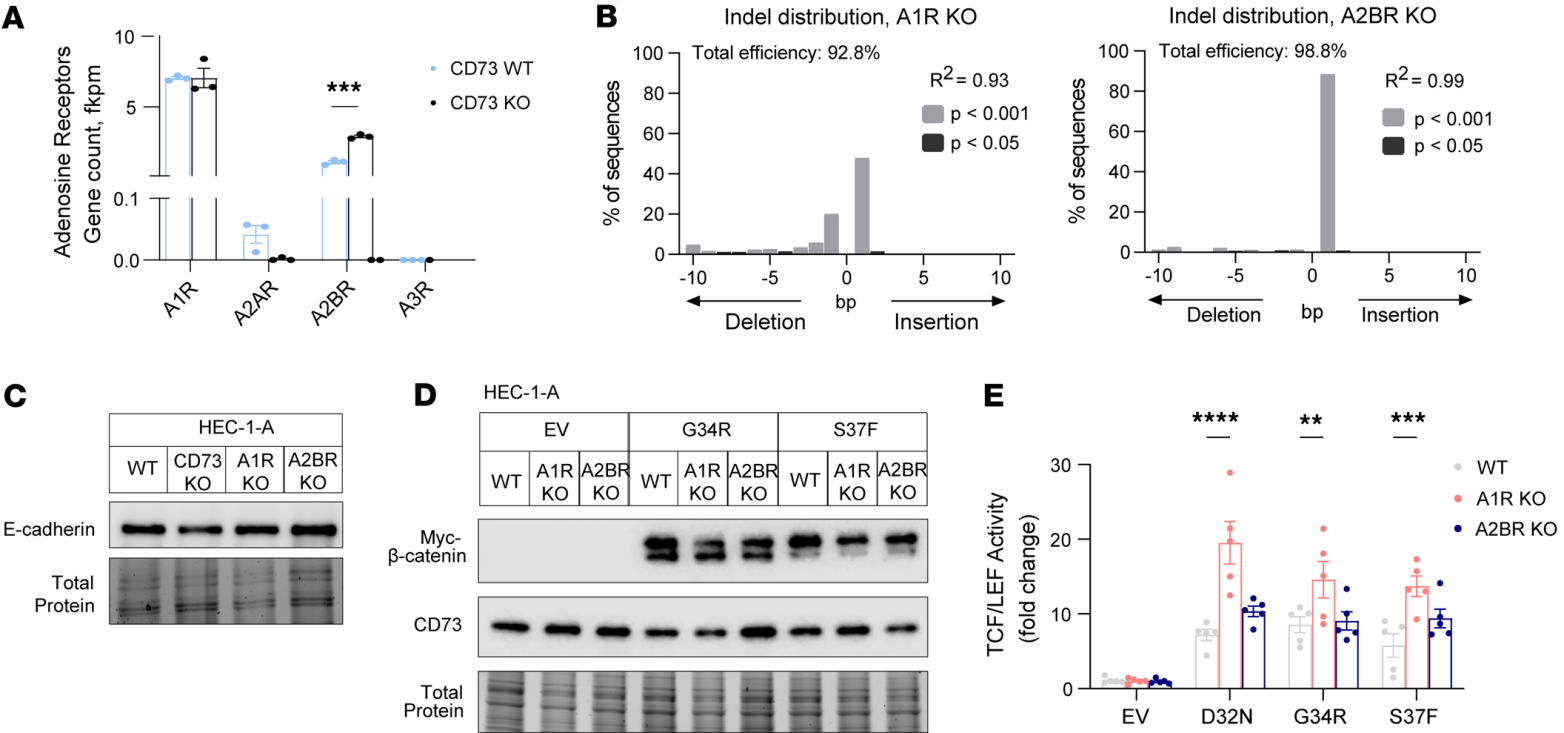
Adenosine A1 receptor signaling restrains transcriptional activity of patient-specific β-catenin mutants. (**A**) Adenosine receptor mRNA in *CD73*-WT versus -KO HEC-1-A cells. (**B**) TIDE (tracking of indels by decomposition) analysis of cells with CRISPR/Cas9 knockout of *ADORA1* (A1R) and *ADORA2B* (A2BR). (**C**) Immunoblots show epithelial integrity (E-cadherin) maintained in adenosine receptor–KO cells. (**D**) Comparable expression of Myc-G34R and Myc-S37F mutant β-catenin in WT, *A1R*-KO, and *A2BR*-KO cells. (**E**) TCF/LEF reporter activity with empty vector (EV), D32N, G34R, or S37F mutant β-catenin. Each dot represents a technical replicate. Graph is representative of *n* = 3 independent experiments; independent experiments are shown in Supplemental Figure 6. (**A** and **E**) Mean ± SEM. *P < 0.05, **P < 0.01, ***P < 0.0005, ****P < 0.0001, 2-way ANOVA with Dunnett’s post hoc test.

To test whether a CD73/adenosine signaling axis regulates mutant β-catenin activity, we generated HEC-1-A cells with CRISPR/Cas9 deletion of adenosine receptors, A1R (gene: *ADORA1*) and A2BR (gene: *ADORA2B*) (Figure 5, A and B). HEC-1-A cells primarily express 2 (A1R and A2BR) of the 4 adenosine receptors (Figure 5A). Therefore, we focused on A1R and A2BR activity. Notably, A1R-KO and A2BR-KO HEC-1-A cells expressed similar E-cadherin levels when compared with WT HEC-1-A cells (Figure 5C and Supplemental Figure 6A).

We measured TCF/LEF reporter activity in response to ectopically expressed patient-specific β-catenin mutants (D32N, G34R, and S37F) in A1R-KO, A2BR-KO, and WT HEC-1-A cells. The mutants were evenly expressed in the different cell lines (Figure 5D). D32N led to an approximately 7-fold increase in TCF/LEF reporter gene activity relative to empty vector (EV) in WT cells. In A1R-KO cells, D32N induced an approximately 20-fold increase in TCF/LEF reporter activity. In A2BR-KO cells, the magnitude of mutant β-catenin–driven TCF/LEF activity was similar to that in WT HEC-1-A cells (Figure 5E). Qualitatively similar results were observed for the G34R and S37F β-catenin mutants (Figure 5E and Supplemental Figure 6, B and C). Together these data show that, similarly to CD73 deficiency, A1R loss leads to derepression of mutant β-catenin transcriptional activity.

### Ectopic CD73 suppresses exon 3 mutant β-catenin EC cell invasiveness.

**Figure 6:**
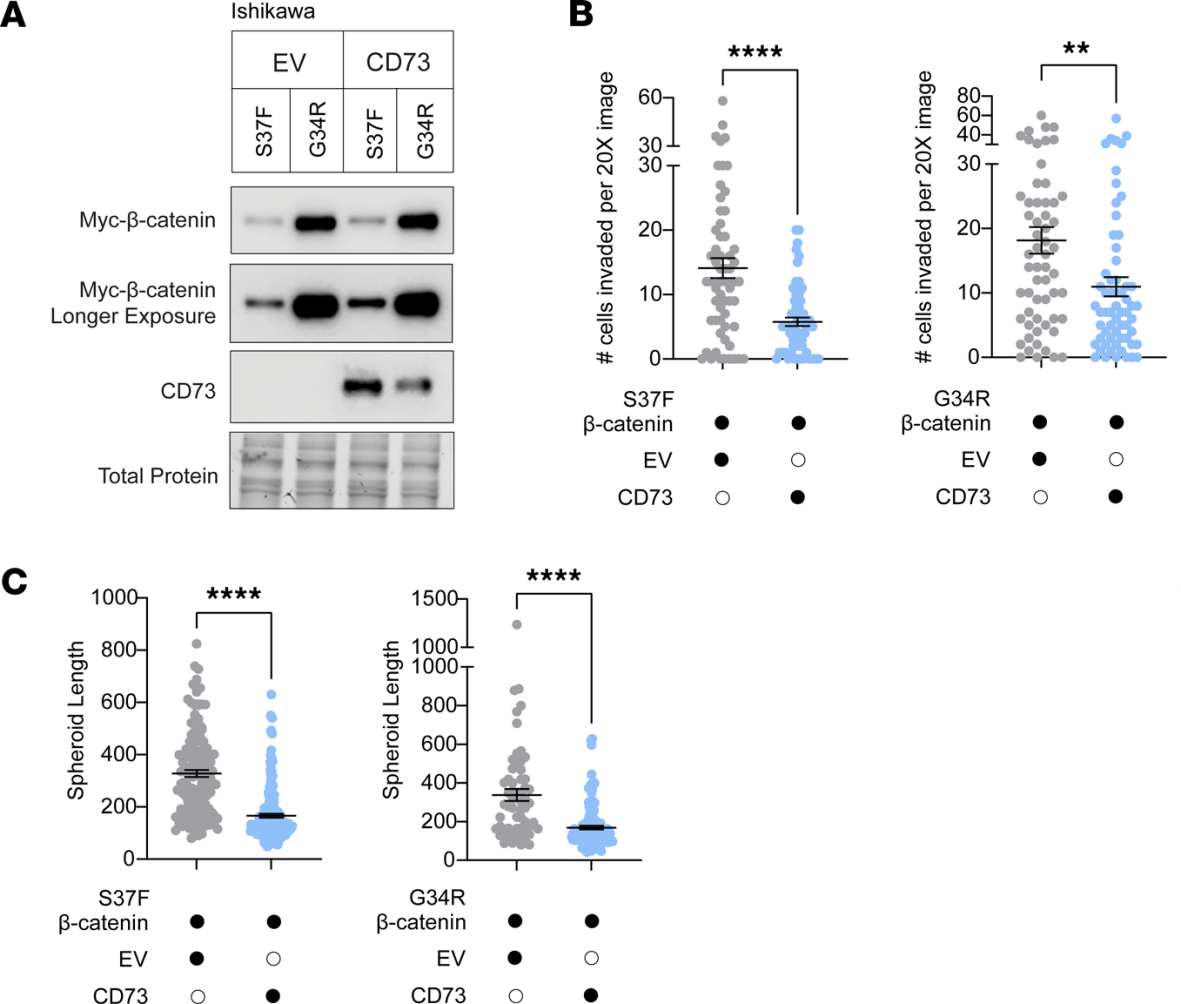
Ectopic CD73 suppresses invasiveness and stemness of exon 3 mutant β-catenin–expressing EC cells. (**A**) Immunoblot of isogenic Ishikawa cells with stable expression of empty vector (EV) or CD73 and G34R or S37F β-catenin mutants. (**B** and **C**) Transwell invasion (**B**) and spheroid formation (**C**) assays. (**B** and **C**) Representative of *n* = 2 independent experiments per mutant (see Supplemental Figure 7 for the second independent experiment). Mean ± SEM. **P < 0.005, ****P < 0.0001, 2-tailed Mann-Whitney *t* test.

To further validate the effect of CD73 on restraining mutant β-catenin, we developed isogenic Ishikawa cells in which we stably expressed CD73 or EV, and then stably expressed 1 of 2 exon 3 β-catenin mutants (S37F or G34R) (Figure 6A). Stable ectopic expression of CD73 in Ishikawa cells led to about 60% reduction in the invasiveness of the cells expressing the S37F mutant compared with EV S37F–expressing cells (Figure 6B and Supplemental Figure 7A). Similar results were found with the G34R mutant. CD73 expression suppressed the invasiveness of G34R-expressing cells by about 60% in comparison with EV G34R–expressing cells (Figure 6B and Supplemental Figure 7A).

### Ectopic CD73 suppresses stemness capacity of exon 3 mutant β-catenin–expressing EC cells.

Recurrence in patients with β-catenin–mutant EC arises approximately 3 years after initial diagnosis (24, 59), suggesting that disseminated cancer cells persist in a stem cell–like state. To test whether CD73 loss promotes stemness, we performed spheroid-forming assays, which assess the ability of individual cells to survive, self-renew, and grow in suspension culture — a functional readout of stemness capacity (60). Ishikawa cells with stable ectopic expression of CD73 and β-catenin–mutant S37F exhibited an approximately 50% reduction in spheroid growth compared with EV S37F–expressing cells (Figure 6C and Supplemental Figure 7B). A similar reduction (~50%) in spheroid size was observed with G34R; CD73-proficient cells formed smaller spheroids compared with EV G34R–expressing cells (Figure 6C and Supplemental Figure 7B). These findings support a role for CD73 in limiting the stemness capacity of β-catenin–mutant EC cells.

### CD73 loss in CTNNB1-mutant EC drives distinct Wnt signaling/TCF/LEF–dependent target gene expression.

Because CD73 loss strongly induced TCF/LEF reporter activity in vitro, we next asked whether CD73 loss broadly enhances Wnt signaling/TCF/LEF target gene expression or preferentially affects a subset of these factors in *CTNNB1*-mutant EC. To address this, we analyzed the transcriptomic profiles of 2 different target gene lists in early-stage *CTNNB1*-mutant and -WT endometrial tumors from the Uterine Cancer TCGA.

**Figure 7:**
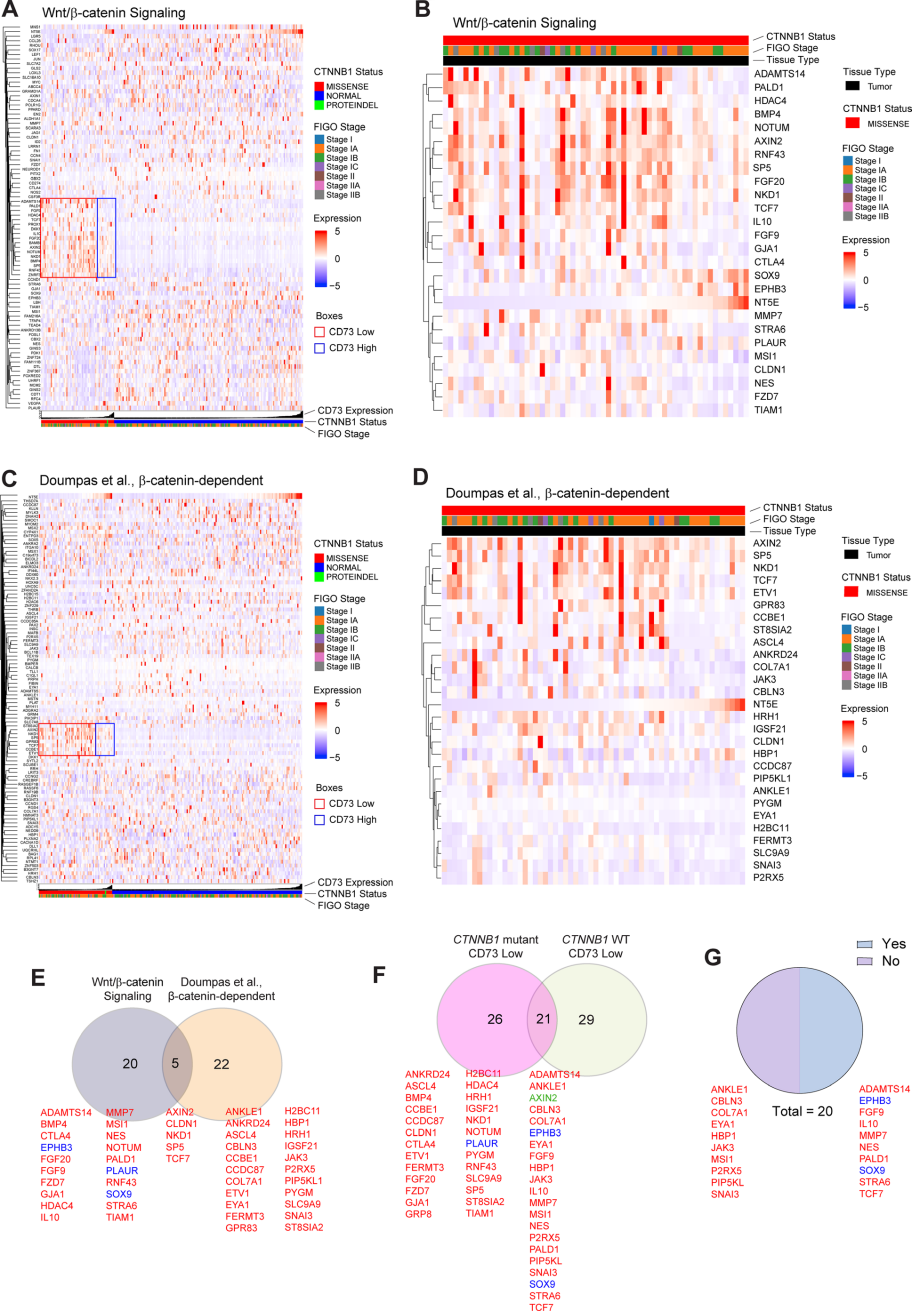
CD73 loss drives expression of a distinct subset of Wnt–TCF/LEF target genes in *CTNNB1*-mutant EC. (**A** and **B**) Computational analyses of TCGA Uterine Corpus Endometrial Carcinoma (UCEC) RNA-Seq data in early-stage, *CTNNB1*-mutant and -WT tumors. Heatmaps are from 2 different gene lists: Wnt/β-catenin signaling and β-catenin–dependent, TCF/LEF–dependent (Supplemental Table 3). Tumors are stratified by *CD73*-high (upper *NT5E* quartile) versus -low tumors (all other quartiles for *NT5E* expression). (**C** and **D**) Heatmaps of genes with *P* value less than 0.05; Welch’s test. (**E**) Venn diagram of altered Wnt signaling and β-catenin–dependent, TCF/LEF–dependent target genes (red, up; blue, down). (**F**) Venn diagram showing genes specifically dysregulated by CD73 loss in *CTNNB1*-mutant versus -WT tumors; AXIN2 has opposite regulation (green). (**G**) Proportion of overlapping genes (20/21; AXIN2 excluded) with amplified activation (Yes) or no amplification (No) in *CTNNB1*-mutant, *CD73*-low tumors versus *CTNNB1*-WT, *CD73*-low tumors.

*CTNNB1*-mutant tumors exhibited increased expression of only a subset of Wnt/β-catenin signaling (Figure 7A) and β-catenin–dependent, TCF/LEF–dependent target genes (Figure 7B) compared with *CTNNB1*-WT tumors. Remarkably, when stratified by CD73 expression, CD73 loss selectively amplified these genes (Figure 7, A and B, red and blue boxes), along with a few additional targets (included in Figure 7, C and D), while leaving the majority of Wnt genes unaffected (Figure 7, A and B). Genes significantly altered for each list are shown in greater detail in Figure 7, C and D. The Wnt/β-catenin signaling list of genes was compiled from various publications. The β-catenin–dependent, TCF/LEF–dependent gene list from Doumpas et al. (61), which reported gene targets of β-catenin based on chromatin immunoprecipitation and TCF deletion in 293T cells, was used as a second, largely non-overlapping target gene list. These data indicate that CD73 restrains the transcriptional output of mutant β-catenin, and its loss specifically enhances the expression of genes most sensitive to mutant β-catenin activity, a subset that may drive critical oncogenic programs in *CTNNB1*-mutant endometrial tumors.

Importantly, these effects were not explained by differences in *CD73* levels in tumor tissue (Supplemental 8A) and between genotypes (Supplemental Figure 8B), nor were they observed with *CD73* stratification alone (Supplemental Figure 8, C and D). In total, 47 genes were identified as significantly altered as a result of CD73 deficiency, of which almost all were upregulated (Figure 7E). Of these genes, 26 were found to be unique to *CD73* loss in *CTNNB1*-mutant tumors (Figure 7F), while 21 were identified to also be altered in *CTNNB1*-WT tumors with CD73 loss (Figure 7F). Notably, half of these genes showed a greater induction in *CTNNB1*-mutant tumors with CD73 loss (Figure 7G). Together, these data reveal that CD73 loss induces a distinct Wnt signaling/TCF/LEF–dependent transcriptional program, characterized by coordinated and amplified activation of target genes.

### Target genes induced by CD73 loss are amplified in nuclear β-catenin–positive cancer cells of exon 3 CTNNB1-mutant tumors.

**Figure 8:**
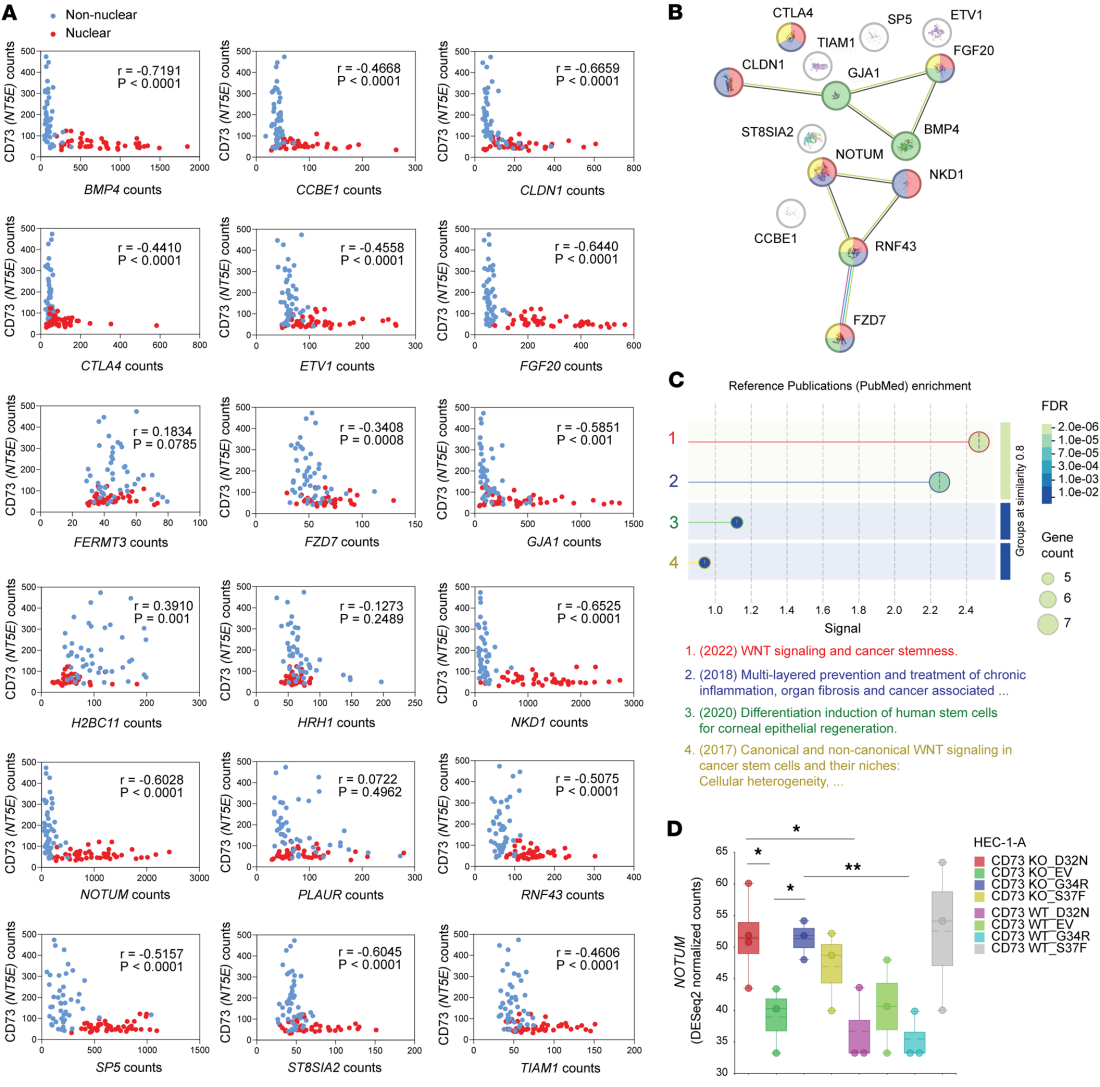
Spatial and functional analyses show that CD73 loss activates a stemness-associated Wnt–TCF/LEF transcriptional program. (**A**) Spearman’s correlations of *CD73* mRNA with Wnt–TCF/LEF target genes specifically altered by CD73 loss in *CTNNB1*-mutant versus -WT tumors (Figure 7F), using GeoMx spatial profiling whole-transcriptome data of nuclear versus non-nuclear ROIs of *n* = 16 exon 3 *CTNNB1*-mutant ECs. Transcriptome data were unavailable for 8 of 26 genes. (**B**) STRING network of 14 genes with significant negative correlations from **A**. (**C**) STRING enrichment analysis of reference publications (top 4 shown, color-coded to **B**). (**D**) In vitro validation: *NOTUM* upregulation in CD73-isogenic HEC-1-A cells with β-catenin mutants. One-way Welch’s ANOVA followed by Welch’s 2-tailed *t* tests. *P < 0.05, **P < 0.005.

Nuclear expression of β-catenin in *CTNNB1*-mutant tumors is highly variable (~5%–60%). Accordingly, we leveraged spatial transcriptomic data, described in Figure 1, which used an independent cohort of *n* = 16 exon 3 *CTNNB1*-mutant tumors, to determine whether the target genes induced by CD73 loss, which were identified by bulk transcriptomic data of *CTNNB1*-mutant tumors, are specifically induced in cancer cells with nuclear β-catenin expression. Of the 26 genes unique to CD73 loss, identified in Figure 7F, 18 were present in the GeoMx DSP dataset. Nonlinear, negative correlations were observed between *CD73* expression and many of the target genes, with several having a strong (≥ –0.6) negative relationship, including *BMP4*, *CLDN1*, *FGF20*, *NOTUM*, and *NKD1* (Figure 8A). High CD73 corresponded to minimal target gene expression and was entirely observed in ROIs with non-nuclear β-catenin. Conversely, low *CD73* corresponded with marked increase in target gene expression and was predominantly seen in ROIs with nuclear β-catenin expression (Figure 8A). The L-shaped relationship of the correlation graphs is consistent with a threshold-dependent effect, in which CD73 loss permits robust transcription of Wnt signaling/TCF/LEF–dependent target genes.

### CD73 loss induces a Wnt signaling/TCF/LEF–mediated transcriptional stemness program.

Considering that the selective amplification of Wnt/β-catenin/TCF/LEF target genes could be functionally important for controlling critical oncogenic programs, we used STRING to objectively map these genes to known interactions and networks. STRING-related enrichment analyses additionally were used to reveal the biological context of these genes, beyond Wnt/β-catenin signaling. STRING identified 2 major clusters of the 14 genes that were found to have significant negative correlations with CD73 (Figure 8B). Enrichment analysis of recent PubMed references strongly aligned these gene targets with biological activity in cancer stemness (Figure 8C), with multiple genes being recognized across different publications for stemness roles (Figure 8C) (62–65). These data are consistent with our in vitro spheroid formation data in Figure 6, which showed that CD73 loss functionally increases the stemness capacity of β-catenin mutant–expressing EC cells.

To functionally test whether CD73 loss directly induces the expression of these genes, we assessed *NOTUM* expression in our CD73-isogenic HEC-1-A cells (Figure 8D and Supplemental Figure 9). Expression of patient-specific β-catenin mutants in HEC-1-A WT cells did not increase *NOTUM* levels (Figure 8D). In contrast, β-catenin mutant expression in CD73-KO cells strongly induced *NOTUM* (Figure 8D), mirroring the *NOTUM* expression pattern observed in cancer cells with nuclear β-catenin and low CD73 expression in human tumors (Figure 8A). Altogether, these findings strongly indicate CD73 as a critical regulator of mutant β-catenin transcriptional activity in EC, and that its absence in *CTNNB1*-mutant tumors reshapes the transcriptional landscape to promote features essential for recurrence.

## Discussion

Our study identifies CD73 as a key regulator of oncogenic β-catenin activity in exon 3 *CTNNB1*-mutant EC. We demonstrate that low CD73 expression is associated with disease recurrence, that CD73 restrains mutant β-catenin transcriptional activity by sequestering it at the membrane, that CD73 loss increases β-catenin mutant–expressing EC cell invasiveness and stemness capacity, and that CD73 loss in *CTNNB1*-mutant tumors selectively amplifies a stemness-associated Wnt/β-catenin–dependent transcriptional program. Together, these findings provide mechanistic insight into why only a subset of *CTNNB1*-mutant ECs recur, and suggest future evaluation of CD73 as a potential biomarker of recurrence risk.

Recent studies highlight the value of molecular testing in guiding EC management, with 4 molecular subtypes now recognized (26, 66–76). Tumors with *CTNNB1* mutations largely fall into the “no specific molecular profile” (NSMP) subtype, a clinically heterogeneous group that includes both indolent and high-risk disease but lacks reliable prognostic markers. Although *CTNNB1* mutation status was initially expected to be informative, it has not proven sufficient to guide clinical decisions, and no alternative markers currently exist to stratify risk in these patients. Our data show that patients with low *CD73* expression were significantly more likely to experience recurrence or die, independent of stage or lymphovascular invasion. This positions CD73 as a potential clinically relevant biomarker that could refine recurrence risk stratification in *CTNNB1*-mutant EC. Future large-scale studies with CD73 are necessary to validate these findings and establish clinical utility. Studies like these are particularly important given recent evidence that adjuvant therapy can improve outcomes for patients with tumors harboring *CTNNB1* mutations or aberrant β-catenin expression (59, 77), whereas clinical surveillance remains the current standard of care for these patients.

To our knowledge, CD73 to sequester mutant β-catenin at the cell membrane is a novel regulatory mechanism for a major oncogene in human tumors. We anticipate that this action is cancer type specific, as CD73 is downregulated in EC (31, 32), whereas in other human cancers, CD73 is overexpressed (78–81). Exon 3 *CTNNB1* mutations are also found in hepatocellular and prostate carcinomas (82–84). Both tumors are reported to downregulate CD73 expression or its enzymatic activity (85–87). Therefore, an oncogenic mechanism involving derepression of mutant β-catenin by CD73 loss may be particularly relevant to these tumors. We also consider that mutant-specific differences will be present. Variability in CD73-A1R to control D32N was observed. Subtle effects on G34R were seen; however, the small variability observed was not consistent across replicates and different experimental protocols and therefore did not affect overall conclusions. The possibility that different β-catenin mutants may be differently controlled by CD73 is not unexpected given that different exon 3 *CTNNB1* mutants have been demonstrated in other cancers to have functional differences (e.g., proliferation and migration capacity), and that protein structure of β-catenin mutants (e.g., electrostatic charge, polar interactions, and stability) is differentially affected by certain amino acid changes (88–91). We have observed in RNA sequencing (RNA-Seq) data that different β-catenin mutants in HEC-1-A cells with or without CD73 have shared and mutant-specific gene expression changes (data not shown). Thus, a different milieu of gene expression may differentially affect mutant proteins. Few studies have investigated different β-catenin mutants in EC biology (88, 92), and it remains largely unclear mechanistically whether different mutations may have different biology that may further explain the variability in disease aggressiveness.

The cell-cell adhesion function of β-catenin, despite being as prominent as its role as a transcriptional cofactor, has been relatively overlooked as a major determinant of mutant β-catenin’s oncogenic potential. Decades of studies have demonstrated that the two functions are independent, and that mutations in exon 3 of *CTNNB1* do not impact β-catenin binding with E-cadherin and reaching the cell membrane (2, 93, 94). More recent studies show that E-cadherin can limit the transforming properties of activating β-catenin mutations (95). Our findings are consistent with these studies and importantly underscore that regulators of β-catenin’s cell-cell adhesion function are as critical in deciding the oncogenicity of mutant β-catenin as its exon 3 mutation.

Whether the ability of CD73 to sequester mutant β-catenin to the membrane is entirely dependent on E-cadherin expression is unclear. E-cadherin expression in low-grade, early-stage EC is variable; E-cadherin staining ranges from 5% to 95% for these tumors (96). N-cadherin, a related cadherin family member, is highly expressed in early-stage EC — including *CTNNB1*-mutant tumor mutations (16) — and, like E-cadherin, binds β-catenin and can inhibit β-catenin/LEF-1 transactivation (97, 98). Although N-cadherin promotes migration, it also mediates stable cell-cell adhesions, a function influenced by posttranslational modifications and interactions with factors such as fibroblast growth factor receptor (99–101). Thus, in some tumors, N-cadherin may act as a molecular sink for mutant β-catenin, and therefore E-cadherin expression may not be a reliable readout of CD73 to restrain mutant β-catenin. Notably, tumor differentiation significantly affects E-cadherin expression in EC, but has no impact on N-cadherin levels (102).

While cell-cell adhesions are advantageous for suppressing the activity of oncogenes, these structures can become saturated. Saturation of adherens junctions by oncogenic β-catenin binding to E-cadherin has been demonstrated in studies using murine models of intestinal cancer (95). Additionally, recent evidence suggests that fold change — not absolute levels — of β-catenin can dictate Wnt signaling (103). Accordingly, expression levels of mutant β-catenin in EC likely also determine the amount of mutant β-catenin that can be sequestered at the cell membrane. These factors may explain why some patients with *CTNNB1*-mutant tumors that retained CD73 expression had disease recurrence (Figure 1). We also consider that the impact of CD73 loss is likely more than losing the localization of mutant β-catenin to the membrane. For example, we found that CD73 loss in human *CTNNB1*-mutant tumors was associated with *SOX9* downregulation. SOX9 is a potent antagonist of Wnt/β-catenin signaling, as it directly binds to and negatively regulates β-catenin activity (52, 104).

Bulk transcriptomic analysis of *CTNNB1*-mutant endometrial tumors revealed no global activation of Wnt/β-catenin signaling or β-catenin–dependent, TCF/LEF–dependent target genes compared with *CTNNB1*-WT tumors. This aligns with the known variability of nuclear β-catenin in endometrial tumors and the context-specific effects of *CTNNB1* mutations and cell type–specific target gene expression (29, 105), and with the findings that β-catenin signaling can be influenced by feedback loops, pathway crosstalk, and other molecular changes (106–108). We found that a restricted set of genes was consistently altered in *CTNNB1*-mutant endometrial tumors, of which, remarkably, over half were specifically associated with CD73 loss. Spatial transcriptomics further pinpointed 14 genes elevated in tumor regions with both CD73 loss and nuclear β-catenin, with functional enrichment linking these genes to stemness.

The association between stemness and recurrence is particularly relevant for *CTNNB1*-mutant ECs, which are prone to late relapse (24, 59). Prior studies in other cancers have connected β-catenin activation with maintenance of cancer stem cell populations (109). Our data indicate that CD73 directly modulates mutant β-catenin’s ability to drive transcriptional programs related to stemness, explaining why CD73 loss correlates with recurrence in *CTNNB1*-mutant EC. Several of the genes amplified by *CD73* loss, including *NOTUM*, *NKD1*, *BMP4*, *CLDN1*, and *FGF20*, have been previously implicated in regulating self-renewal, stemness, and tumorigenicity (62–65). For instance, *NOTUM* has emerged as a potent β-catenin target and regulator of stem cell niches across multiple tissues, with recent work in gastric cancer showing that *NOTUM* overexpression promotes stemness and tumorigenesis (110). In colon cancer, *NOTUM* has been shown to drive stem cells into a quiescent state (111). Therefore, *NOTUM* has the ability to both stimulate cancer stem cell proliferation and maintain stem cells in a resting state, which are equally essential mechanisms for supporting recurrence. *NOTUM* is a direct target for β-catenin/TCF transcription (112).

While our TCGA bulk transcriptomic analysis helped identify genes induced by CD73 loss in *CTNNB1*-mutant EC, it likely underrepresents the full spectrum of β-catenin–dependent transcriptional changes. Our focus was intentionally limited to Wnt signaling and β-catenin–dependent, TCF/LEF–dependent targets with the intent of identifying genes directly driven by mutant β-catenin. Even so, the strong enrichment of Wnt/β-catenin targets linked to CD73 loss underscores CD73’s central role as a gatekeeper of devastating transcriptional programs in β-catenin–mutant tumors.

Overall, our study provides a compelling explanation for the clinical variability of *CTNNB1*-mutant EC: high CD73 expression restrains mutant β-catenin and aligns with indolent behavior, whereas loss of CD73 removes this brake, unleashing β-catenin–mediated transcriptional programs that support recurrence.

## Methods

### Sex as a biological variable.

Our study exclusively examined female tissues and cell lines because endometrial cancer is only relevant in females.

### Human tissues.

All human tissues and/or associated data used in this study originated from previously published tumor cohorts (17, 29, 75, 113). For Figure 1, mRNA and formalin-fixed, paraffin-embedded (FFPE) sections were obtained from tumor cohorts previously described in Kurnit et al. (17) and Kim et al. (29).

### Cell lines.

HEC-1-A cells and CRISPR/Cas9 NT5E (CD73-KO) HEC-1-A cells were purchased from ATCC and maintained in McCoy’s 5A medium with 10% FBS and 1× penicillin/streptomycin (100 U/mL, 100 mg/mL). Ishikawa cells (gift of Changping Zou, University of Arizona, Tucson, Arizona, USA) were cultured in MEM with 10% FBS, 1× penicillin/streptomycin, 1 mM sodium pyruvate, and 0.1 mM non-essential amino acids. All lines were authenticated by the MD Anderson Characterized Cell Line Core and maintained at 37°C, 5% CO_2_.

### Generation of A1R-KO and A2BR-KO cell lines.

CRISPR/Cas9 plasmids targeting *ADORA1* (A1R) or *ADORA2B* (A2BR) (Vector Builder, VB240110) contained sgRNAs (ADORA1: 5′-TCTCCTTCGTGGTGGGACTGA-3′; ADORA2B: 5′-CACAGGACGCGCTGTACGTGG-3′), *Streptococcus* *pyogenes* Cas9, and ampicillin/puromycin resistance. Guides were designed with CHOPCHOP (https://chopchop.cbu.uib.no/). HEC-1-A cells were transfected (2 μg plasmid) and selected with puromycin (2–5 μg/mL over 3 days). After selection, cells were maintained in McCoy’s 5A with 10% FBS and used for experiments at passages 4–13.

### Generation of G34R-CD73 and S37F-CD73 cell lines.

Ishikawa cells (80% confluence) were transfected with G34R or S37F β-catenin plasmids, selected with 800 μg/mL hygromycin for 4 days, and cultured for 3 passages over 4 weeks in standard medium. Myc–β-catenin expression was confirmed by immunoblot. Passage 3 cells were then transfected with CD73 (Vector Builder, VB250113) or empty vector, selected with 800 μg/mL G418 for 5 days, and maintained in MEM. Cells (passages 2–10) were used for invasion and spheroid assays.

### Constructs and reagents.

Patient-specific exon 3 *CTNNB1* mutants (D32N, S33F, S33Y, G34R, S37C, S37F) and WT plasmids (Vector Builder, VB220927 backbone) carried RSV/EF1A promoters, ampicillin/hygromycin resistance, and C-terminal 6×Myc tags. Plasmids were expressed transiently via Lipofectamine 3000 (Invitrogen) unless noted. TOPFlash (Addgene 12456), FOPFlash (Addgene 12457), and Xenopus β-catenin^ΔEX3^ (Addgene 16840, deposited by Randall Moon, provided by Pierre McCrea, University of Texas MD Anderson Cancer Center, Houston, Texas, USA) were used for reporter assays. LentiV_Neo (Addgene 108101) (114) and pACCMV (generated in-house) served as empty vector controls. CMV-pRenilla-LUC (Promega E2261) was used as a control reporter. Plasmids were propagated in *E*. *coli* DH5α and purified with QIAGEN or ZymoPURE kits. siRNA sequences are as follows: non-targeting: 5′-GAUCAUACGUGCGAUCAGATT-3′ (MilliporeSigma); *NT5E*: 5′-CGCAACAAUGGCACAAUUATT-3′, as previously described (31); and *CTNNB1*: 5′-CUCAGAUGGUGUCUGCUAU-3′ (MilliporeSigma). A complete list of antibodies is provided in Supplemental Table 2.

### Design of adenoviral vectors.

The HA-tagged CD73 ORF was cloned into the pACCMV vector (115) and purified (QIAGEN EndoFree Maxi Kit). Adenoviruses were generated by cotransfection of 293T cells with CD73-pACCMV (or empty vector) and pJM17, followed by PEG precipitation, CsCl gradient centrifugation, and gel filtration (115). Cells were infected at 4 × 10^8^ IU/mL. pACCMV-CD73 and pACCMV empty vector were also used for transient transfections.

### TCF/LEF–luciferase reporter assay.

HEC-1-A and Ishikawa cells (2.8 × 10^4^ per well, 96-well plates) were cotransfected with 100 ng TOPFlash/FOPFlash, 100 pg CMV-pRenilla-LUC, and 100 ng WT or mutant β-catenin or empty vector using Lipofectamine 3000. After 48 hours, cells were lysed (Biotium passive buffer) and assayed with the Firefly/Renilla Single Tube Kit (Biotium). Cell lysates were diluted 1:20 in 1× PBS for firefly luciferase measurement and then 1:20 in 1× PBS again for Renilla luciferase measurement. Measurements were captured using a TD-20/20 Luminometer (Turner BioSystems). *n* = 4–9 technical replicates per condition; data are shown as fold change versus endogenous luciferase, unless otherwise stated.

### Immunohistochemistry.

FFPE tumor sections (4 μm) from *n* = 11 endometrial tumors from our previously published cohort (29), validated to have exon 3 *CTNNB1* mutations, were processed for immunohistochemistry for CD73 and β-catenin as previously described (17, 32). Tumor images (10–30 per sample, ×10 magnification) were captured using an Olympus BX41 microscope. CD73 and β-catenin staining was manually quantified across *n* = 15–30 ×20-magnified images per tumor using cellSens software (Olympus).

### Real-time quantitative PCR.

Quantitative reverse transcription PCR for *CD73* was performed on *n* = 30 EC tissues with validated exon 3 *CTNNB1* mutation by NGS (17). RNA was isolated from frozen tissues using TRIzol Reagent (Invitrogen), followed by purification with RNeasy columns (QIAGEN). Real-time quantitative PCR was performed as previously described (17, 31). Two samples (*n* = 2) were excluded from analyses, one owing to a PCR amplification error and one identified as an extreme outlier based on GraphPad Prism’s outlier detection criteria. The final dataset included *n* = 28 samples.

### Protein isolation and immunoblotting.

Cells were lysed in 1× RIPA buffer with protease/phosphatase inhibitors (Thermo Fisher Scientific) by scraping of frozen plates or after trypsinization. Total protein was quantified (Bio-Rad assay, 595 nm), and equal amounts (10–20 μg) were separated by SDS-PAGE. Total protein was visualized with 2,2,2-trichloroethanol. Proteins were transferred to PVDF or nitrocellulose membranes, blocked in 5% milk/PBST, incubated with primary antibodies overnight (4°C), and detected with HRP-conjugated secondary antibodies and ECL (SuperSignal West Pico, Thermo Fisher Scientific). Western blot densitometry was performed using ImageJ (NIH).

### Cellular fractionation.

*CD73-*WT and *CD73-*KO HEC-1-A cells were plated in 150-cm plates at 6 × 10^6^ cells per plate and then transfected at 85% confluence with *Xenopus* β-catenin^ΔEX3^, G34R, or S37F β-catenin construct (2 μg). At 48 hours after transfection, cells were trypsinized and counted, and 12 × 10^6^ cells were pelleted for cellular fractionation. A second pellet of 2 × 10^6^ cells was collected from the same samples for total cell protein extract. Cellular fractionation was performed using a Pierce Subcellular Protein Fractionation Kit (Thermo Fisher Scientific). Total cell protein extracts were prepared using 1× RIPA buffer.

### Digital spatial profiling.

GeoMx data used in this study originated from a previously published study with publicly available datasets (113), which we reanalyzed for the purposes of this work. The dataset includes nuclear and non-nuclear ROIs from *n* = 16 NGS-validated exon 3 *CTNNB1*-mutant endometrial tumors (113). Six ROIs (200–400 μm, >100 nuclei each) were selected per tumor (3 nuclear, 3 non-nuclear), totaling 96 ROIs, and whole-transcriptome profiling, targeting 18,677, genes was performed. Data were processed in the GeoMx digital spatial profiling (DSP) portal, including quality control and Q3 normalization. Quality control excluded 2 ROIs because of low read counts and filtered segments and probes with low negative control signals. Q3 normalization adjusted for variability by scaling to the 75th percentile (113).

### TCGA Uterine Corpus Endometrial Carcinoma analyses.

Gene expression data were analyzed using RStudio (2023.06.2 build 561), R (4.2.2), ComplexHeatmap (2.14.0), colorRamp2 (0.1.0), dplyr (1.1.4), ggplot2 (3.5.1), and ggpubr (0.6.1). Briefly, *z* score–transformed STAR gene counts were used to compare WT (*n* = 158) and mutant β-catenin (*n* = 60) as well as *NT5E* expression (upper quartile versus the rest) in early-stage (FIGO I and II) tumors. Samples with no available *NT5E* expression were excluded. Exploratory data analysis was performed with heatmaps, and box-and-whisker plots were generated.

### Invasion assay.

Boyden Chamber inserts (6.5 mm, 8 μm; Corning 3422) were coated with Matrigel/serum-free MEM (200 μL Matrigel in 3 mL MEM) for 20–30 minutes, washed, and seeded with 60,000 cells in 200 μL serum-free MEM. Lower chambers contained 500 μL MEM plus 5% FBS. After 36 hours, non-invaded cells were removed, and invaded cells were fixed with methanol, stained with hematoxylin, and imaged (20–22 images per membrane, ×20; Keyence BZ-X810). Cells were quantified using ImageJ Cell Counter.

### Spheroid formation assay.

Cells (5,000 per well, 3 replicates) were seeded as single cells in ultra-low-attachment 6-well plates and cultured in DMEM/F-12 with 20 ng/mL EGF (Gibco, PHG0314), 20 ng/mL bFGF (Gibco, 13256029), 2% B27 (Gibco, 17504044), and 1% penicillin/streptomycin. Spheroids were imaged at day 2 using an ×4 phase-contrast lens (Olympus CKX41), 10–16 images per well; and diameters were measured with ImageJ.

Details regarding TIDE (tracking of indels by decomposition), immunofluorescence, coimmunoprecipitation, RNA extraction for RNA sequencing, and RNA-Seq analyses are provided in Supplemental Methods.

### Statistics.

*P* values were calculated as indicated in figure legends using GraphPad Prism 10. A *P* value of less than 0.05 was considered significant. STRING v12.0 and R2 Genomic Analysis and Visualization Platform (https://hgserver1.amc.nl/cgi-bin/r2/main.cgi?open_page=login) were used where indicated.

### Study approval.

Use of human tissues was approved (LAB01-718) by the Institutional Review Board of the University of Texas MD Anderson Cancer Center.

### Data availability.

RNA-Seq data from HEC-1-A cells are available on the NCBI Biosample database, BioProject accession PRJNA1251118. Values for all data points in graphs are reported in the Supporting Data Values file.

## Author contributions

RMH and JLB designed and performed experiments and analyzed data. LFC and CV developed the CD73 adenoviral vector. SP, HNL, and EMR performed experiments and analyzed data. GND conducted TCGA analyses, and GND, CV, and JLB interpreted the results. KCK and RRB provided patient samples and clinical data. MLP and RRB generated GeoMx DSP data. RMH and JLB wrote the original draft of the manuscript. JLB wrote the revised draft. All authors read, revised, and approved the manuscript. RRB, CV, and JLB supervised the study.

## Funding support

This work is the result of NIH funding, in whole or in part, and is subject to the NIH Public Access Policy. Through acceptance of this federal funding, the NIH has been given a right to make the work publicly available in PubMed Central.

- NIH P50CA098258 (to RRB).
- Uterine SPORE Career Enhancement Award from NIH P50CA098258 (to JLB).
- UNC Environmental Health and Susceptibility Pilot Award P30ES010126 (to JLB).
- International Anesthesia Research Society Mentored Research Award (to JLB).
- NIH training grants T32GM122741 (to RMH and EMR) and 5T32GM135128 (to LFC).

## Supplementary Material

Supplemental data

Unedited blot and gel images

Supporting data values

## Version 1 01/23/2026

Electronic publication
