# Supplementary material for: CD73 restrains mutant β-catenin oncogenic activity in endometrial carcinomas
Source: JCI Insight. 2026 Jan 23;11(2):e189510. doi: 10.1172/jci.insight.189510 (PMC12892904; doi:10.1172/jci.insight.189510)

Supplemental Methods

Tracking of indels by decomposition (TIDE). gDNA extraction was performed with QIAGEN columns. Target-specific PCR products were generated and sequenced by Azenta for analysis of CRISPR-Cas9 editing in CD73-KO, A1R-KO, and A2BR-KO cell lines. The TIDE webtool (available for free courtesy of Eva Brinkman, Tao Chen and Bas van Steensel) was used to calculate the frequency and spectrum of genome alterations introduced by CRISPR-Cas9 editing in *NT5E*, *ADORA1*, and *ADORA2B* genes (1).

Immunofluorescence. Cells grown on 18x18 mm coverslips were fixed (4% paraformaldehyde), permeabilized (0.1% Triton X-100), and blocked (Background Snipper, Biocare Medical). Primary antibodies were incubated overnight at 4°C, followed by secondary antibodies. Bleed-through controls included single antibody/fluorochrome incubations. For nuclear localization of myc-tagged *Xenopus* β-catenin$^{\Delta EX3}$, ~90 images/group (20X) were captured and fluorescence intensity determined with BZ-X800 Analyzer Macro Cell Counting software (Keyence).

Co-immunoprecipitation (Co-IP). HEC-1-A CD73-WT and -KO cells were transfected with *Xenopus* β-catenin$^{\Delta EX3}$, G34R mutated β-catenin vector, or S37F mutated β-catenin vector (2 ug). Co-IP was performed using a Pierce myc-tag Magnetic IP/Co-IP Kit (Thermo Scientific). Protein (500 µg) was incubated overnight at 4°C with 25 µl anti-myc magnetic beads. The resulting immune-bound complexes were eluted in 2X reducing sample buffer and assessed by SDS-PAGE and immunoblotting methods.

RNA extraction for RNA-sequencing. HEC-1-A CD73 WT or KO cells were plated (5 × 10^5/well, 6-well plates), transfected at ~85% confluency with empty vector (LentiV_Neo) or D32N, G34R, or S37F β-catenin mutant constructs (2 μg), and harvested 48 hours later. Cells were washed with 1X PBS, frozen at -80 °C, and RNA isolated using QIAshredder and RNeasy Mini Kits (QIAGEN). RNA concentration was measured by Nanodrop, and integrity confirmed by agarose gel electrophoresis. Secondary QC and RNA sequencing were performed by Novogene.

RNA-seq analyses. Stranded Bulk mRNA sequencing was performed on Poly-T selected total RNA isolates following size distribution detection (Novogene). Transcriptomic sequences were gathered at 150bp paired-end reads at or exceeding 35 million reads per replicate. Several analyses were performed on the sequences: mapping by hisat2 (2.05), assembly by Stringtie (1.3.3b), quantification by featureCount (1.5.0-p3), and DE analysis by DESeq2 (1.20.0). R2 Genomics Analysis and Visualization Platform (http://r2.amc.nl) (2) was used in Figure 8D.

Supplemental References

1. Brinkman EK, Chen T, Amendola M, and van Steensel B. Easy quantitative assessment of genome editing by sequence trace decomposition. *Nucleic Acids Research.* 2014;42(22):e168.
2. Koster J, Volckmann R, Zwijnenburg D, Molenaar P, and Versteeg R. Abstract 2490: R2: Genomics analysis and visualization platform. *Cancer Research.* 2019;79(13_Supplement):2490.

Supplemental Table 1

Clinicopathological features of qRT-PCR cohort of exon 3 *CTNNB1*-mutant endometrial carcinomas.

**Characteristics (n = 28)**	**No Recurrence**	**Recurrence**
Histology	**n =**	**n =**
Endometrioid	17	10
Non-endometrioid	1	0
FIGO Stage	**n =**	**n =**
I	13	8
II	1	0
III	3	1
IV	0	1
Unknown	1	
Grade	**n =**	**n =**
G1	2	2
G2	15	7
G3	1	1
Lymphovascular Space Invasion (LVSI)	**n =**	**n =**
Yes	4	4
No	14	4
Unknown		2

Supplemental Table 2

Antibodies

Antibody	Source	Company	Catalog #/Clone
Alexa Fluor 594 goat anti-mouse IgG	Goat polyclonal	Invitrogen	#A21206
Alexa Fluor 594 goat anti-mouse IgG	Goat polyclonal	Invitrogen	#A11032
Goat anti-mouse biotinylated IgG	Goat polyclonal	Vector Laboratories	#BP-9200-50
β-catenin (WB)	Rabbit polyclonal	Cell Signaling	#8480/D10AB
β-catenin (IHC)	Mouse monoclonal	BD Biosciences	#310154/Clone 14
CD73	Rabbit monoclonal	Cell Signaling	#13160/D7F9A
CD73	Mouse monoclonal	HycultBiotech	#HM2215/4G4
E-cadherin	Mouse monoclonal	BD Biosciences	#610181/ #36/E-Cadherin
GAPDH	Rabbit polyclonal	Cell Signaling	# 3683/14C10
HRP-conjugated anti-mouse IgG	Horse	Cell Signaling	#7076
HRP-conjugated anti-rabbit IgG	Goat	Cell Signaling	#7074
Myc-tag (WB)	Rabbit polyclonal	Cell Signaling	#2278/71D10
Myc-tag (IF)	Mouse monoclonal	Cell Signaling	#2276/9B11
SP 1	Rabbit polyclonal	Cell Signaling	#9389/D4C3
Rab11a	Rabbit polyclonal	ABClonal	#A3251/ARC0767
H2AX	Rabbit polyclonal	Cell Signaling	#7631/D17A3
α-catenin	Rabbit monoclonal	Cell Signaling	#3240/23B2
GAPDH-HRP	Rabbit monoclonal	Cell Signaling	#3683S/14c10

Supplemental Table 3

Genes in heatmaps of Figure 7A and 7B.

Figure 7A		Figure 7B	
Position	Gene (Top to Bottom)	Position	Gene (Top to Bottom)
1	MNS1	1	NT5E
2	NT5E	2	THSD7A
3	LGR5	3	CCDC87
4	CCL28	4	KLLN
5	RHOU	5	MYLK3
6	SOX17	6	DNAH2
7	LEF1	7	SMOC1
8	JUN	8	MYOM2
9	SLC7A2	9	MSX2
10	GLS2	10	CYP4X1
11	LOXL3	11	ENTPD3
12	SLC16A10	12	SOX5
13	MYC	13	ANKRA2
14	ABCC4	14	ITGA10
15	GRAMD1A	15	MSX1
16	AXIN1	16	c19orf73
17	CDCA4	17	BICDL2
18	POLR1G	18	ELMO3
19	PPARD	19	ANKRD24
20	EN2	20	IFI44L
21	ALDH1A1	21	DDX60
22	MMP7	22	NKX2.3
23	SCARA3	23	HOXA9
24	JAC1	24	UNC5C
25	CLDN1	25	ZFAND2A
26	ID2	26	H2BC15
27	LRRN1	27	HEBC11
28	FN1	28	H2AC6
29	CCN4	29	ZNF229
30	SNAI1	30	THRB
31	FZDF	31	ASCL4
32	NEUROD1	32	IGSF21
33	PITX2	33	CCDC85A
34	GBX2	34	PAX2
35	CD274	35	INSC
36	CTLA4	36	MAFB

37	NOS2	37	P2RX5
38	CSF3R	38	FERMT3
39	ADAMTS14	39	SLC9A9
40	PALD1	40	JAK3
41	FGF9	41	BCL11B
42	HDAC4	42	TEX19
43	TCF7	43	PYGM
44	PROX1	44	BMPER
45	DKK1	45	CALCB
46	IL10	46	TLL1
47	FGF20	47	C1QL1
48	BAMBI	48	PRPH
49	AXIN2	49	FIBIN
50	NOTUM	50	EYA1
51	NKD1	51	ADAMTS5
52	BMP4	52	ANKLE1
53	SP5	53	MSTN
54	RNF43	54	PLAT
55	ZNRF3	55	MYH11
56	CCND1	56	ADGRA2
57	STRA6	57	PIK3IP1
58	GJA1	58	SLC7A8
59	SOX9	59	ST8SIA2
60	EPHB3	60	AXIN2
61	LBH	61	NKD1
62	TIAM1	62	SP5
63	MSI1	63	GPR83
64	FAM216A	64	TCF7
65	TFAP4	65	CCBE1
66	TEAD4	66	ETV1
67	ANKD13B	67	DDK1
68	FOSL1	68	SYTL2
69	CBX2	69	SCUBE1
70	NES	70	RRH
71	GINS3	71	LRIT3
72	PDK1	72	CCNG2
73	ZNF724	73	CREBRF
74	FAM111B	74	RASGEF1B
75	DTL	75	RASSF6
76	ZNF367	76	RNF19B
77	FOXRED2	77	CLDN1
78	UHRF1	78	B3GNT3
79	MCM2	79	CCND1

80	GINS2	80	RGS4
81	CDT1	81	COL7A1
82	RFC4	82	NMNAT3
83	VEGFA	83	PIP5KL1
84	PLAUR	84	SNAI3
		85	ADCY5
		86	NEDD9
		87	HBP1
		88	PLXNA2
		89	CACNA1D
		90	DLL1
		91	UQCRHL
		92	BAG1
		93	RPL41
		94	NTMT1
		95	ZNF503
		96	B3GNT7
		97	HRH1
		98	CBLN3
		99	TSHZ1

Supplemental Figure 1. CD73 expression stratified by FIGO stage, LVSI, and non-nuclear and nuclear β-catenin ROIs in *CTNNB1*-mutant endometrial tumors. Whole tumor mRNA for CD73 stratified **(A)** FIGO stage *(n* = 28*)* and **(B)** LVSI *(n* = 27*)*. Box plots show median, IQR, mean (cross), whiskers (±1.5×IQR), and outliers (circles). Values are molecules of CD73 transcripts/molecules of 18S rRNA**. (C)** DSP data for CD73 mRNA expression in *n = 16* NGS-confirmed exon 3 *CTNNB1*-mutant endometrial tumors. Three nuclear and non-nuclear ROIs were sampled for each tumor (unless otherwise indicated, see individual circles for each tumor). Data represent the mean ± SEM. *P < 0.05, **P < 0.005, ***P < 0.0005, ***P < 0.0001; two-way ANOVA with Sidak's post test

Supplemental Figure 1

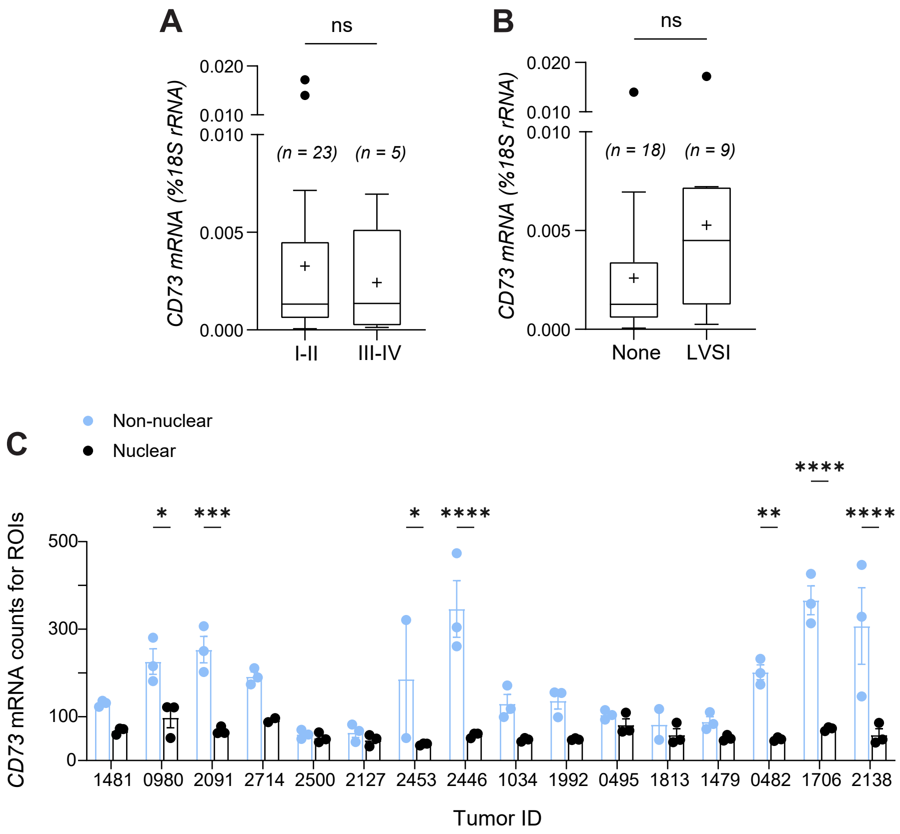

Supplemental Figure 2. Induced expression of CD73 via *NT5E* adenovirus transduction in Ishikawa cells. (A) CD73 protein expression with different *NT5E* AdV viral titers compared with HEC-1-A cells which serve as positive controls. CD73 Positive Control 1 = HEC-1-A cells at 100% confluency, 2 = HEC-1-A cells at 2 days post-confluency. **(B)** Validation of continued CD73 expression in Ishikawa cells. Expression 91 persists for 96 hours, the endpoint in which TCF/LEF luciferase assays were performed. HEC-1-A cells serve as CD73 positive controls. **(C)** Uncropped immunofluorescence images corresponding to Figure 2H. Cropped areas indicated with white rectangle. Scale bars 20 um.

Supplemental Figure 2

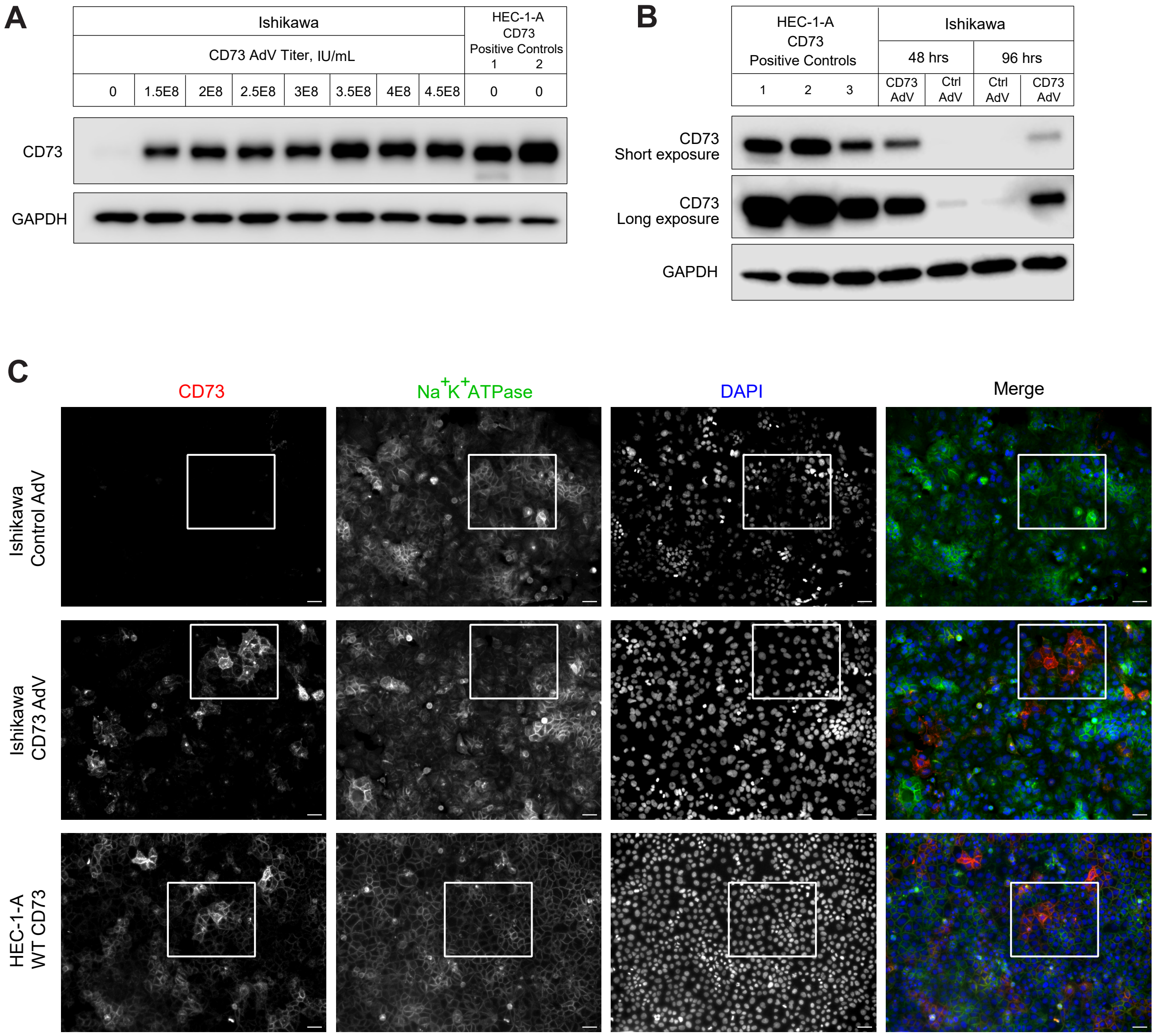

Supplemental Figure 3. CD73 restrains transcriptional activity of β-catenin mutant G34R. (A-D) Validation of TCF/LEF reporter activity for patient-specific β-catenin mutant G34R in Ishikawa cells. Additional independent experiments were conducted with G34R due to variability observed with the mutant as seen in Figure 3E. Reporter assays were performed in Ishikawa cells with no empty vector transfection **(**endogenous, **A)** and transfection of an empty vector **(B-C)** in addition to *NT5E* (CD73) AdV DNA constructs. **(D)** Combination of data from **(B)** and **(C)**. Three independent experiments are shown for β-catenin mutant G34R, which are **(A)**, **(B)**, and **(C)**. Data represent the mean ± SEM. ***P < 0.005, ****P < 0.0001; two-way ANOVA with uncorrected Fisher's LSD.

Supplemental Figure 3

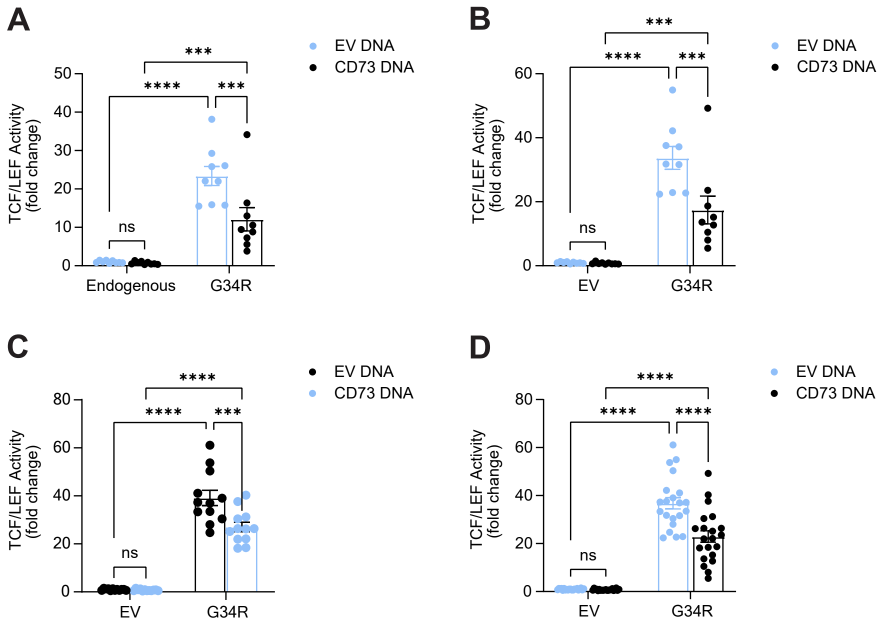

Supplemental Figure 4. Independent replicates of cellular fractionations with patient-specific β-catenin mutations. (A-E) Independent replicates of cellular fractionation experiments described in Figure 4 from CD73-WT and -KO HEC-1-A cells. CD73-WT and -KO HEC-1-A cells were transfected with **(A)** Xenopus β-catenin$^{\Delta EX3}$ or patient-specific β-catenin mutants **(B)** S37F or **(C-E)** G34R. β-catenin mutant G34R showed the most variability between independent replicates. Densitometry graphs are shown for myc-β-catenin mutant expression for each cellular fraction normalized to myc-β-catenin mutant expression in the whole cell lysate (WCL) in addition to total protein. Cellular fraction markers: Rab11a (membrane), SP1 (nuclear), and H2AX (chromatin).

Supplemental Figure 4

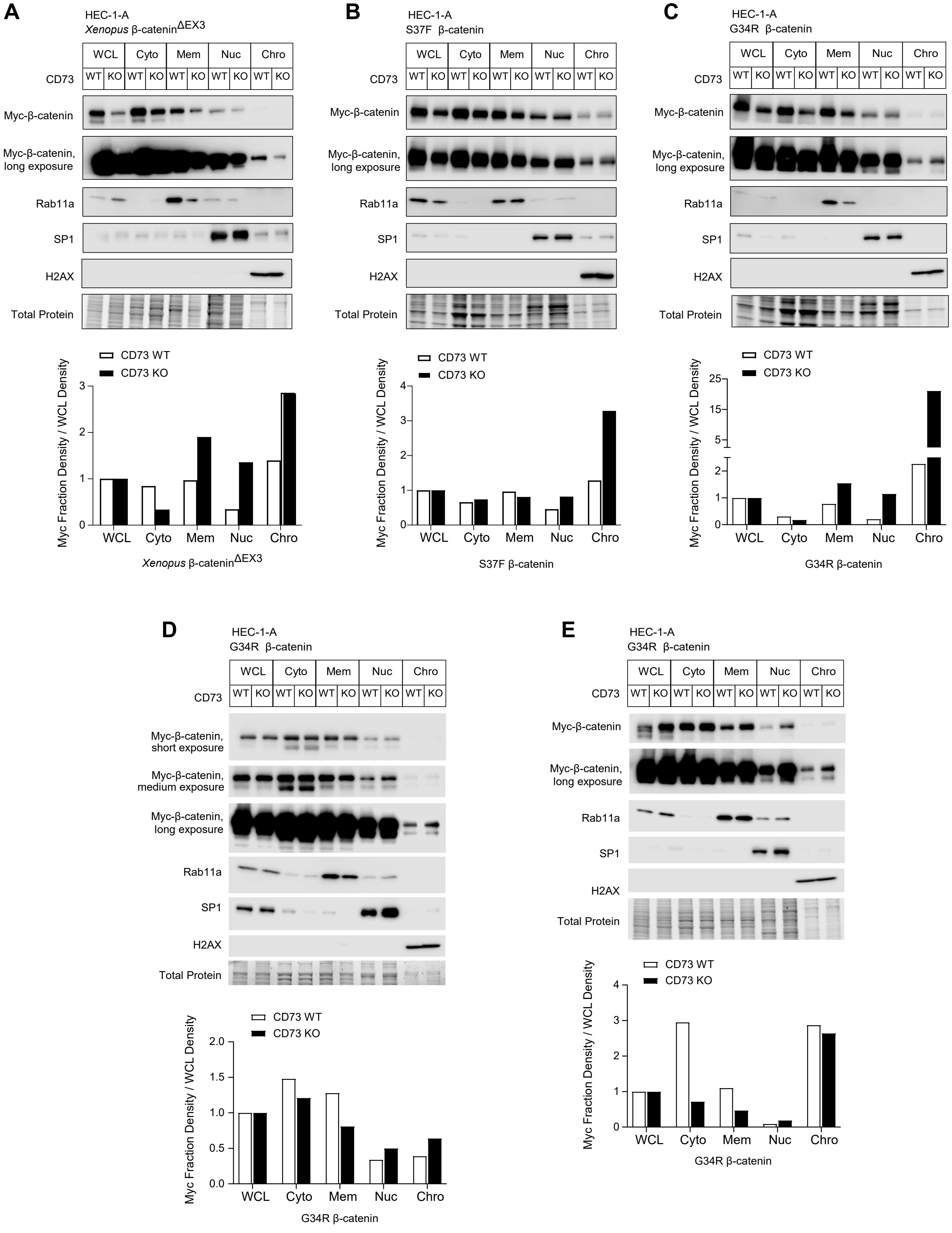

Supplemental Figure 5. Patient-specific exon 3 β-catenin mutant binds with E-cadherin. Immunoblots from co-immunoprecipitation experiment in CD73-WT and -KO HEC-1-A cells. Myc-β-catenin was precipitated and samples were probed for E-cadherin and myc-tagged expression of β-catenin mutant G34R. Downregulation of E-cadherin **(B)** and other cell-cell adhesion and barrier function genes **(B-C)** occurs in CD73-KO cells compared with CD73-WT cells. **(C)** RNA-seq data from CD73-KO and -WT cells. Data represent the mean ± SEM. **$P < 0.001$, ***$P < 0.0005$, ****$P < 0.0001$; multiple unpaired t test (2-tailed).

Supplemental Figure 5

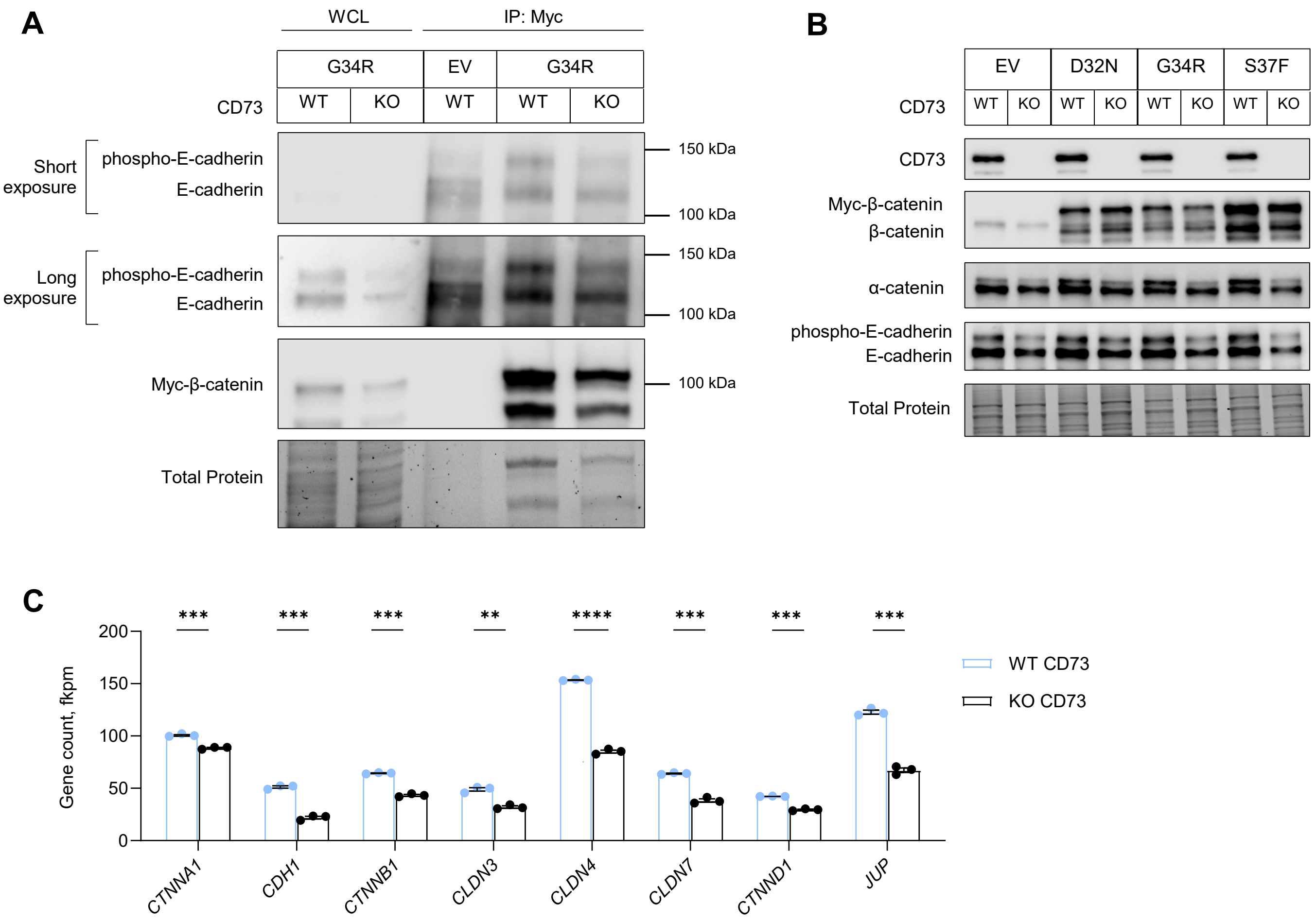

Supplemental Figure 6. Reduced transcriptional activity of patient-specific β-catenin mutants in A1R KO cells. (A) Immunoblot showing E-cadherin expression is unchanged in *ADORA1* (A1R)-KO and *ADORA2B* (A2BR)-KO cells compared with WT HEC-1-A cells. **(B-C)** Independent replicate experiments for data shown in Figure 5E. TCF/LEF reporter activity in cells transfected with empty vector (EV) or patient-specific β-catenin mutants D32N, G34R, or S37F. Each dot represents one technical replicate. Data represent mean ± SEM. *$P < 0.05$, ****$P < 0.0001$; two-way ANOVA with Dunnett's post-test.

Supplemental Figure 6

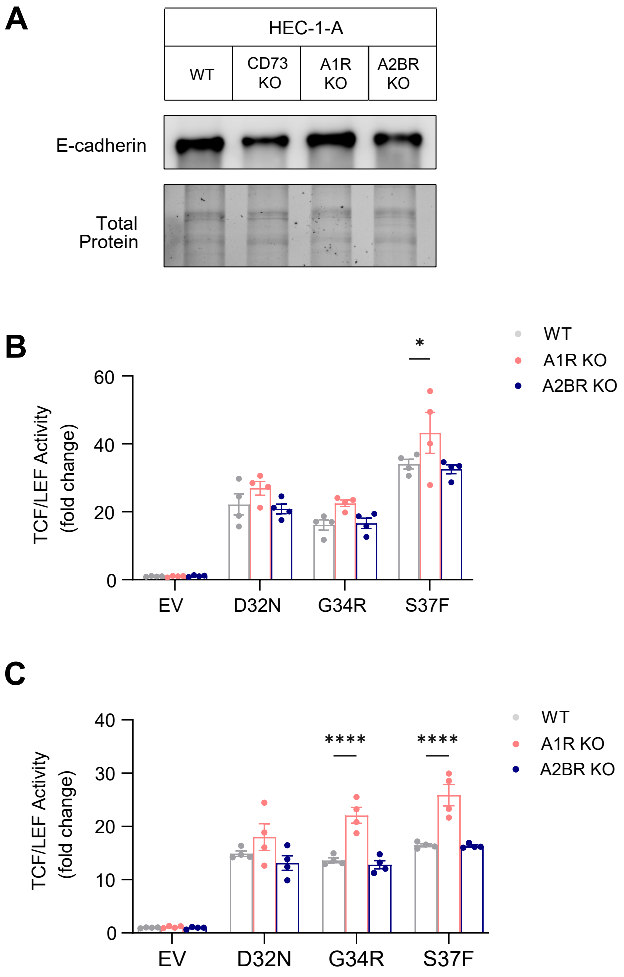

Supplemental Figure 7. CD73 restrains invasiveness and stemness capacity of S37F and G34R β-catenin mutants. (A-B) Independent replicate experiments for data shown in Figure 6B-6C. **(A)** Invasion and **(B)** spheroid-forming assays in Ishikawa cells with stable ectopic expression of CD73 (or empty vector) and either S37F or G34R β-catenin mutants. Data represent mean ± SEM. ****$P < 0.0001$; Mann-Whitney t-test (2-tailed).

Supplemental Figure 7

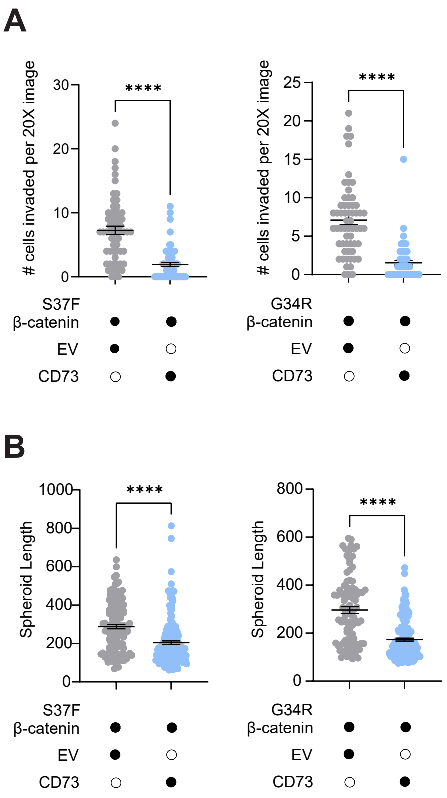

Supplemental Figure 8. CD73, Wnt signaling, and β-catenin-dependent, TCF/LEF-dependent gene expression in Uterine Cancer TCGA dataset. (A) CD73 levels in normal tissue and tumors. **(B)** CD73 mRNA expression in *CTNNB1*-mutant vs. -WT and CD73 High vs. Low early-stage EC. Unmarked heatmaps shown in Figure 7A and 7C of gene expression of gene lists: Wnt signaling **(C)** and β-catenin-dependent **(D)**. **(C-D)** Tumors are stratified by CD73 expression levels and without stratifying by *CTNNB1* genotype (mutant vs. WT). **(A)** 2-tailed t-test. **(B)** Data represent mean ± SEM. Kruskal-Wallis test with Benjamini-Hochberg FDR, post-hoc Dunn test. ***P < 0.001.

Supplemental Figure 8

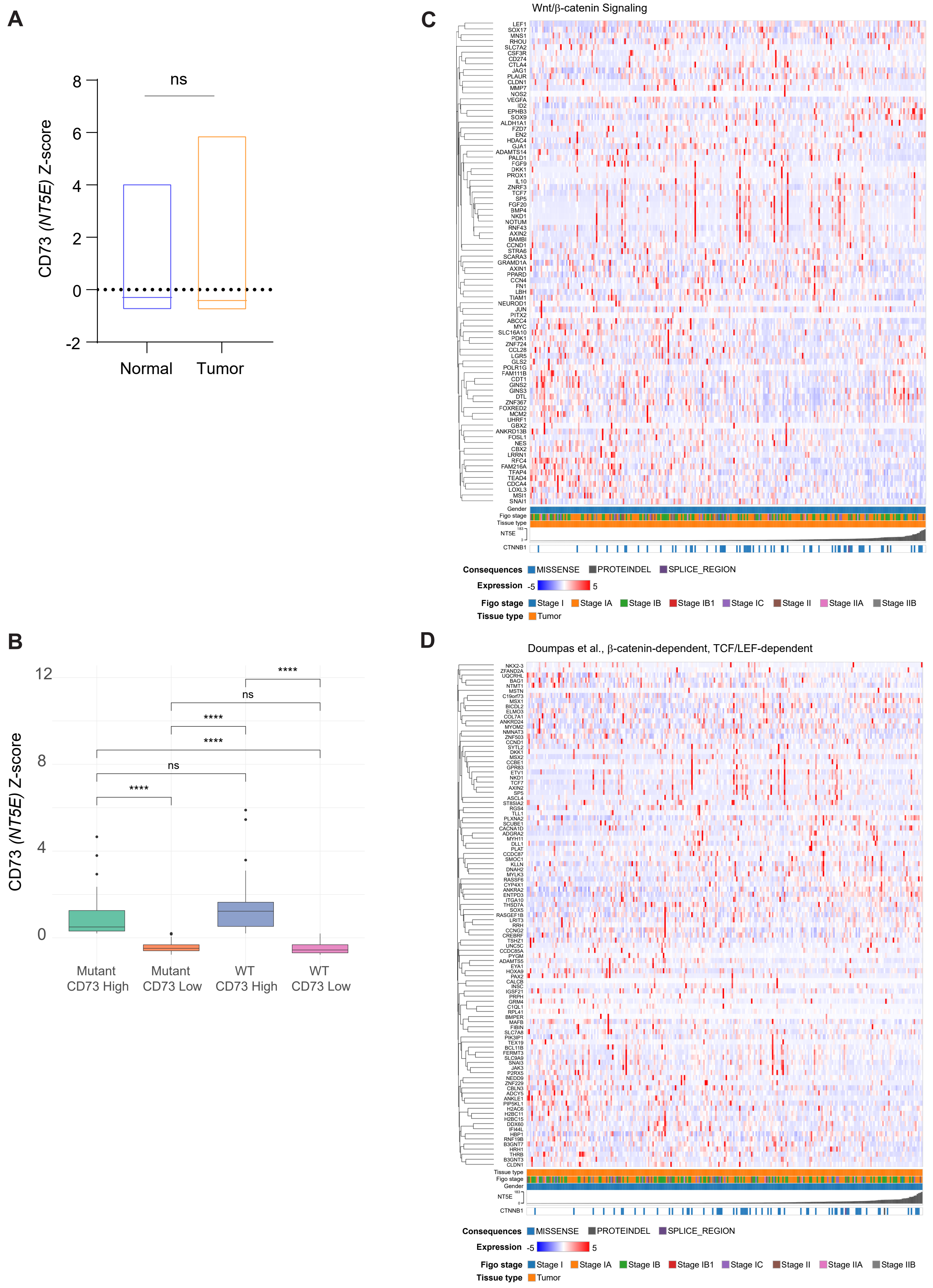

Supplemental Figure 9. Validation of patient-specific β-catenin mutant expression and activity in RNA-seq samples. (A) Protein samples were collected in sync with samples process and submitted for RNA-sequencing. Immunoblots were used to assess equal or near equal expression of each patient-specific β-catenin mutant between CD73-WT and -KO HEC-1-A cells. Due to unequal protein expression of S33F between CD73 WT and CD73 KO samples, RNA from these samples was not submitted for sequencing. **(B)** Densitometry for myc-tagged β-catenin mutants from samples in **(A)**. **(C)** Mutation frequencies for β-catenin mutants D32N, G34R, and S37F in RNA sequences, calculated using Integrative Genomics Viewer. **(D-E)** Validation of our experimental system. **(D)** CD73 and β-catenin expression. **(E)** β-catenin mutants induce Wnt signaling gene targets TCF7 and AXIN2.

Supplemental Figure 9

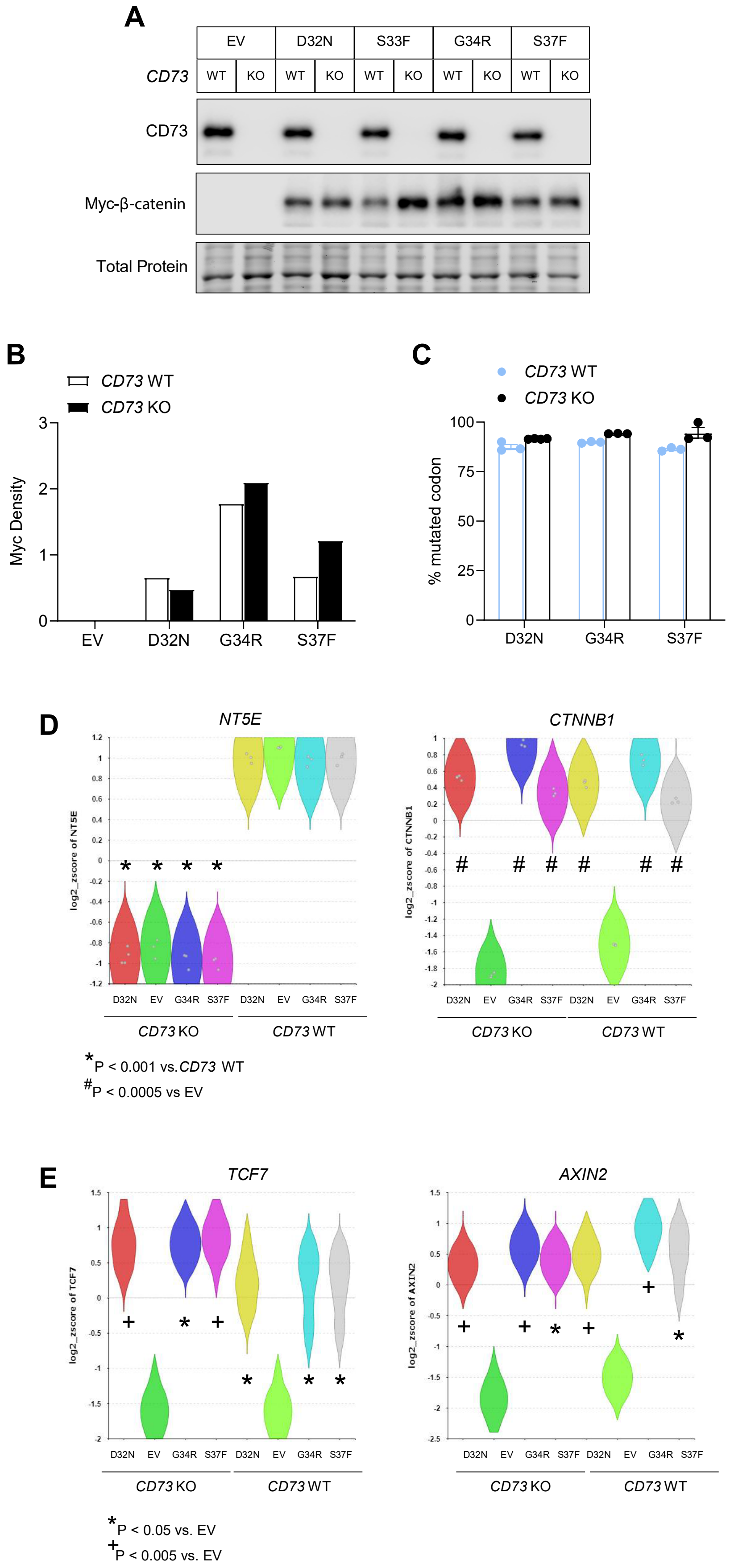

Supplement: Supplemental data [file jciinsight-11-189510-s047.pdf]
