# Supplementary material for: CD73 restrains mutant β-catenin oncogenic activity in endometrial carcinomas
Source: JCI Insight. 2026 Jan 23;11(2):e189510. doi: 10.1172/jci.insight.189510 (PMC12892904; doi:10.1172/jci.insight.189510)
Supplement: Unedited blot and gel images [file jciinsight-11-189510-s048.pdf]

Figure 2

2B

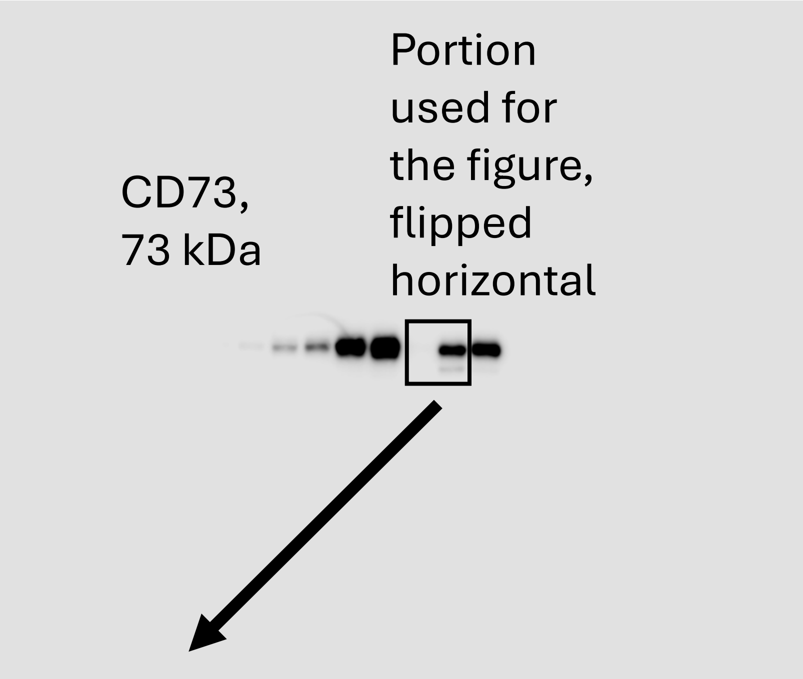

9	8
HEC-1-A 100% confluent	Ishikawa 2D post confluency

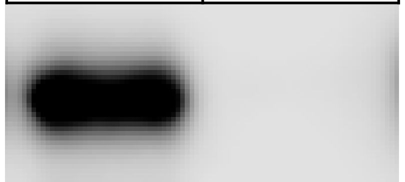

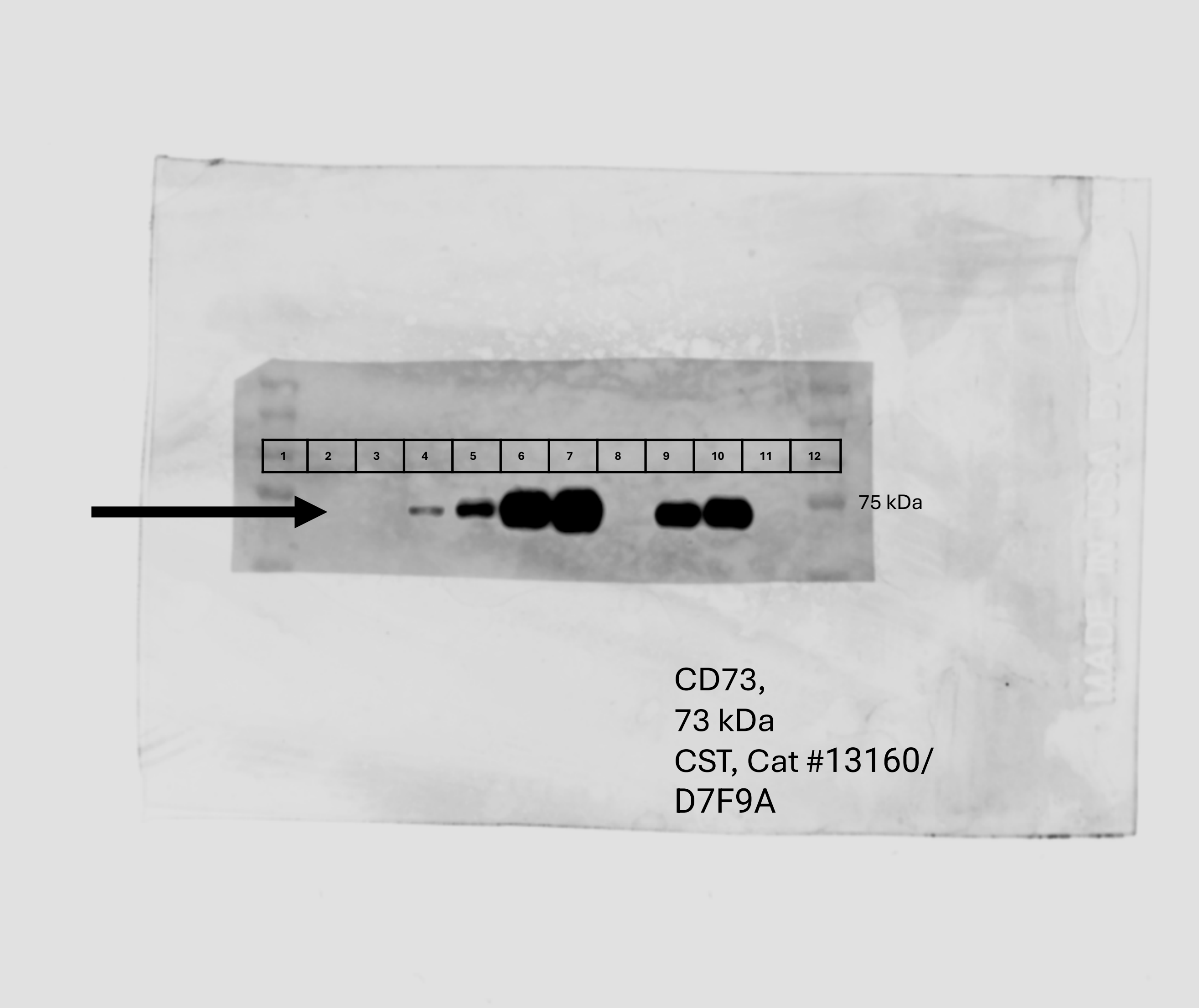

1	2	3	4	5	6	7	8	9	10	11	12
Ladder	No transductio n	NT5E AdV, 1E7 IU/ml	NT5E AdV, 5E7 IU/ml	NT5E AdV, 1E8 IU/ml	NT5E AdV, 5E8 IU/ml	NT5E AdV, 1E9 IU/ml	Ishikawa 2D post confluency	HEC-1-A 100% confluent	HEC-1-A 2D post confluency		Ladder

2B

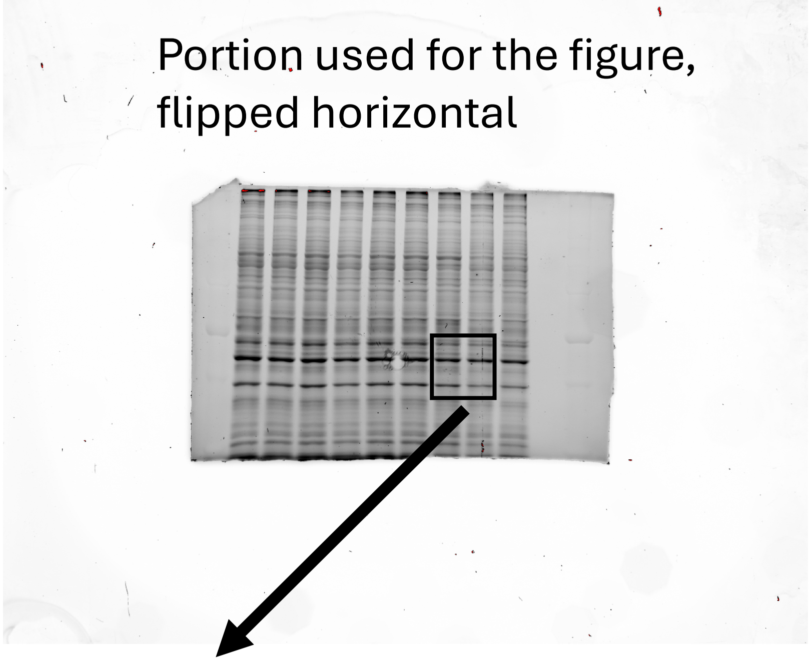

9	8
HEC-1-A 100% confluent	Ishikawa 2D post confluency

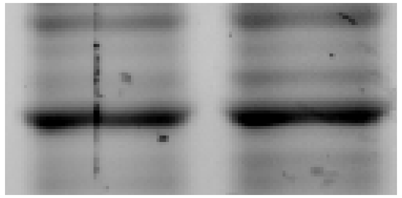

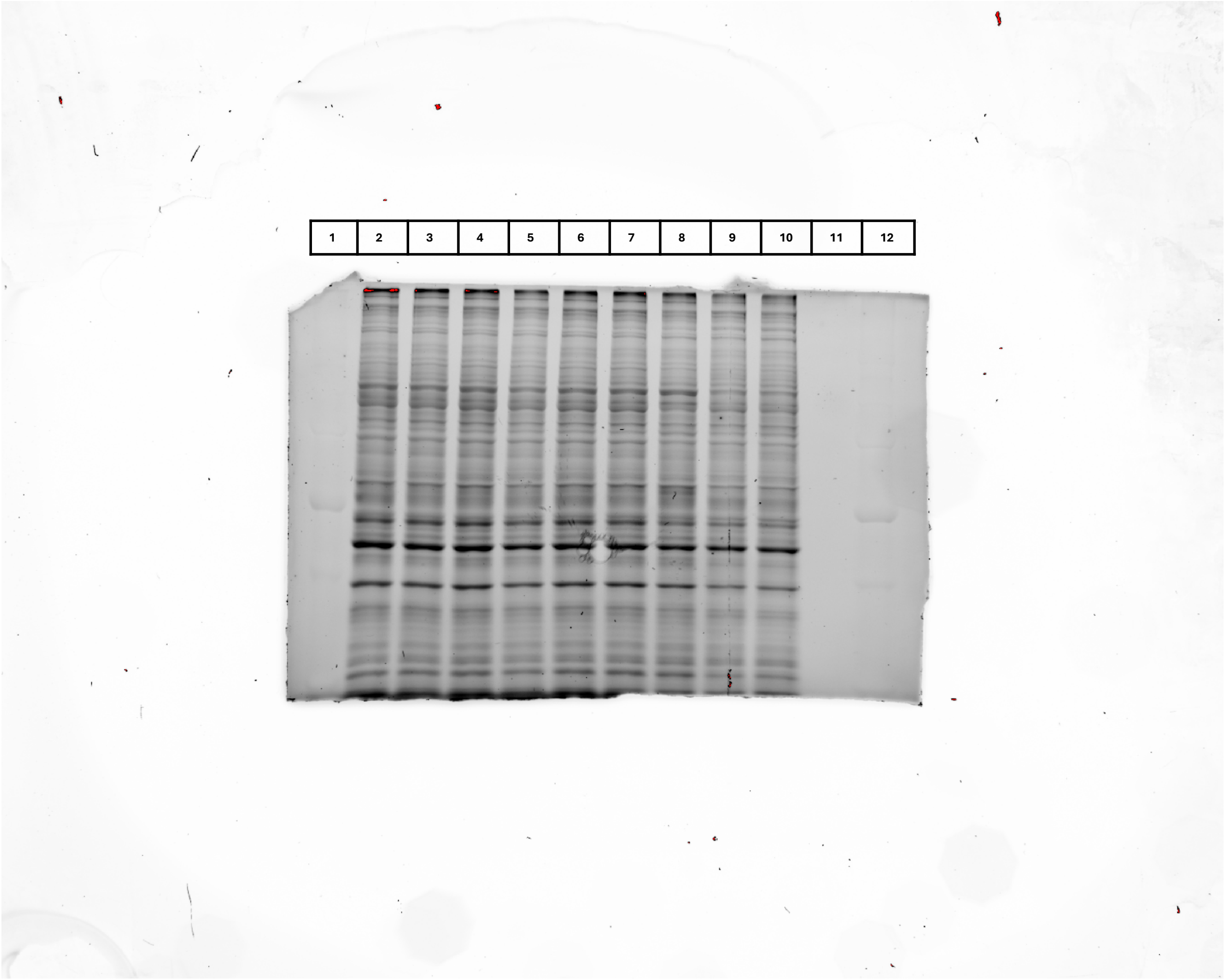

1	2	3	4	5	6	7	8	9	10	11	12
Ladder	No transductio n	NT5E AdV, 1E7 IU/ml	NT5E AdV, 5E7 IU/ml	NT5E AdV, 1E8 IU/ml	NT5E AdV, 5E8 IU/ml	NT5E AdV, 1E9 IU/ml	Ishikawa 2D post confluency	HEC-1-A 100% confluent	HEC-1-A 2D post confluency		Ladder

2E

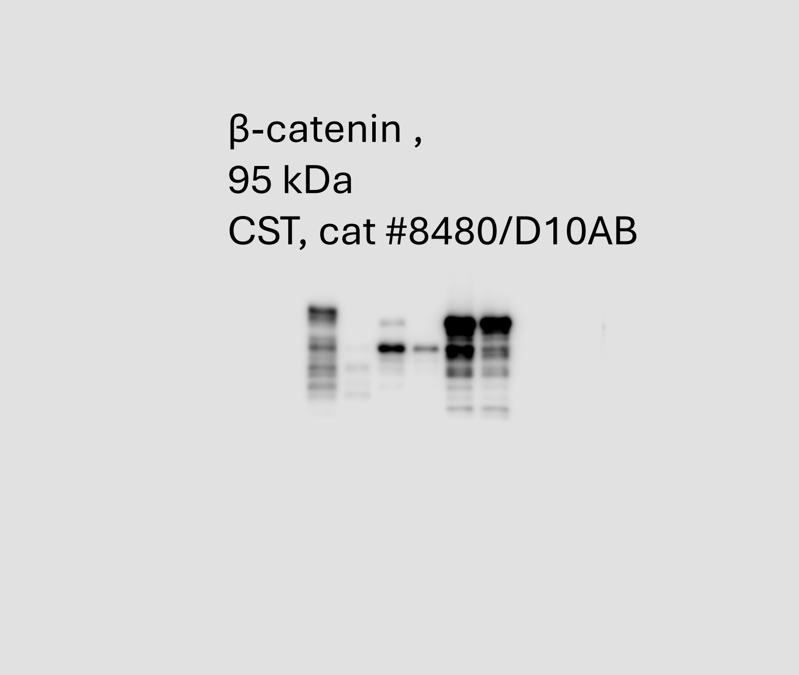

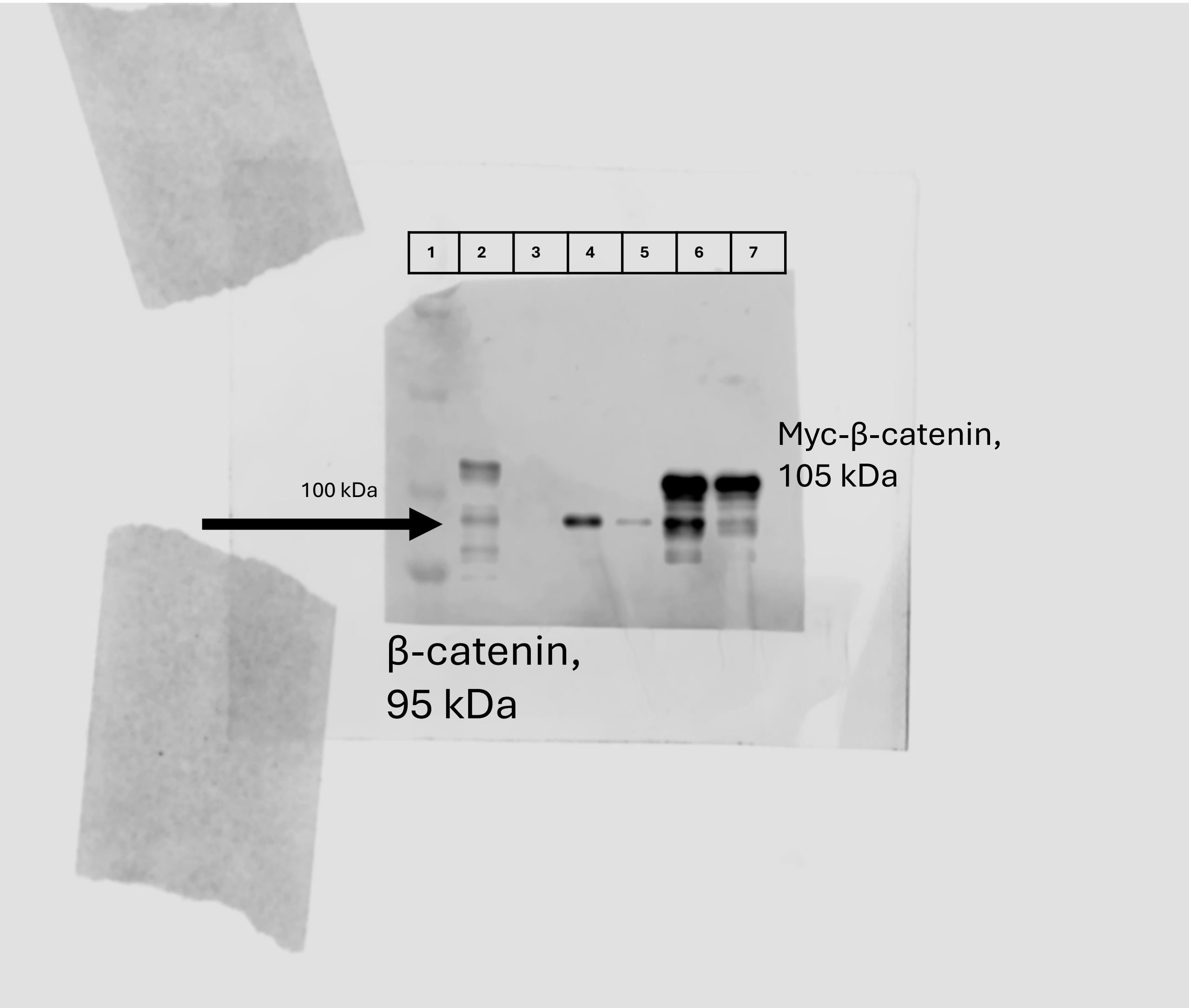

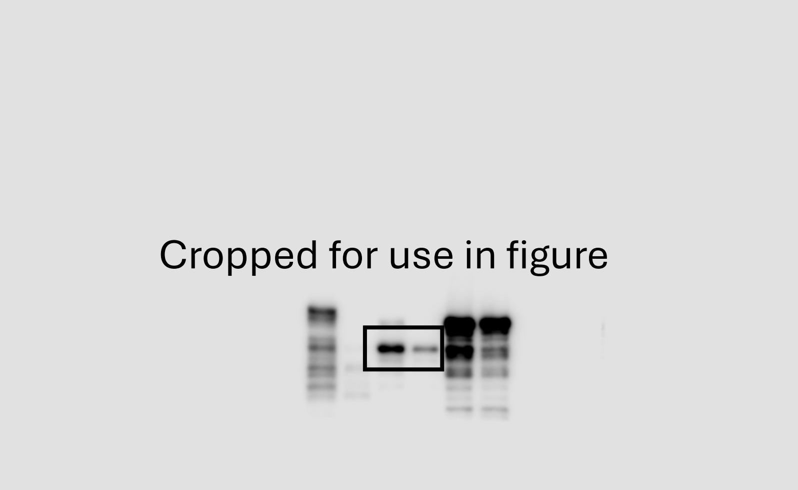

1	2	3	4	5	6	7
Ladder	Xenopus myc-β-catenin	HEC-1-A<br>+ CTNNB1 siRNA, old exp control	HEC-1-A	HEC-1-A<br>+CTNNB1 siRNA	HEC-1-A<br>+ WT myc-β-catenin	HEC-1-A<br>+ WT myc-β-catenin<br>+ CTNNB1 siRNA

2E

Total protein

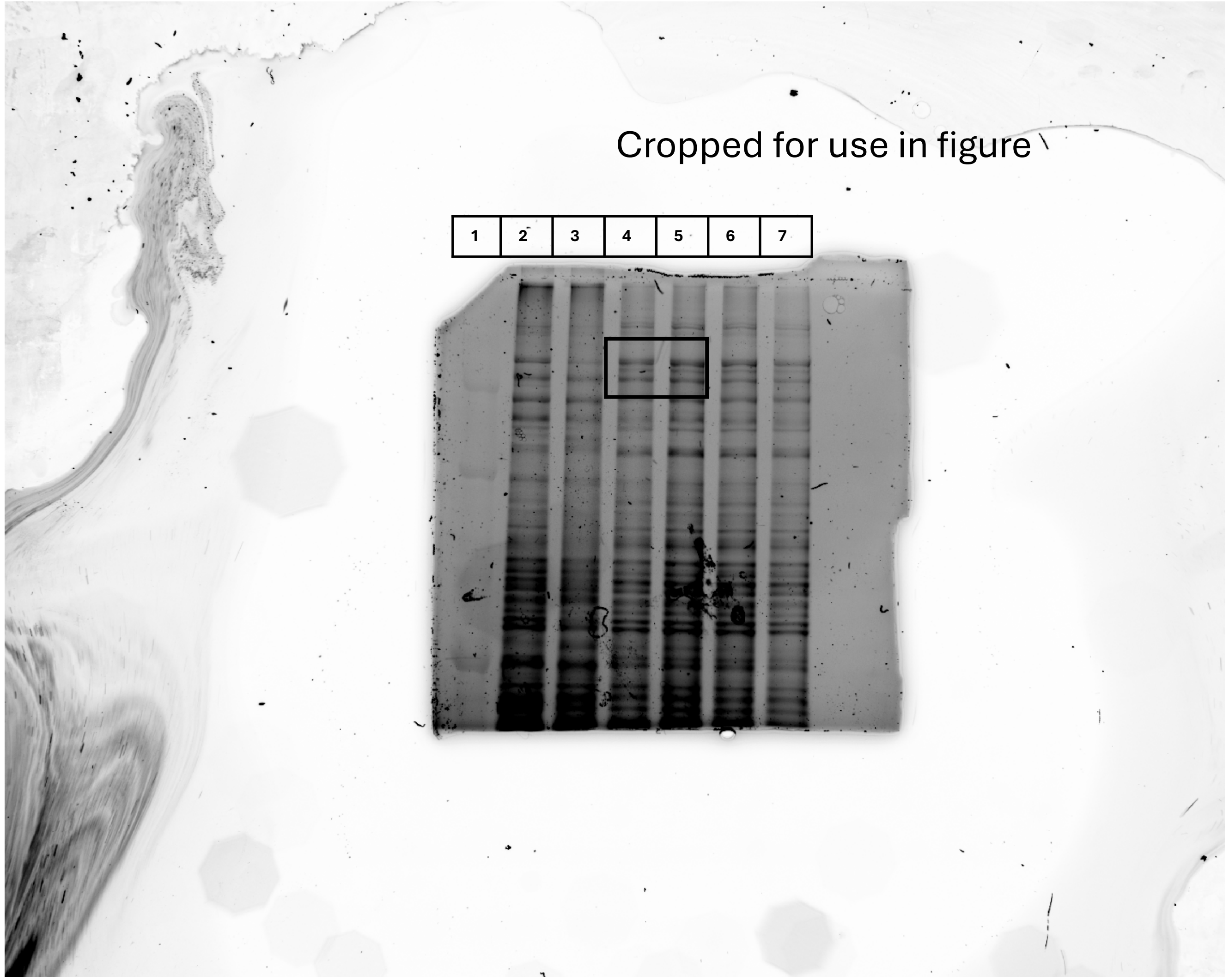

1	2	3	4	5	6	7
Ladder	Xenopus myc-β-catenin	HEC-1-A<br>+ CTNNB1 siRNA, old exp control	HEC-1-A	HEC-1-A<br>+CTNNB1 siRNA	HEC-1-A<br>+ WT myc-β-catenin	HEC-1-A<br>+ WT myc-β-catenin<br>+ CTNNB1 siRNA

2G

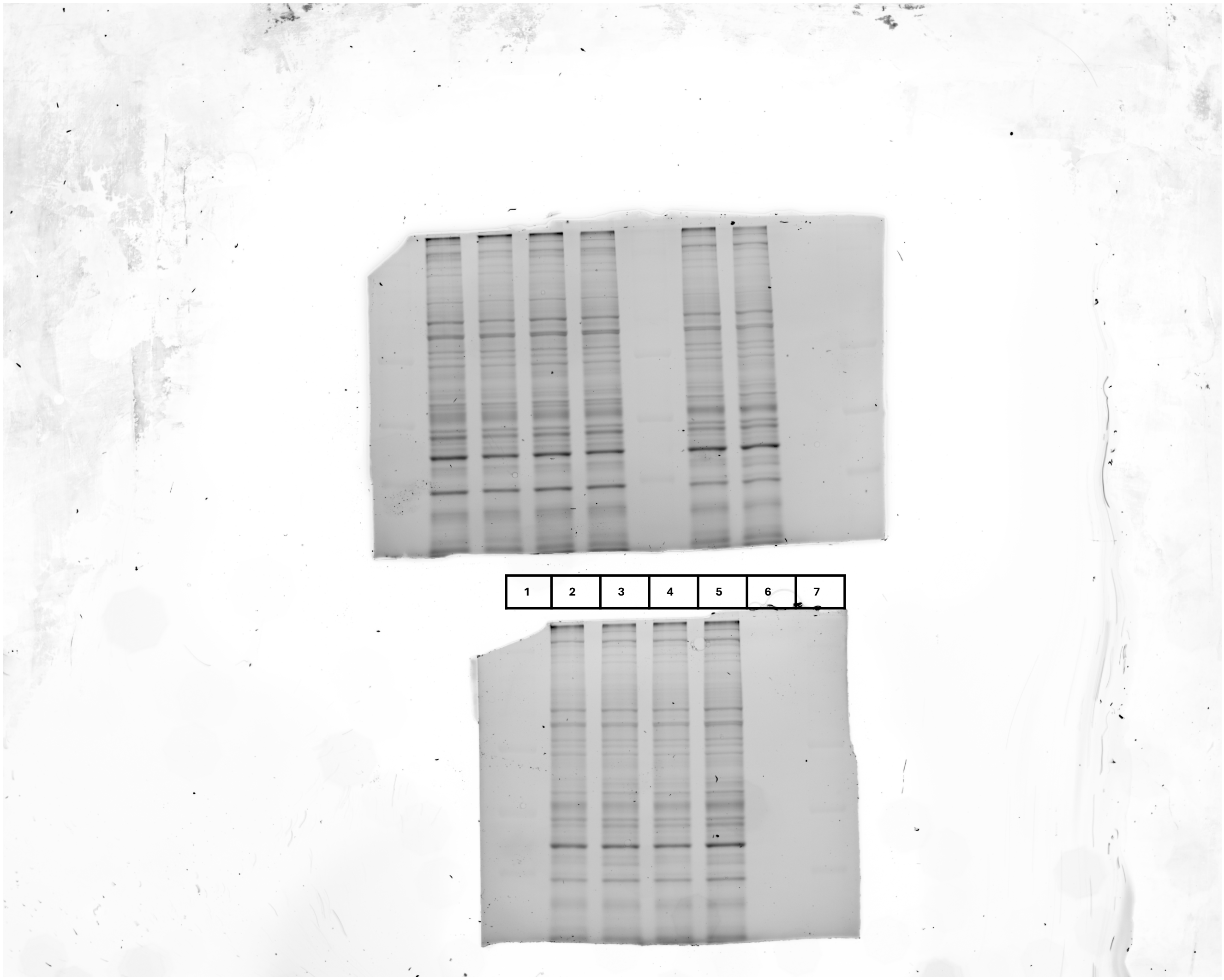

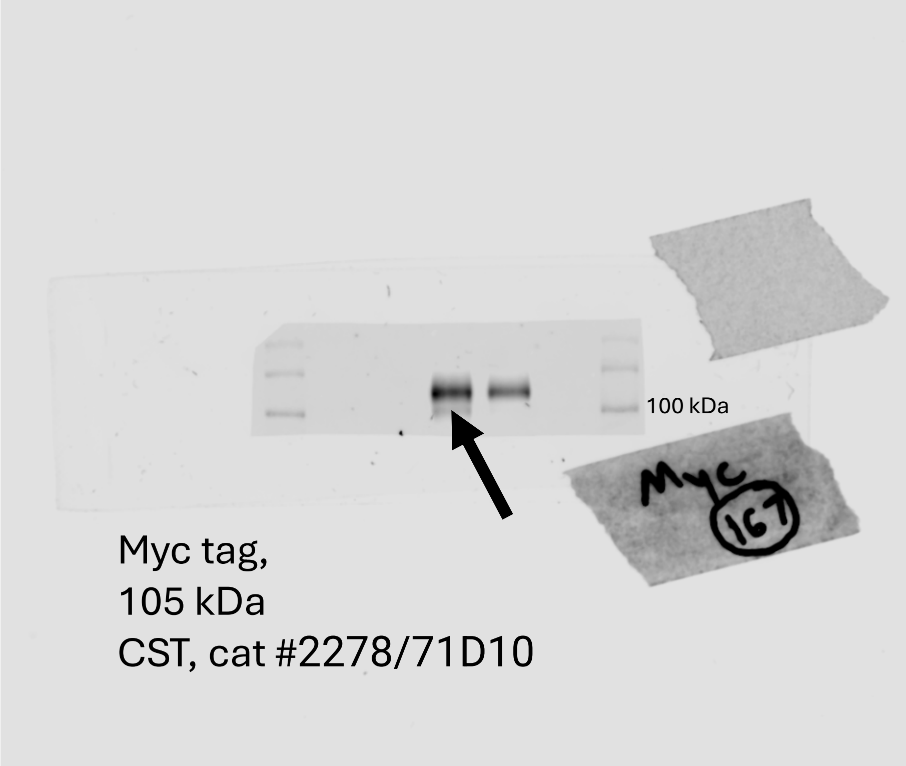

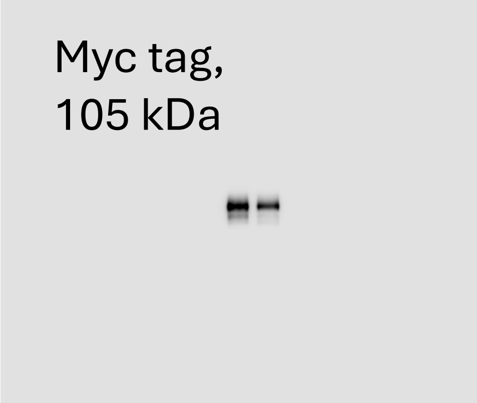

1	2	3	4	5	6	7
Ladder	NT5E WT EV	NT5E KO EV	NT5E WT, myc-β-catenin	NT5E KO, myc-β-catenin		Ladder

2G

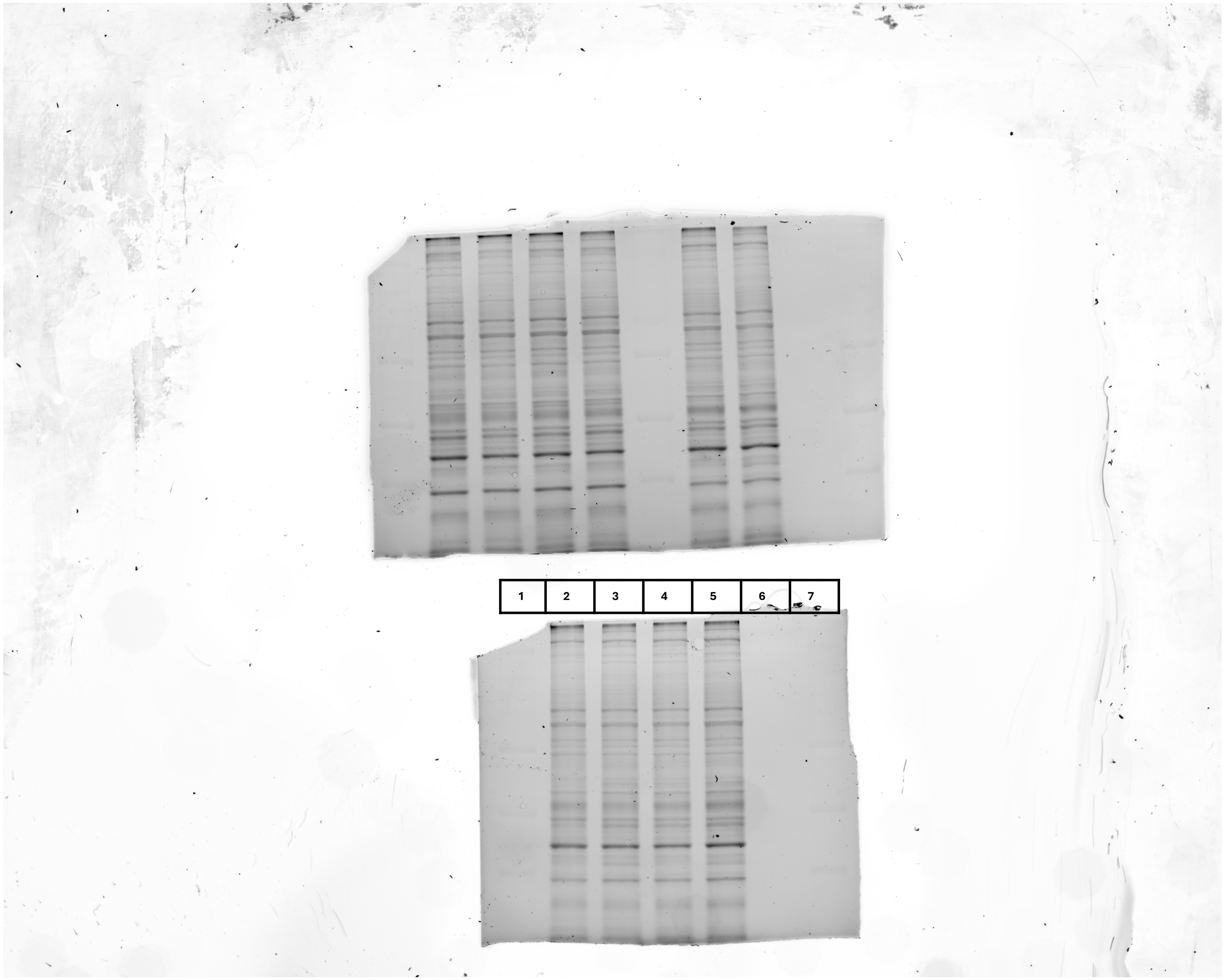

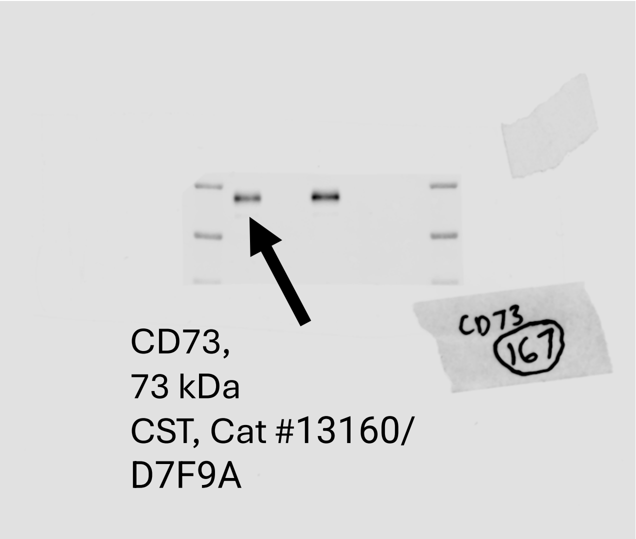

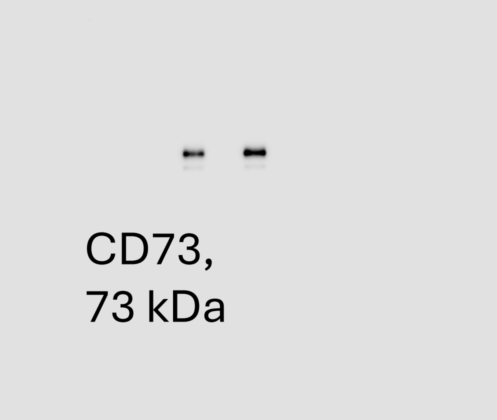

1	2	3	4	5	6	7
Ladder	NT5E WT<br>EV	NT5E KO<br>EV	NT5E WT, myc-<br>β-catenin	NT5E KO, myc-<br>β-catenin		Ladder

2I

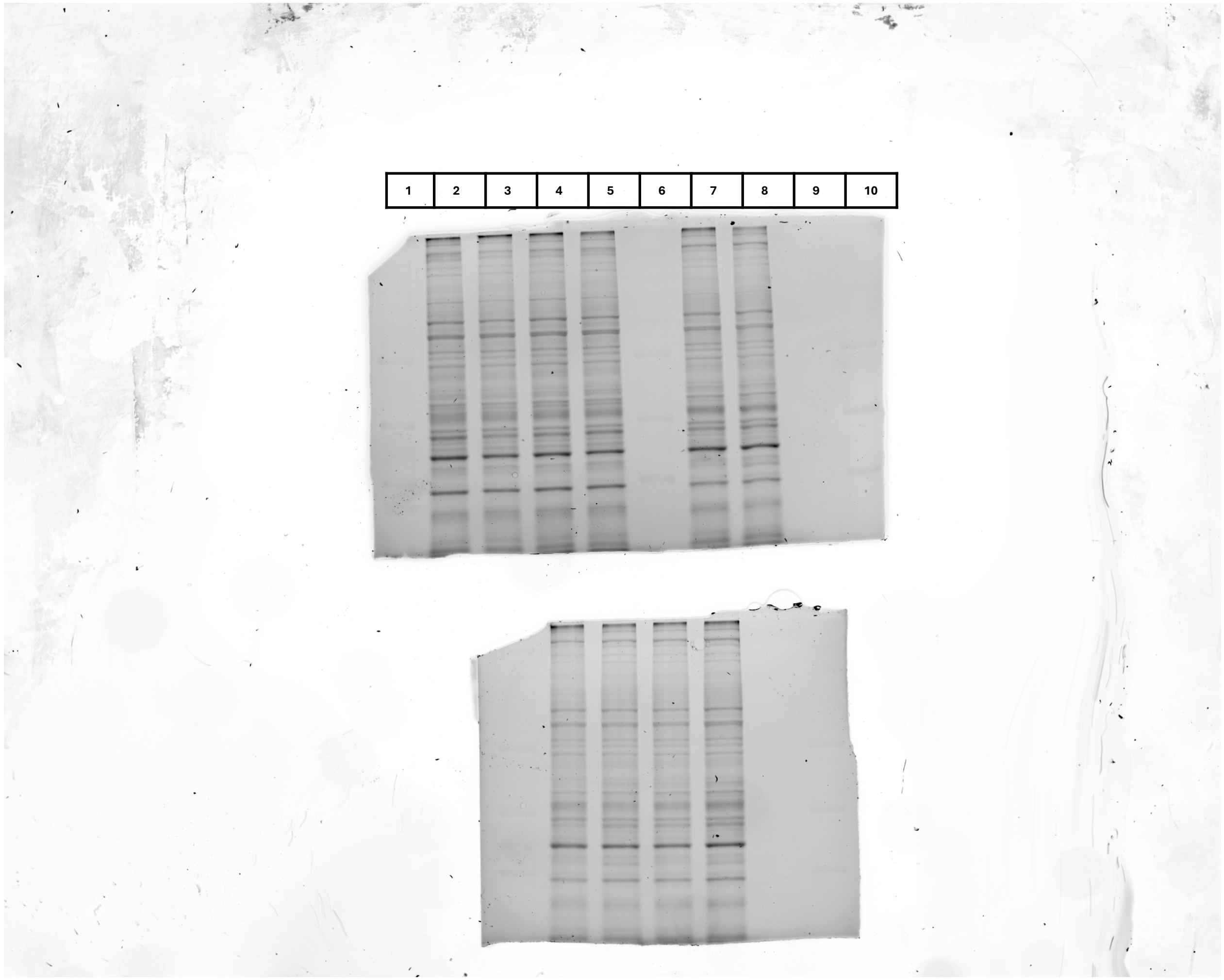

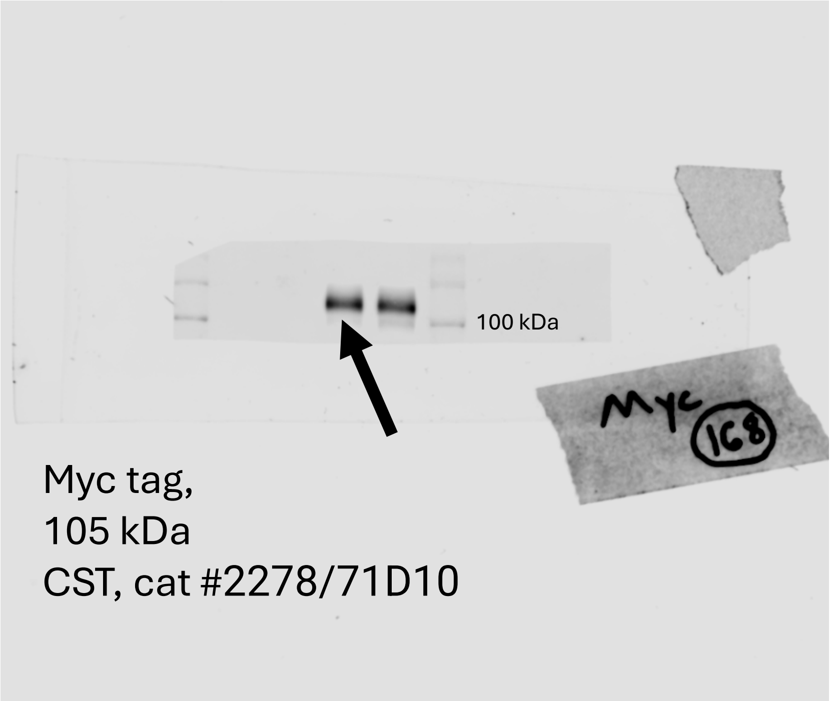

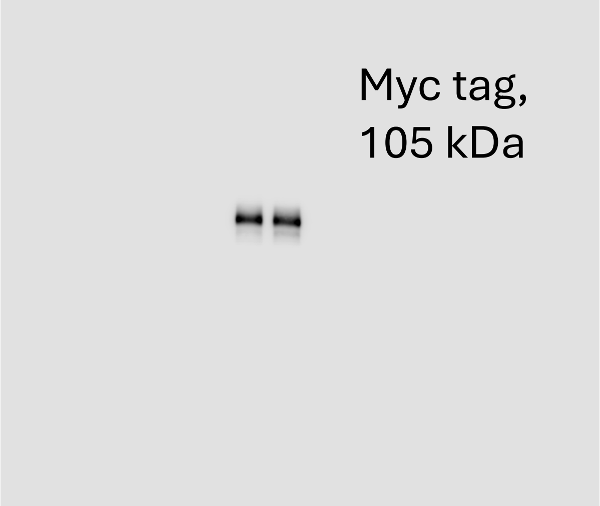

1	2	3	4	5	6	7	8	9	10
Ladder	Ishikawa Ctrl AdV EV	Ishikawa NT5E AdV EV	Ishikawa Ctrl AdV Myc-β-catenin	Ishikawa NT5E AdV Myc-β-catenin	Ladder	HEC-1-A NT5E WT	HEC-1-A NT5E KO		Ladder

2I

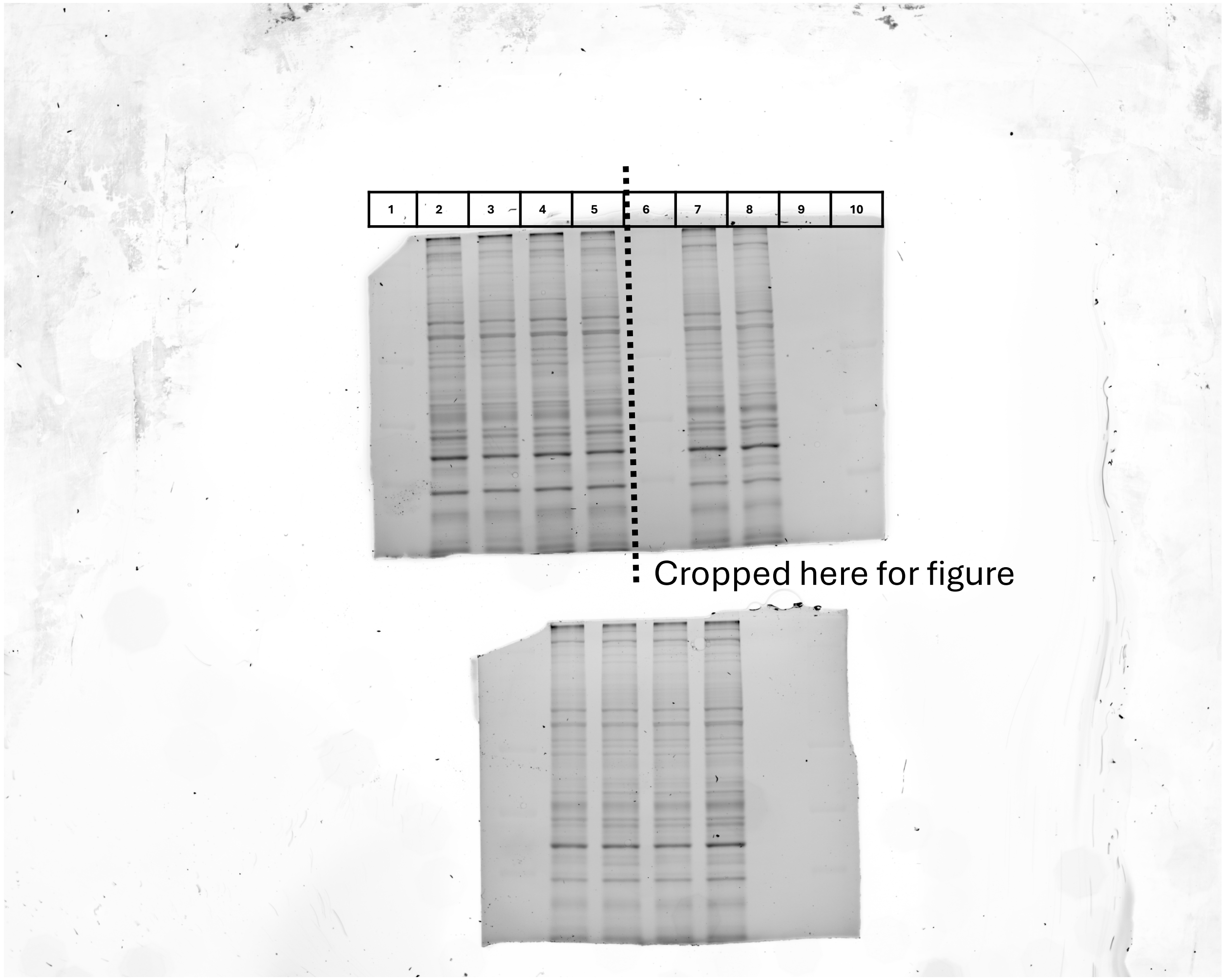

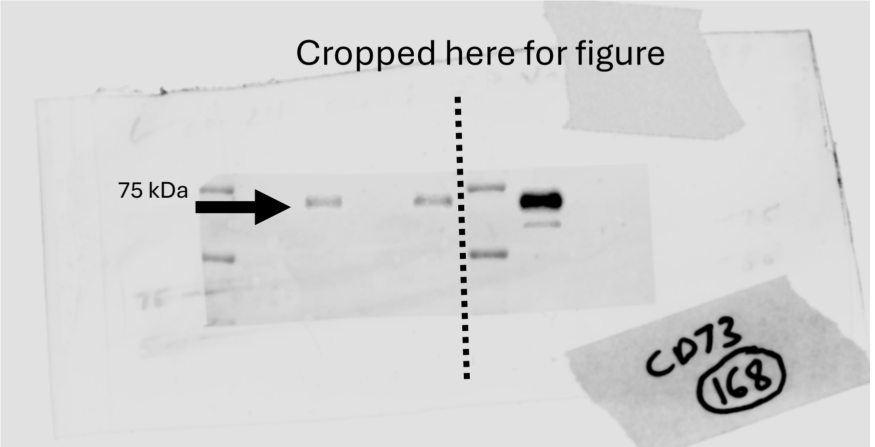

CD73,
73 kDa
CST, Cat #13160/
D7F9A

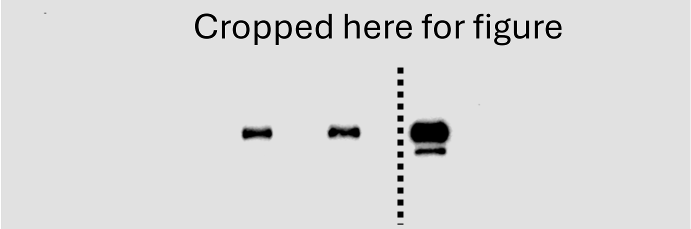

CD73,
73 kDa

1	2	3	4	5	6	7	8	9	10
Ladder	Ishikawa Ctrl AdV EV	Ishikawa NT5E AdV EV	Ishikawa Ctrl AdV Myc-β-catenin	Ishikawa NT5E AdV Myc-β-catenin	Ladder	HEC-1-A NT5E WT	HEC-1-A NT5E KO		Ladder

Figure 3

3B, CD73

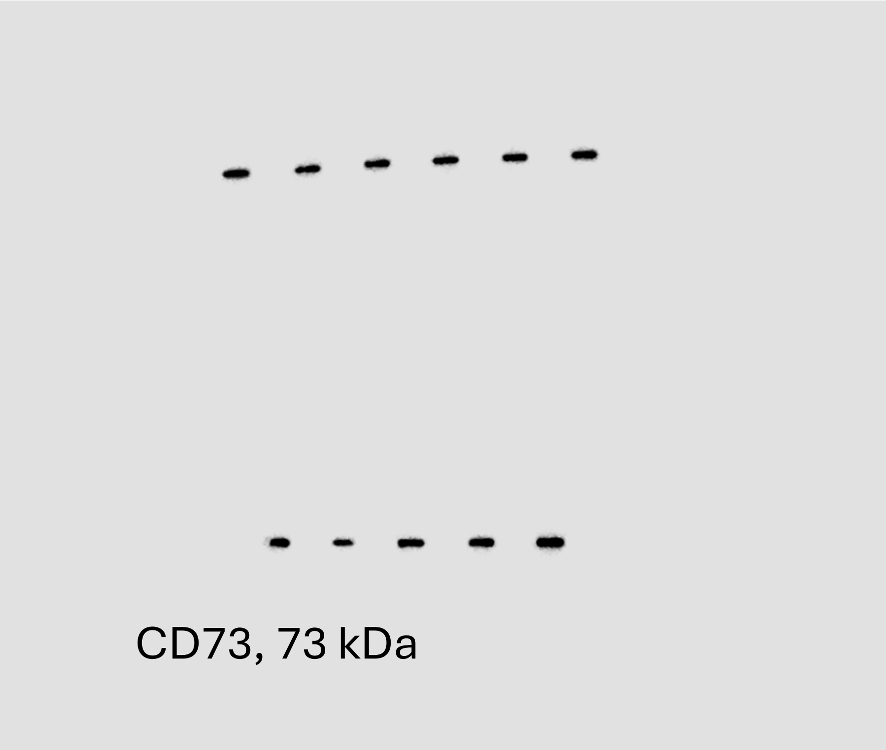

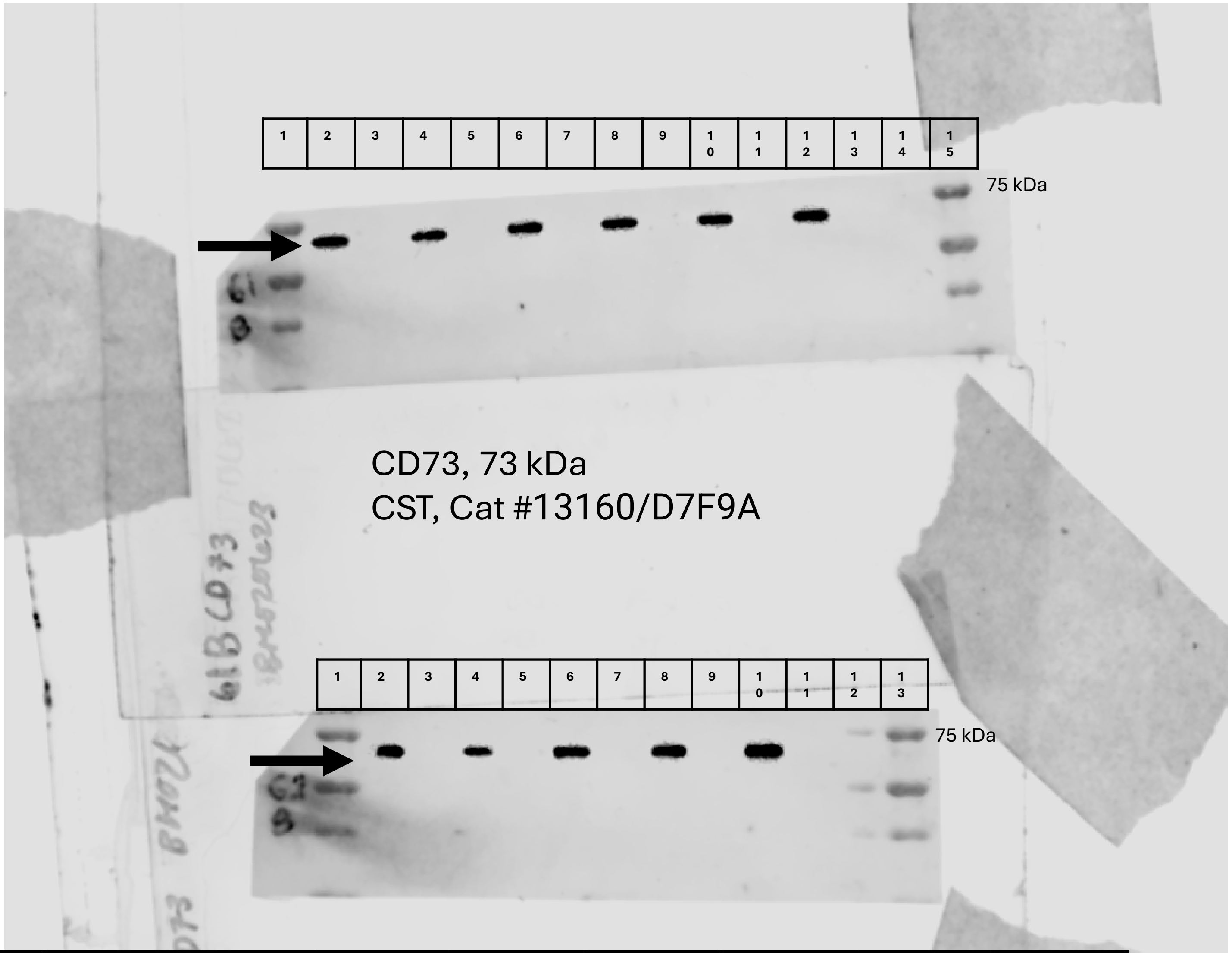

1	2	3	4	5	6	7	8	9	10	11	12	13	14	15
Ladder	NT5E WT No DNA	NT5E KO No DNA	NT5E WT WT	NT5E KO WT	NT5E WT D32N	NT5E KO D32N	NT5E WT S33F	NT5E KO S33F	NT5E WT S33Y	NT5E KO S33Y	NT5E WT G34R	NT5E KO G34R		Ladder

1	2	3	4	5	6	7	8	9	10	11	12	13
Ladder	NT5E WT WT	NT5E KO WT	NT5E WT D32N	NT5E KO D32N	NT5E WT S37C	NT5E KO S37C	NT5E WT S37F	NT5E KO S37F	NT5E WT S45F	NT5E KO S45F		Ladder

3B

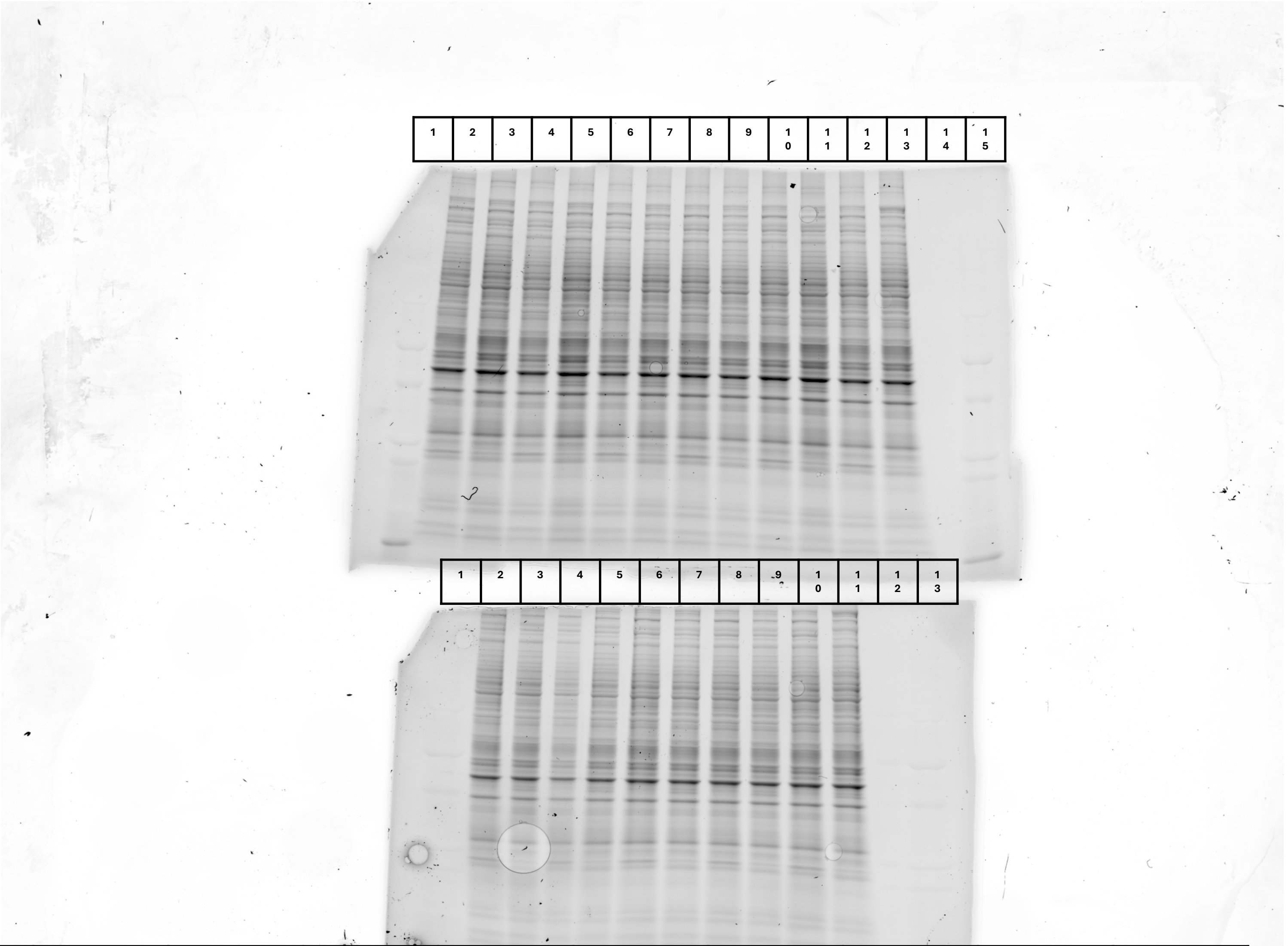

Total protein, 61 & 62

1	2	3	4	5	6	7	8	9	10	11	12	13	14	15
Ladder	NT5E WT No DNA	NT5E KO No DNA	NT5E WT WT	NT5E KO WT	NT5E WT D32N	NT5E KO D32N	NT5E WT S33F	NT5E KO S33F	NT5E WT S33Y	NT5E KO S33Y	NT5E WT G34R	NT5E KO G34R		Ladder

1	2	3	4	5	6	7	8	9	10	11	12	13
Ladder	NT5E WT WT	NT5E KO WT	NT5E WT D32N	NT5E KO D32N	NT5E WT S37C	NT5E KO S37C	NT5E WT S37F	NT5E KO S37F	NT5E WT S45F	NT5E KO S45F		Ladder

3B, Myc

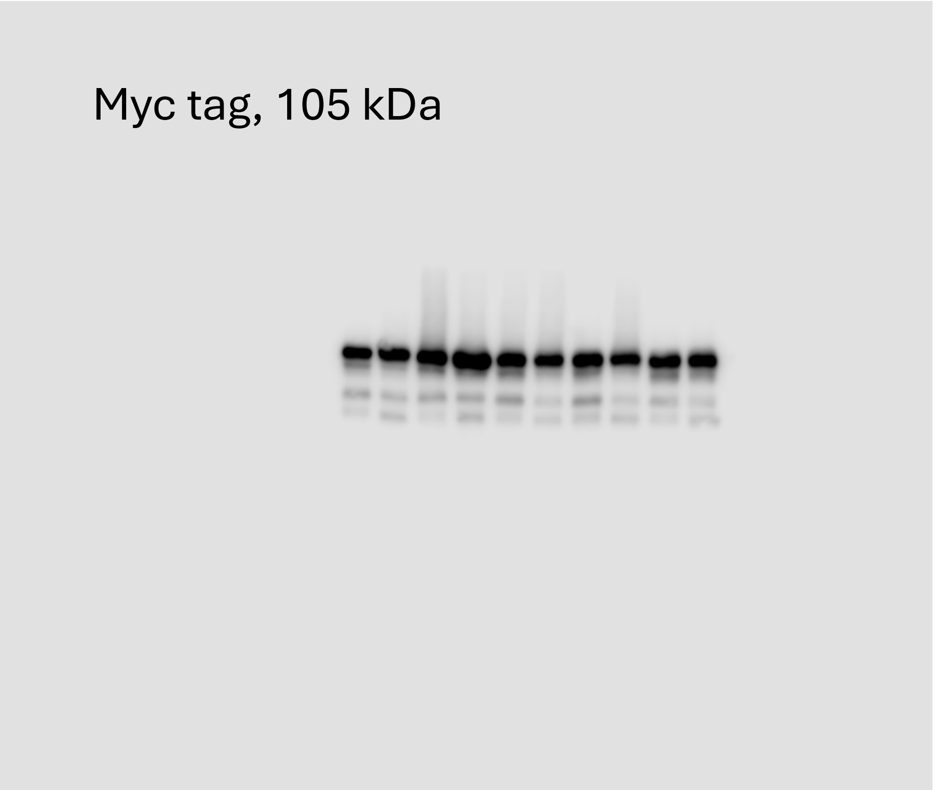

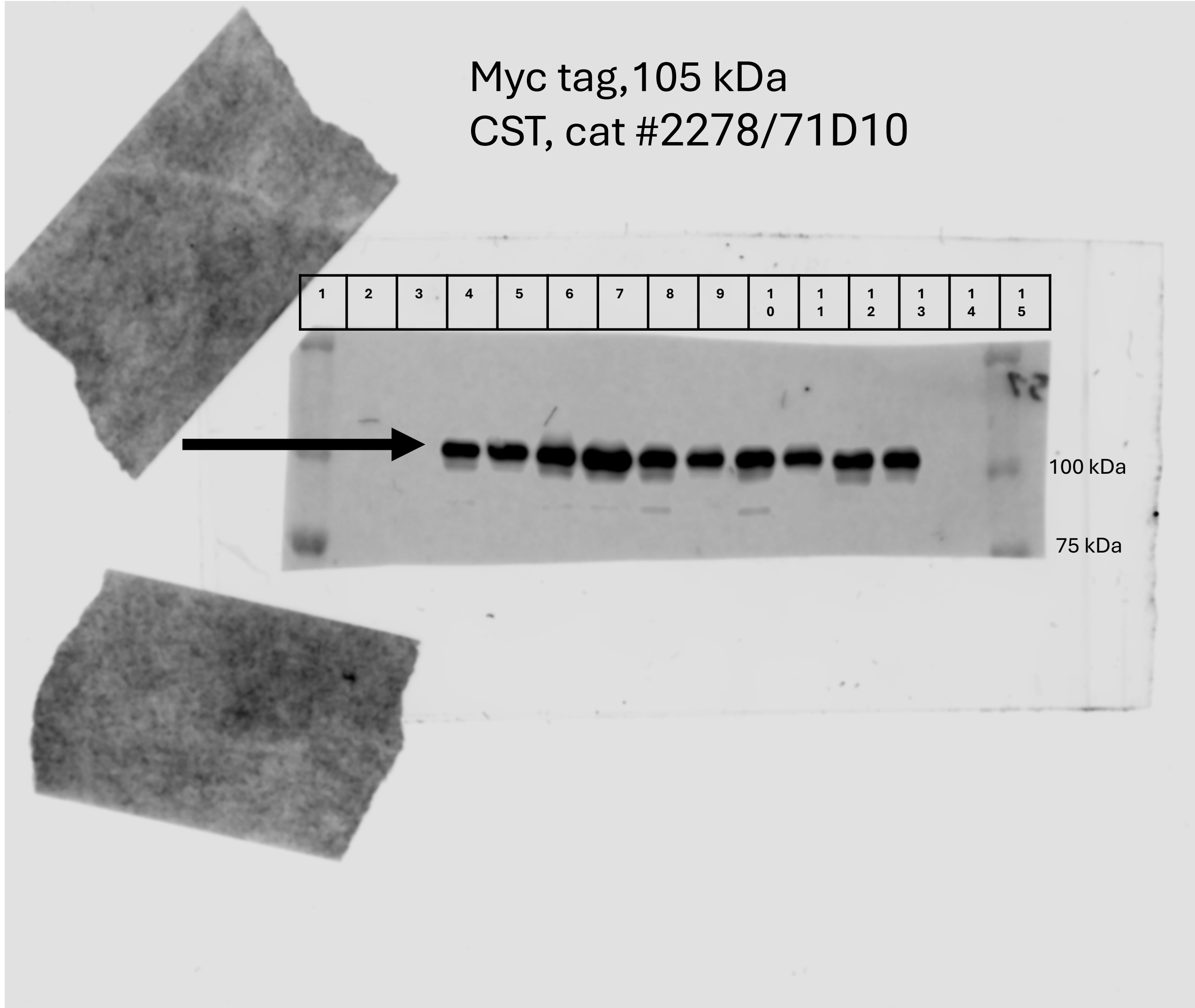

1	2	3	4	5	6	7	8	9	10	11	12	13	14	15
Ladder	NT5E WT No DNA	NT5E KO No DNA	NT5E WT WT	NT5E KO WT	NT5E WT D32N	NT5E KO D32N	NT5E WT S33F	NT5E KO S33F	NT5E WT S33Y	NT5E KO S33Y	NT5E WT G34R	NT5E KO G34R		Ladder

3B, Myc

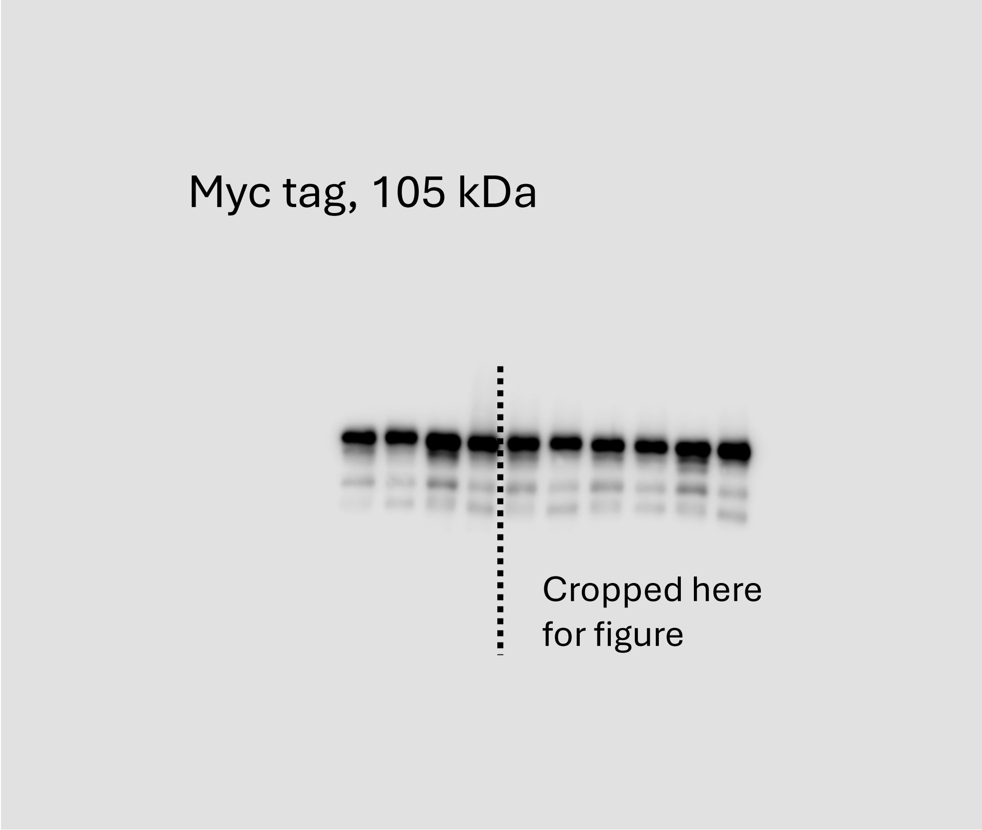

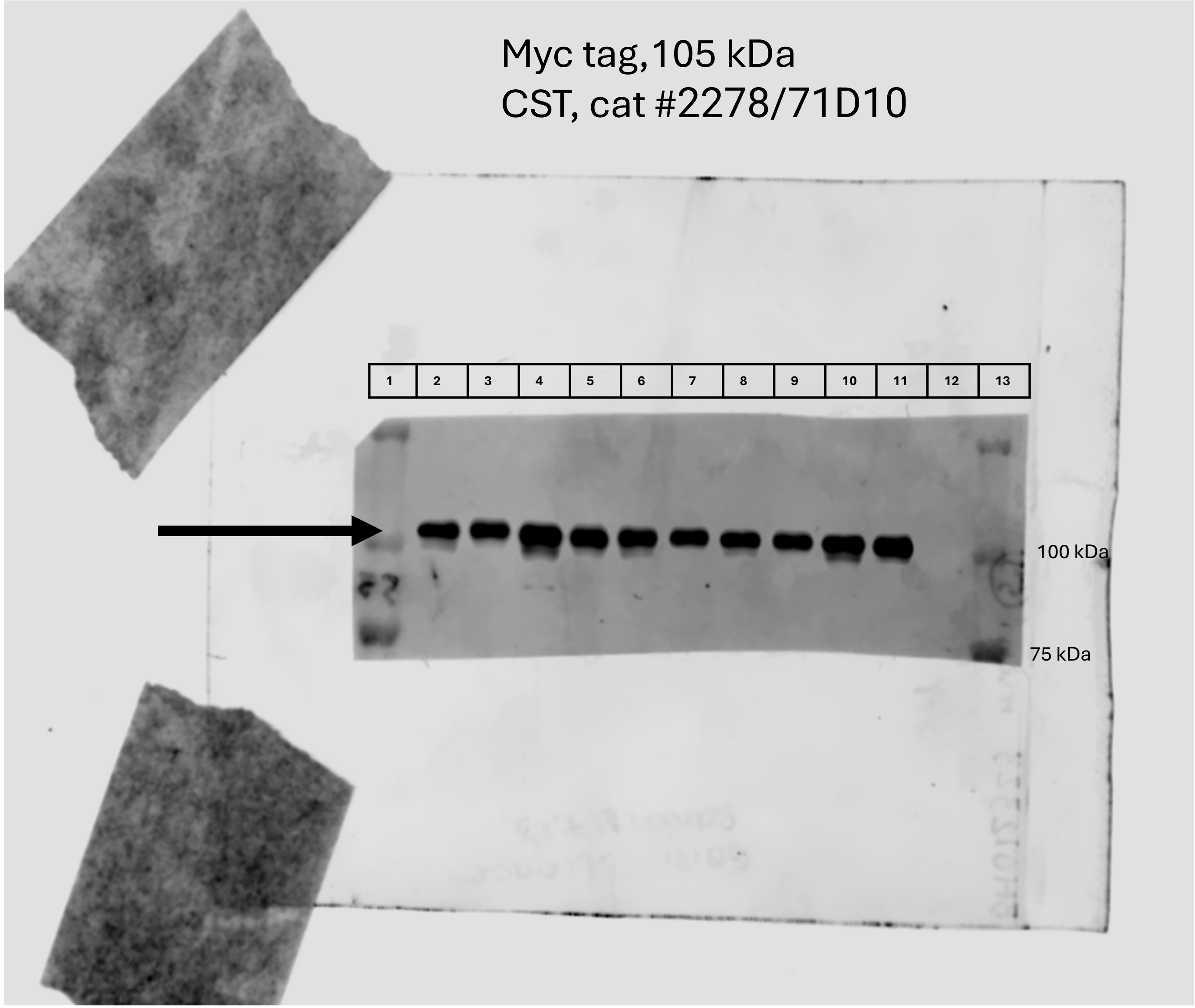

1	2	3	4	5	6	7	8	9	10	11	12	13
Ladder	NT5E WT WT	NT5E KO WT	NT5E WT D32N	NT5E KO D32N	NT5E WT S37C	NT5E KO S37C	NT5E WT S37F	NT5E KO S37F	NT5E WT S45F	NT5E KO S45F		Ladder

3B

Total protein, 57 & 58

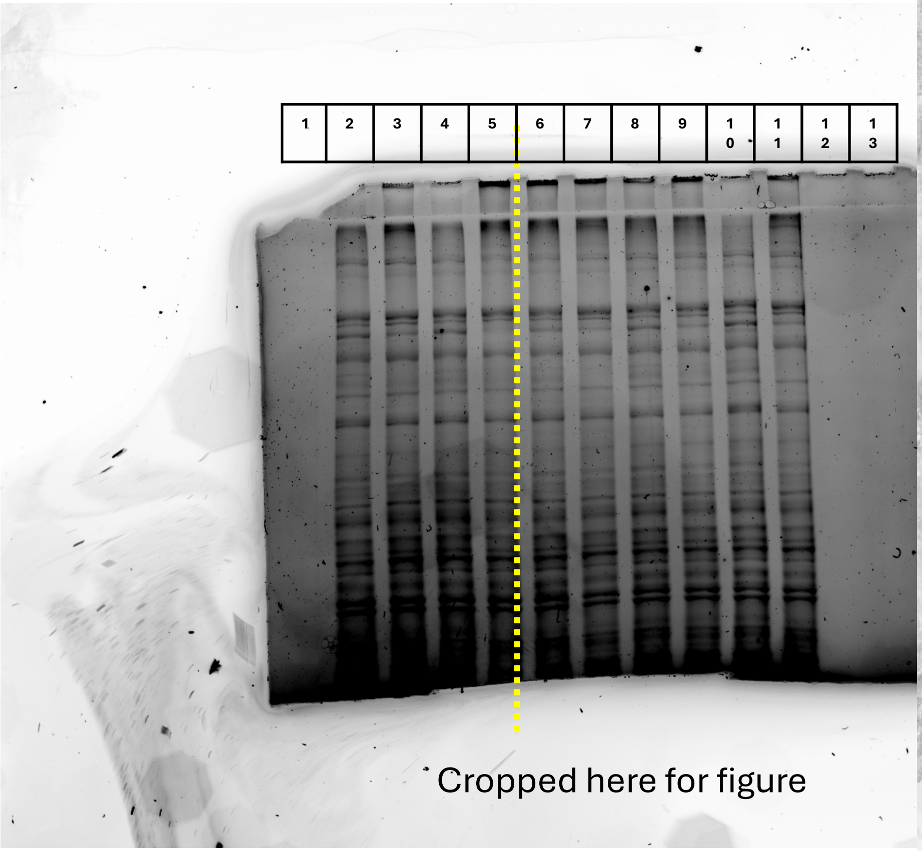

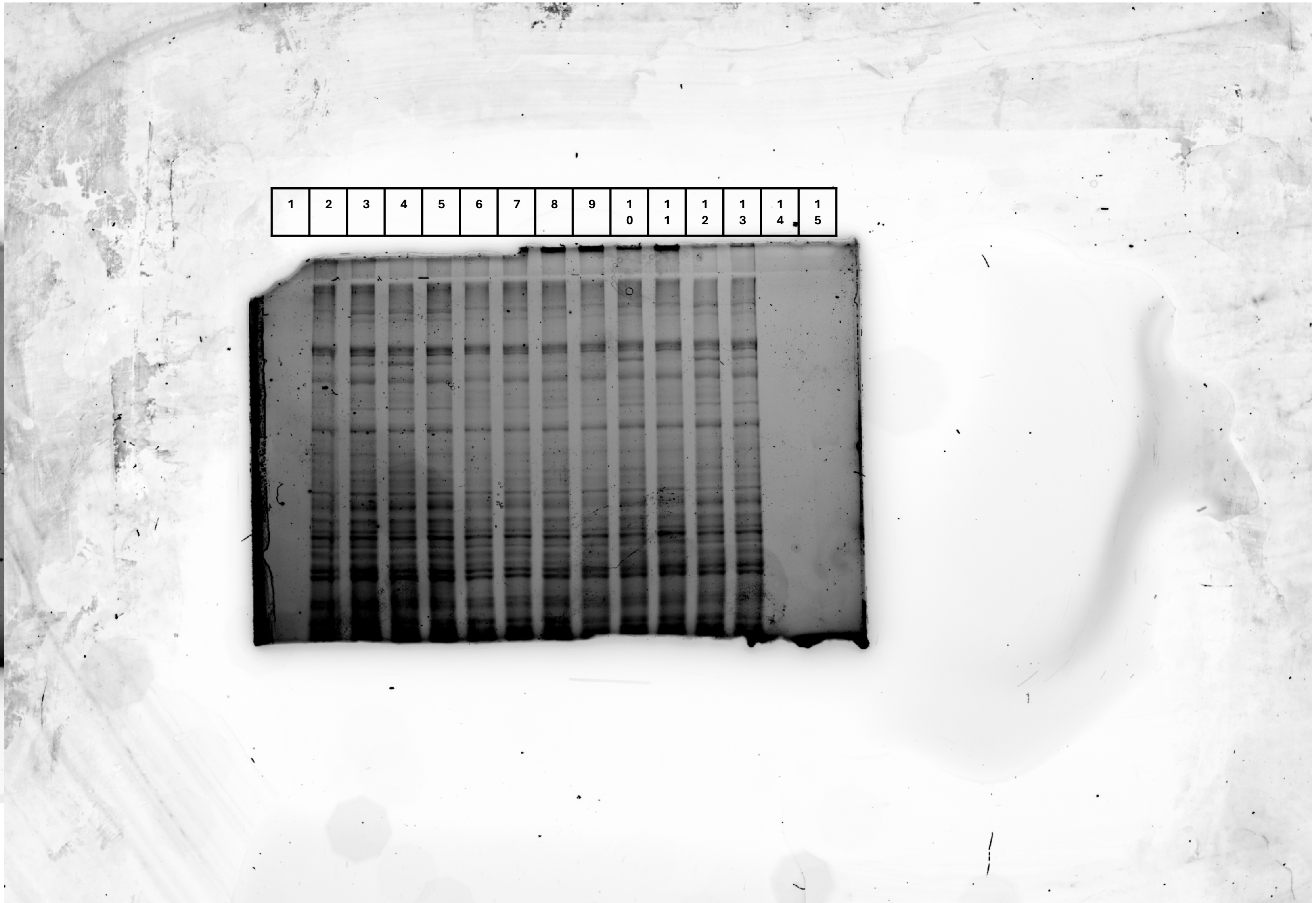

1	2	3	4	5	6	7	8	9	10	11	12	13	14	15
Ladder	NT5E WT No DNA	NT5E KO No DNA	NT5E WT WT	NT5E KO WT	NT5E WT D32N	NT5E KO D32N	NT5E WT S33F	NT5E KO S33F	NT5E WT S33Y	NT5E KO S33Y	NT5E WT G34R	NT5E KO G34R		Ladder

1	2	3	4	5	6	7	8	9	10	11	12	13
Ladder	NT5E WT WT	NT5E KO WT	NT5E WT D32N	NT5E KO D32N	NT5E WT S37C	NT5E KO S37C	NT5E WT S37F	NT5E KO S37F	NT5E WT S45F	NT5E KO S45F		Ladder

3B

Total protein, 57 & 58 – brightened images for figure in paper

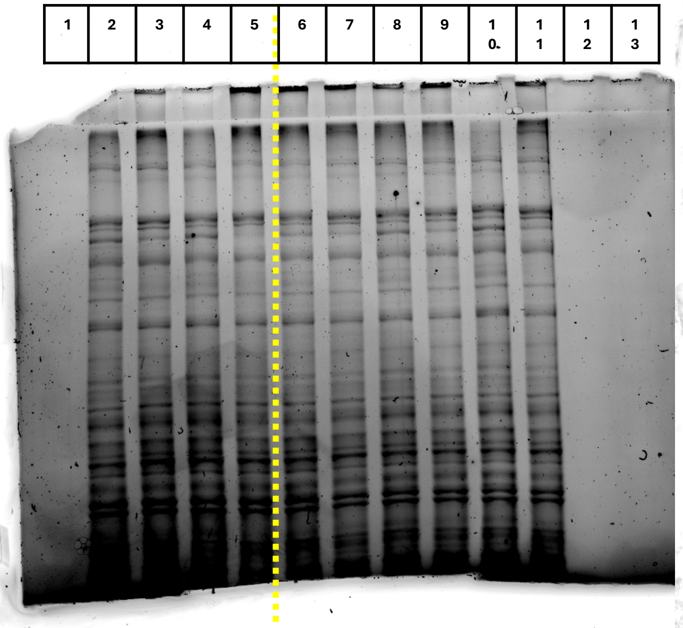

Cropped here for figure

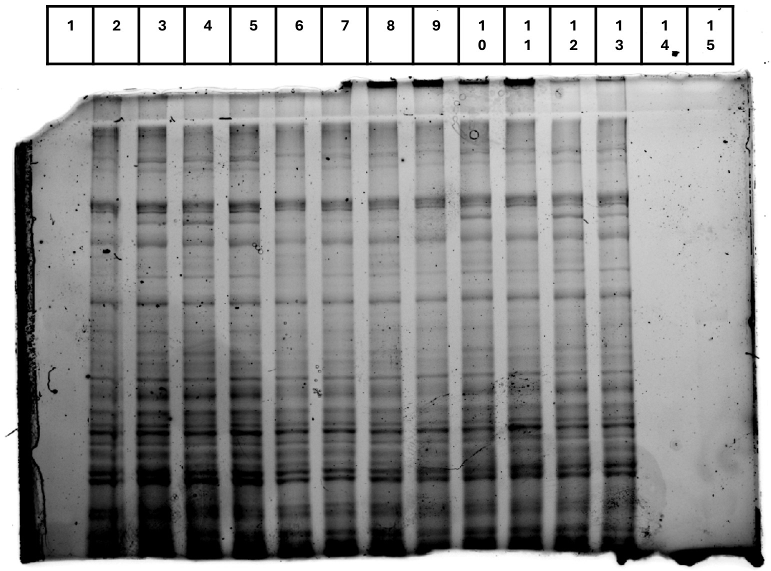

1	2	3	4	5	6	7	8	9	10	11	12	13	14	15
Ladder	NT5E WT No DNA	NT5E KO No DNA	NT5E WT WT	NT5E KO WT	NT5E WT D32N	NT5E KO D32N	NT5E WT S33F	NT5E KO S33F	NT5E WT S33Y	NT5E KO S33Y	NT5E WT G34R	NT5E KO G34R		Ladder

1	2	3	4	5	6	7	8	9	10	11	12	13
Ladder	NT5E WT WT	NT5E KO WT	NT5E WT D32N	NT5E KO D32N	NT5E WT S37C	NT5E KO S37C	NT5E WT S37F	NT5E KO S37F	NT5E WT S45F	NT5E KO S45F		Ladder

3B, β-catenin

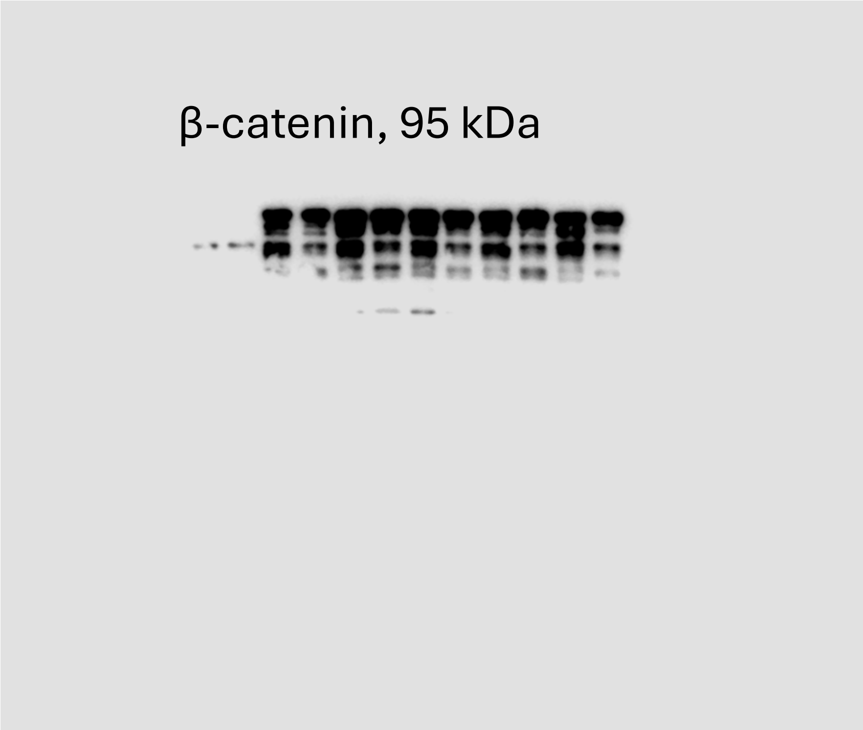

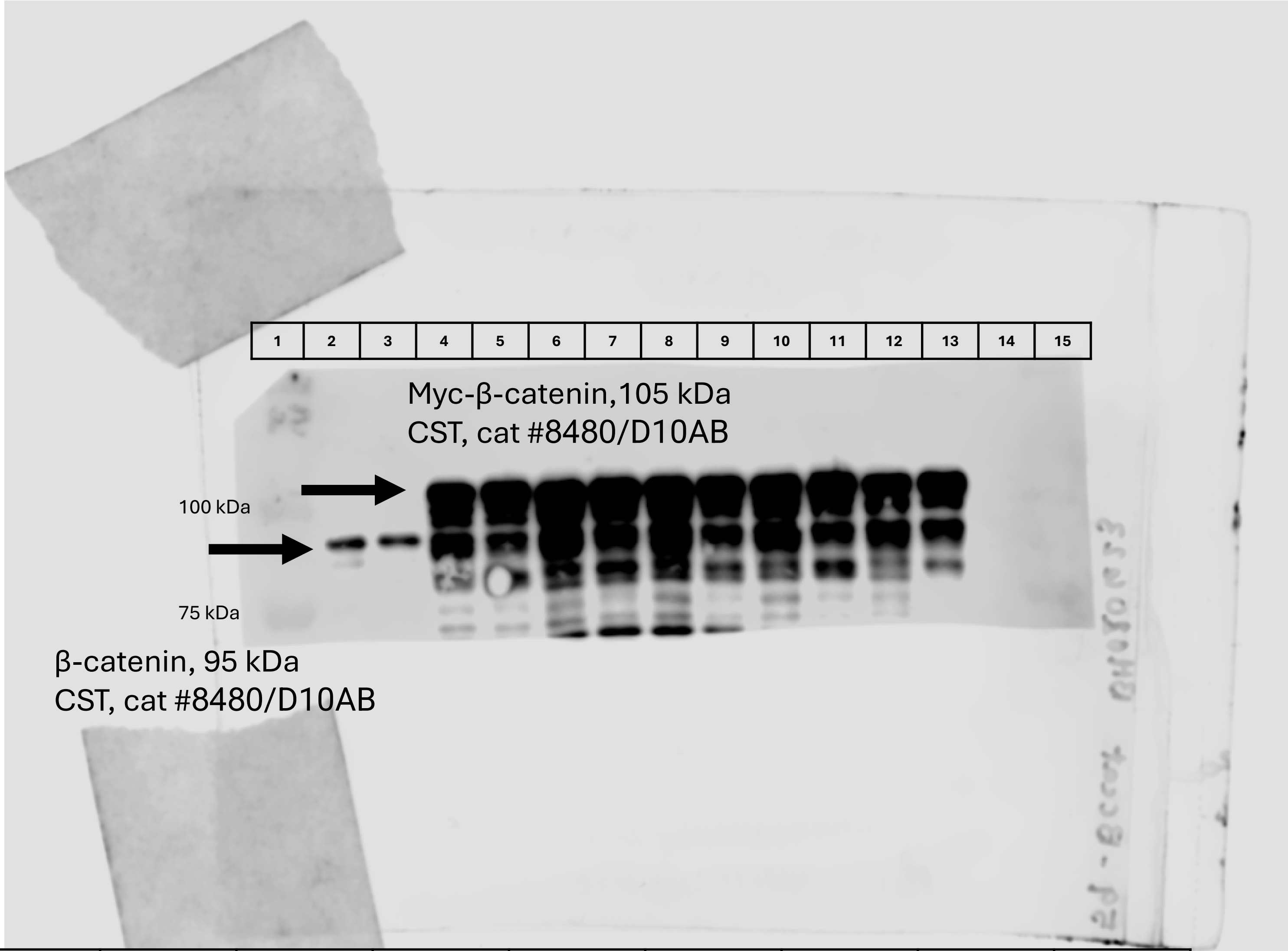

1	2	3	4	5	6	7	8	9	10	11	12	13	14	15
Ladder	NT5E WT No DNA	NT5E KO No DNA	NT5E WT WT	NT5E KO WT	NT5E WT D32N	NT5E KO D32N	NT5E WT S33F	NT5E KO S33F	NT5E WT S33Y	NT5E KO S33Y	NT5E WT G34R	NT5E KO G34R		Ladder

3B, β-catenin

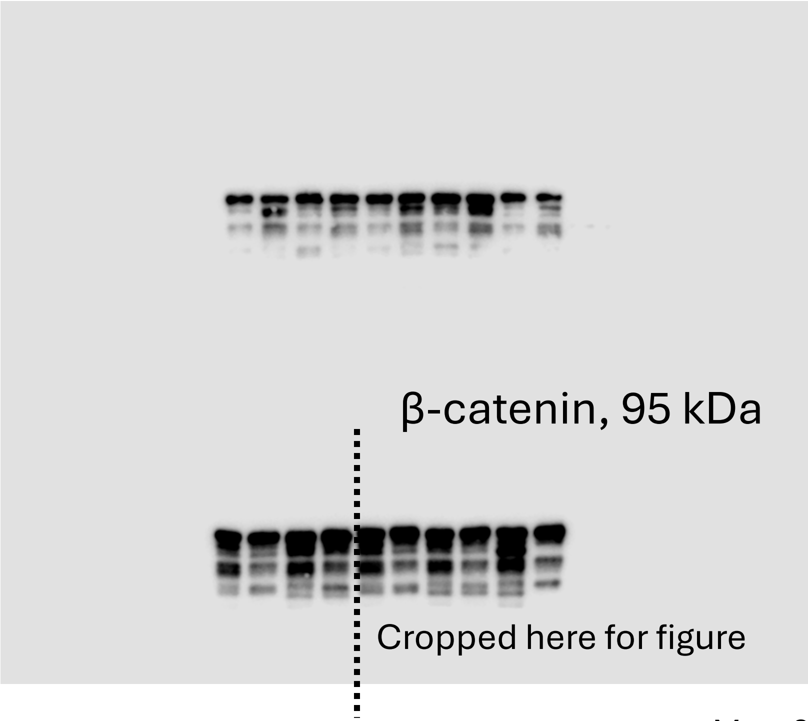

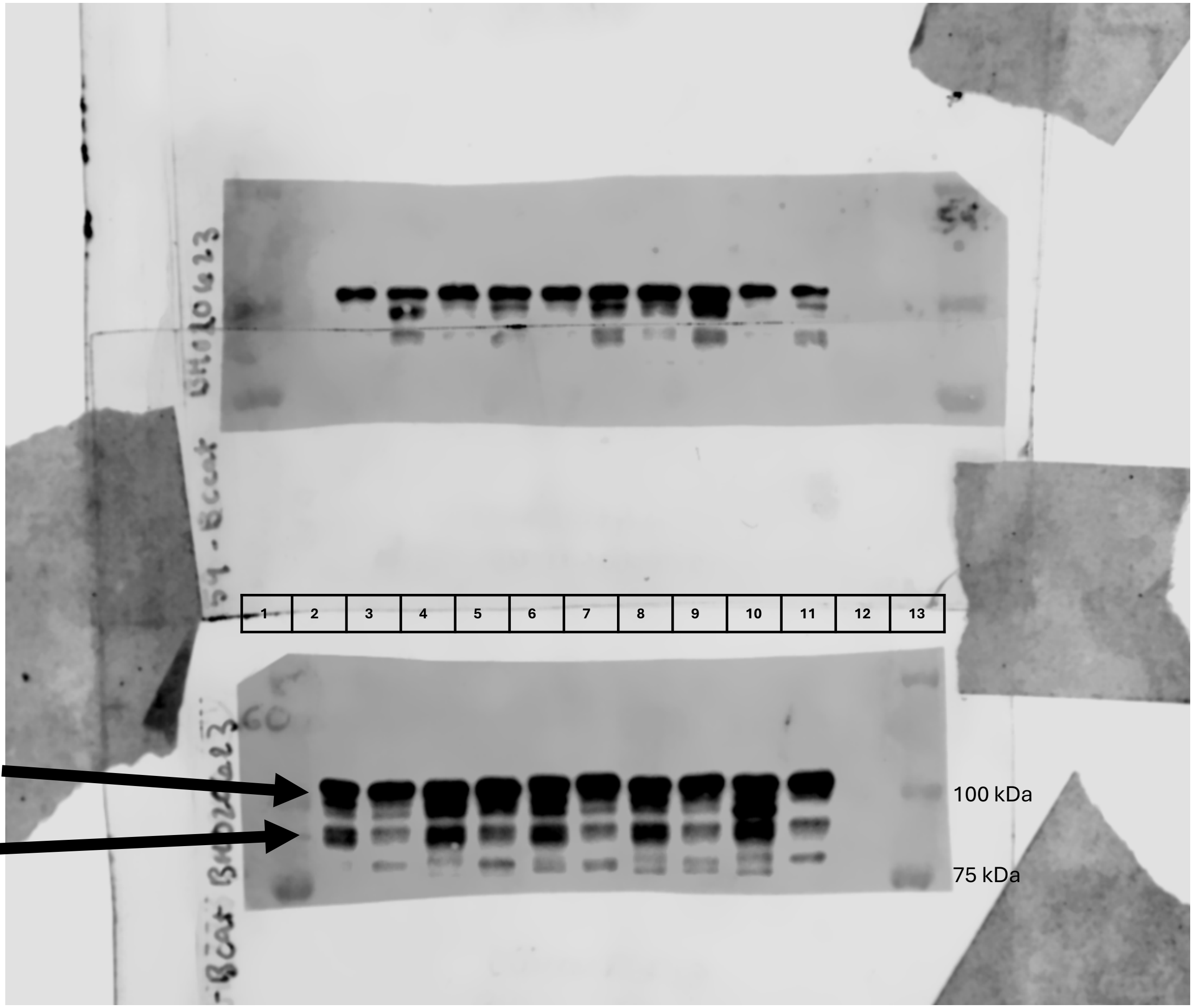

Myc-β-catenin,105 kDa
CST, cat #8480/D10AB

β-catenin,95 kDa
CST, cat #8480/D10AB

1	2	3	4	5	6	7	8	9	10	11	12	13
Ladder	NT5E WT WT	NT5E KO WT	NT5E WT D32N	NT5E KO D32N	NT5E WT S37C	NT5E KO S37C	NT5E WT S37F	NT5E KO S37F	NT5E WT S45F	NT5E KO S45F		Ladder

3B

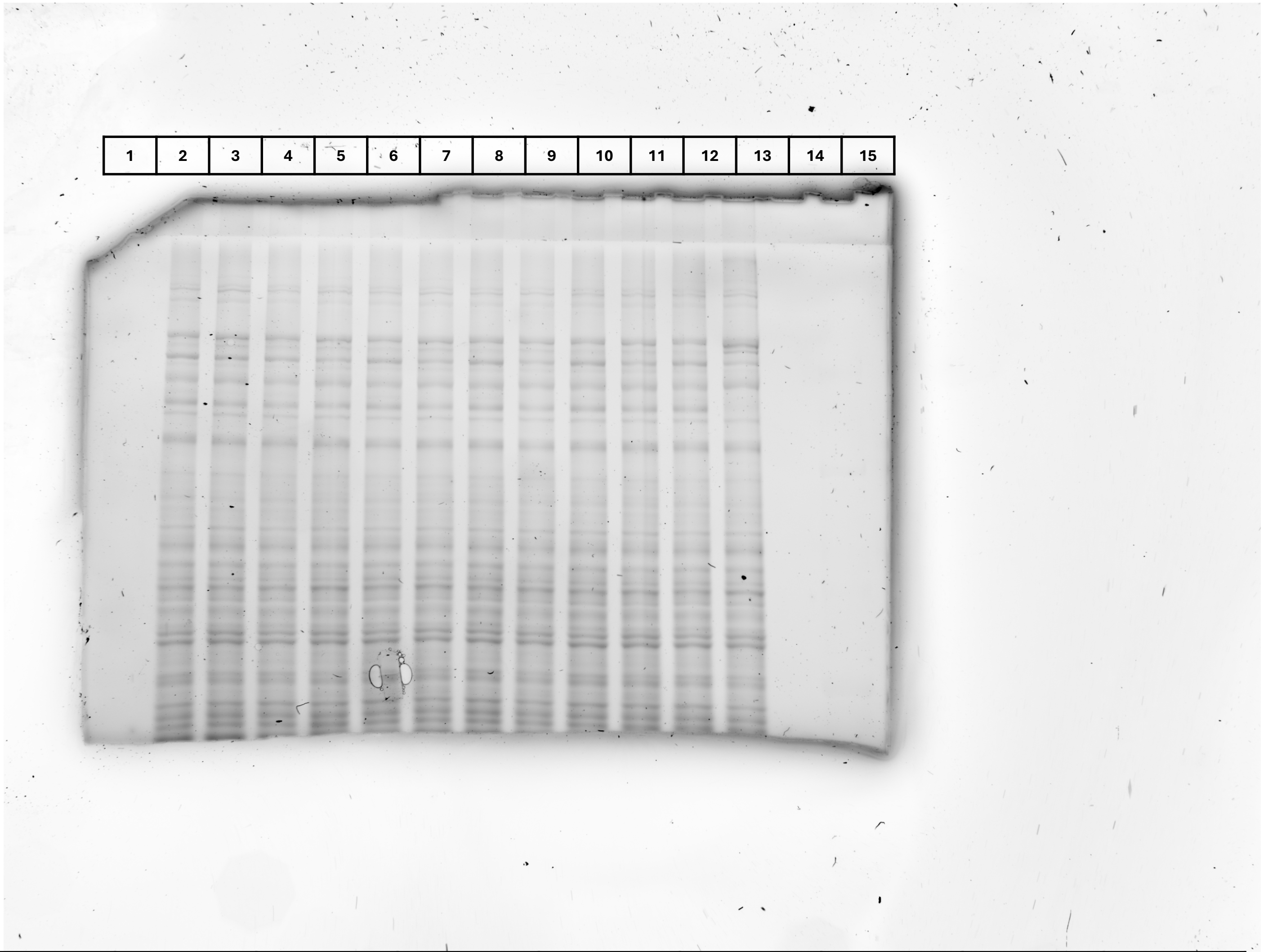

Total protein, 59

1	2	3	4	5	6	7	8	9	10	11	12	13	14	15
Ladder	NT5E WT No DNA	NT5E KO No DNA	NT5E WT WT	NT5E KO WT	NT5E WT D32N	NT5E KO D32N	NT5E WT S33F	NT5E KO S33F	NT5E WT S33Y	NT5E KO S33Y	NT5E WT G34R	NT5E KO G34R		Ladder

3B

Raw image
(gels are backwards)

Flipped
horizontal
for figure

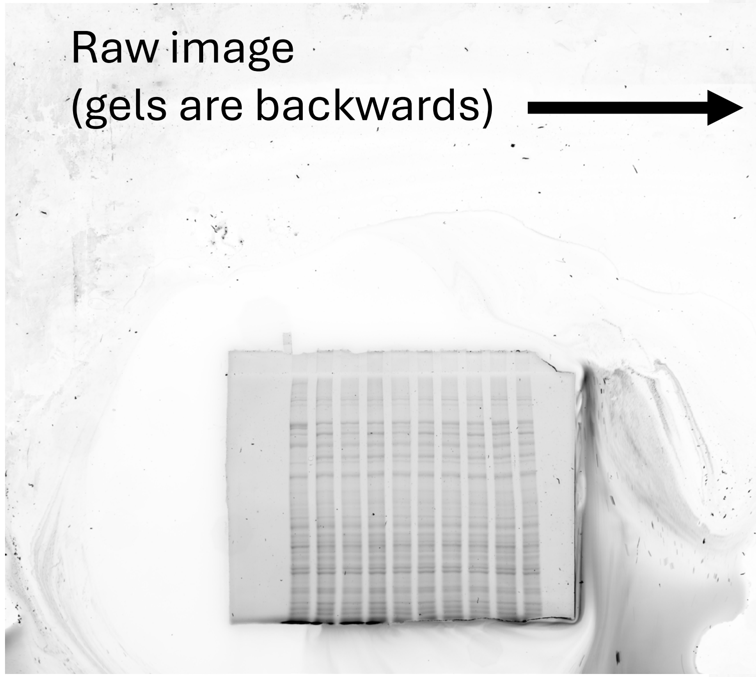

Total protein, 60

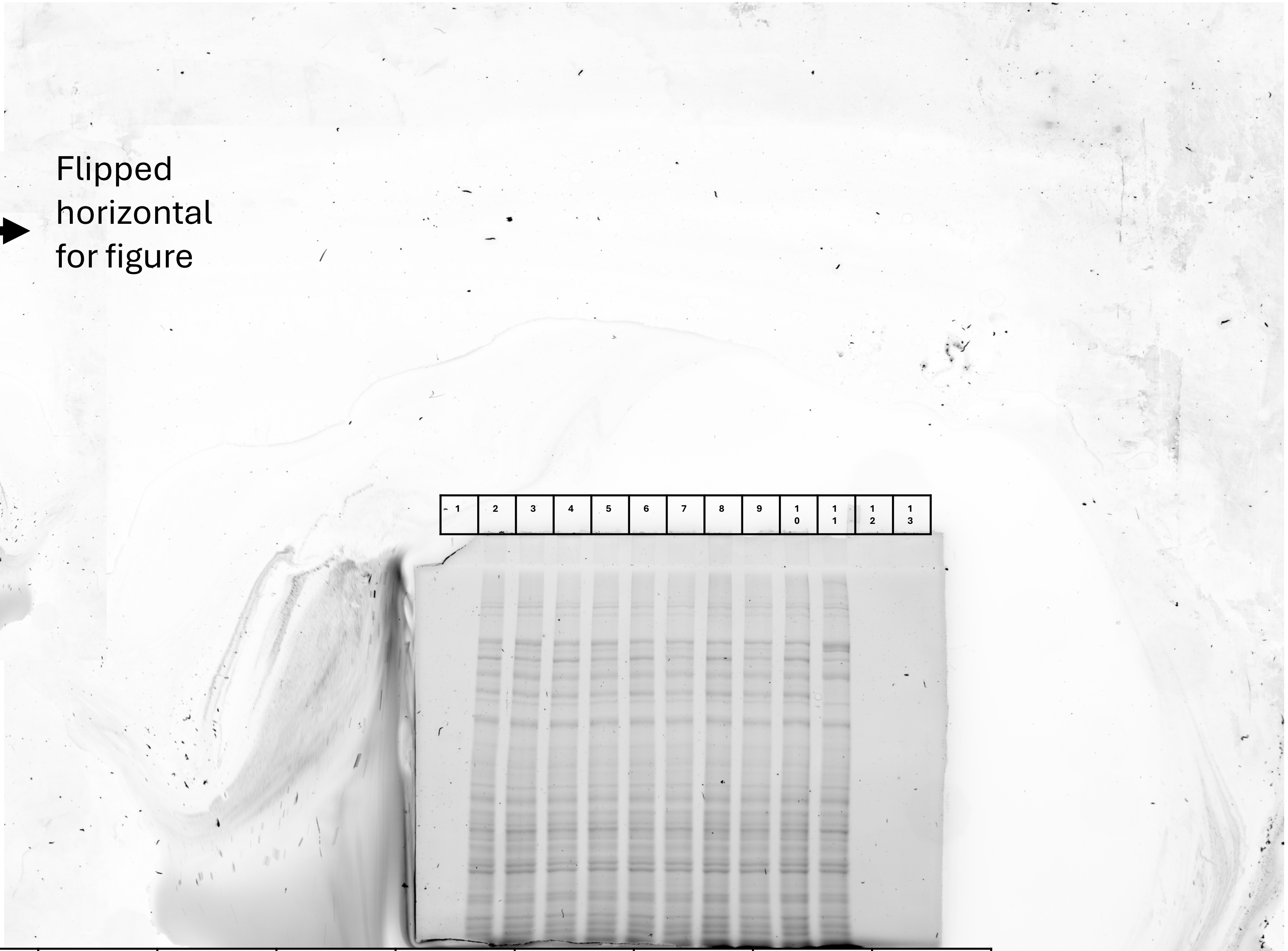

1	2	3	4	5	6	7	8	9	10	11	12	13
Ladder	NT5E WT WT	NT5E KO WT	NT5E WT D32N	NT5E KO D32N	NT5E WT S37C	NT5E KO S37C	NT5E WT S37F	NT5E KO S37F	NT5E WT S45F	NT5E KO S45F		Ladder

3D, β-catenin

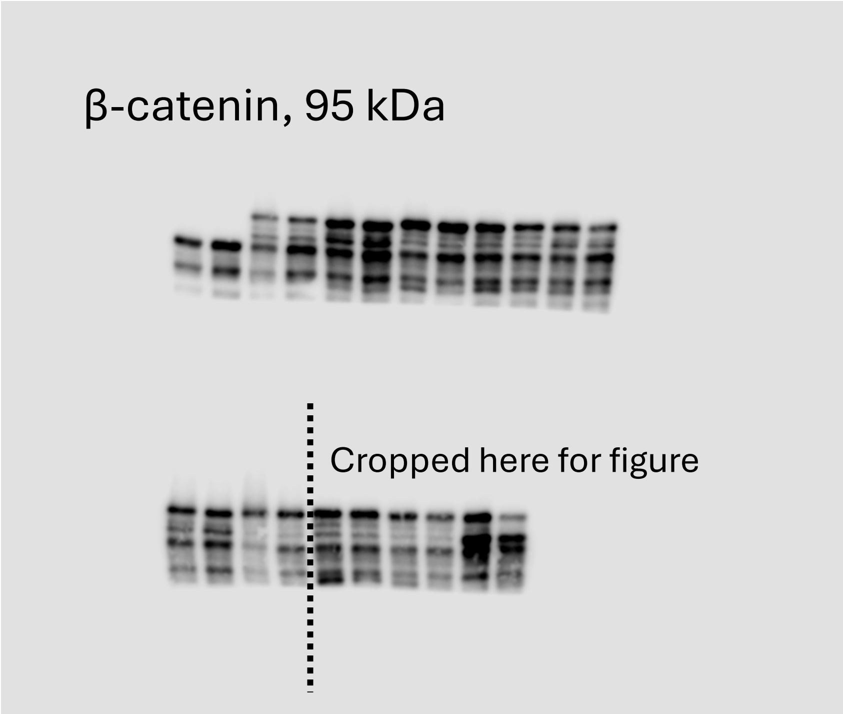

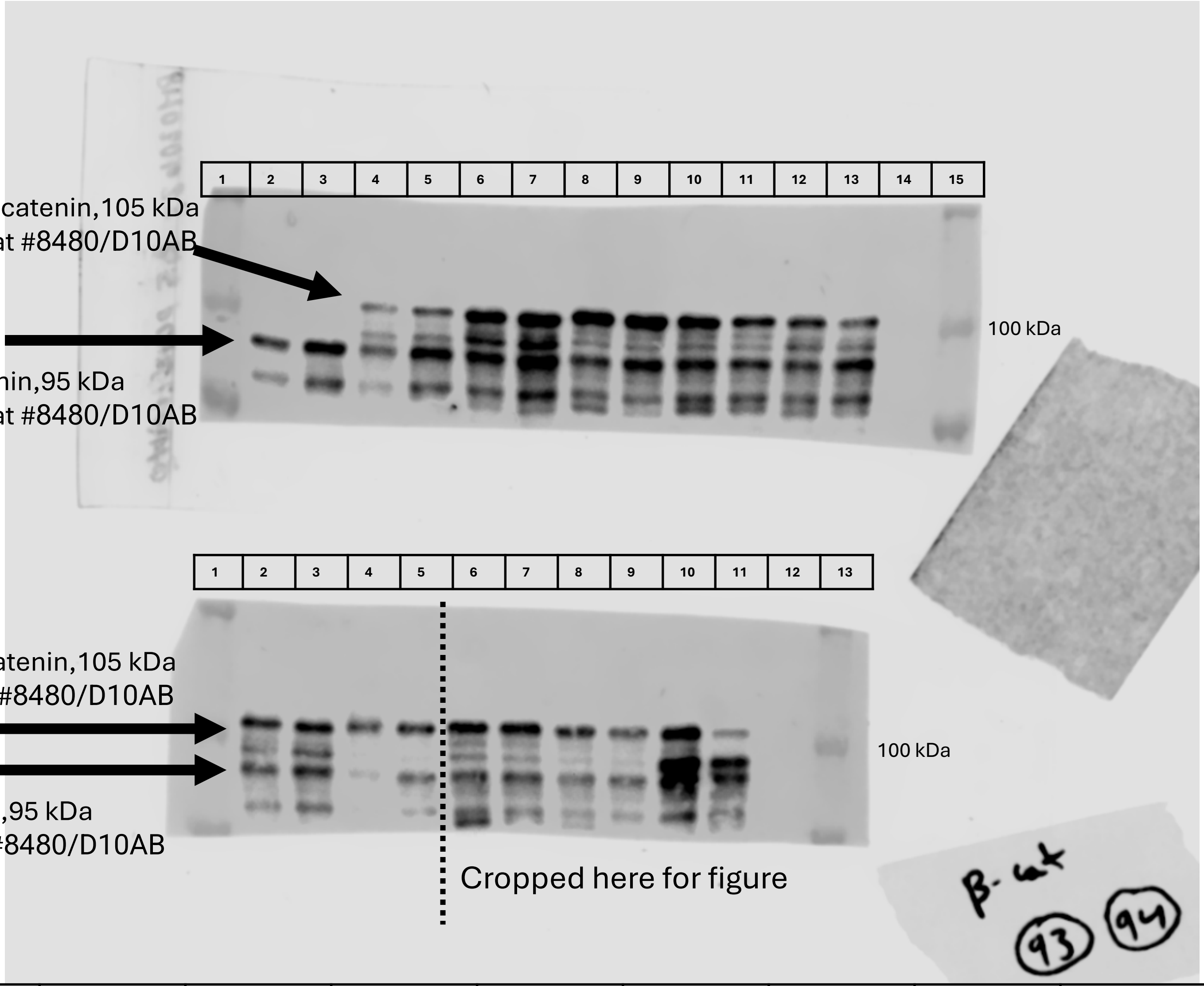

1	2	3	4	5	6	7	8	9	10	11	12	13	14	15
Ladder	Ishikawa EV No DNA	Ishikawa NT5E No DNA	Ishikawa EV WT	Ishikawa NT5E WT	Ishikawa EV D32N	Ishikawa NT5E D32N	Ishikawa EV S33F	Ishikawa NT5E S33F	Ishikawa EV S33Y	Ishikawa NT5E S33Y	Ishikawa EV G34R	Ishikawa NT5E G34R		Ladder

1	2	3	4	5	6	7	8	9	10	11	12	13
Ladder	Ishikawa EV D32N	Ishikawa NT5E D32N	Ishikawa EV S33F	Ishikawa NT5E S33F	Ishikawa EV S37C	Ishikawa NT5E S37C	Ishikawa EV S37F	Ishikawa NT5E S37F	Ishikawa EV S45F	Ishikawa NT5E S45F		Ladder

3D

Raw image
(gels are backwards)

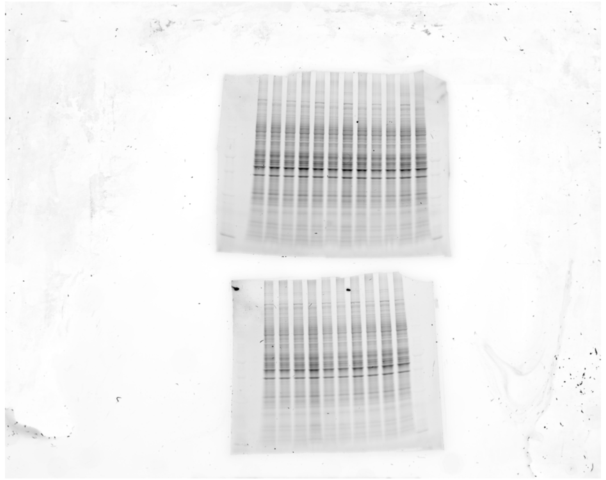

Flipped
horizontal
for figure

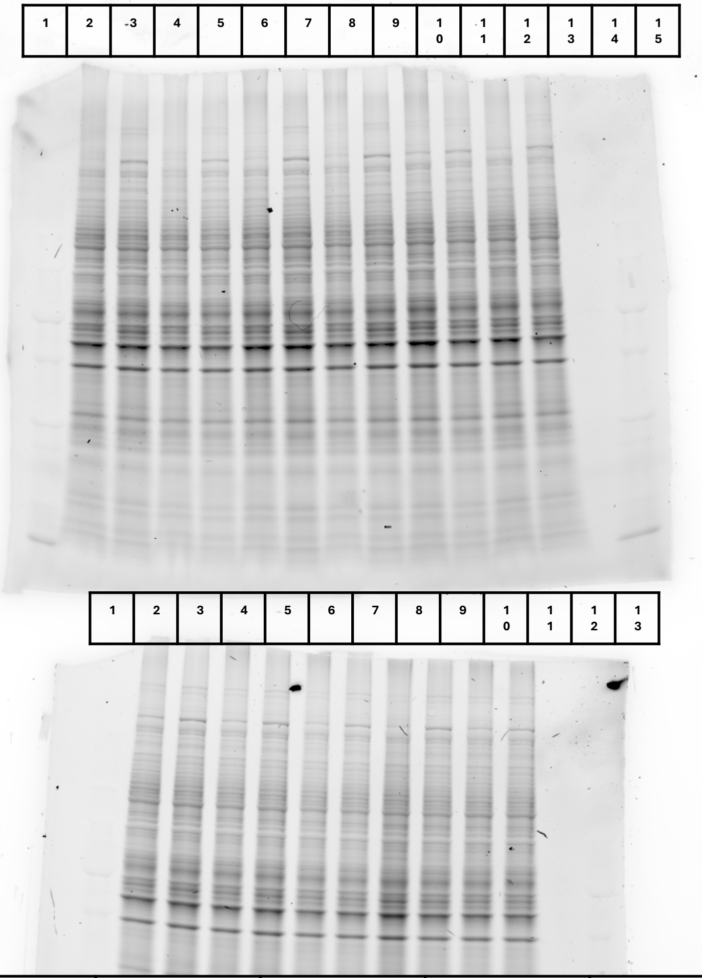

Total protein, 93 & 94

1	2	3	4	5	6	7	8	9	10	11	12	13	14	15
Ladder	Ishikawa EV No DNA	Ishikawa NT5E No DNA	Ishikawa EV WT	Ishikawa NT5E WT	Ishikawa EV D32N	Ishikawa NT5E D32N	Ishikawa EV S33F	Ishikawa NT5E S33F	Ishikawa EV S33Y	Ishikawa NT5E S33Y	Ishikawa EV G34R	Ishikawa NT5E G34R		Ladder

1	2	3	4	5	6	7	8	9	10	11	12	13
Ladder	Ishikawa EV D32N	Ishikawa NT5E D32N	Ishikawa EV S33F	Ishikawa NT5E S33F	Ishikawa EV S37C	Ishikawa NT5E S37C	Ishikawa EV S37F	Ishikawa NT5E S37F	Ishikawa EV S45F	Ishikawa NT5E S45F		Ladder

3D, CD73

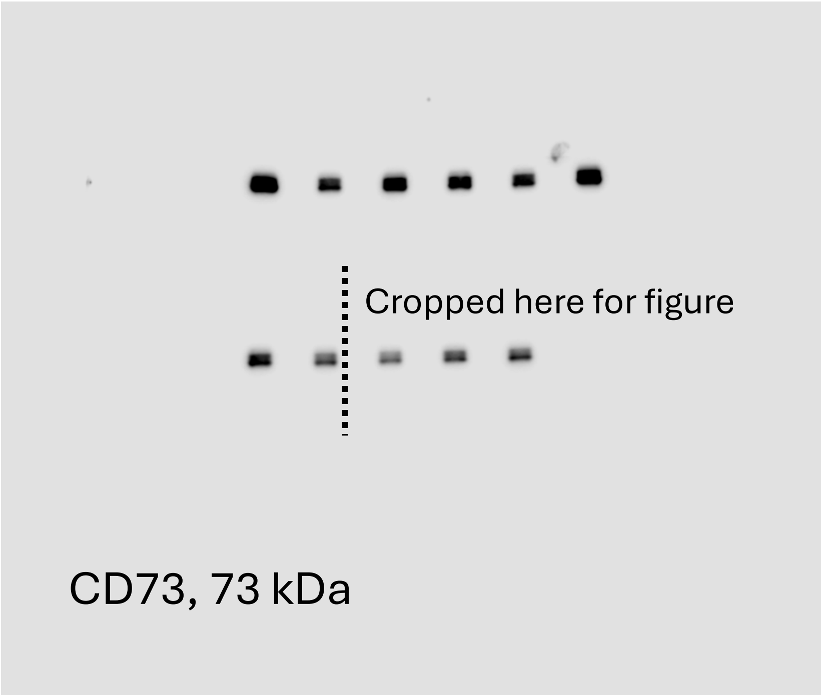

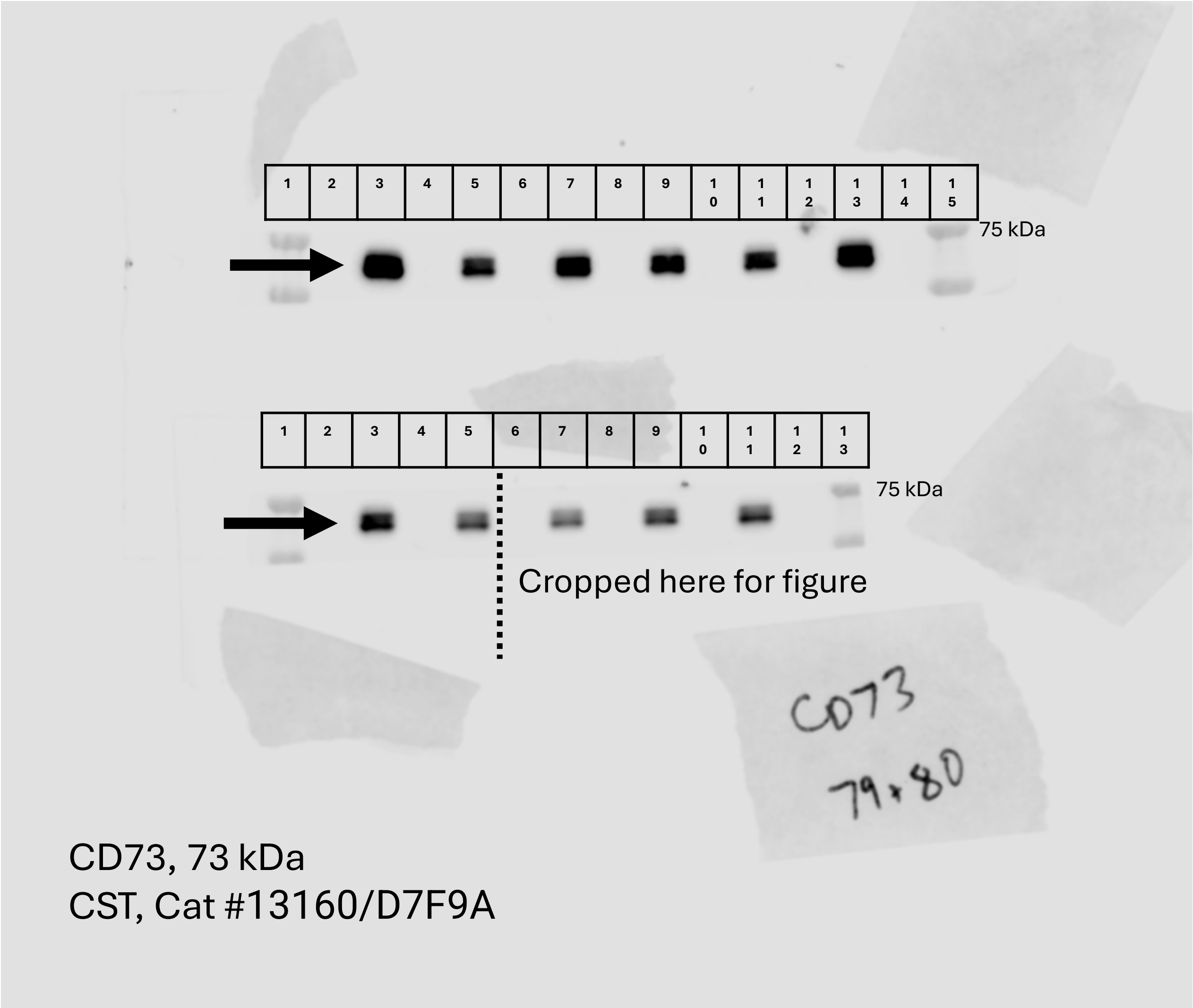

1	2	3	4	5	6	7	8	9	10	11	12	13	14	15
Ladder	Ishikawa EV No DNA	Ishikawa NT5E No DNA	Ishikawa EV WT	Ishikawa NT5E WT	Ishikawa EV D32N	Ishikawa NT5E D32N	Ishikawa EV S33F	Ishikawa NT5E S33F	Ishikawa EV S33Y	Ishikawa NT5E S33Y	Ishikawa EV G34R	Ishikawa NT5E G34R		Ladder

1	2	3	4	5	6	7	8	9	10	11	12	13
Ladder	Ishikawa EV WT	Ishikawa NT5E WT	Ishikawa EV D32N	Ishikawa NT5E D32N	Ishikawa EV S37C	Ishikawa NT5E S37C	Ishikawa EV S37F	Ishikawa NT5E S37F	Ishikawa EV S45F	Ishikawa NT5E S45F		Ladder

3D, Myc

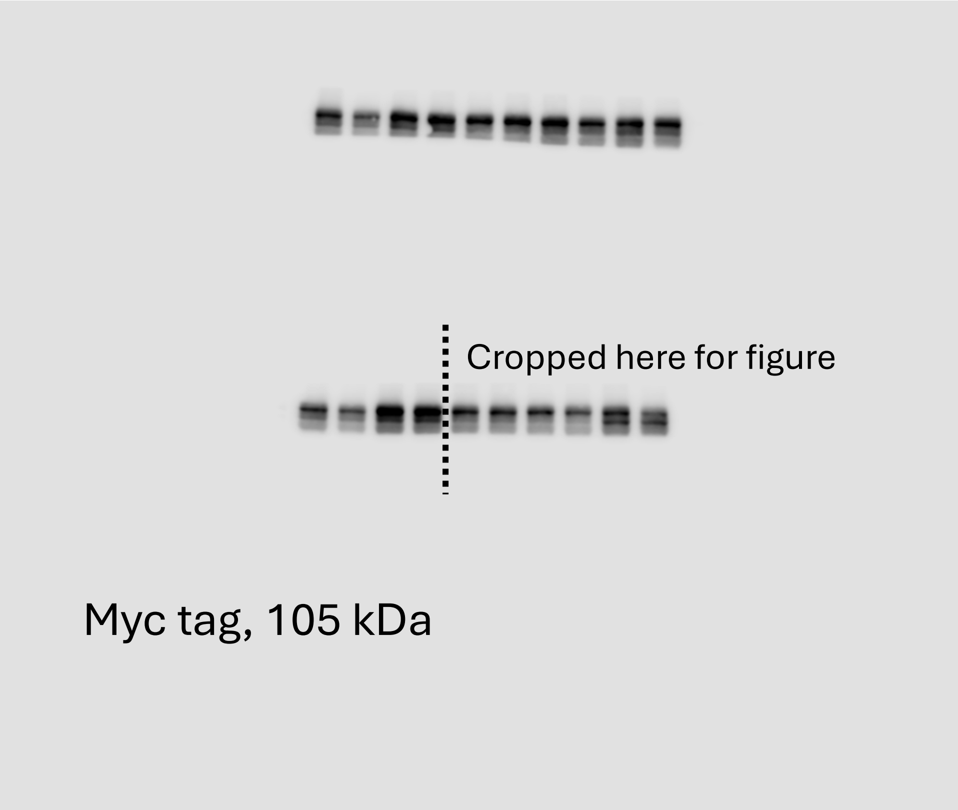

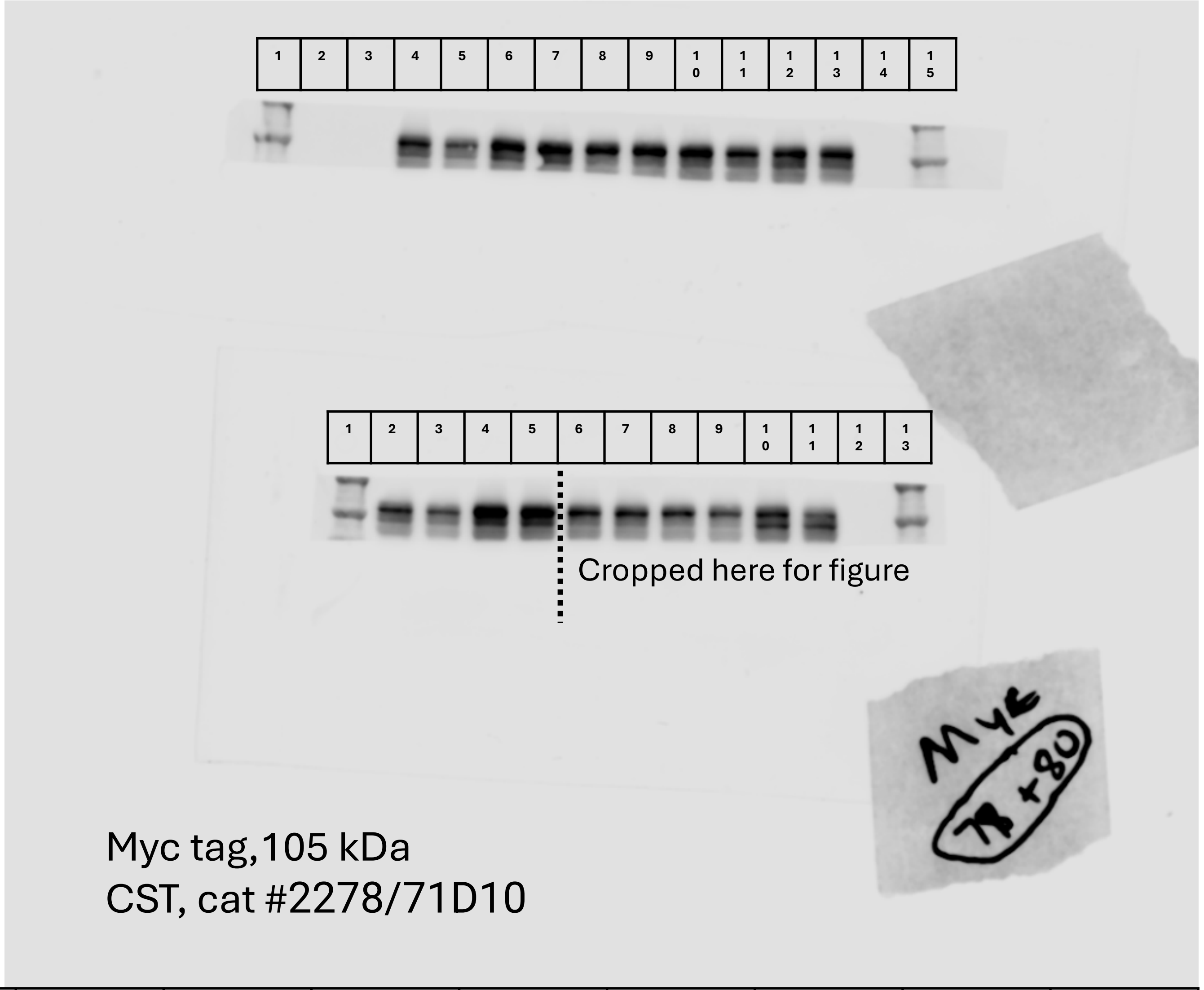

1	2	3	4	5	6	7	8	9	10	11	12	13	14	15
Ladder	Ishikawa EV No DNA	Ishikawa NT5E No DNA	Ishikawa EV WT	Ishikawa NT5E WT	Ishikawa EV D32N	Ishikawa NT5E D32N	Ishikawa EV S33F	Ishikawa NT5E S33F	Ishikawa EV S33Y	Ishikawa NT5E S33Y	Ishikawa EV G34R	Ishikawa NT5E G34R		Ladder

1	2	3	4	5	6	7	8	9	10	11	12	13
Ladder	Ishikawa EV WT	Ishikawa NT5E WT	Ishikawa EV D32N	Ishikawa NT5E D32N	Ishikawa EV S37C	Ishikawa NT5E S37C	Ishikawa EV S37F	Ishikawa NT5E S37F	Ishikawa EV S45F	Ishikawa NT5E S45F		Ladder

3D

Raw image
(gels are backwards)

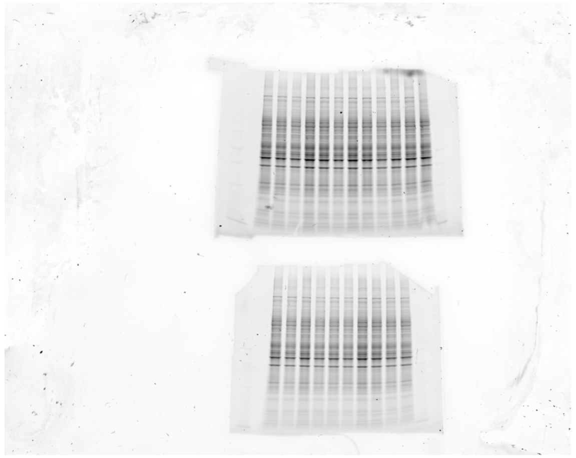

Flipped
horizontal
for figure

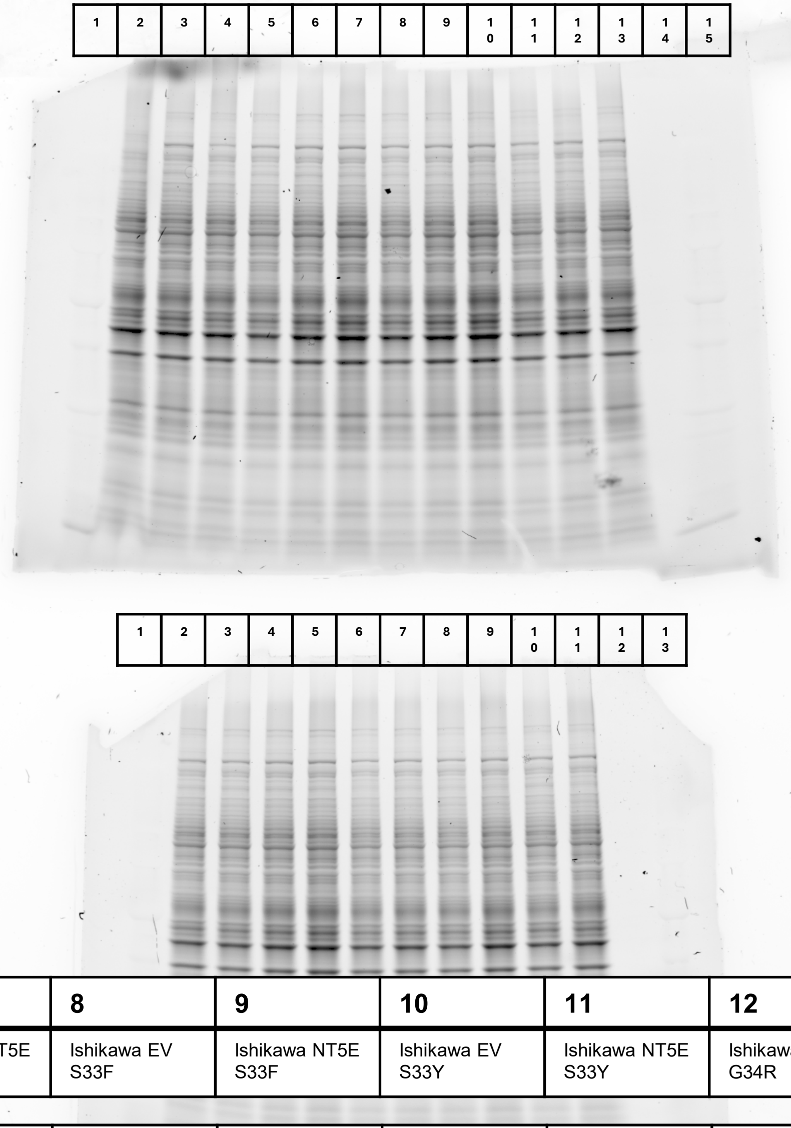

Total protein, 79 & 80

1	2	3	4	5	6	7	8	9	10	11	12	13	14	15
Ladder	Ishikawa EV No DNA	Ishikawa NT5E No DNA	Ishikawa EV WT	Ishikawa NT5E WT	Ishikawa EV D32N	Ishikawa NT5E D32N	Ishikawa EV S33F	Ishikawa NT5E S33F	Ishikawa EV S33Y	Ishikawa NT5E S33Y	Ishikawa EV G34R	Ishikawa NT5E G34R		Ladder

1	2	3	4	5	6	7	8	9	10	11	12	13
Ladder	Ishikawa EV WT	Ishikawa NT5E WT	Ishikawa EV D32N	Ishikawa NT5E D32N	Ishikawa EV S37C	Ishikawa NT5E S37C	Ishikawa EV S37F	Ishikawa NT5E S37F	Ishikawa EV S45F	Ishikawa NT5E S45F		Ladder

Figure 4
WB Slides

4D images

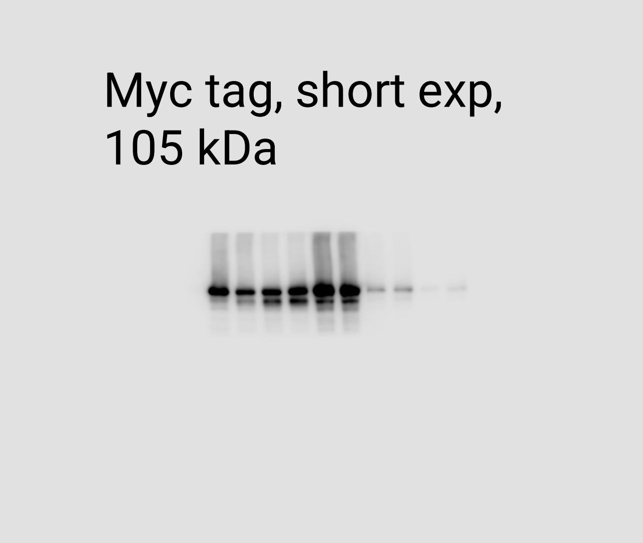

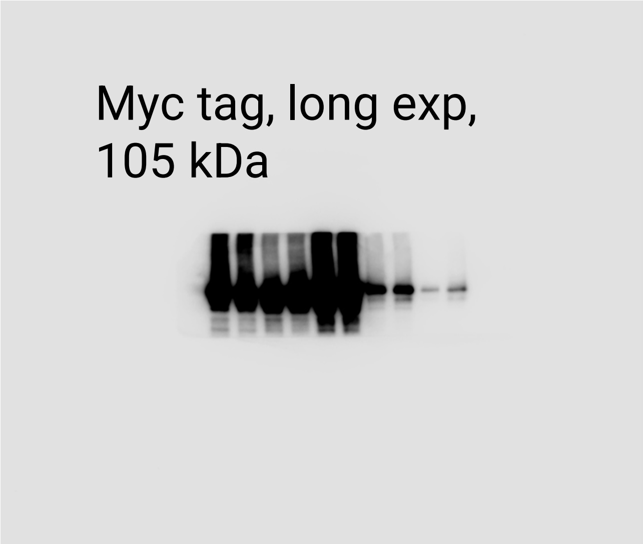

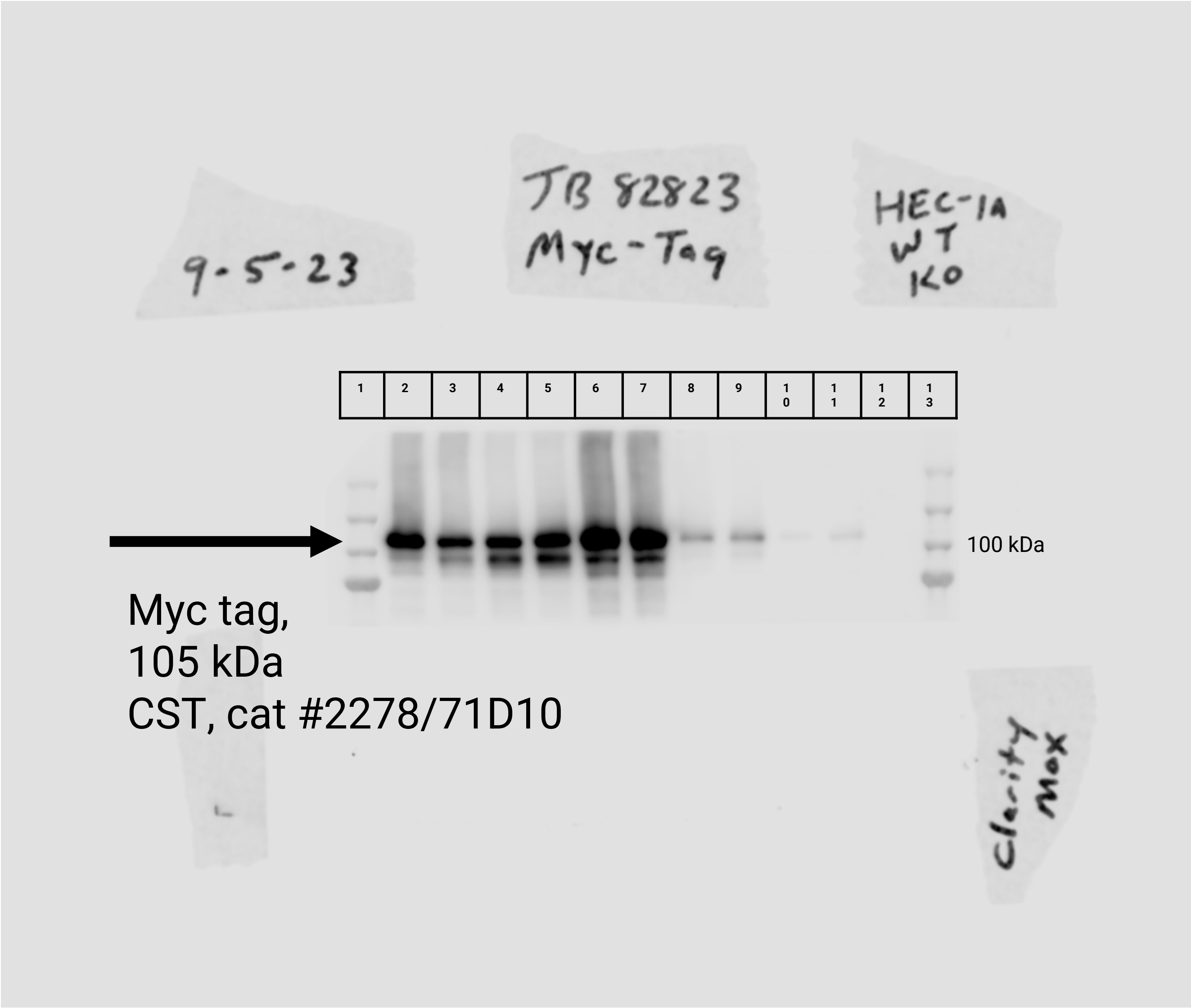

1	2	3	4	5	6	7	8	9	10	11	12	13
Ladder	NT5E $^{+/+}$ WCL	NT5E $^{-/-}$ WCL	NT5E $^{+/+}$ Cyto	NT5E $^{-/-}$ Cyto	NT5E $^{+/+}$ Mem	NT5E $^{-/-}$ Mem	NT5E $^{+/+}$ Nuc	NT5E $^{-/-}$ Nuc	NT5E $^{+/+}$ Chro	NT5E $^{-/-}$ Chro		Ladder

4D images

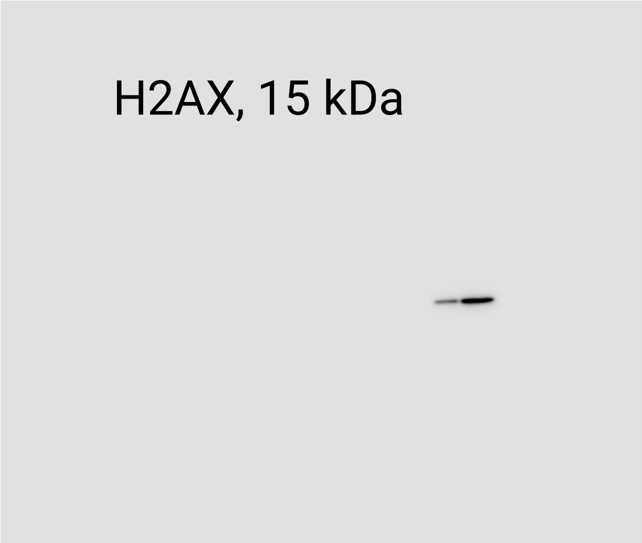

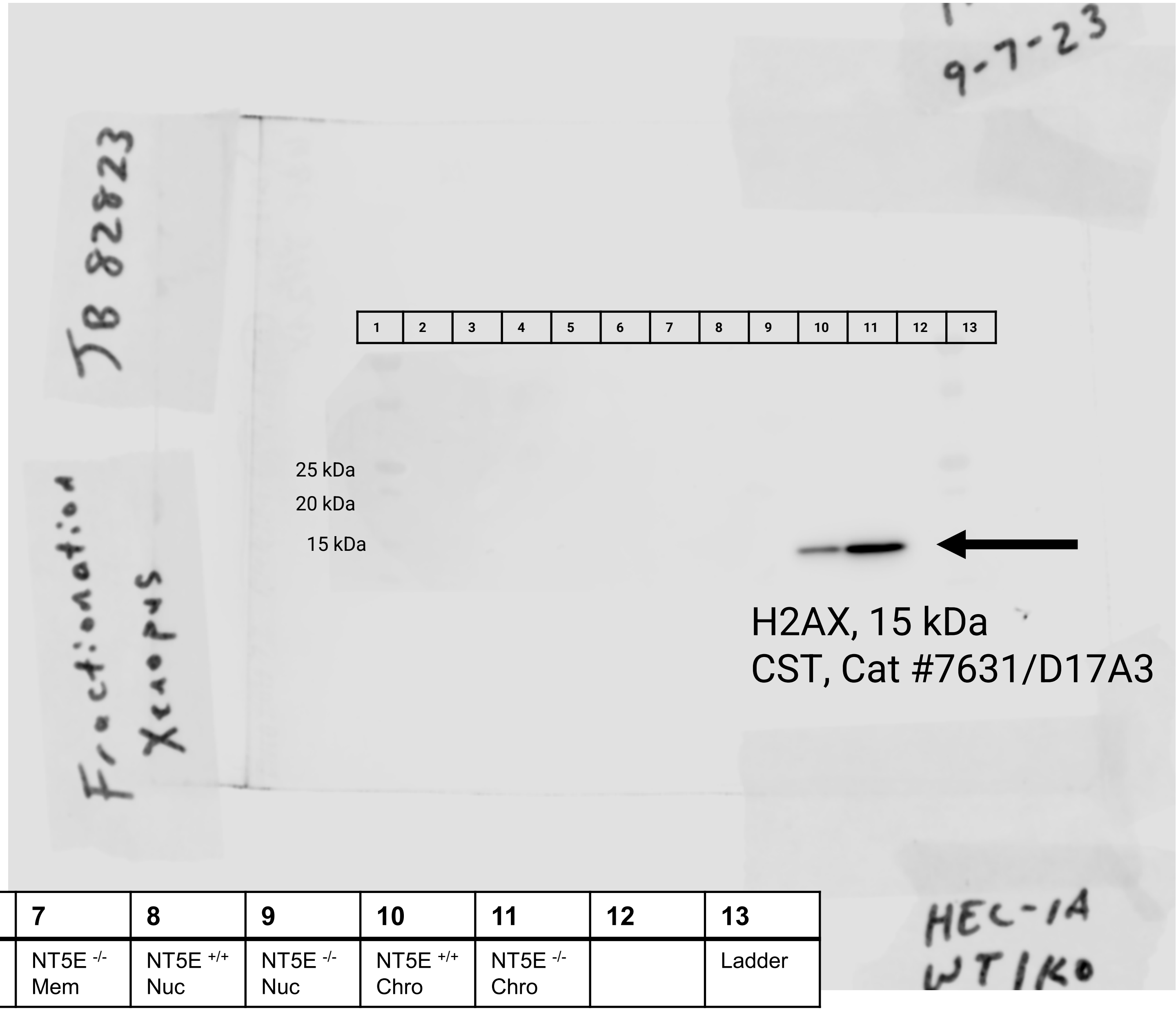

1	2	3	4	5	6	7	8	9	10	11	12	13
Ladder	NT5E $^{+/+}$ WCL	NT5E $^{-/-}$ WCL	NT5E $^{+/+}$ Cyto	NT5E $^{-/-}$ Cyto	NT5E $^{+/+}$ Mem	NT5E $^{-/-}$ Mem	NT5E $^{+/+}$ Nuc	NT5E $^{-/-}$ Nuc	NT5E $^{+/+}$ Chro	NT5E $^{-/-}$ Chro		Ladder

4D images

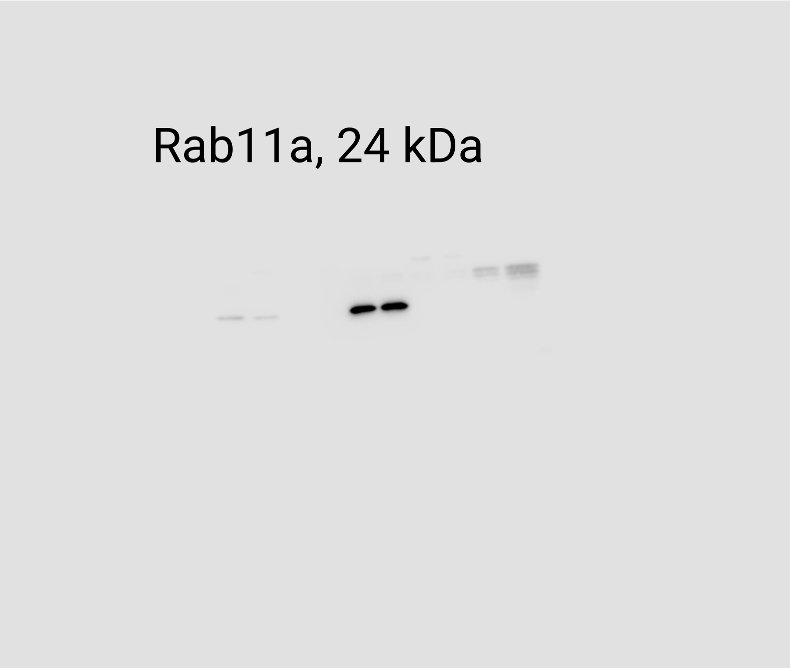

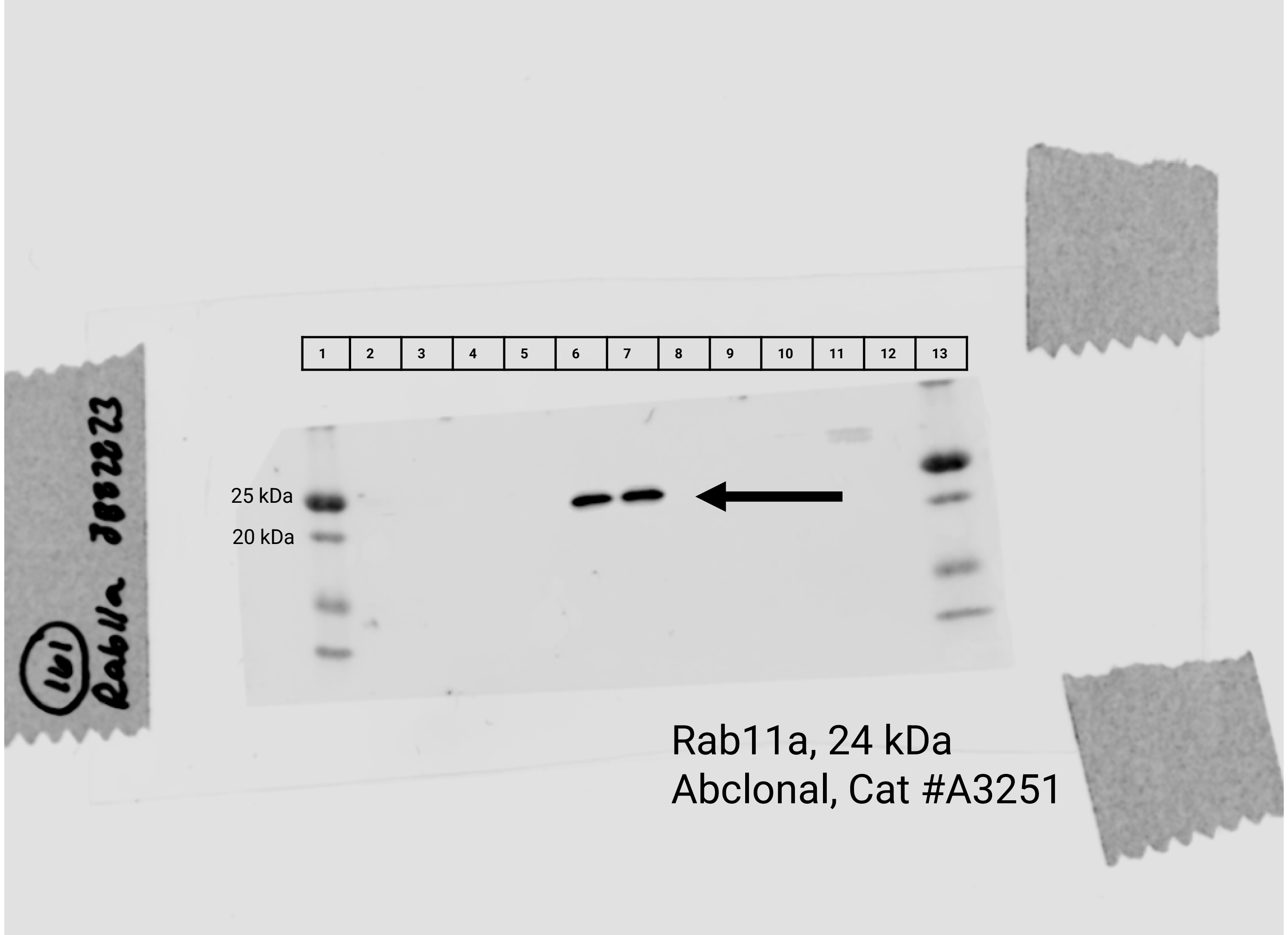

1	2	3	4	5	6	7	8	9	10	11	12	13
Ladder	NT5E $^{+/+}$ WCL	NT5E $^{-/-}$ WCL	NT5E $^{+/+}$ Cyto	NT5E $^{-/-}$ Cyto	NT5E $^{+/+}$ Mem	NT5E $^{-/-}$ Mem	NT5E $^{+/+}$ Nuc	NT5E $^{-/-}$ Nuc	NT5E $^{+/+}$ Chro	NT5E $^{-/-}$ Chro		Ladder

4D images

SP1, 90 kDa

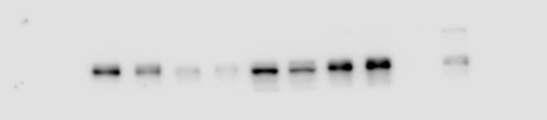

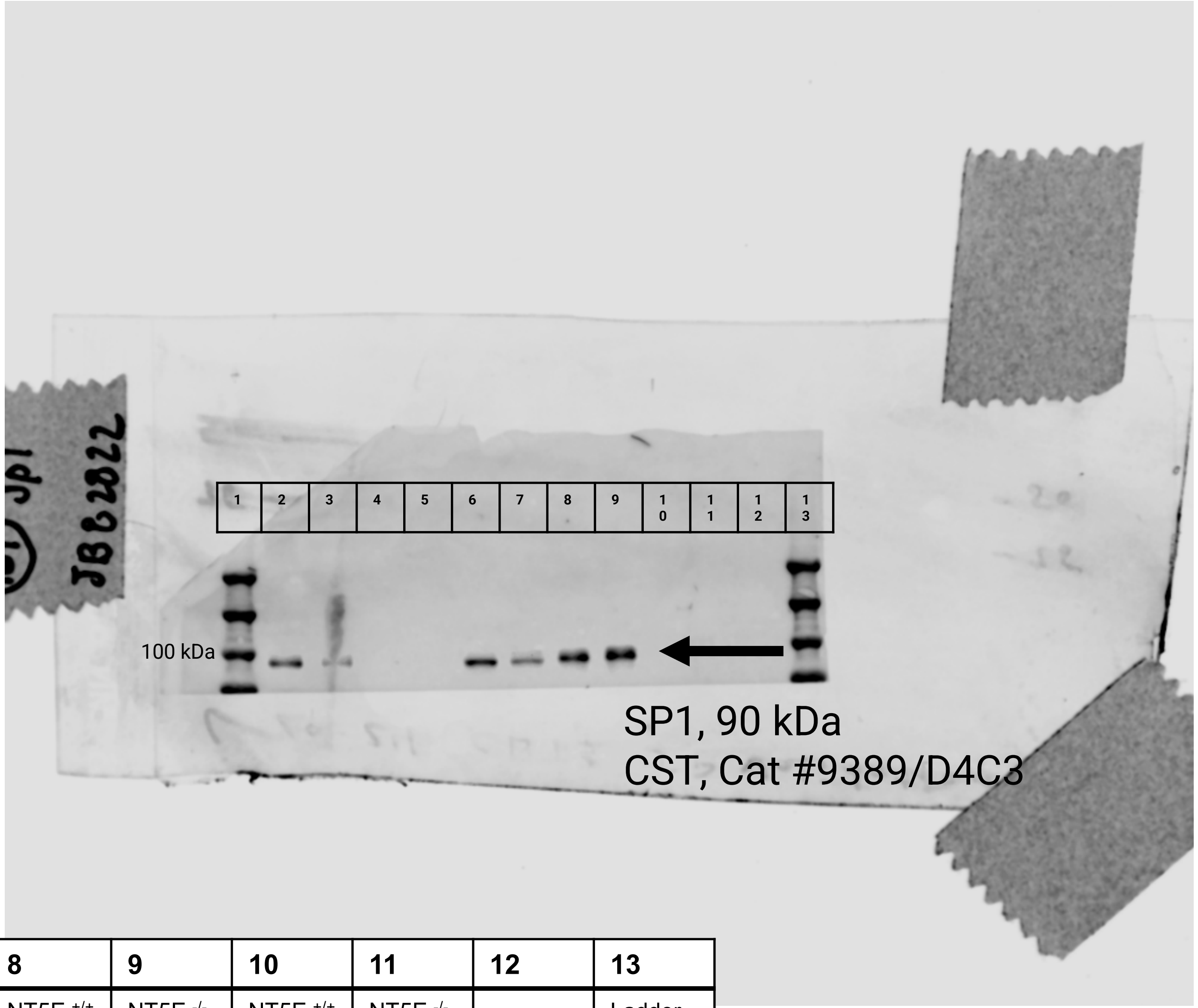

1	2	3	4	5	6	7	8	9	10	11	12	13
Ladder	NT5E $^{+/+}$ WCL	NT5E $^{-/-}$ WCL	NT5E $^{+/+}$ Cyto	NT5E $^{-/-}$ Cyto	NT5E $^{+/+}$ Mem	NT5E $^{-/-}$ Mem	NT5E $^{+/+}$ Nuc	NT5E $^{-/-}$ Nuc	NT5E $^{+/+}$ Chro	NT5E $^{-/-}$ Chro		Ladder

4D images

Total protein:
10% 2,2,2-trichloroethanol

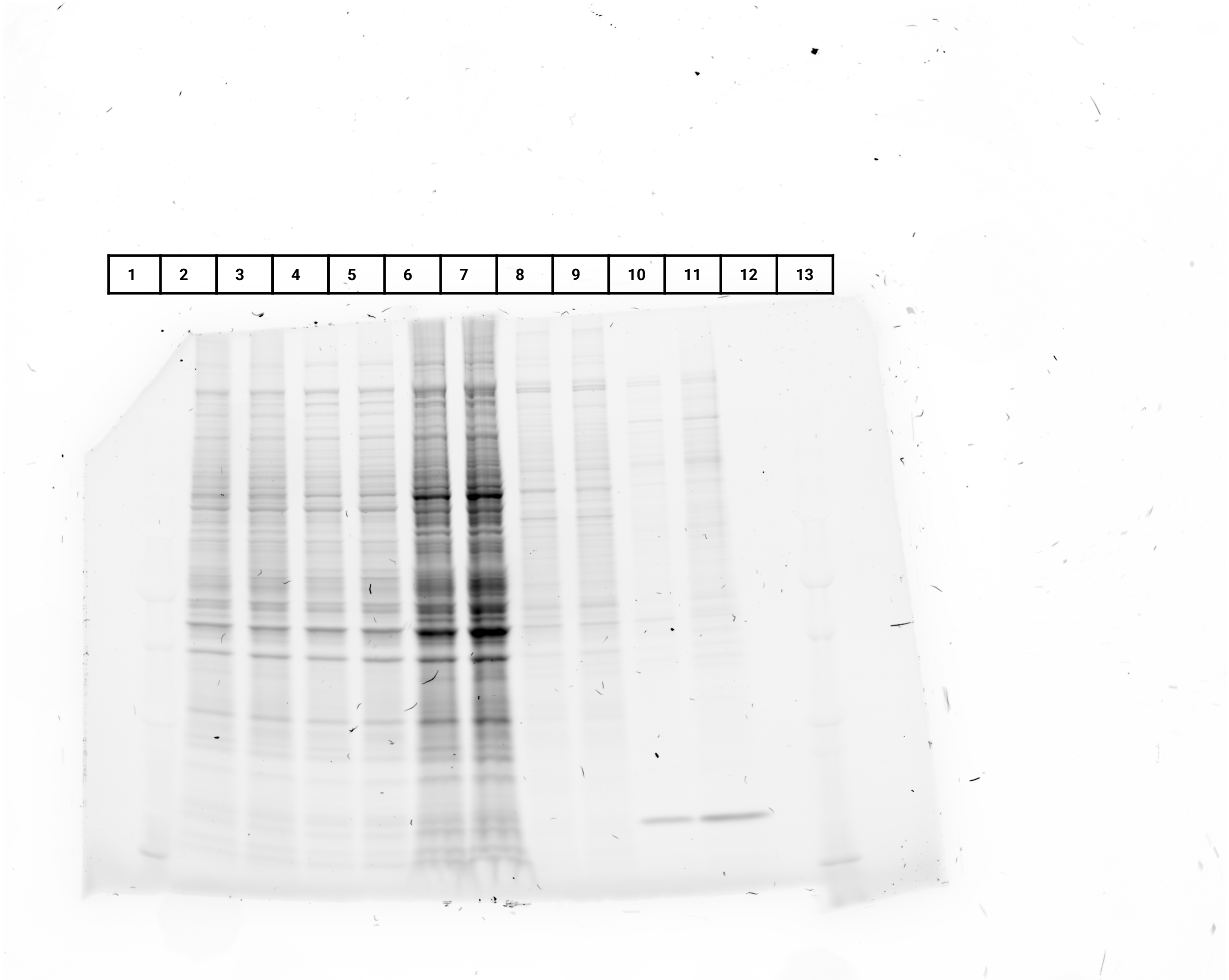

1	2	3	4	5	6	7	8	9	10	11	12	13
Ladder	NT5E $^{+/+}$ WCL	NT5E $^{-/-}$ WCL	NT5E $^{+/+}$ Cyto	NT5E $^{-/-}$ Cyto	NT5E $^{+/+}$ Mem	NT5E $^{-/-}$ Mem	NT5E $^{+/+}$ Nuc	NT5E $^{-/-}$ Nuc	NT5E $^{+/+}$ Chro	NT5E $^{-/-}$ Chro		Ladder

4E images - Myc

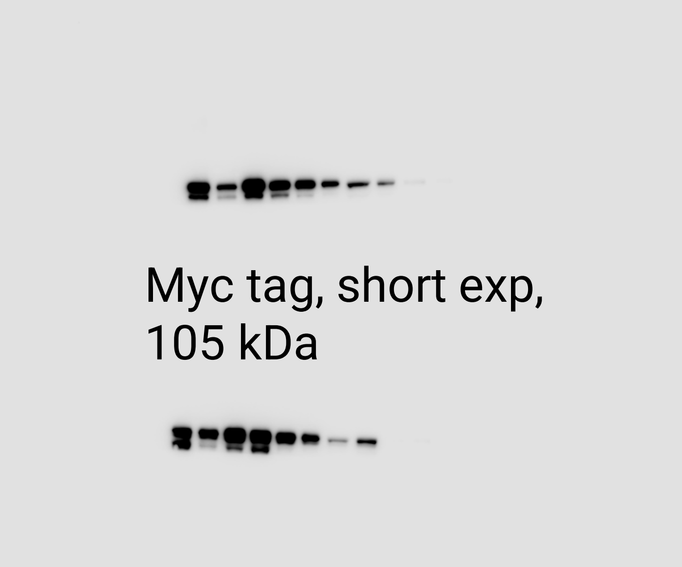

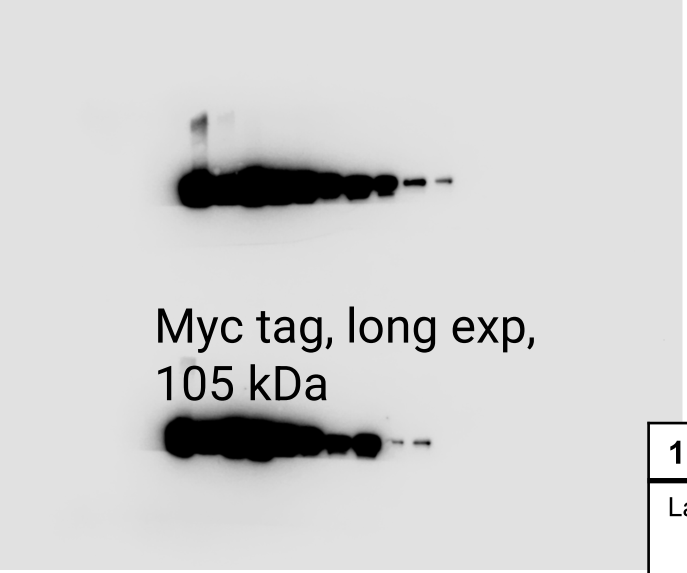

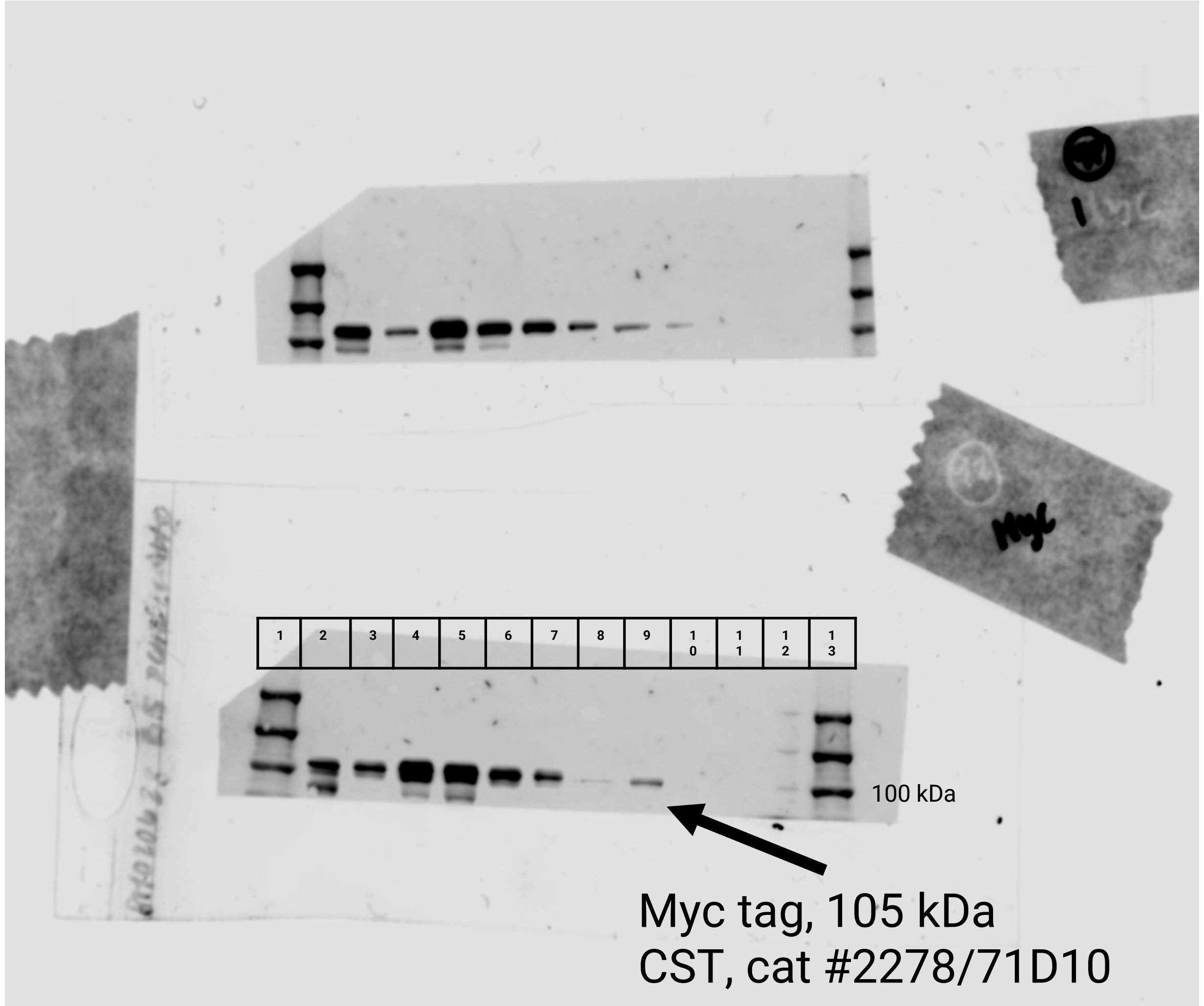

1	2	3		4	5	6	7	8	9	10	11	12	13
Ladder	NT5E $^{+/+}$ WCL	NT5E $^{-/-}$ WCL		NT5E $^{+/+}$ Cyto	NT5E $^{-/-}$ Cyto	NT5E $^{+/+}$ Mem	NT5E $^{-/-}$ Mem	NT5E $^{+/+}$ Nuc	NT5E $^{-/-}$ Nuc	NT5E $^{+/+}$ Chro	NT5E $^{-/-}$ Chro		Ladder

4E images – H2AX

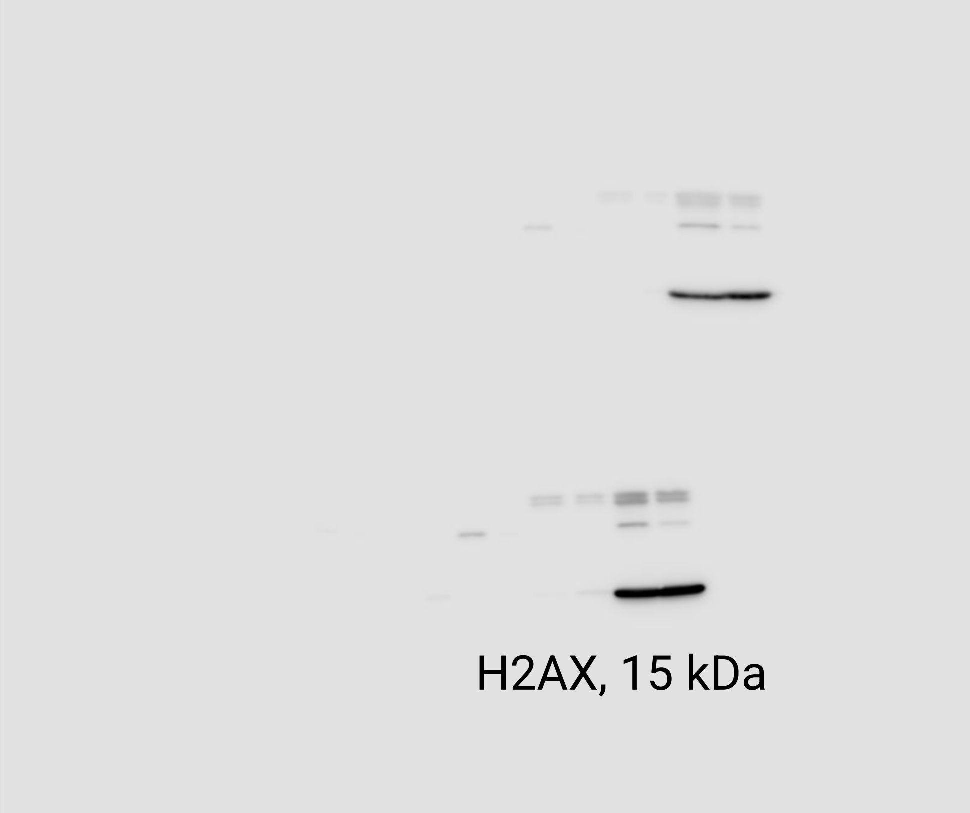

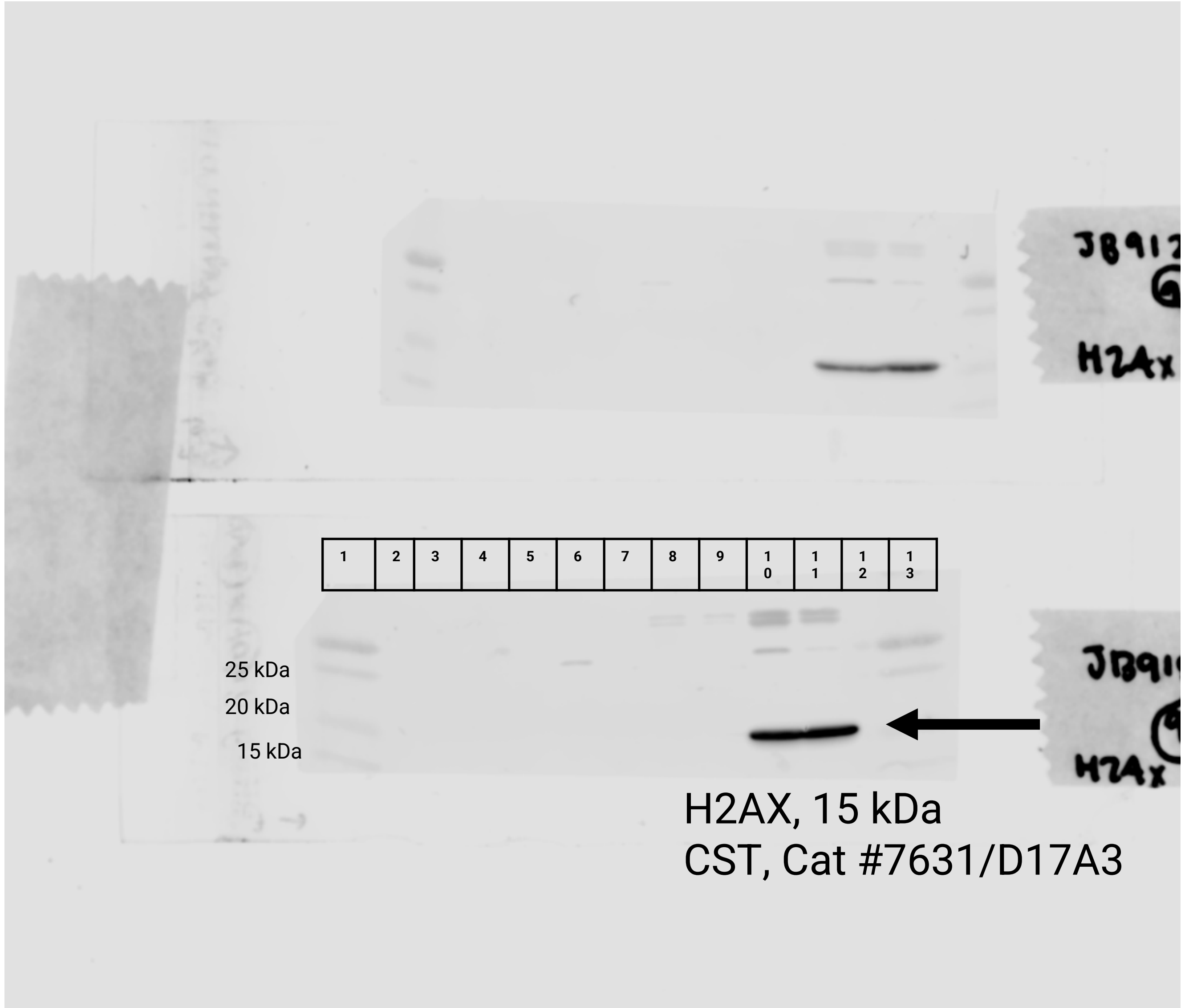

1	2	3	4	5	6	7	8	9	10	11	12	13
Ladder	NT5E $^{+/+}$ WCL	NT5E $^{-/-}$ WCL	NT5E $^{+/+}$ Cyto	NT5E $^{-/-}$ Cyto	NT5E $^{+/+}$ Mem	NT5E $^{-/-}$ Mem	NT5E $^{+/+}$ Nuc	NT5E $^{-/-}$ Nuc	NT5E $^{+/+}$ Chro	NT5E $^{-/-}$ Chro		Ladder

4E images – Rab11A

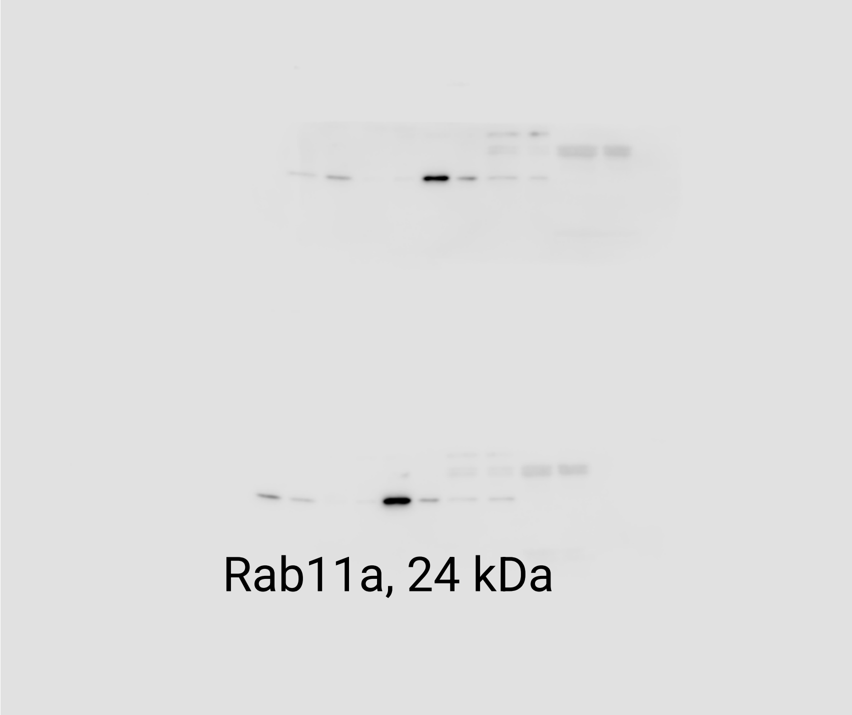

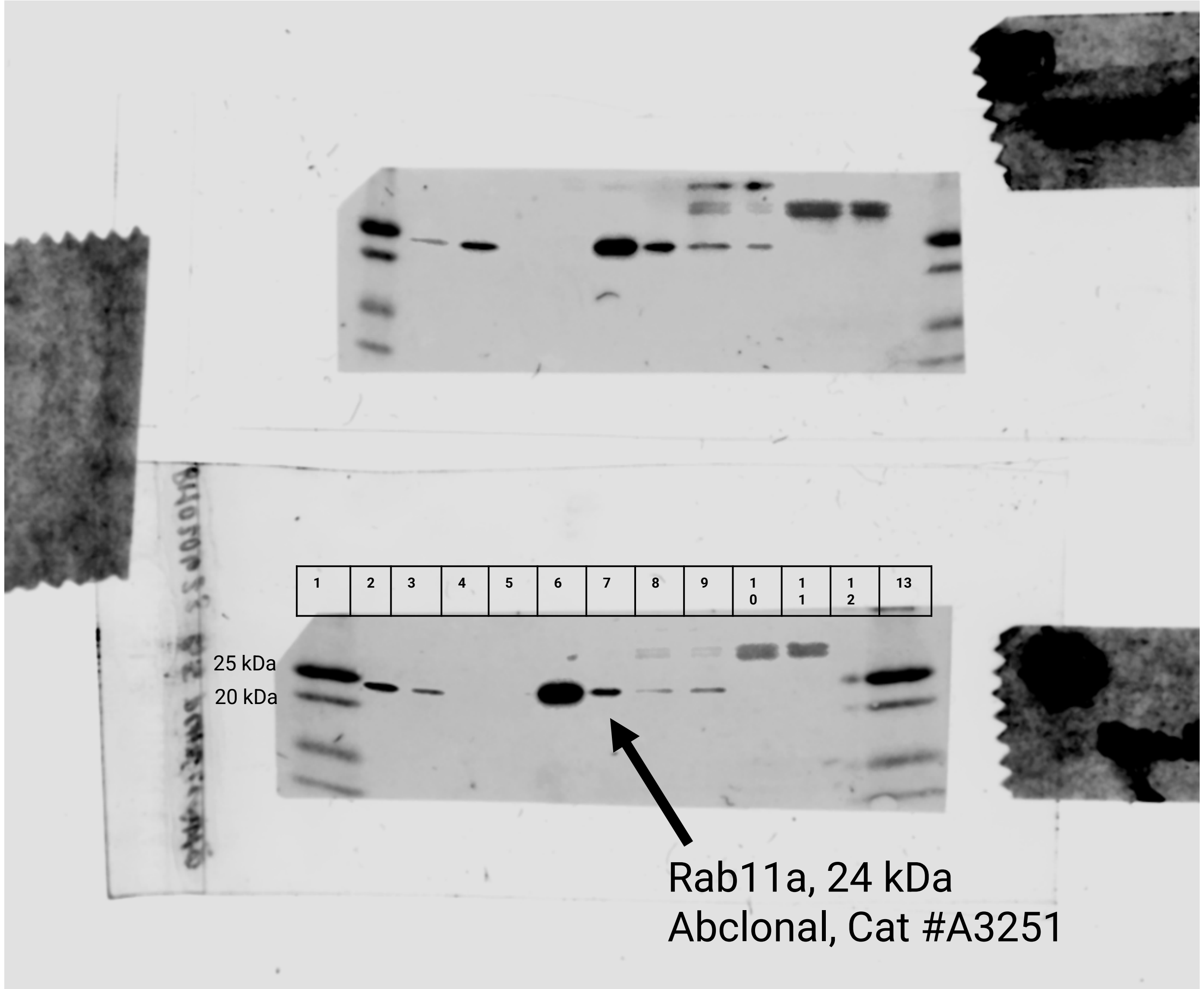

1	2	3	4	5	6	7	8	9	10	11	12	13
Ladder	NT5E $^{+/+}$ WCL	NT5E $^{-/-}$ WCL	NT5E $^{+/+}$ Cyto	NT5E $^{-/-}$ Cyto	NT5E $^{+/+}$ Mem	NT5E $^{-/-}$ Mem	NT5E $^{+/+}$ Nuc	NT5E $^{-/-}$ Nuc	NT5E $^{+/+}$ Chro	NT5E $^{-/-}$ Chro		Ladder

4E images – SP1

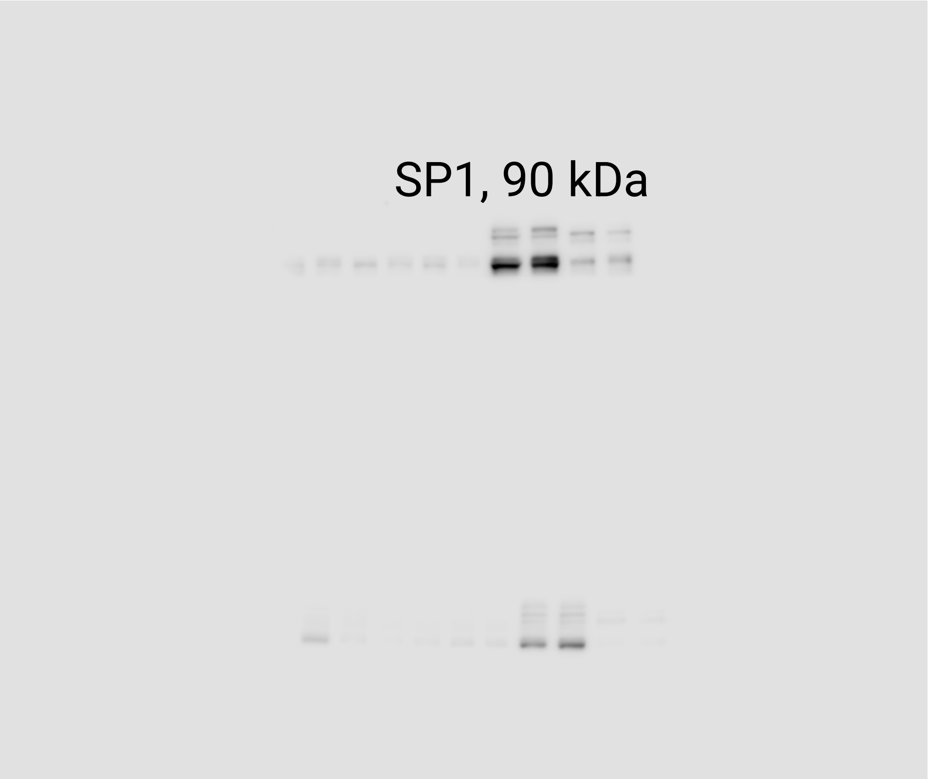

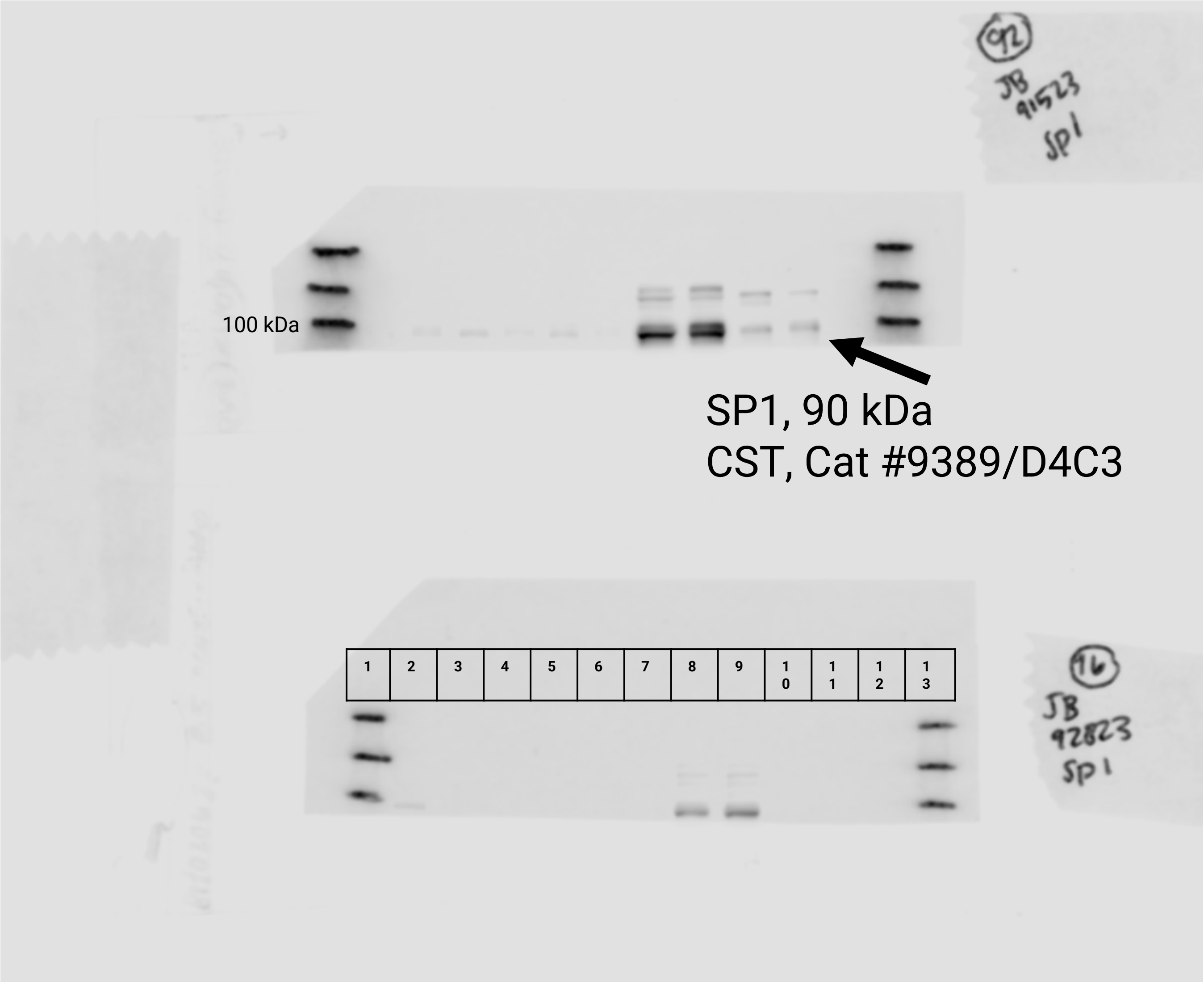

1	2	3	4	5	6	7	8	9	10	11	12	13
Ladder	NT5E $^{+/+}$ WCL	NT5E $^{-/-}$ WCL	NT5E $^{+/+}$ Cyto	NT5E $^{-/-}$ Cyto	NT5E $^{+/+}$ Mem	NT5E $^{-/-}$ Mem	NT5E $^{+/+}$ Nuc	NT5E $^{-/-}$ Nuc	NT5E $^{+/+}$ Chro	NT5E $^{-/-}$ Chro		Ladder

4E images

Total protein:
10% 2,2,2-trichloroethanol

Invitrogen gel 4-20%

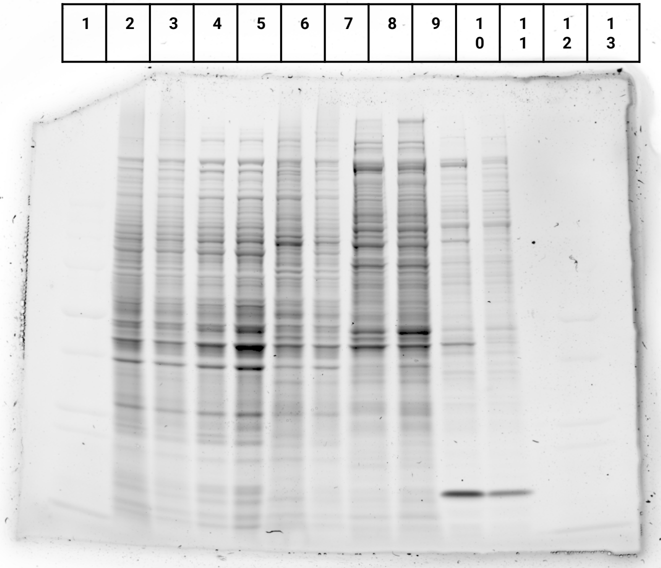

1	2	3	4	5	6	7	8	9	10	11	12	13
Ladder	NT5E $^{+/+}$ WCL	NT5E $^{-/-}$ WCL	NT5E $^{+/+}$ Cyto	NT5E $^{-/-}$ Cyto	NT5E $^{+/+}$ Mem	NT5E $^{-/-}$ Mem	NT5E $^{+/+}$ Nuc	NT5E $^{-/-}$ Nuc	NT5E $^{+/+}$ Chro	NT5E $^{-/-}$ Chro		Ladder

4F Images - Myc

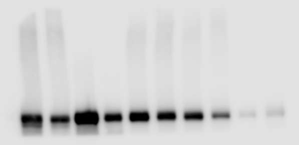

Myc tag, short exp, 105 kDa

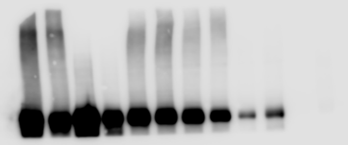

Myc tag, long exp, 105 kDa

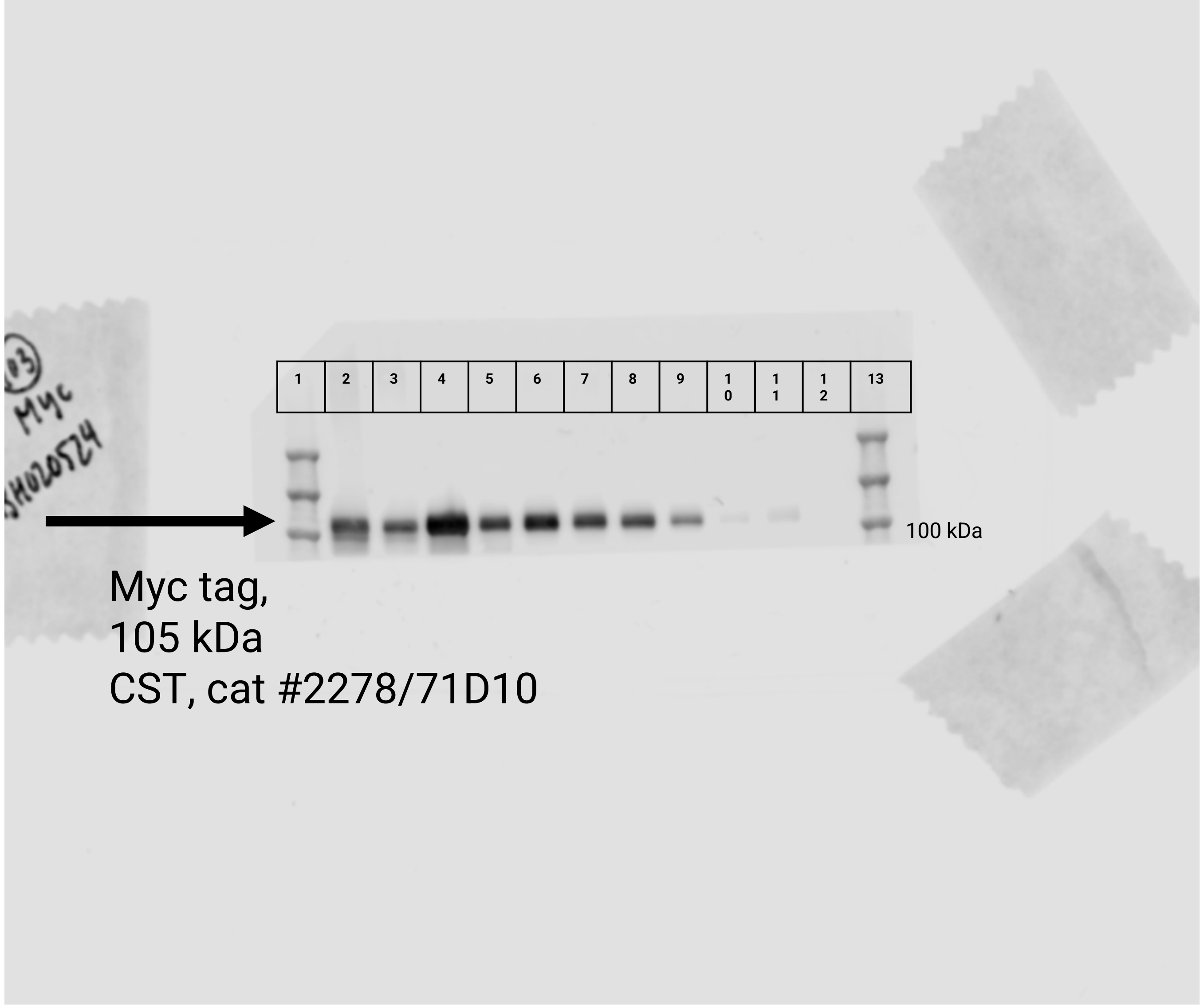

1	2	3	4	5	6	7	8	9	10	11	12	13
Ladder	NT5E $^{+/+}$ WCL	NT5E $^{-/-}$ WCL	NT5E $^{+/+}$ Cyto	NT5E $^{-/-}$ Cyto	NT5E $^{+/+}$ Mem	NT5E $^{-/-}$ Mem	NT5E $^{+/+}$ Nuc	NT5E $^{-/-}$ Nuc	NT5E $^{+/+}$ Chro	NT5E $^{-/-}$ Chro		Ladder

4F Images - Rab11a

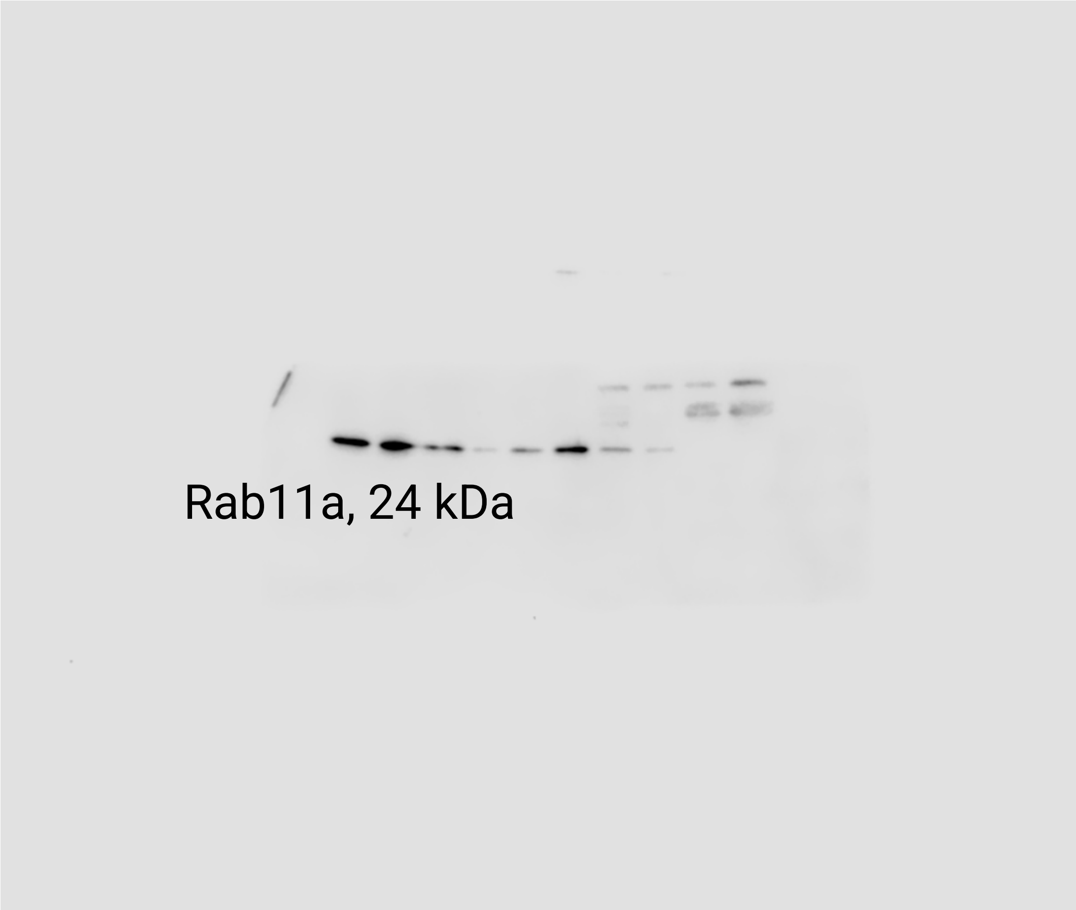

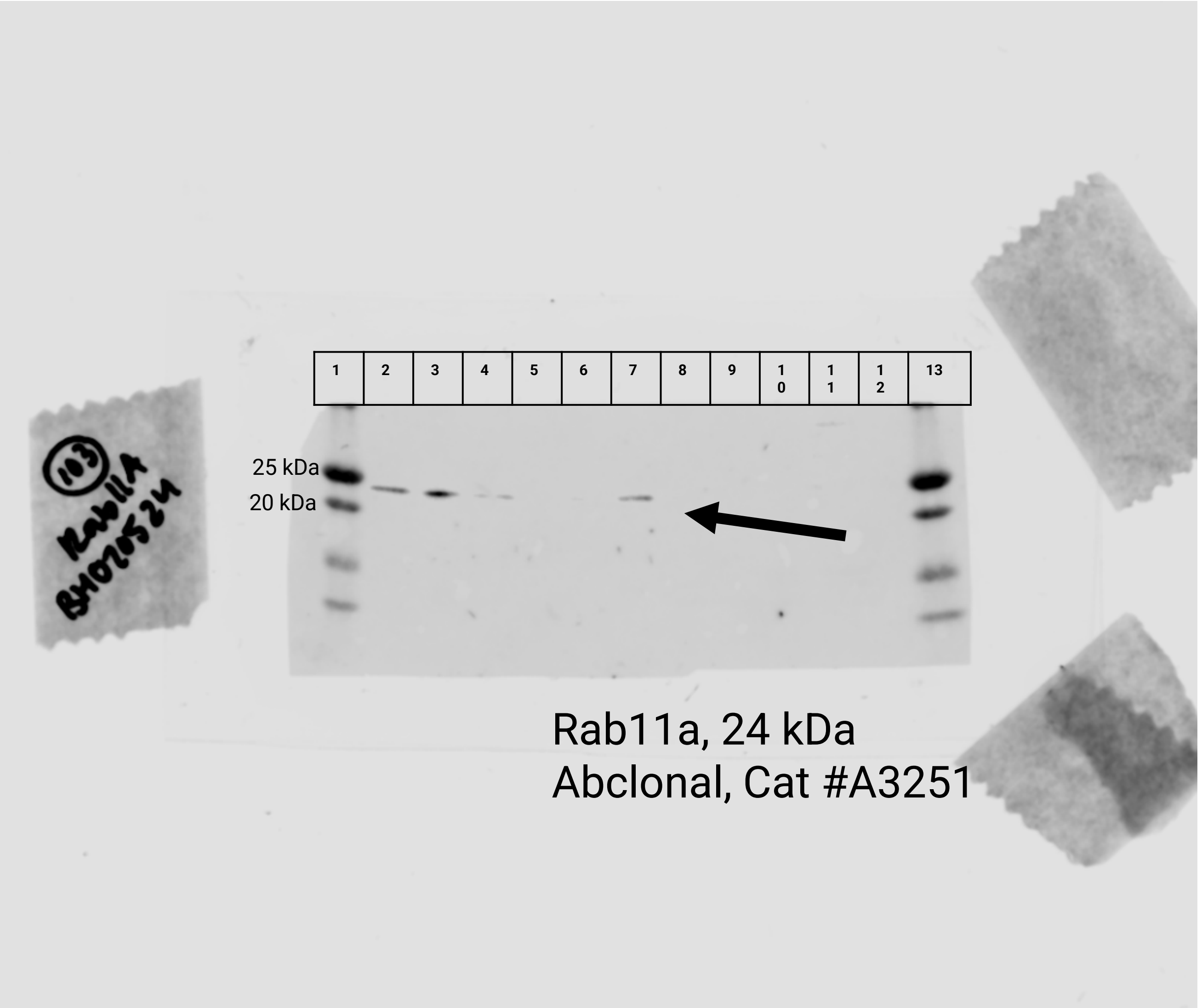

1	2	3	4	5	6	7	8	9	10	11	12	13
Ladder	NT5E $^{+/+}$ WCL	NT5E $^{-/-}$ WCL	NT5E $^{+/+}$ Cyto	NT5E $^{-/-}$ Cyto	NT5E $^{+/+}$ Mem	NT5E $^{-/-}$ Mem	NT5E $^{+/+}$ Nuc	NT5E $^{-/-}$ Nuc	NT5E $^{+/+}$ Chro	NT5E $^{-/-}$ Chro		Ladder

4F Images - SP1

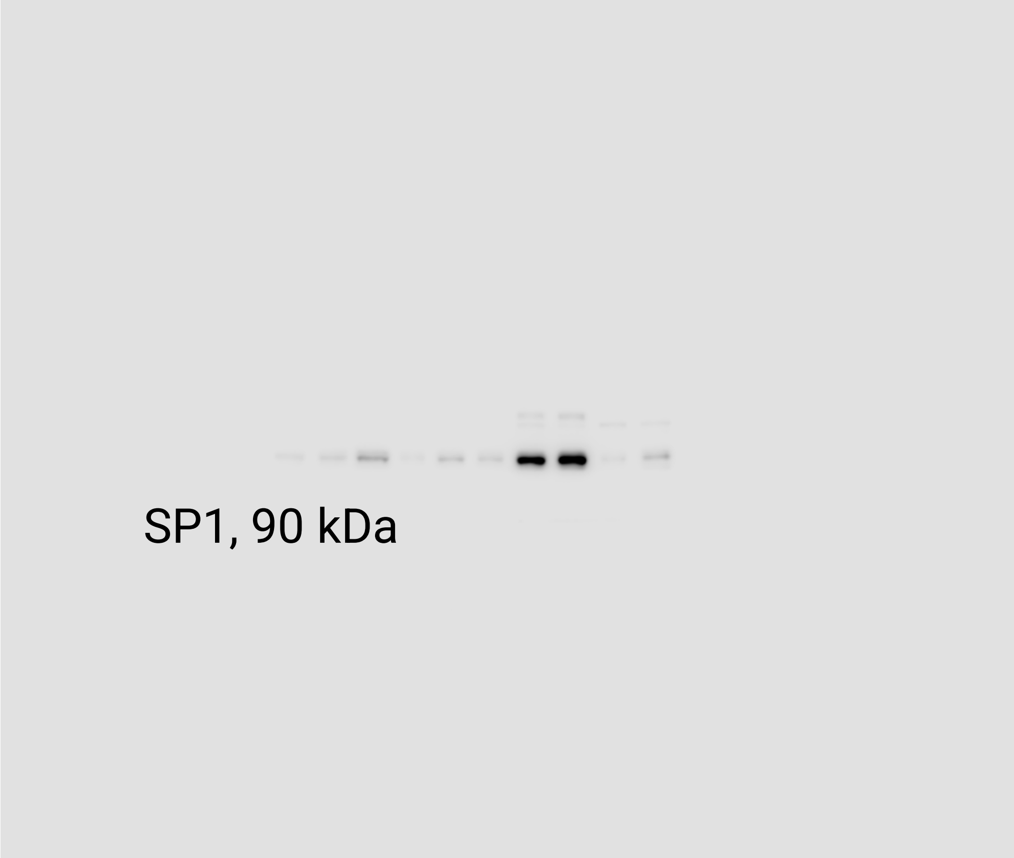

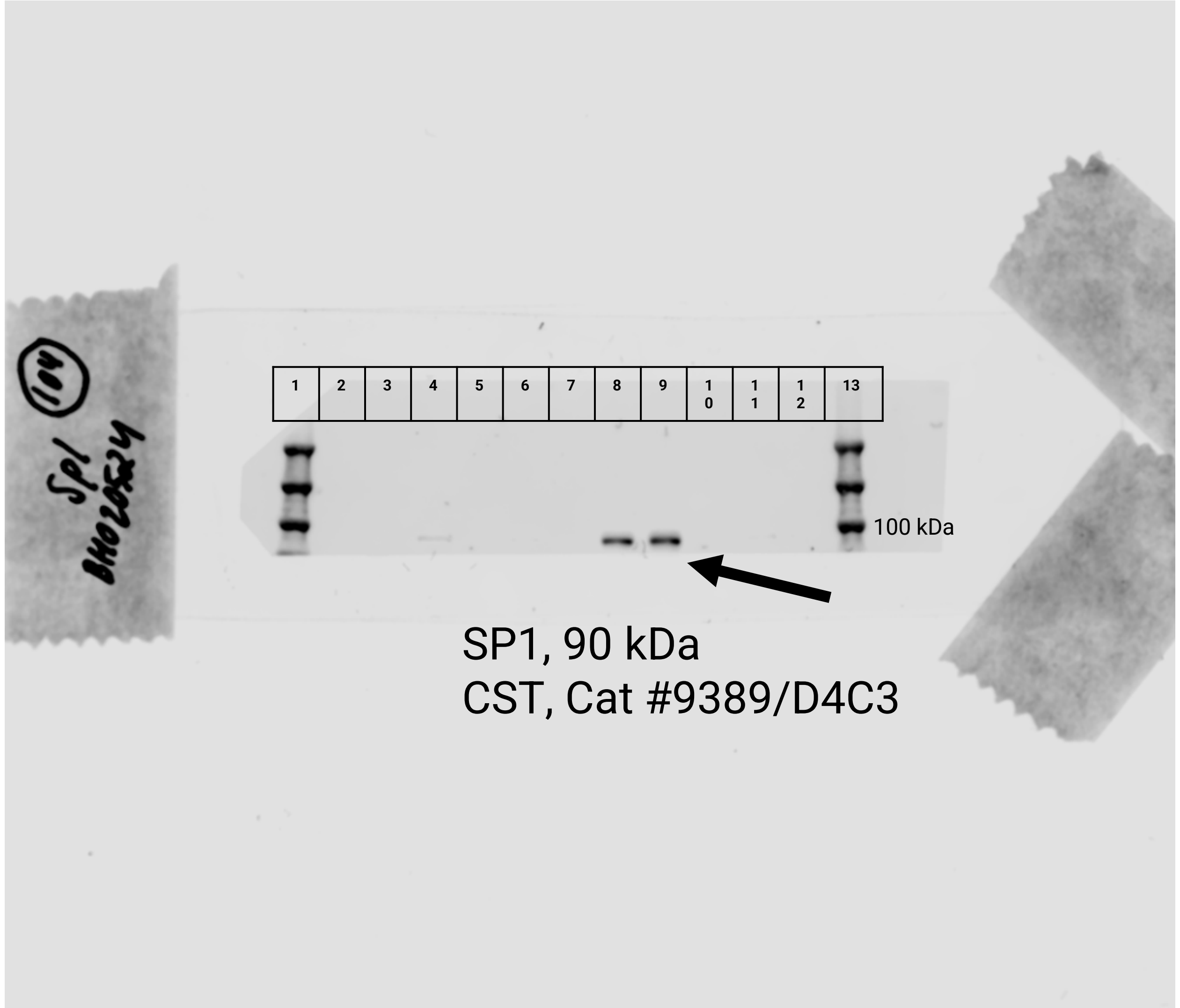

1	2	3	4	5	6	7	8	9	10	11	12	13
Ladder	NT5E $^{+/+}$ WCL	NT5E $^{-/-}$ WCL	NT5E $^{+/+}$ Cyto	NT5E $^{-/-}$ Cyto	NT5E $^{+/+}$ Mem	NT5E $^{-/-}$ Mem	NT5E $^{+/+}$ Nuc	NT5E $^{-/-}$ Nuc	NT5E $^{+/+}$ Chro	NT5E $^{-/-}$ Chro		Ladder

4F Images - H2AX

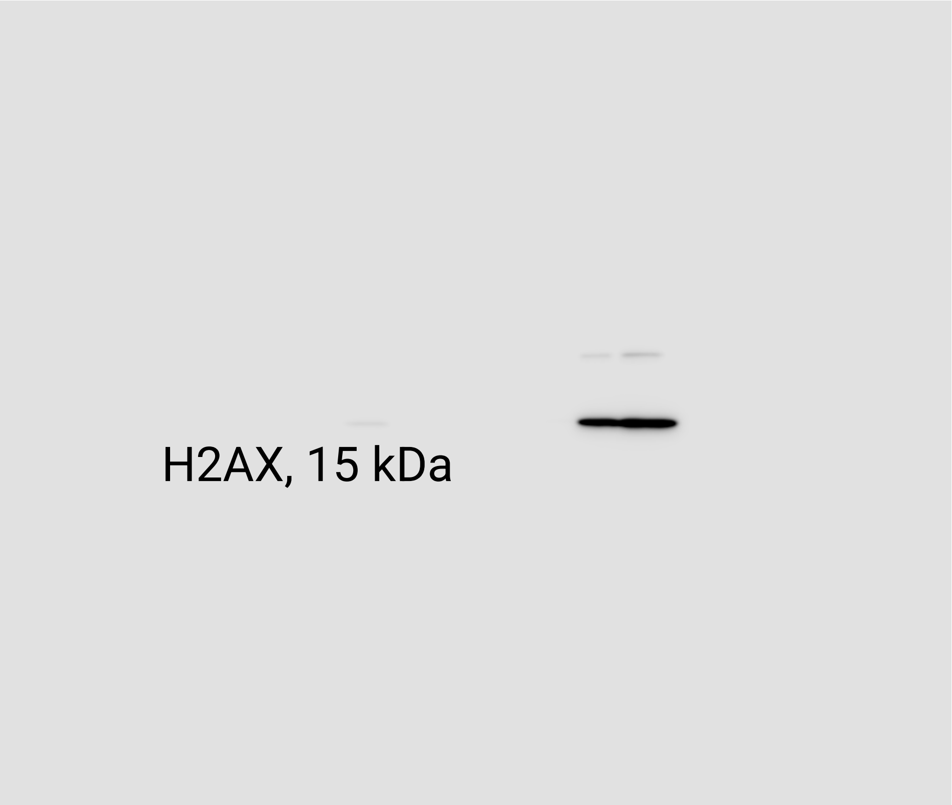

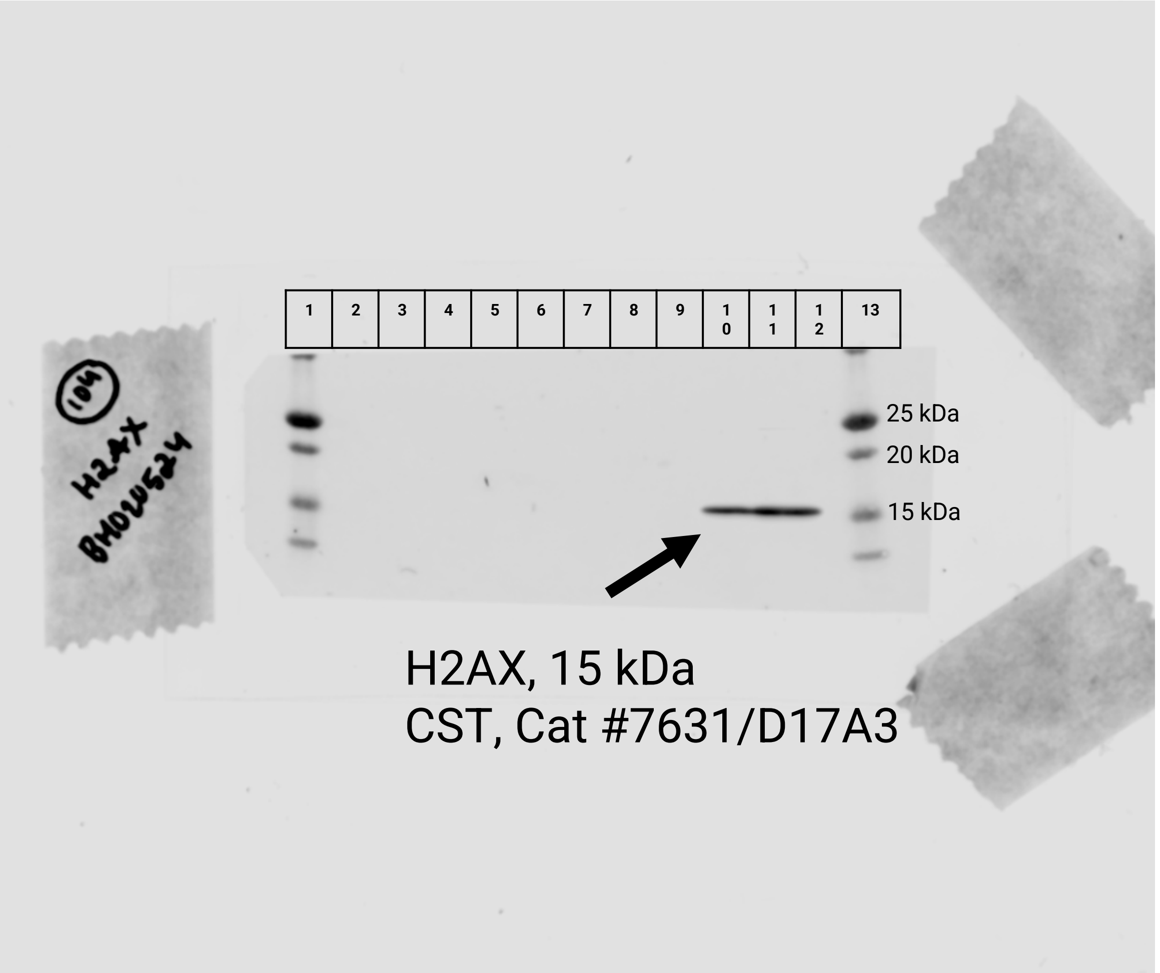

1	2	3	4	5	6	7	8	9	10	11	12	13
Ladder	NT5E $^{+/+}$ WCL	NT5E $^{-/-}$ WCL	NT5E $^{+/+}$ Cyto	NT5E $^{-/-}$ Cyto	NT5E $^{+/+}$ Mem	NT5E $^{-/-}$ Mem	NT5E $^{+/+}$ Nuc	NT5E $^{-/-}$ Nuc	NT5E $^{+/+}$ Chro	NT5E $^{-/-}$ Chro		Ladder

4F Images

Total protein:
10% 2,2,2-trichloroethanol

Invitrogen gel 4-20%

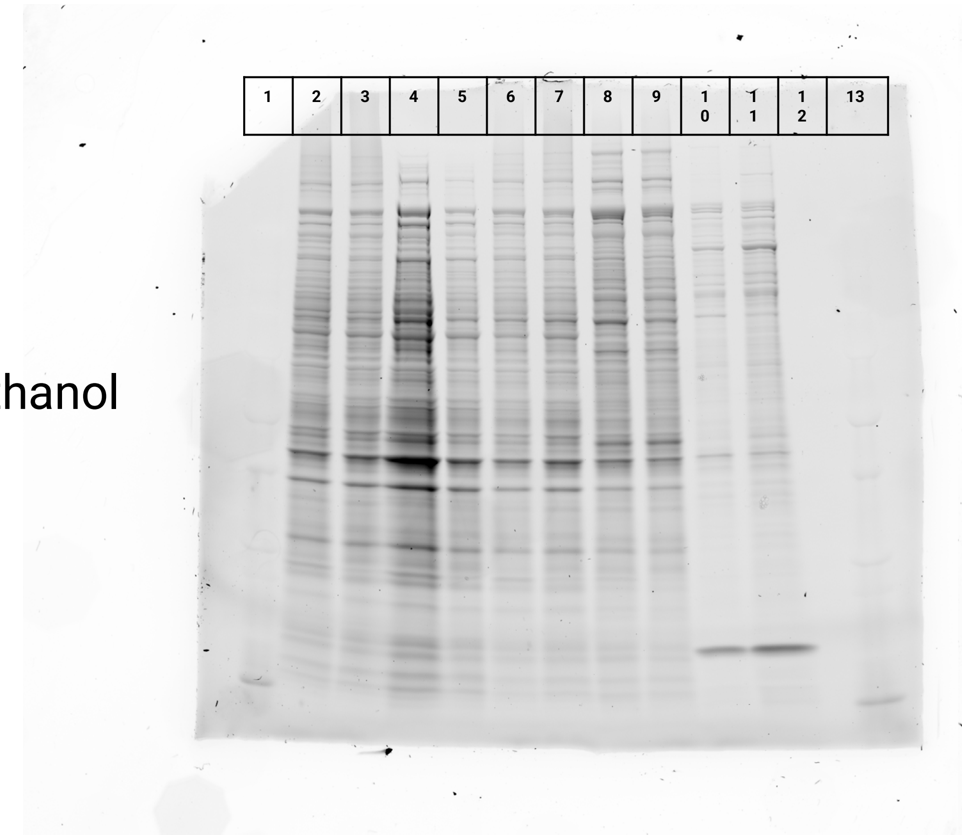

Imaged Myc, CD73, and Rab11a

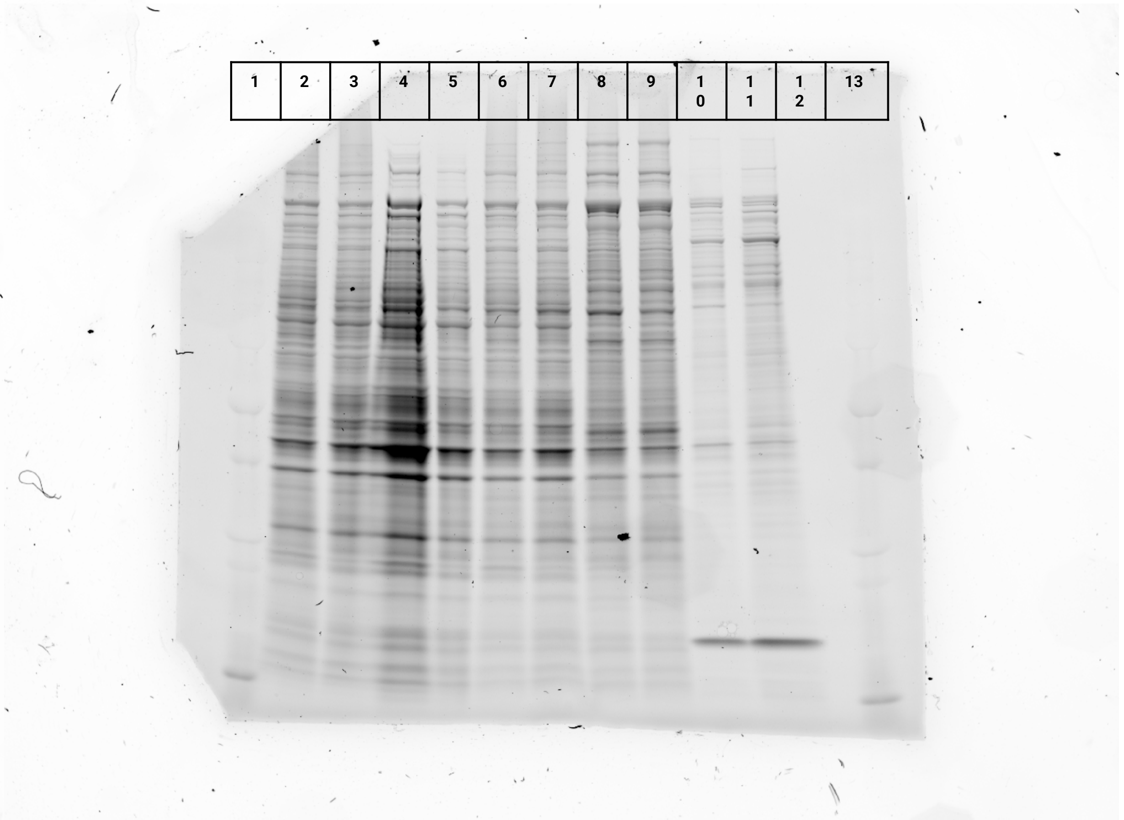

Imaged SP1 and H2AX

1	2	3	4	5	6	7	8	9	10	11	12	13
Ladder	NT5E +/+ WCL	NT5E -/- WCL	NT5E +/+ Cyto	NT5E -/- Cyto	NT5E +/+ Mem	NT5E -/- Mem	NT5E +/+ Nuc	NT5E -/- Nuc	NT5E +/+ Chro	NT5E -/- Chro		Ladder

Figure 5

5C

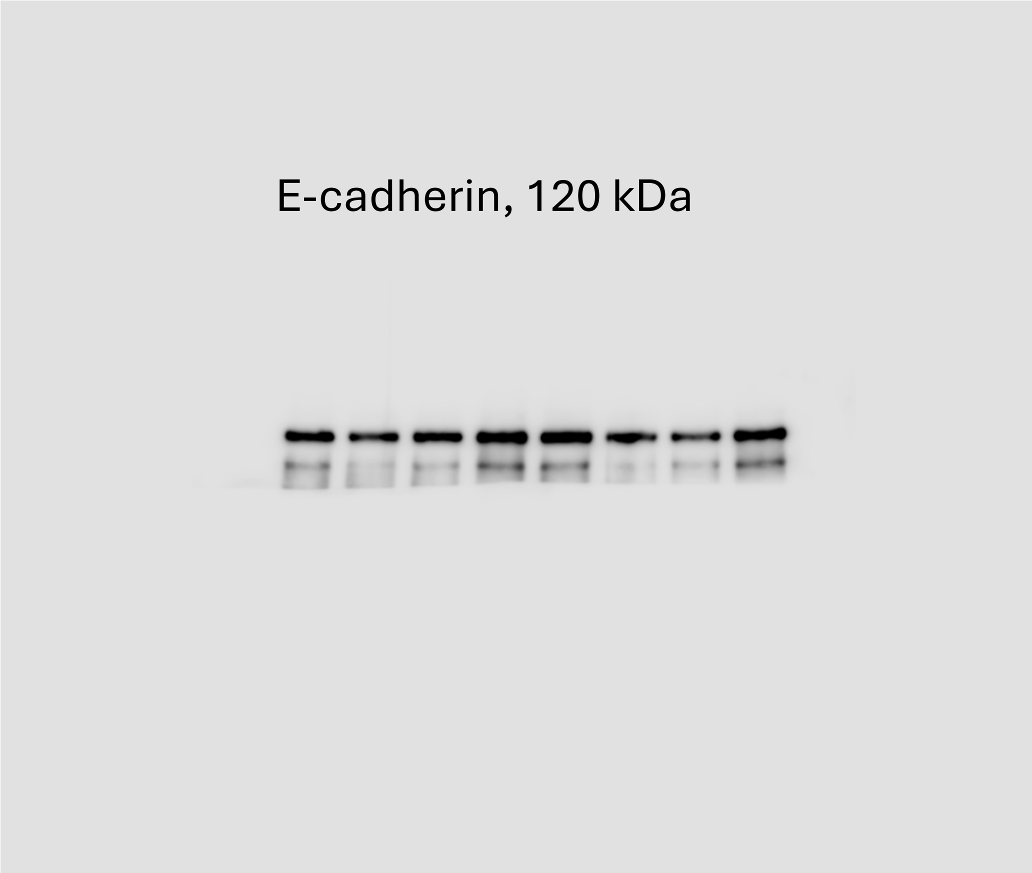

E-cadherin, 120 kDa
BD Biosciences, Cat #36/E-Cadherin

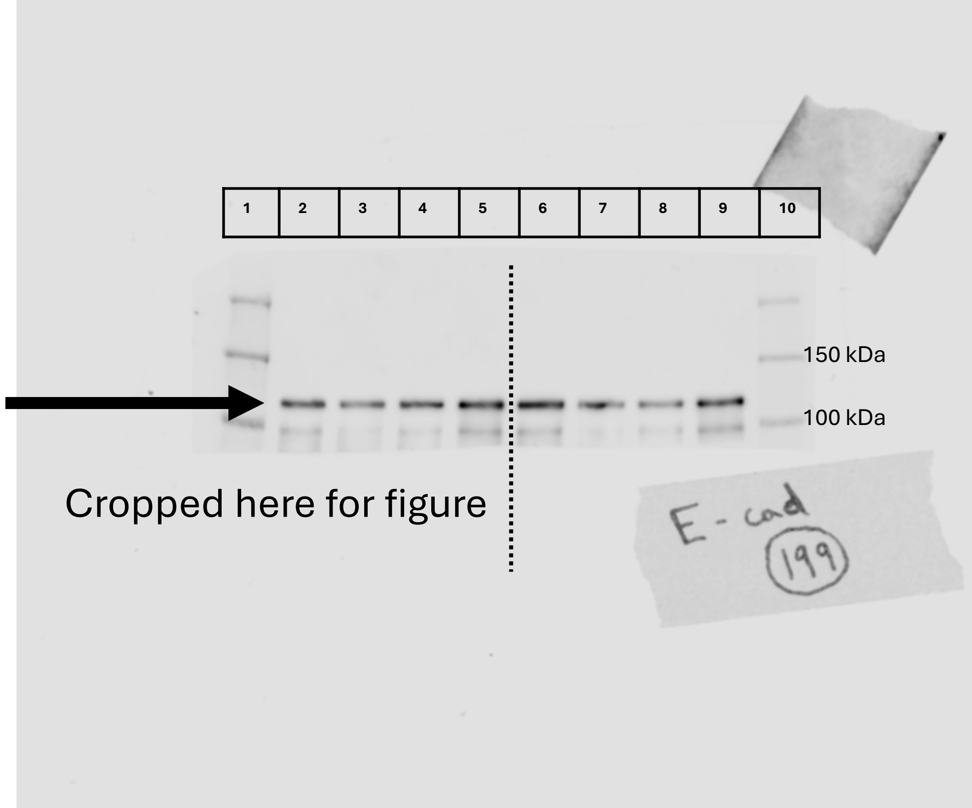

1	2	3	4	5	6	7	8	9	10
Ladder (dark)	HEC-1-A WT	HEC-1-A NT5E KO	HEC-1-A A1R KO	HEC-1-A A2BR KO	HEC-1-A WT	HEC-1-A NT5E KO	HEC-1-A A1R KO	HEC-1-A A2BR KO	Ladder (light)

5C

Total Protein

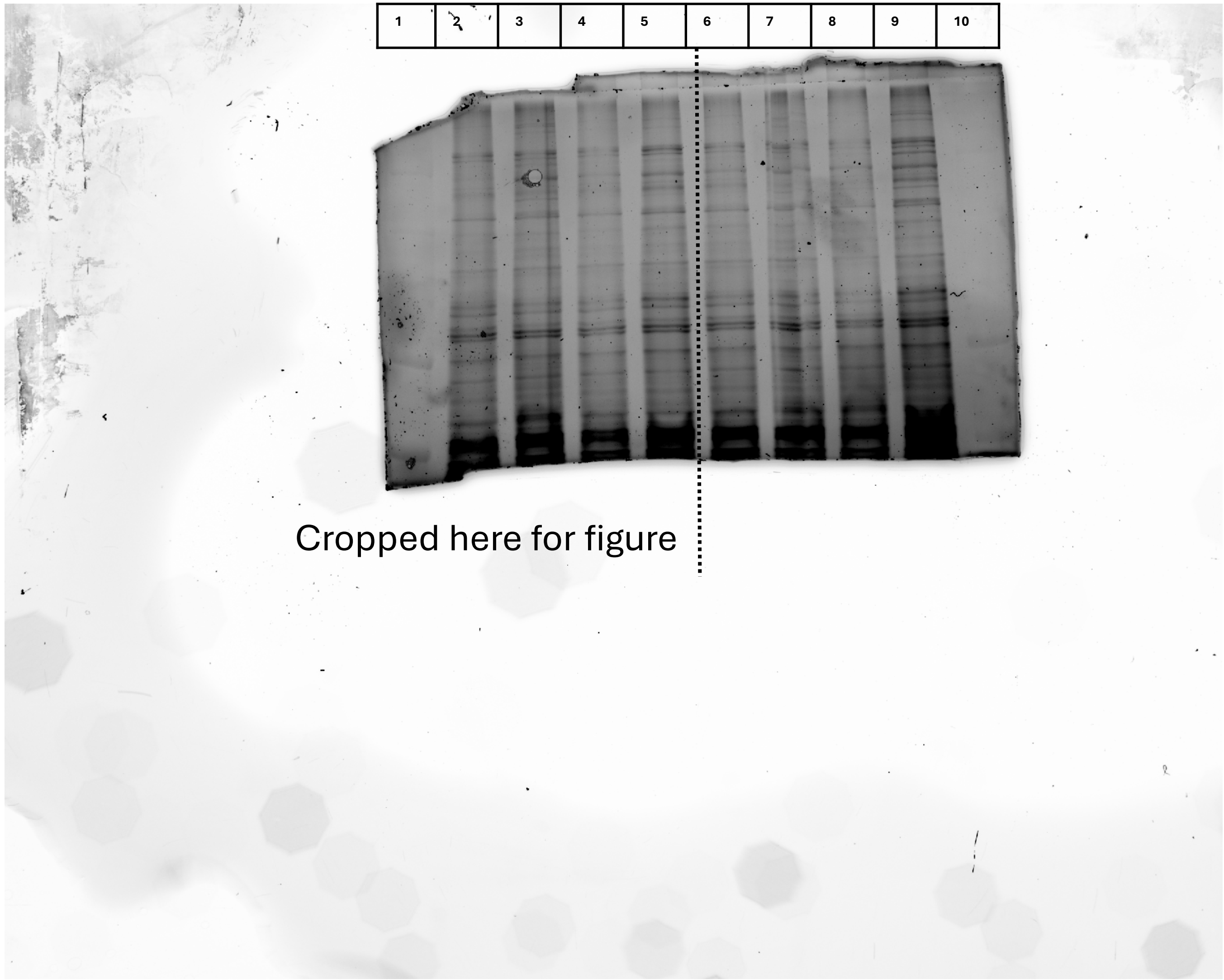

1	2	3	4	5	6	7	8	9	10
Ladder (dark)	HEC-1-A WT	HEC-1-A NT5E KO	HEC-1-A A1R KO	HEC-1-A A2BR KO	HEC-1-A WT	HEC-1-A NT5E KO	HEC-1-A A1R KO	HEC-1-A A2BR KO	Ladder (light)

5D

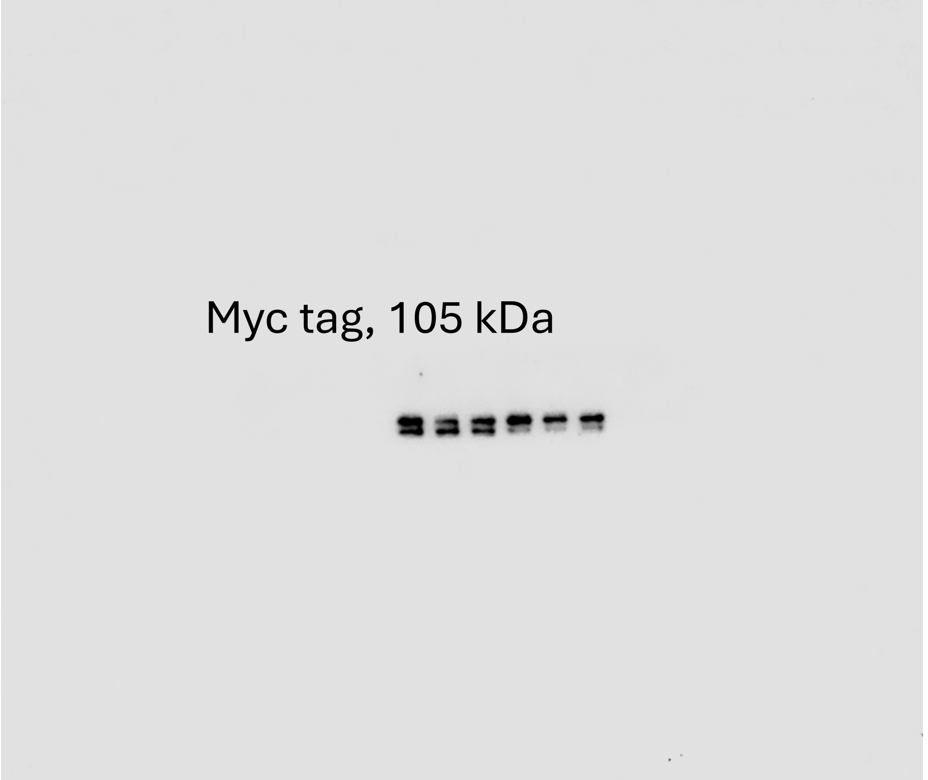

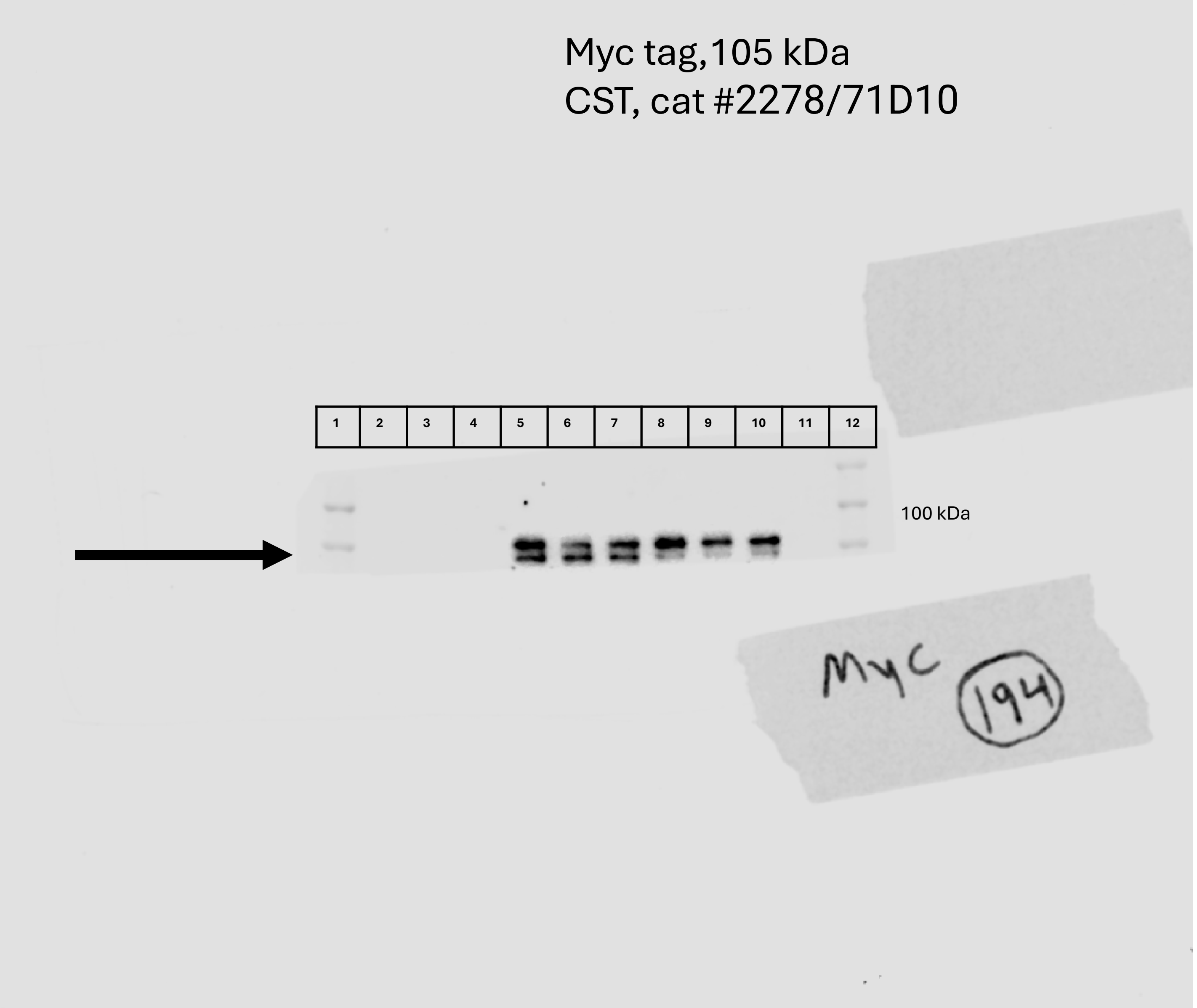

1	2	3	4	5	6	7	8	9	10	11	12
Ladder	HEC-1-A WT, EV	A1R KO, EV	A2BR KO, EV	HEC-1-A WT, G34R	A1R KO, G34R	A2BR KO, G34R	HEC-1-A WT, S37F	A1R KO, S37F	A2BR KO, S37F		Ladder

5D

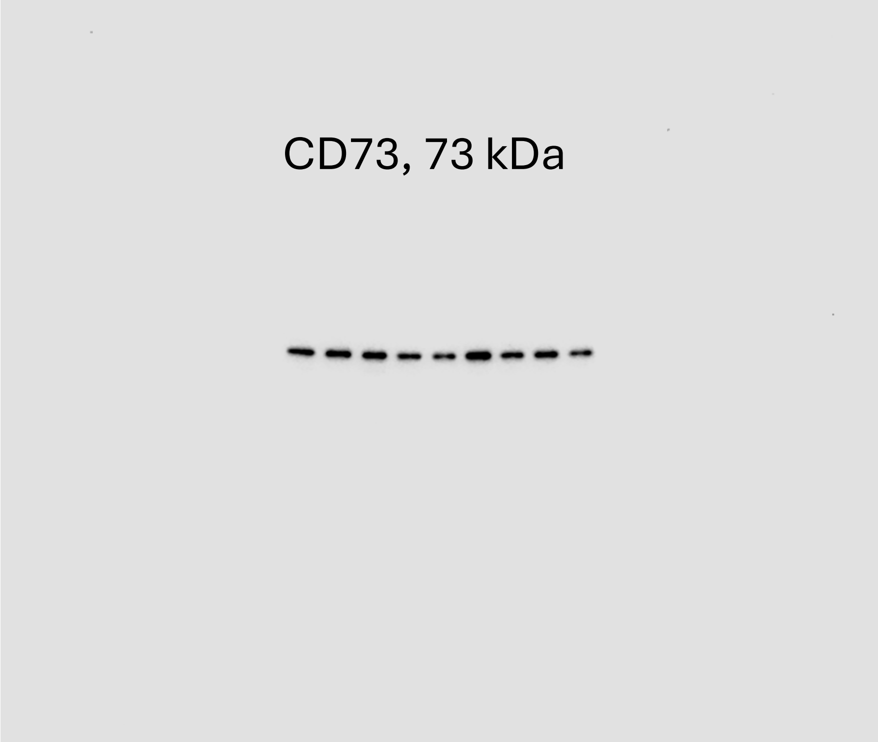

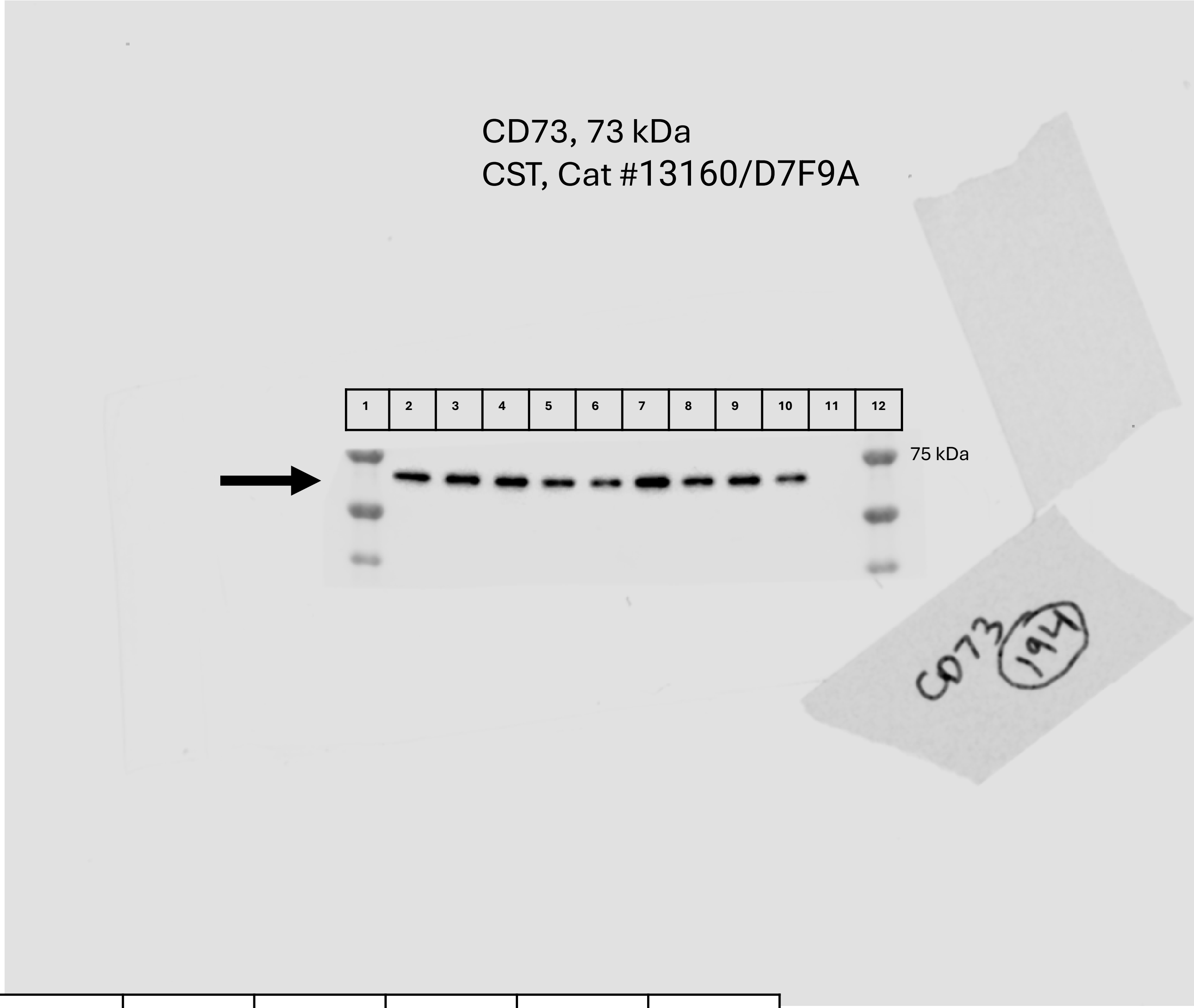

1	2	3	4	5	6	7	8	9	10	11	12
Ladder	HEC-1-A WT, EV	A1R KO, EV	A2BR KO, EV	HEC-1-A WT, G34R	A1R KO, G34R	A2BR KO, G34R	HEC-1-A WT, S37F	A1R KO, S37F	A2BR KO, S37F		Ladder

5D

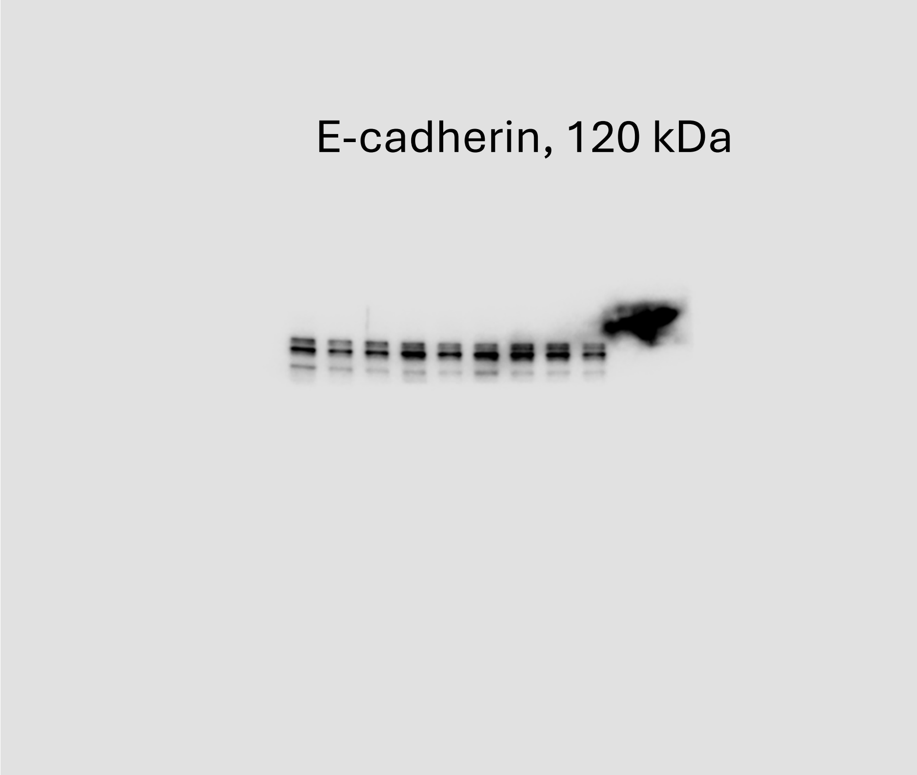

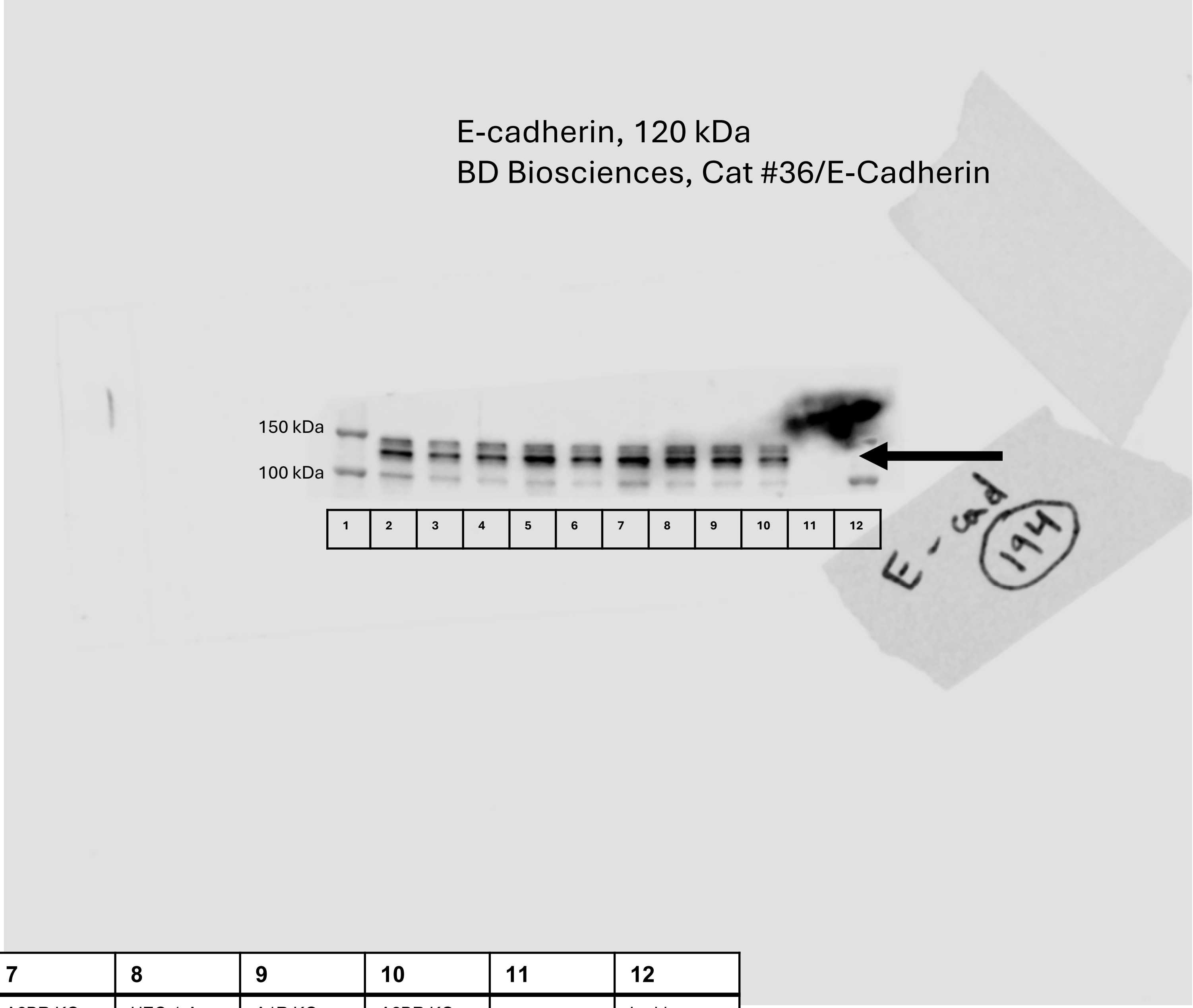

1	2	3	4	5	6	7	8	9	10	11	12
Ladder	HEC-1-A WT, EV	A1R KO, EV	A2BR KO, EV	HEC-1-A WT, G34R	A1R KO, G34R	A2BR KO, G34R	HEC-1-A WT, S37F	A1R KO, S37F	A2BR KO, S37F		Ladder

5D

Total Protein

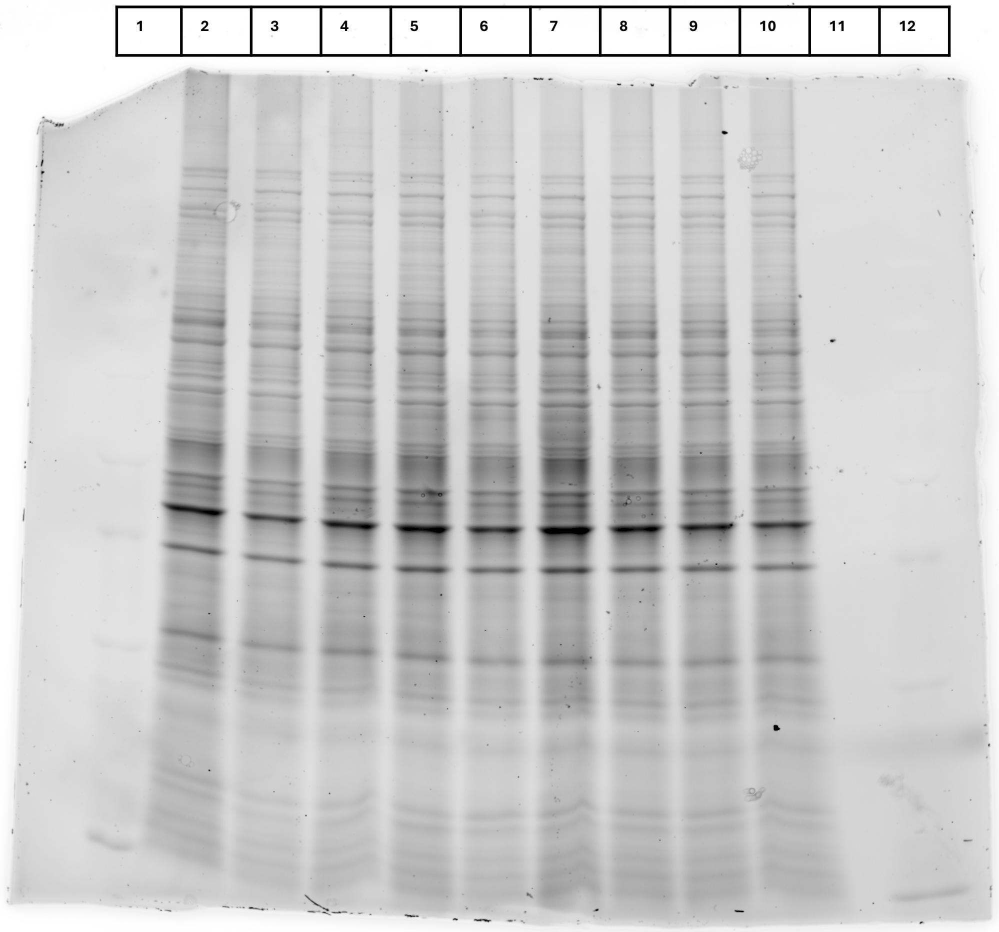

1	2	3	4	5	6	7	8	9	10	11	12
Ladder	HEC-1-A WT, EV	A1R KO, EV	A2BR KO, EV	HEC-1-A WT, G34R	A1R KO, G34R	A2BR KO, G34R	HEC-1-A WT, S37F	A1R KO, S37F	A2BR KO, S37F		Ladder

Figure 6

6A - Myc

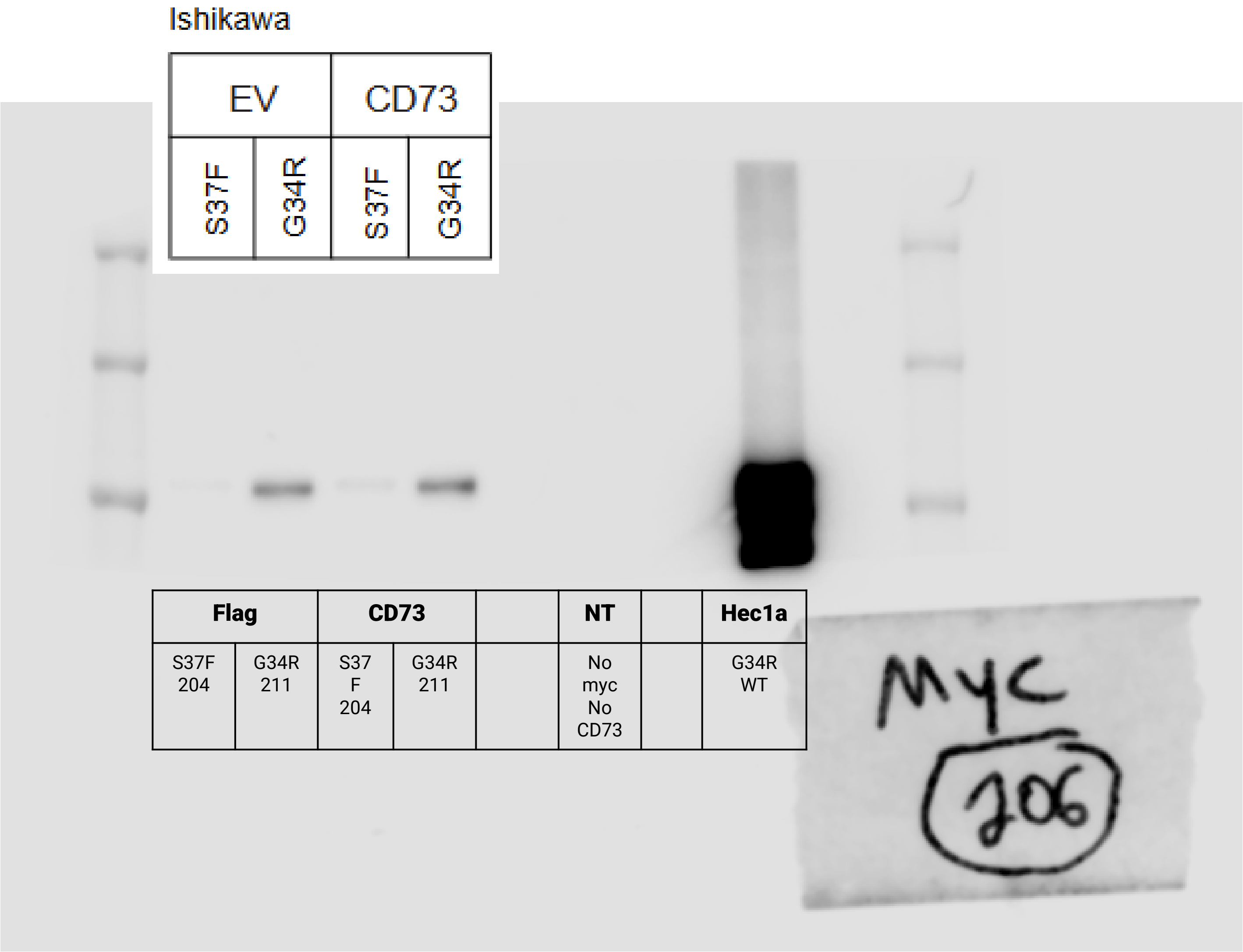

Short exposure

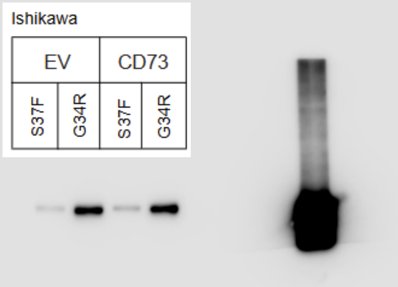

Long exposure

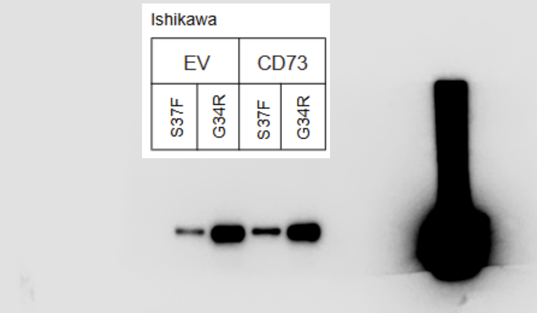

6A – CD73 & total protein

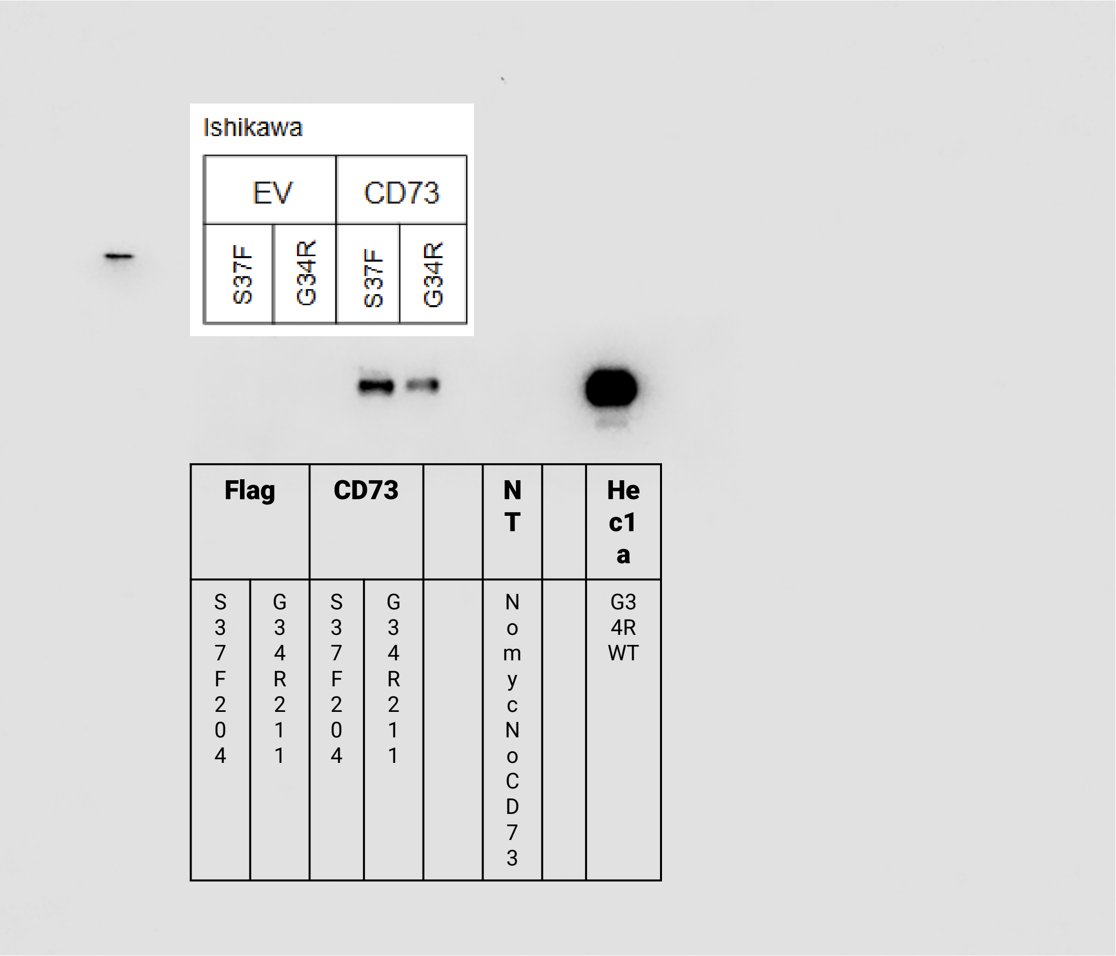

Flag		CD73			NT		Hec1a
S37F 204	G34R 211	S37 F 204	G34R 211		No myc No CD73		G34R WT

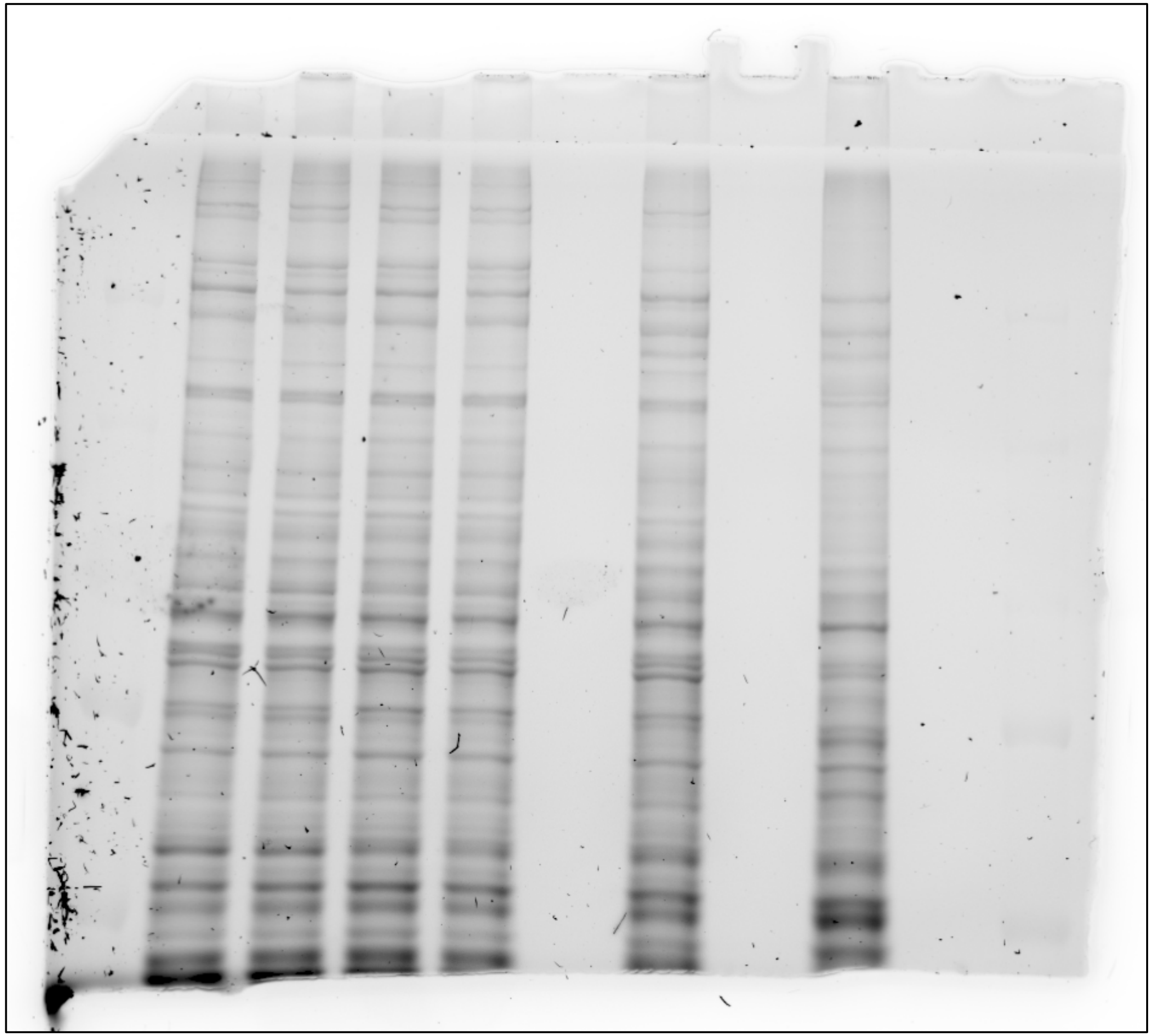

6A – CD73

Flag		CD73			NT		Hec1 a
S37F 204	G34 R 211	S37 F 204	G34 R 211		No myc No CD7 3		G34R WT

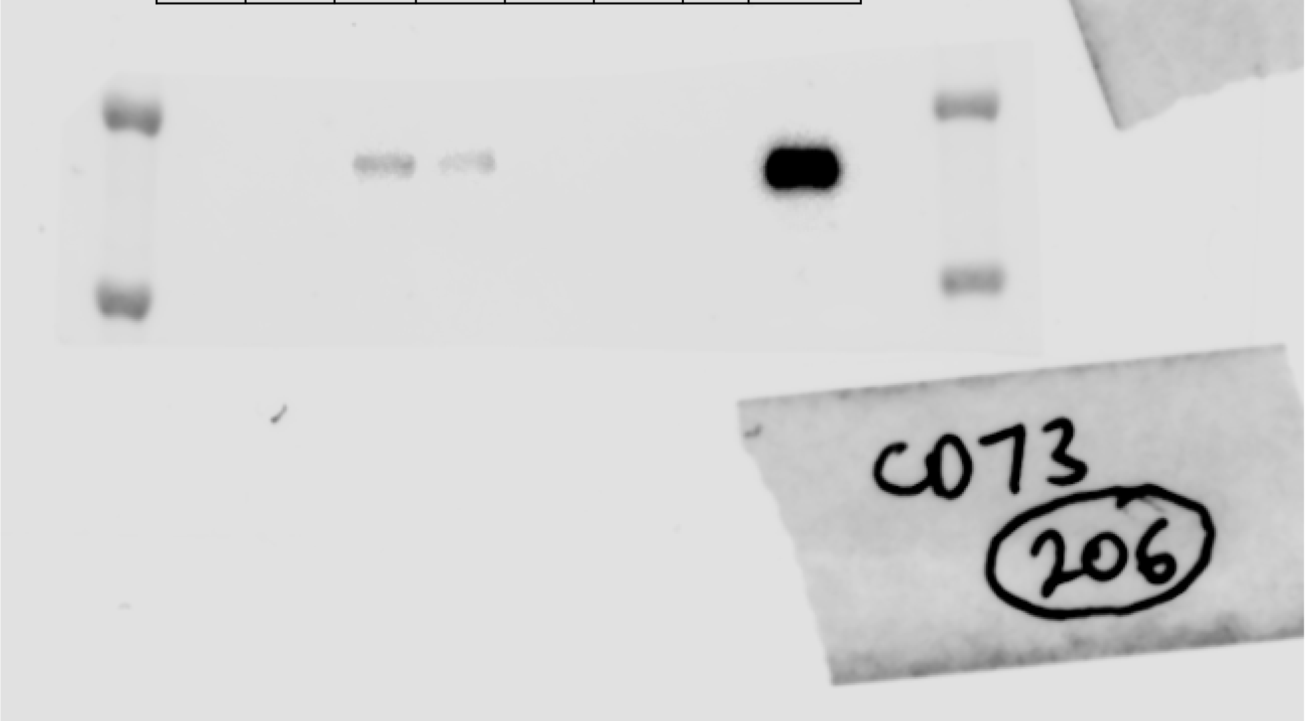

Figure S2

S2 A

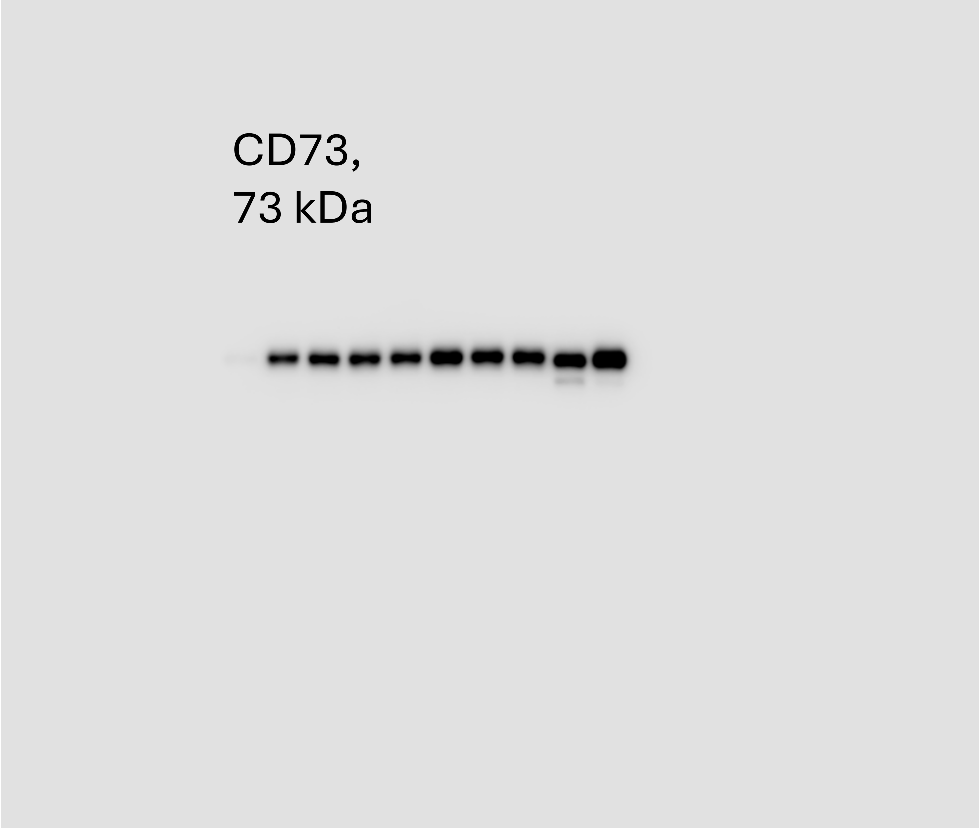

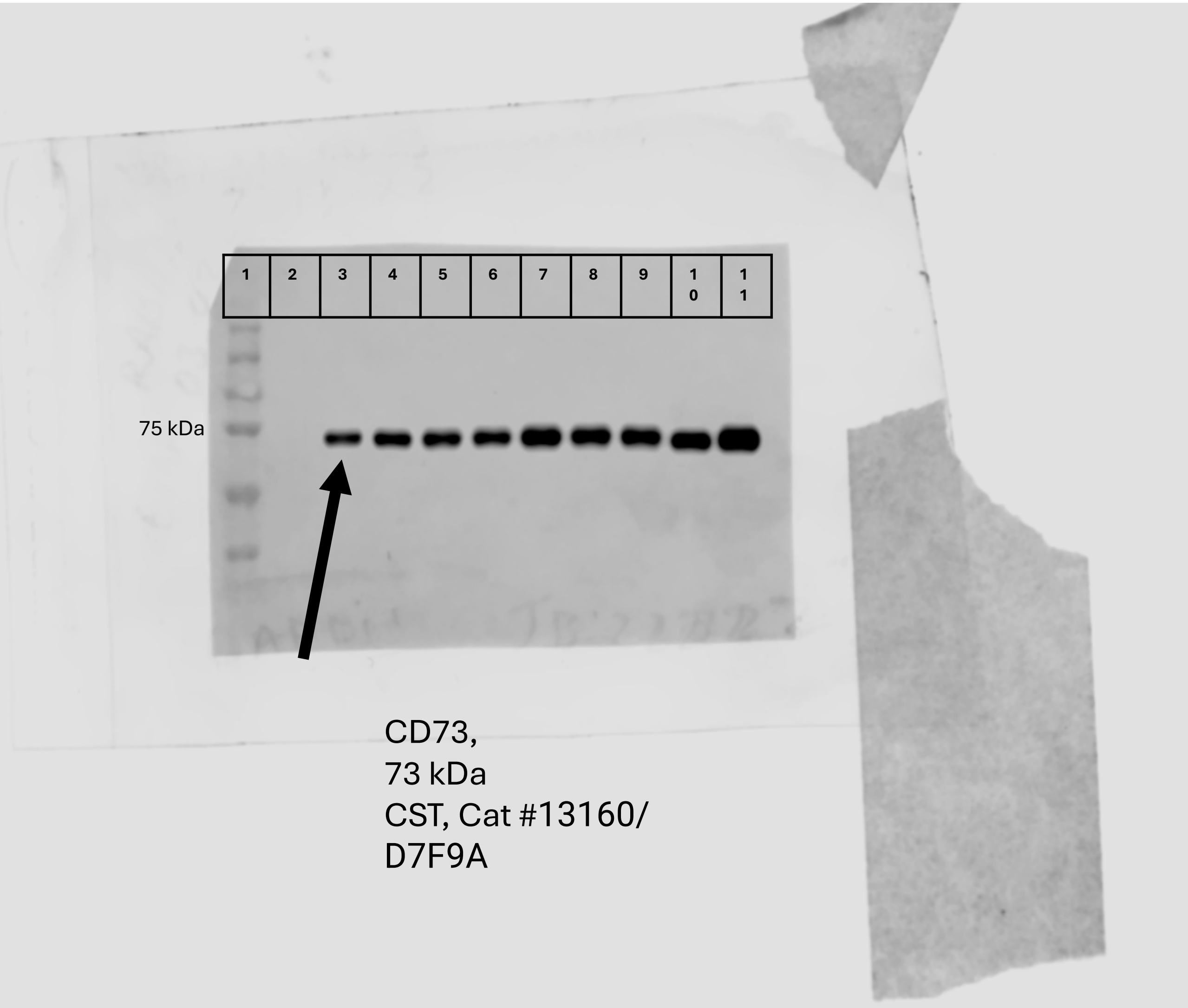

1	2	3	4	5	6	7	8	9	10	11
Ladder	No transduction	NT5E AdV, 1.5E8 IU/ml	NT5E AdV, 2E8 IU/ml	NT5E AdV, 2.5E8 IU/ml	NT5E AdV, 3E8 IU/ml	NT5E AdV, 3.5E8 IU/ml	NT5E AdV, 4E8 IU/ml	NT5E AdV, 4.5E8 IU/ml	HEC-1-A 100% confluency	HEC-1-A 2D post confluency

S2 A

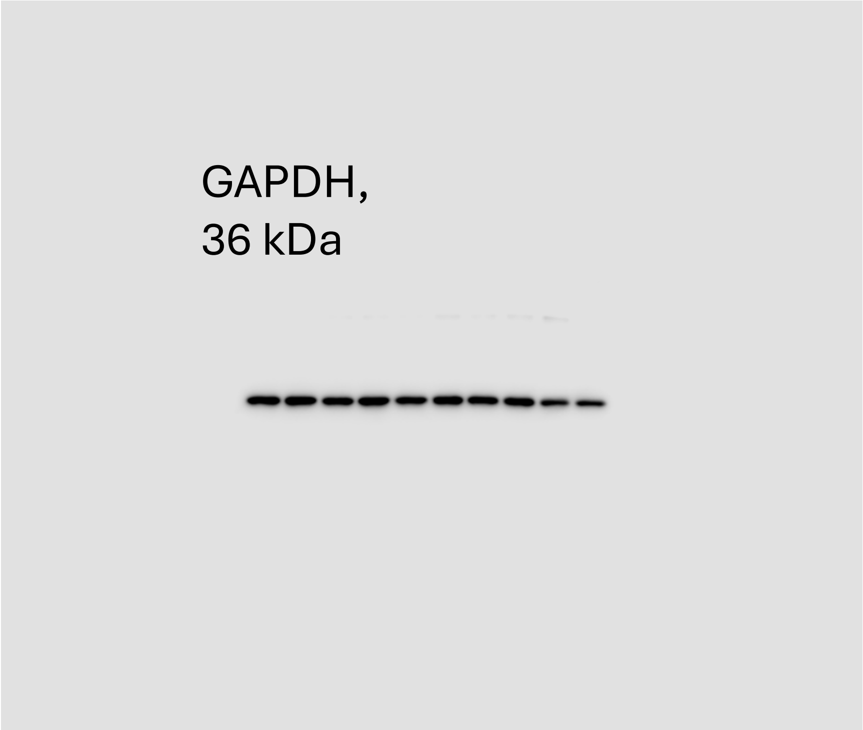

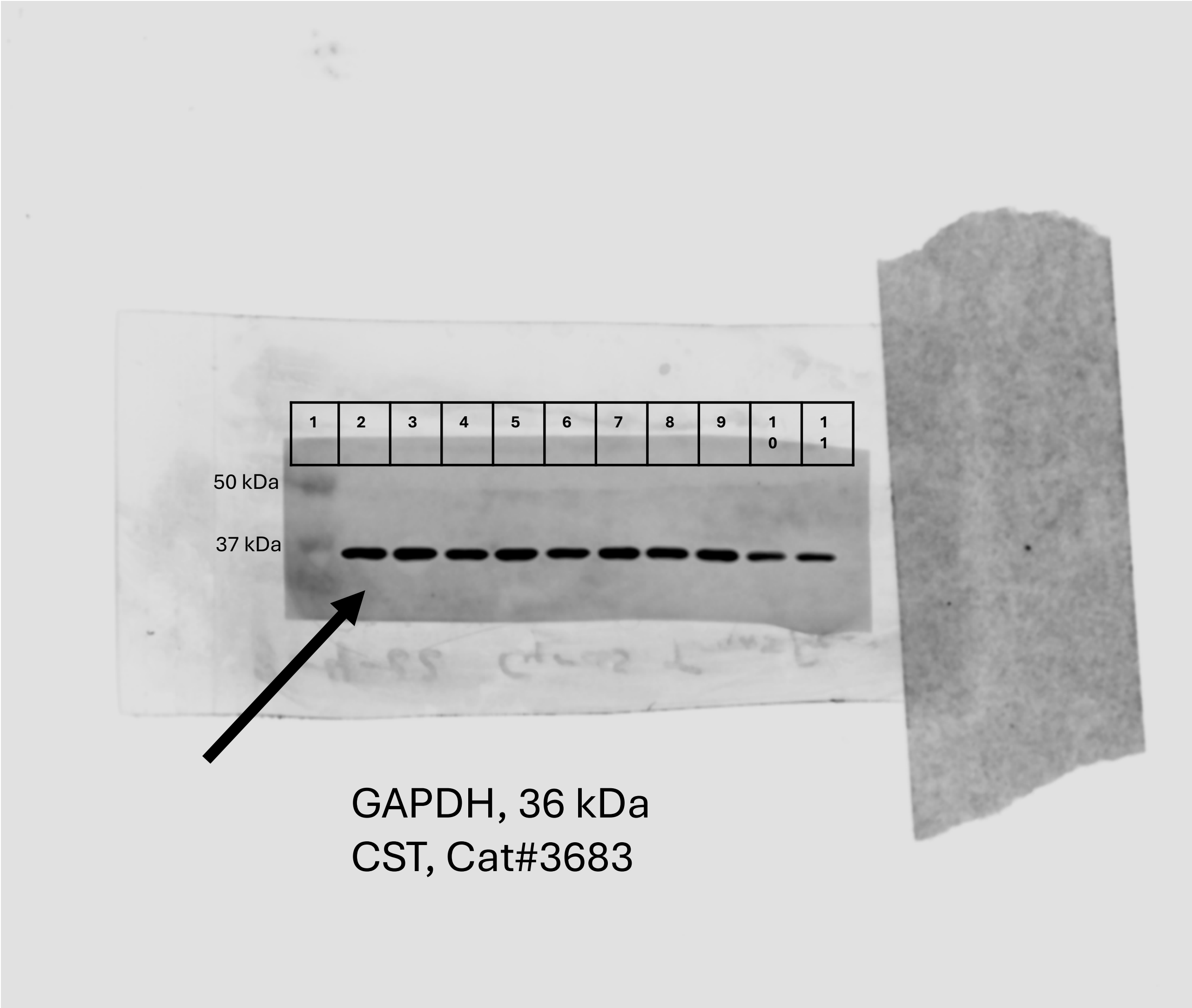

1	2	3	4	5	6	7	8	9	10	11
Ladder	No transduction	NT5E AdV, 1.5E8 IU/ml	NT5E AdV, 2E8 IU/ml	NT5E AdV, 2.5E8 IU/ml	NT5E AdV, 3E8 IU/ml	NT5E AdV, 3.5E8 IU/ml	NT5E AdV, 4E8 IU/ml	NT5E AdV, 4.5E8 IU/ml	HEC-1-A 100% confluency	HEC-1-A 2D post confluency

S2 B

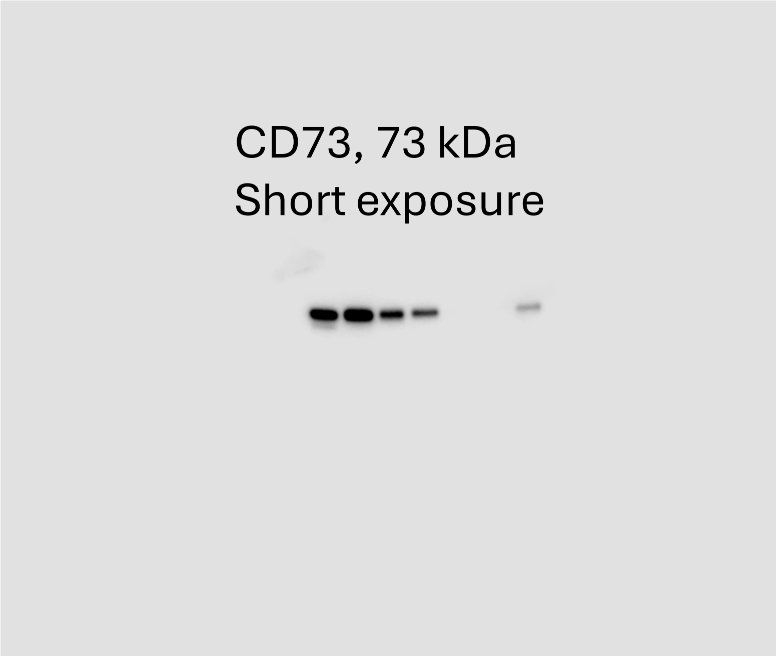

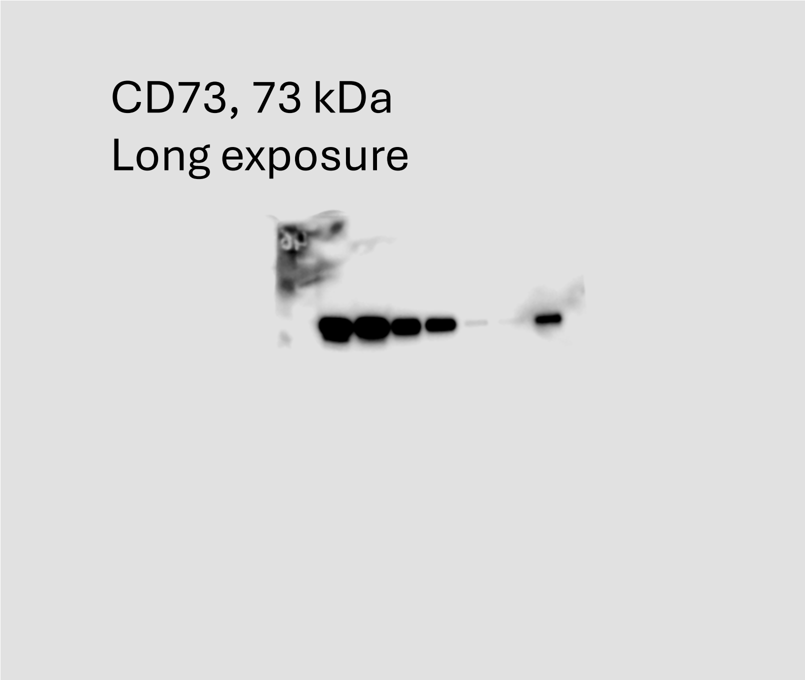

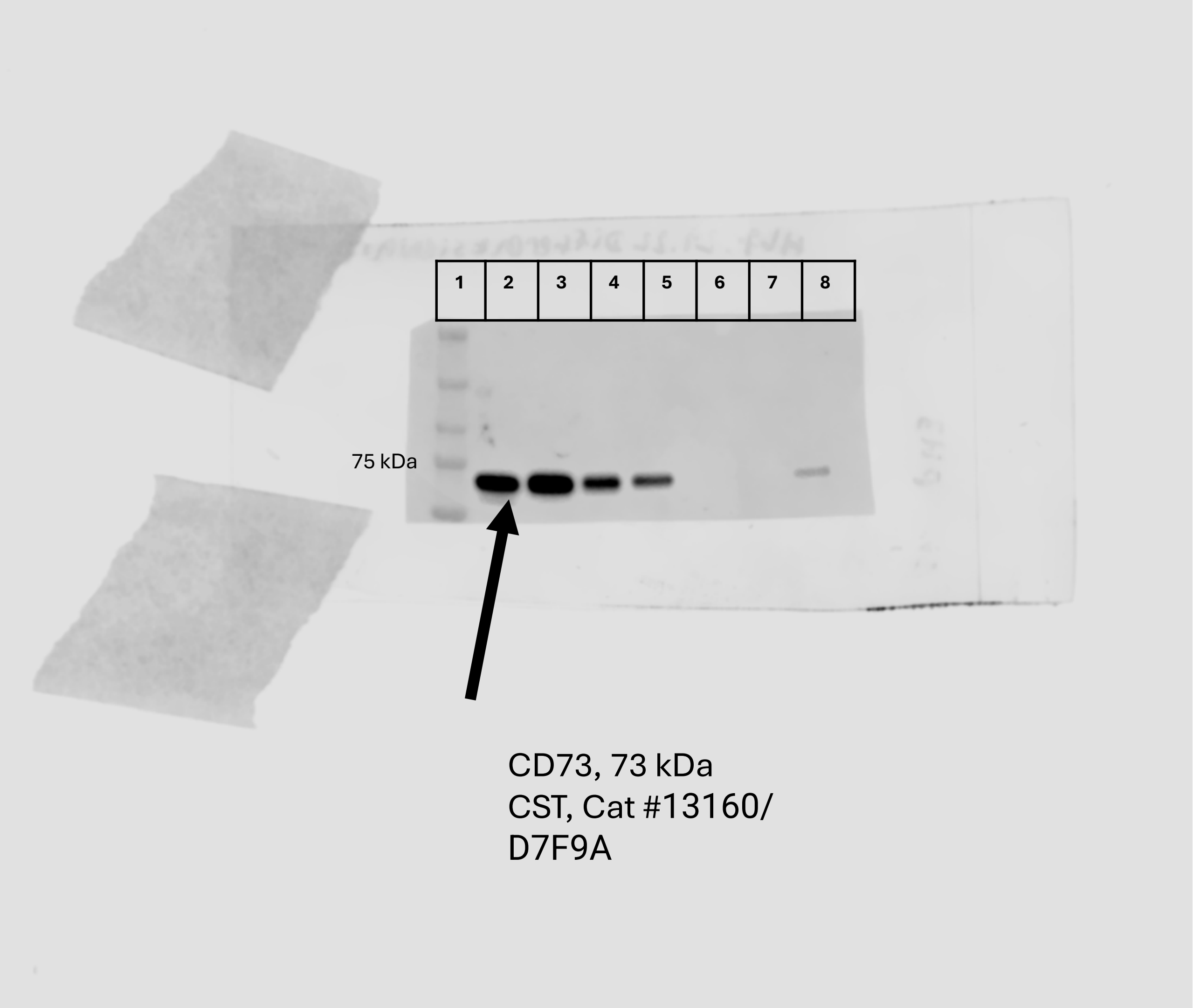

1	2	3	4	5	6	7	8
Ladder	HEC-1-A 100% confluent	HEC-1-A 2D post confluency	NT5E AdV, 4E8 IU/ml 48 hr, old exp	NT5E AdV, 4E8 IU/ml 48 hr	Ctrl AdV, 4E8 IU/ml 48 hr	Ctrl AdV, 1E9 IU/ml 96 hr	NT5E AdV, 4E8 IU/ml 96 hr

S2 B

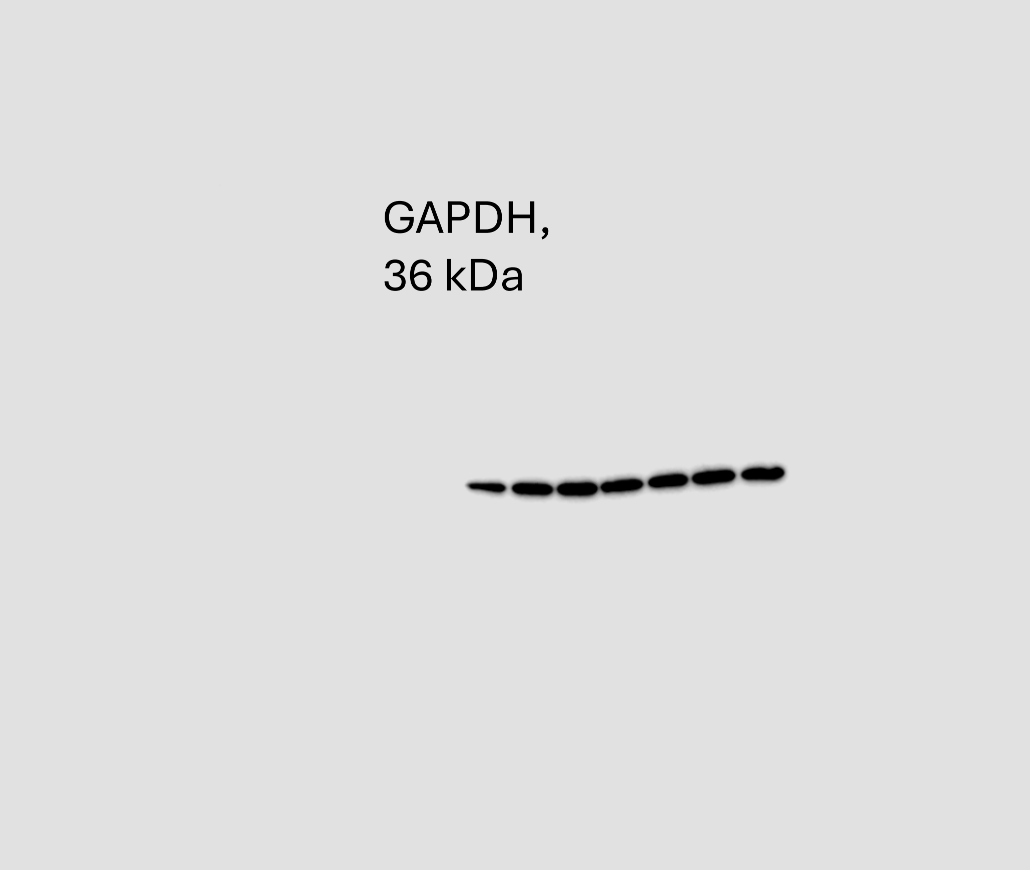

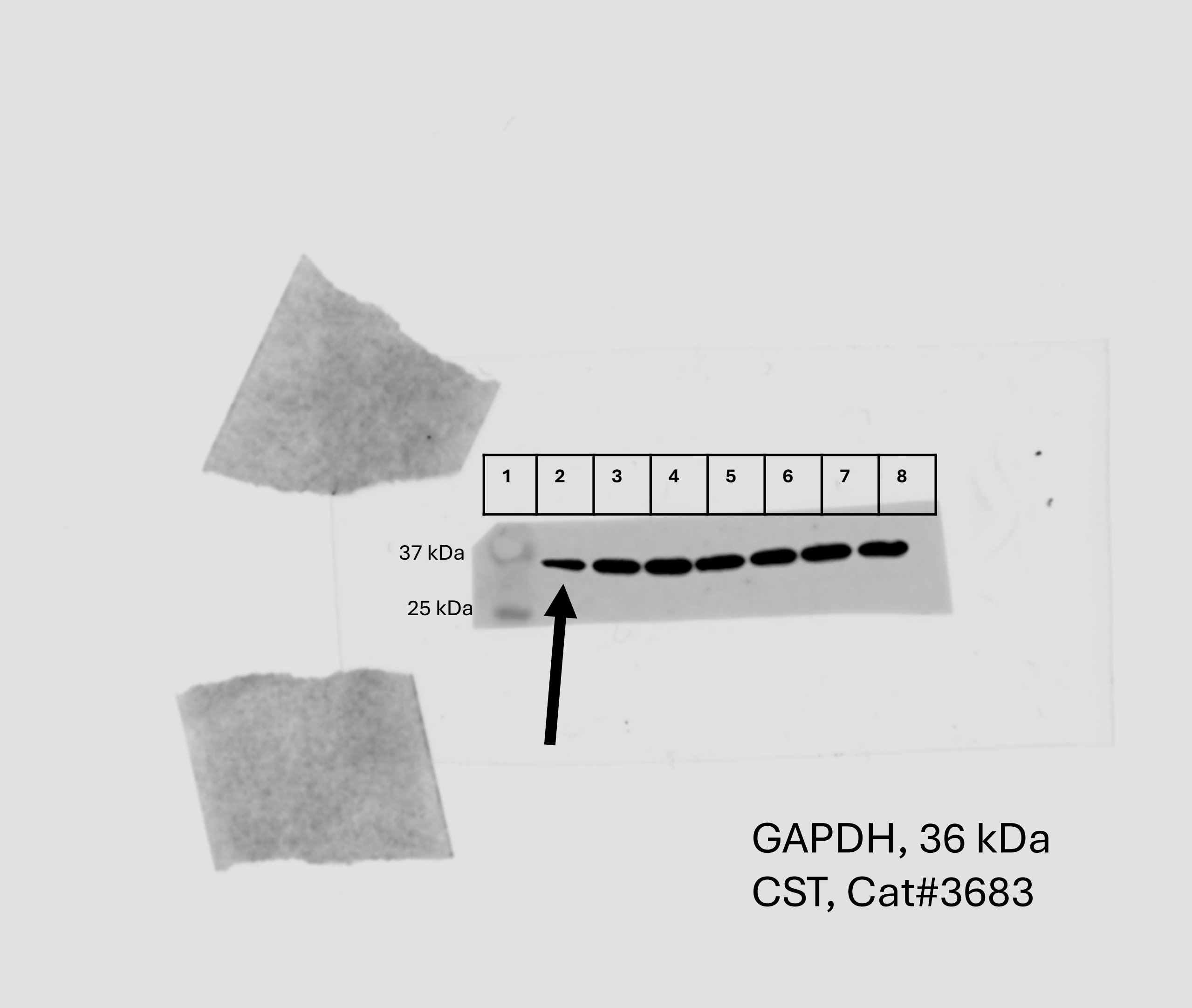

1	2	3	4	5	6	7	8
Ladder	HEC-1-A<br>100%<br>confluent	HEC-1-A 2D<br>post<br>confluency	NT5E AdV, 4E8<br>IU/ml<br>48 hr, old exp	NT5E AdV, 4E8<br>IU/ml<br>48 hr	Ctrl AdV, 4E8<br>IU/ml<br>48 hr	Ctrl AdV, 1E9<br>IU/ml<br>96 hr	NT5E AdV, 4E8<br>IU/ml<br>96 hr

Figure S4
WB Images

S4 A images, Myc

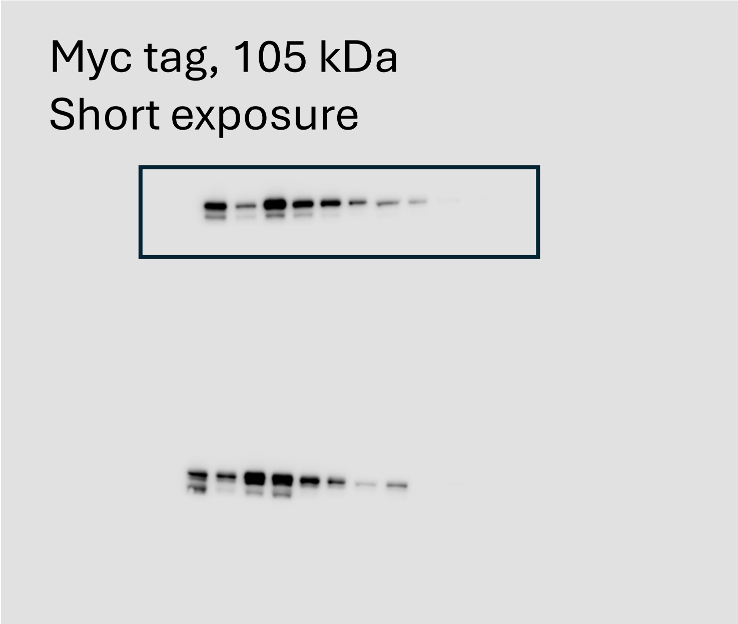

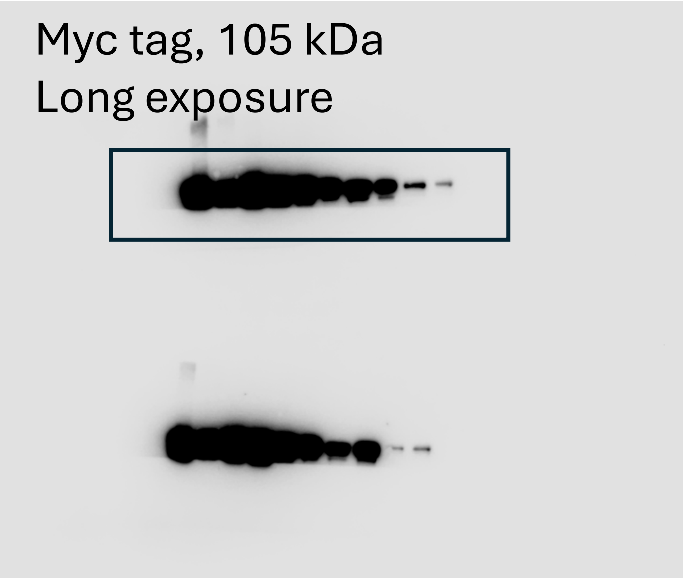

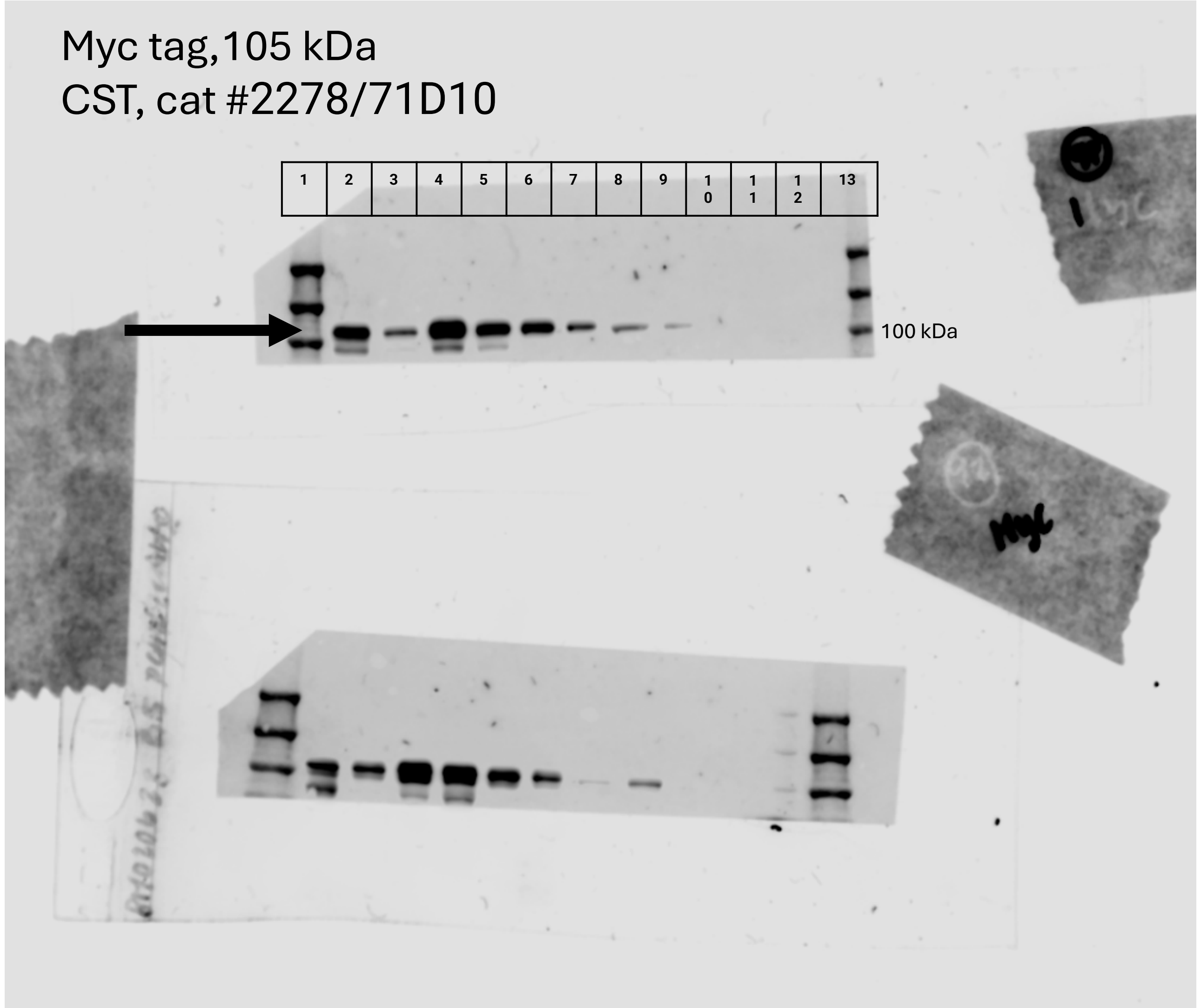

1	2	3	4	5	6	7	8	9	10	11	12	13
Ladder	NT5E $^{+/+}$ WCL	NT5E $^{-/-}$ WCL	NT5E $^{+/+}$ Cyto	NT5E $^{-/-}$ Cyto	NT5E $^{+/+}$ Mem	NT5E $^{-/-}$ Mem	NT5E $^{+/+}$ Nuc	NT5E $^{-/-}$ Nuc	NT5E $^{+/+}$ Chro	NT5E $^{-/-}$ Chro		Ladder

S4 A images, Rab11a

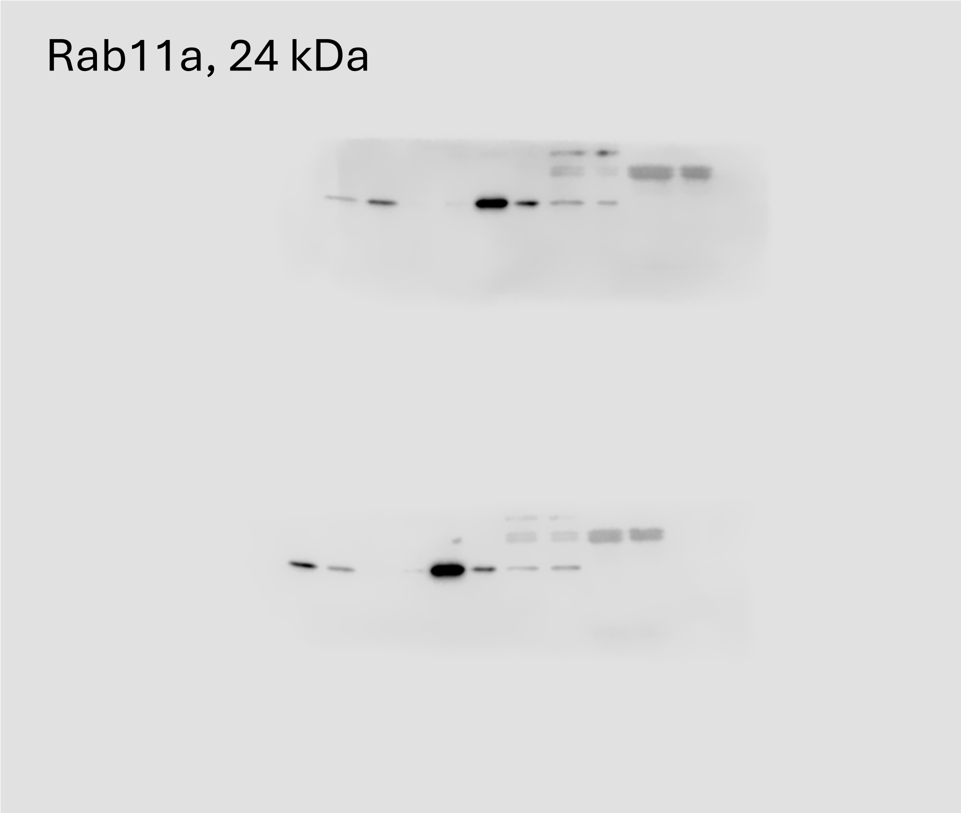

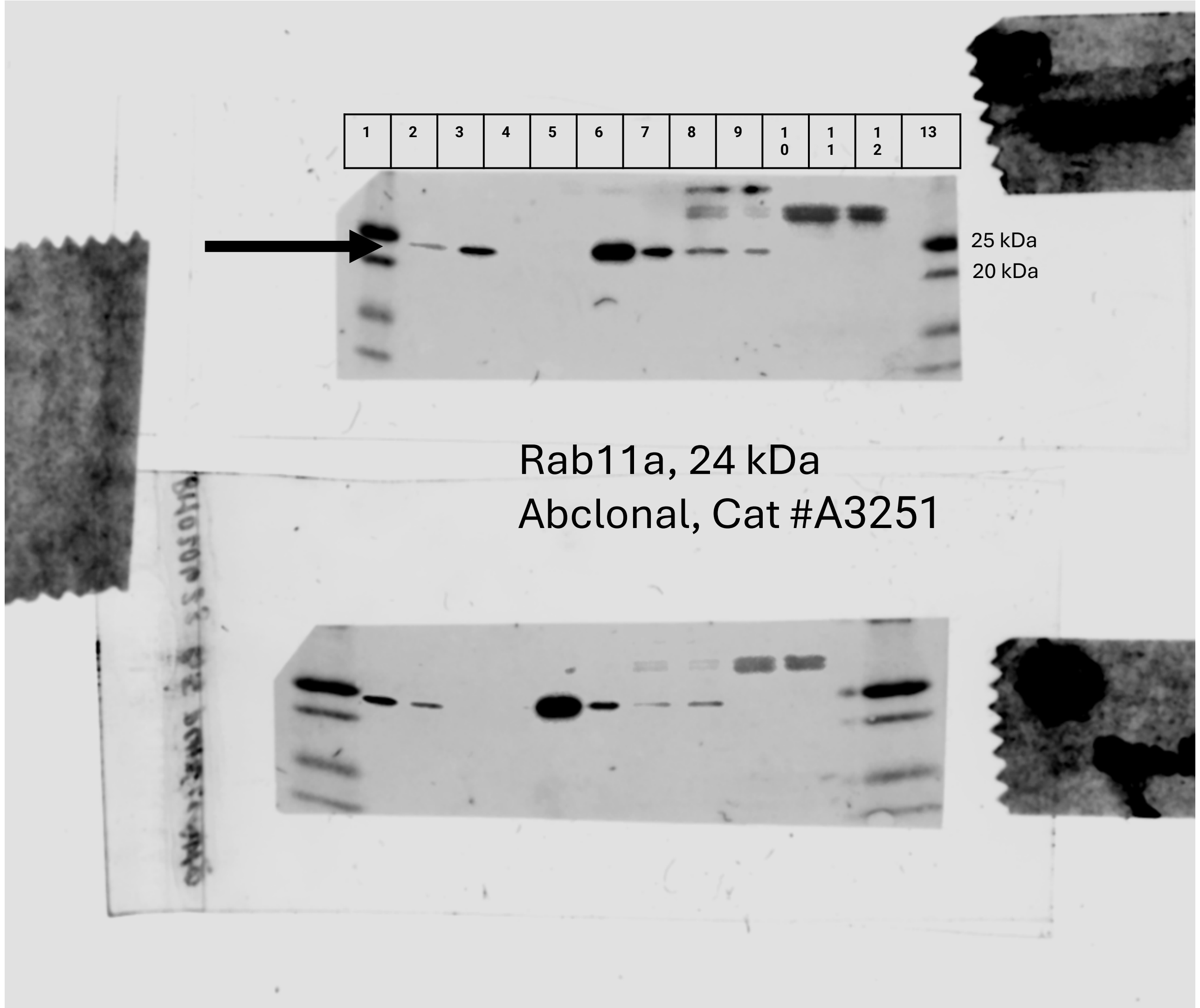

1	2	3	4	5	6	7	8	9	10	11	12	13
Ladder	NT5E $^{+/+}$ WCL	NT5E $^{-/-}$ WCL	NT5E $^{+/+}$ Cyto	NT5E $^{-/-}$ Cyto	NT5E $^{+/+}$ Mem	NT5E $^{-/-}$ Mem	NT5E $^{+/+}$ Nuc	NT5E $^{-/-}$ Nuc	NT5E $^{+/+}$ Chro	NT5E $^{-/-}$ Chro		Ladder

S4 A images, SP1

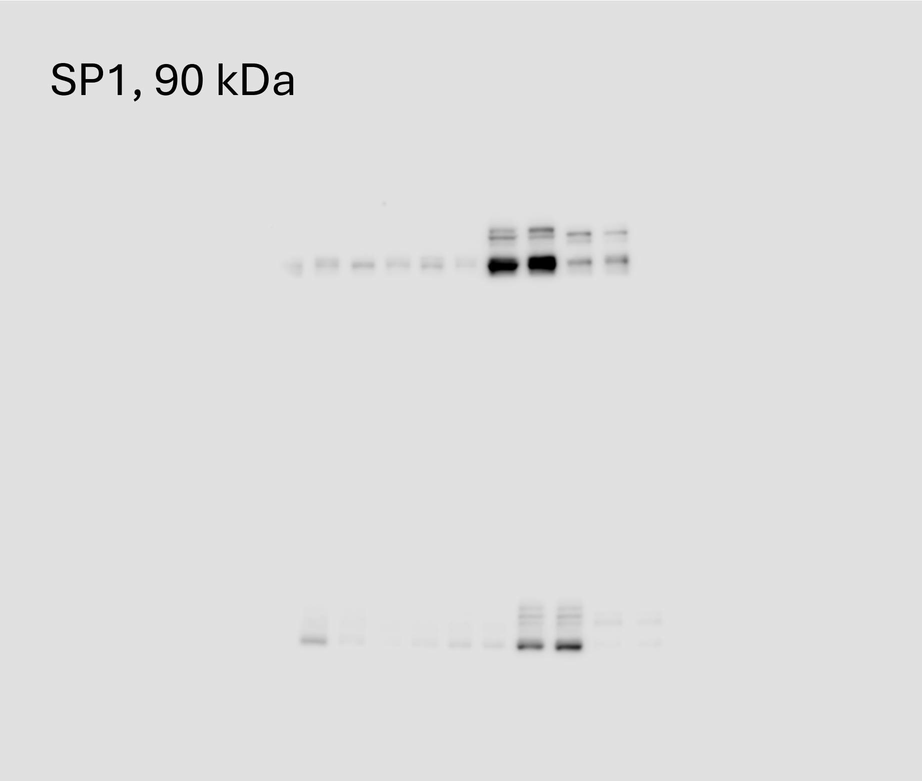

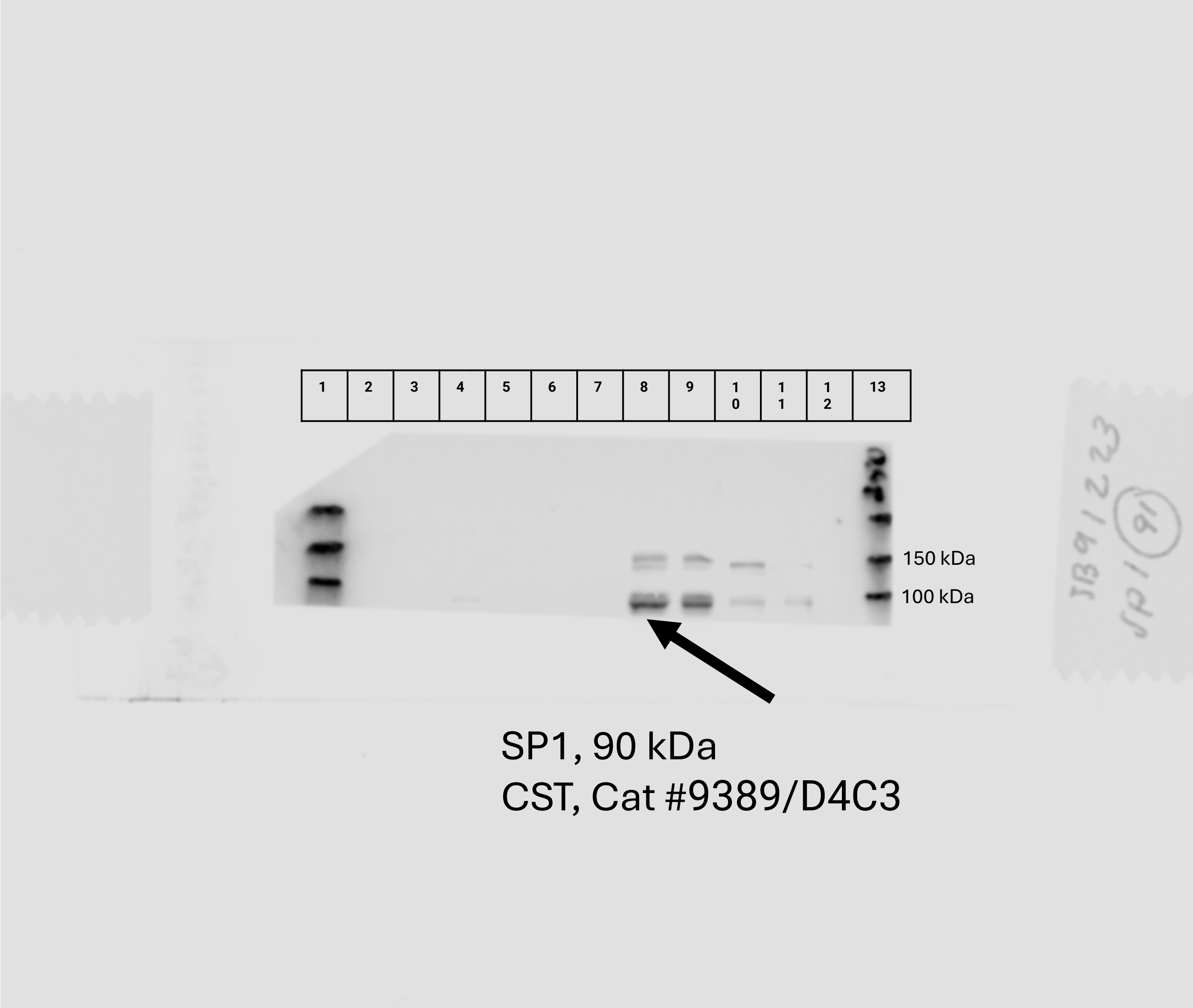

1	2	3	4	5	6	7	8	9	10	11	12	13
Ladder	NT5E $^{+/+}$ WCL	NT5E $^{-/-}$ WCL	NT5E $^{+/+}$ Cyto	NT5E $^{-/-}$ Cyto	NT5E $^{+/+}$ Mem	NT5E $^{-/-}$ Mem	NT5E $^{+/+}$ Nuc	NT5E $^{-/-}$ Nuc	NT5E $^{+/+}$ Chro	NT5E $^{-/-}$ Chro		Ladder

S4 A images, H2AX

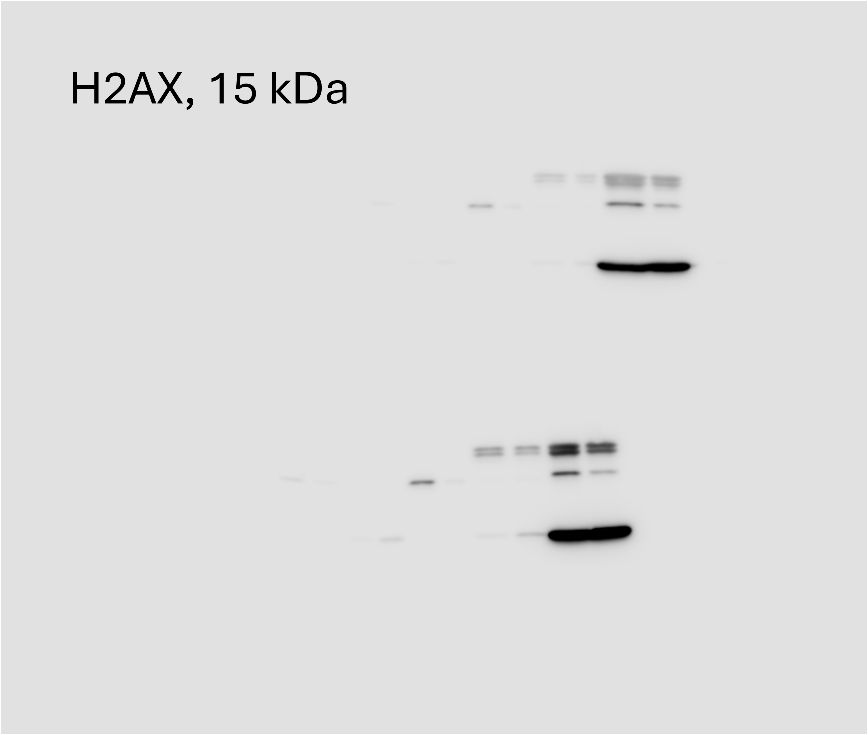

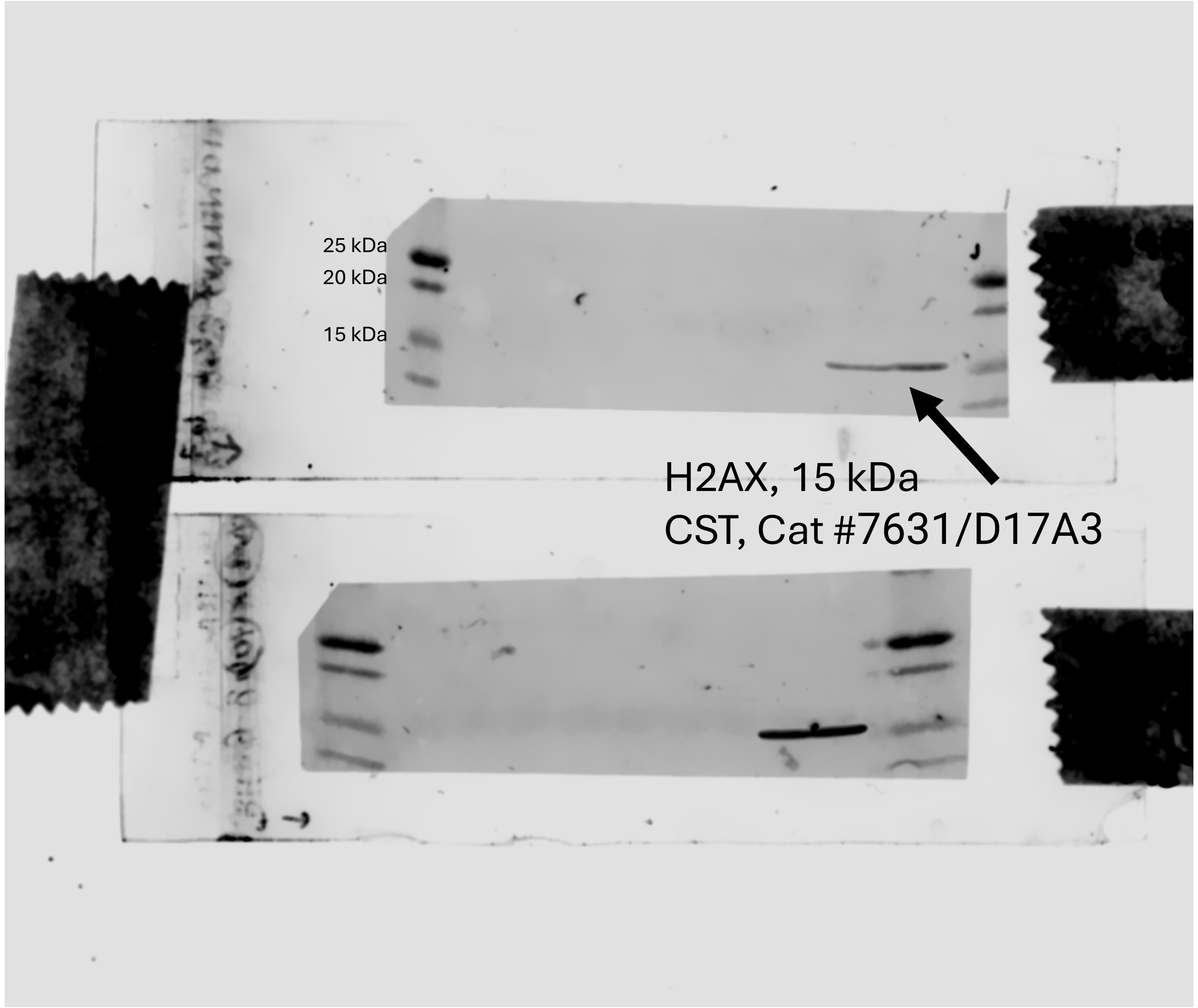

1	2	3	4	5	6	7	8	9	10	11	12	13
Ladder	NT5E $^{+/+}$ WCL	NT5E $^{-/-}$ WCL	NT5E $^{+/+}$ Cyto	NT5E $^{-/-}$ Cyto	NT5E $^{+/+}$ Mem	NT5E $^{-/-}$ Mem	NT5E $^{+/+}$ Nuc	NT5E $^{-/-}$ Nuc	NT5E $^{+/+}$ Chro	NT5E $^{-/-}$ Chro		Ladder

S4 A images

Total protein:
10% 2,2,2-trichloroethanol

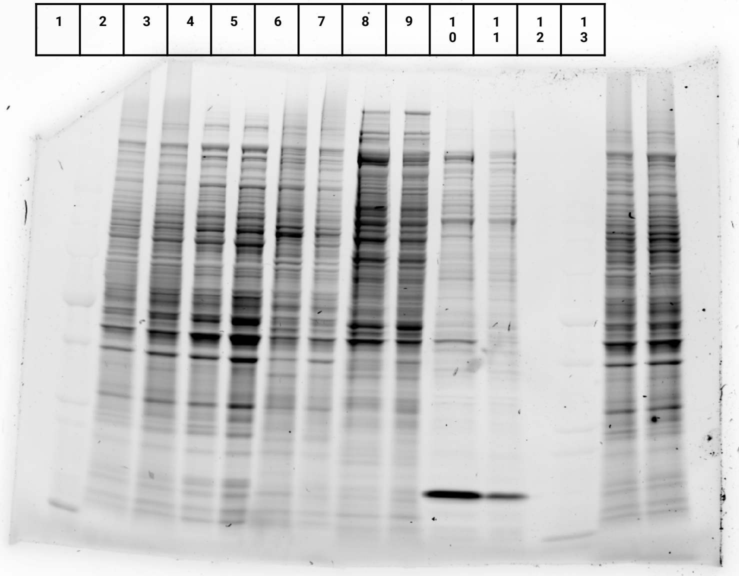

1	2	3	4	5	6	7	8	9	10	11	12	13
Ladder	NT5E +/+ WCL	NT5E -/- WCL	NT5E +/+ Cyto	NT5E -/- Cyto	NT5E +/+ Mem	NT5E -/- Mem	NT5E +/+ Nuc	NT5E -/- Nuc	NT5E +/+ Chro	NT5E -/- Chro		Ladder

S4 B Images - Myc

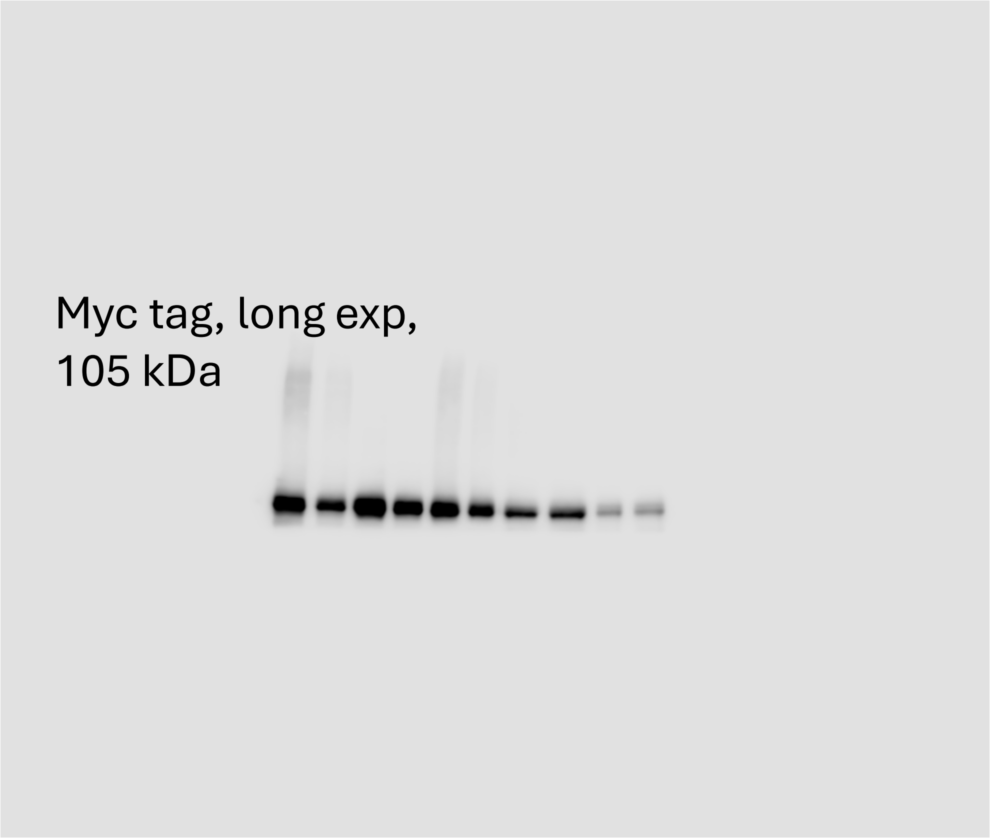

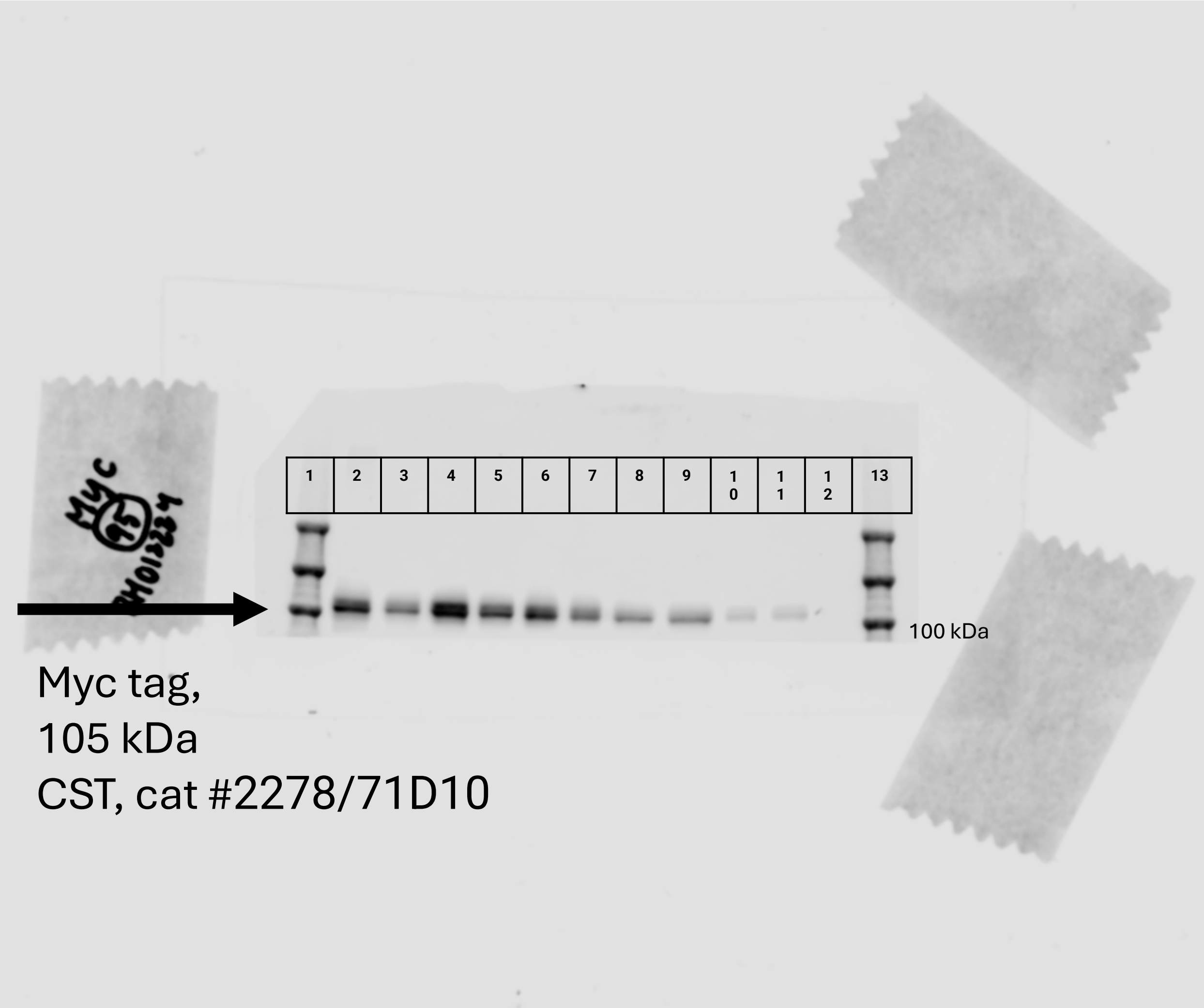

1	2	3	4	5	6	7	8	9	10	11	12	13
Ladder	NT5E $^{+/+}$ WCL	NT5E $^{-/-}$ WCL	NT5E $^{+/+}$ Cyto	NT5E $^{-/-}$ Cyto	NT5E $^{+/+}$ Mem	NT5E $^{-/-}$ Mem	NT5E $^{+/+}$ Nuc	NT5E $^{-/-}$ Nuc	NT5E $^{+/+}$ Chro	NT5E $^{-/-}$ Chro		Ladder

S4 B Images - Rab11a

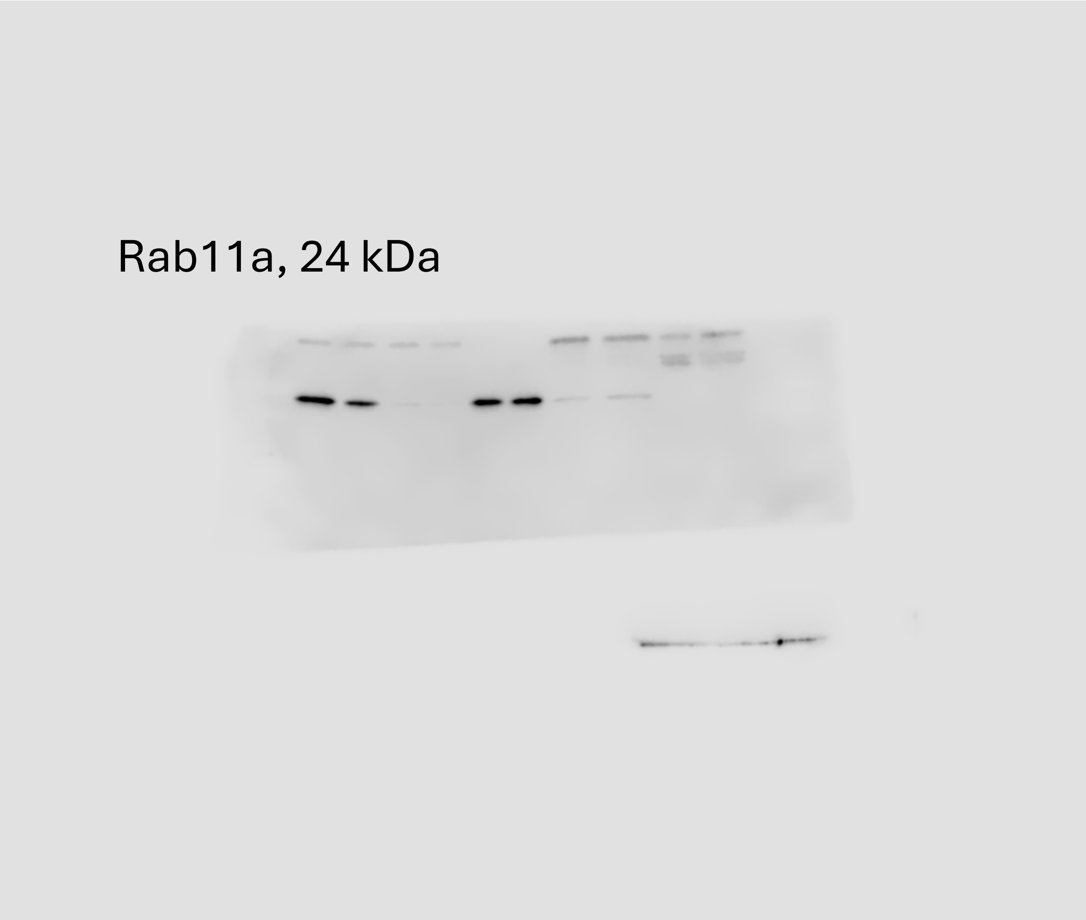

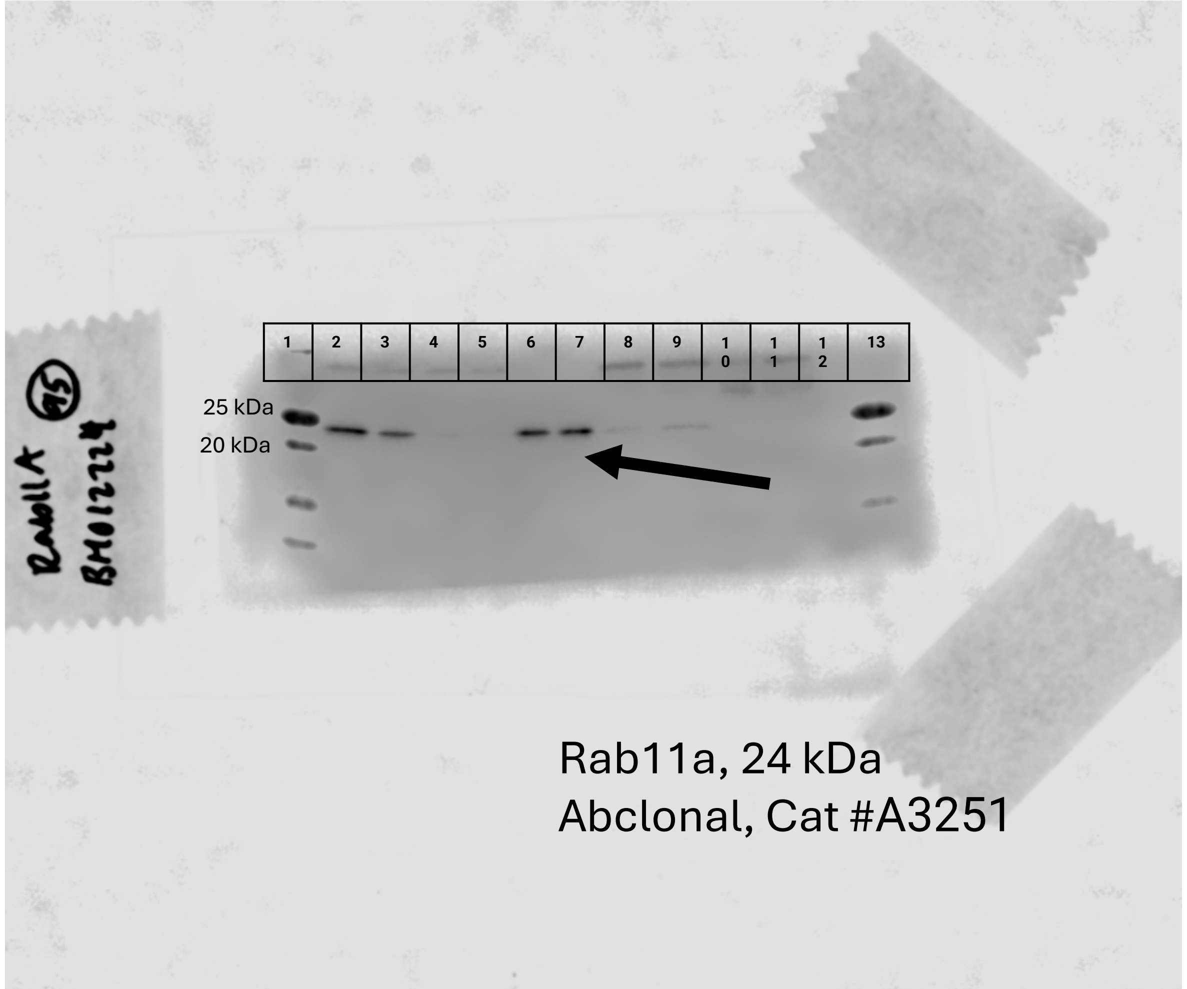

1	2	3	4	5	6	7	8	9	10	11	12	13
Ladder	NT5E $^{+/+}$ WCL	NT5E $^{-/-}$ WCL	NT5E $^{+/+}$ Cyto	NT5E $^{-/-}$ Cyto	NT5E $^{+/+}$ Mem	NT5E $^{-/-}$ Mem	NT5E $^{+/+}$ Nuc	NT5E $^{-/-}$ Nuc	NT5E $^{+/+}$ Chro	NT5E $^{-/-}$ Chro		Ladder

S4 B Images - SP1

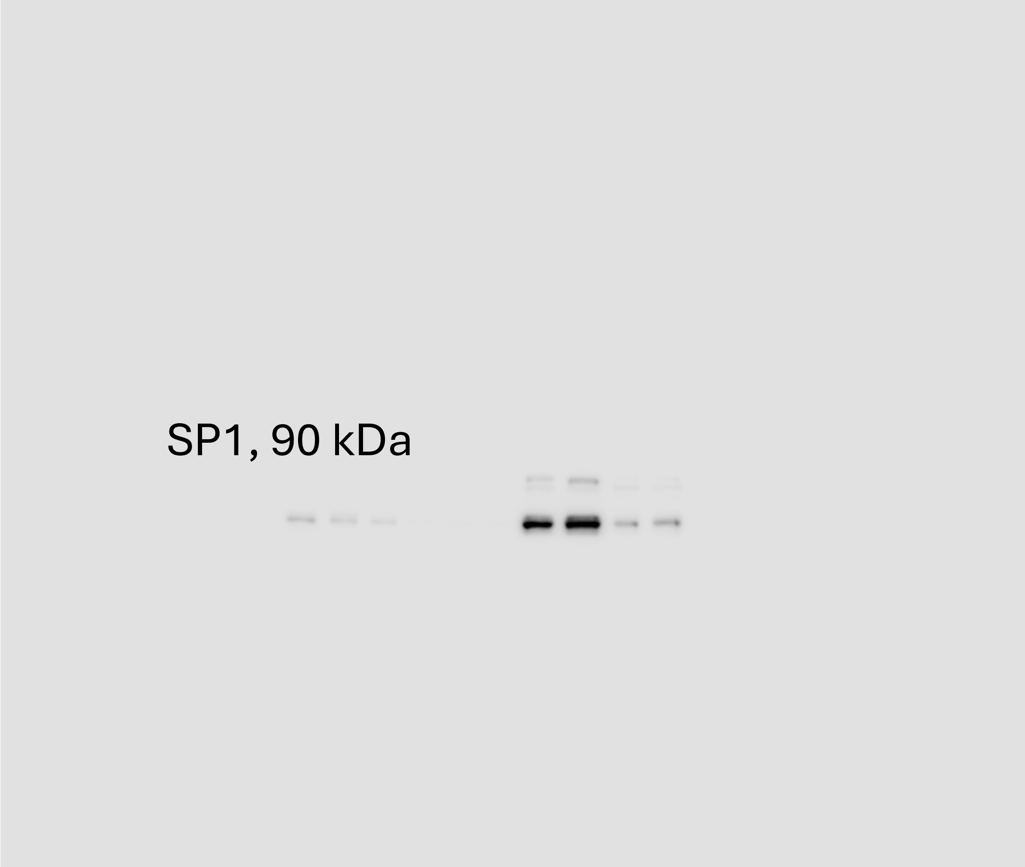

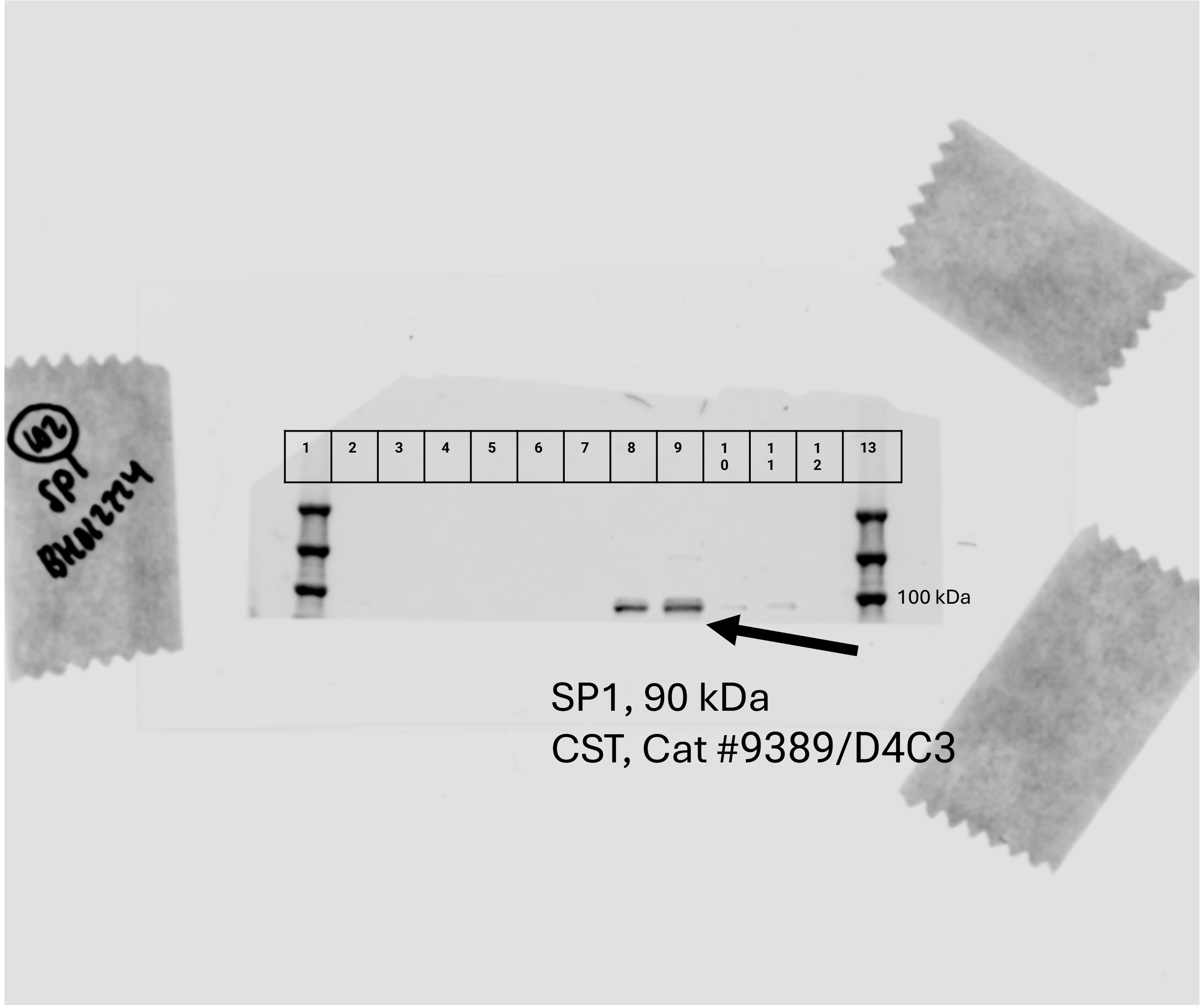

1	2	3	4	5	6	7	8	9	10	11	12	13
Ladder	NT5E $^{+/+}$ WCL	NT5E $^{-/-}$ WCL	NT5E $^{+/+}$ Cyto	NT5E $^{-/-}$ Cyto	NT5E $^{+/+}$ Mem	NT5E $^{-/-}$ Mem	NT5E $^{+/+}$ Nuc	NT5E $^{-/-}$ Nuc	NT5E $^{+/+}$ Chro	NT5E $^{-/-}$ Chro		Ladder

S4 B Images - H2AX

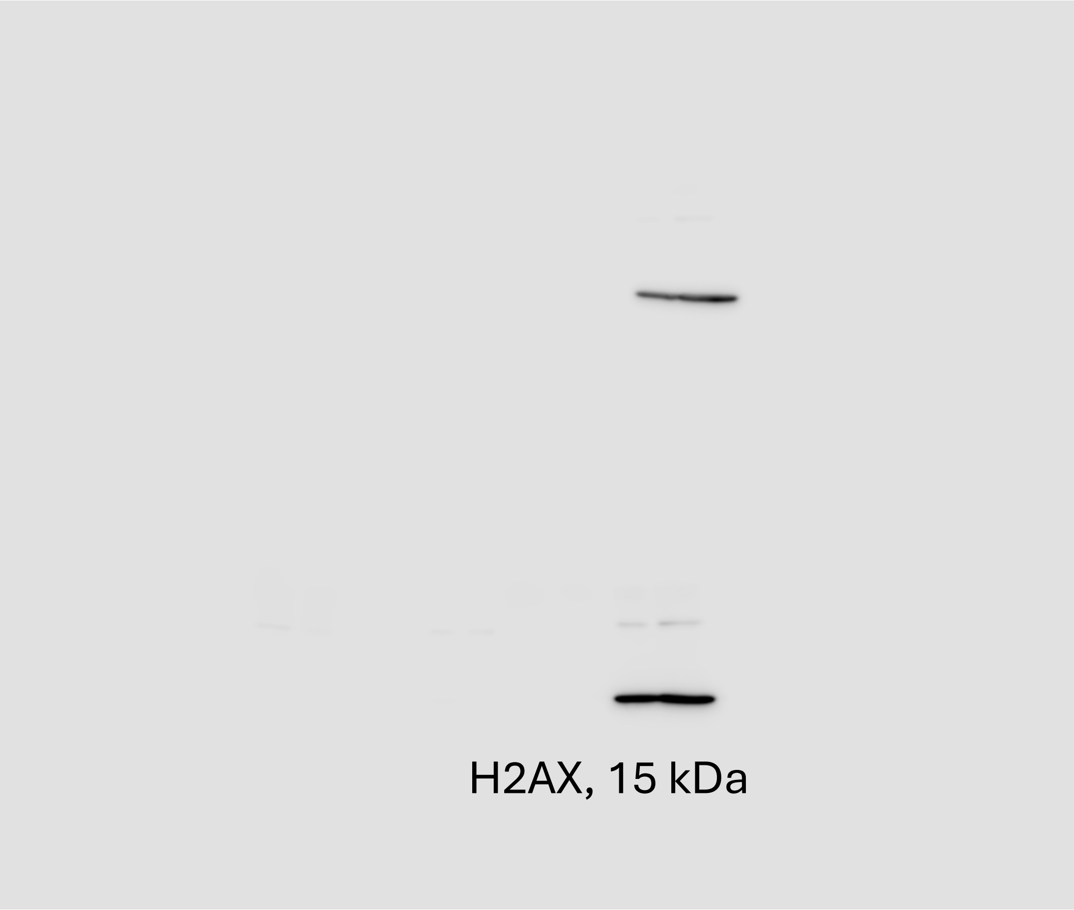

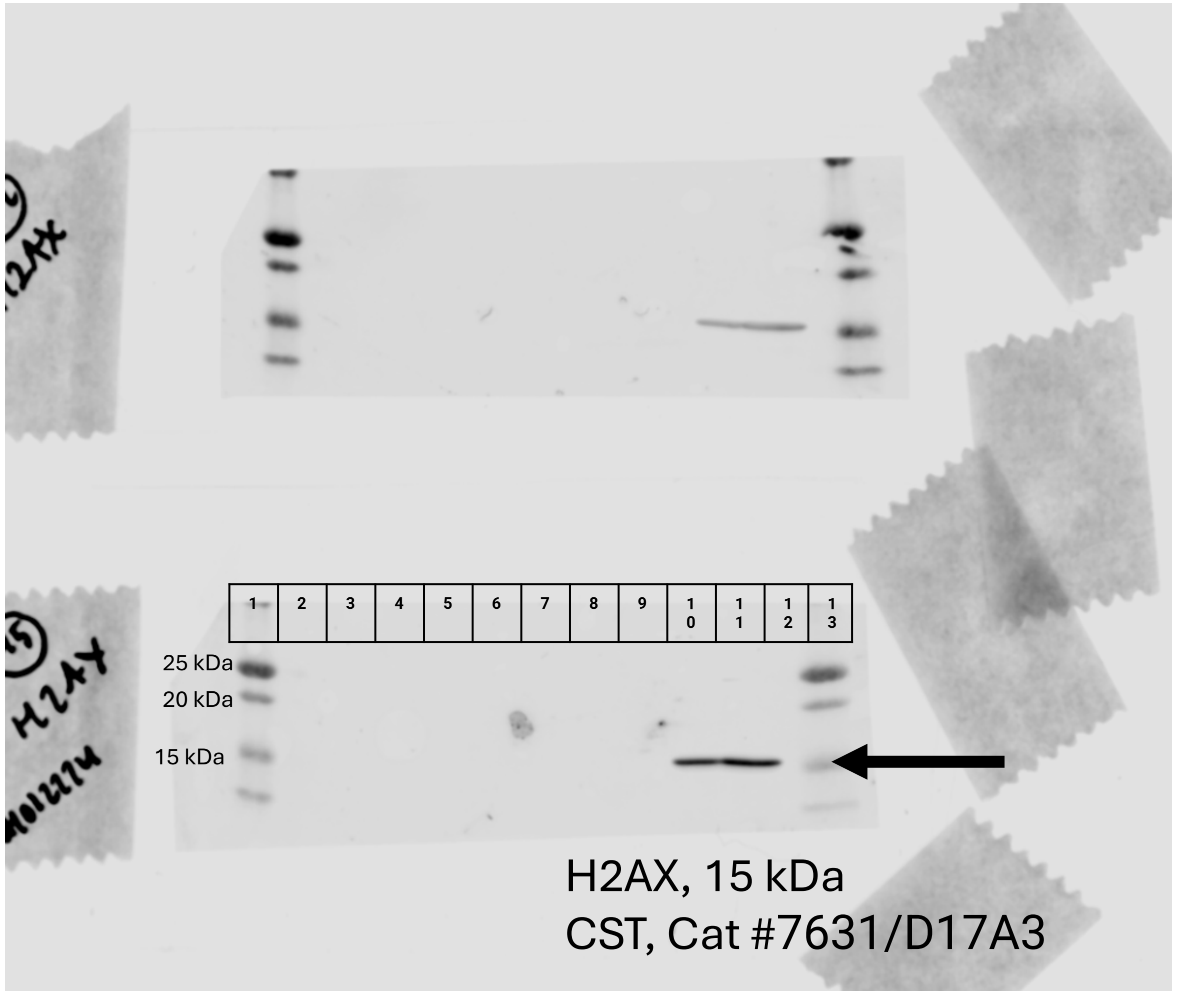

1	2	3	4	5	6	7	8	9	10	11	12	13
Ladder	NT5E +/+ WCL	NT5E -/- WCL	NT5E +/+ Cyto	NT5E -/- Cyto	NT5E +/+ Mem	NT5E -/- Mem	NT5E +/+ Nuc	NT5E -/- Nuc	NT5E +/+ Chro	NT5E -/- Chro		Ladder

S4 B Images

Total protein:
10% 2,2,2-trichloroethanol

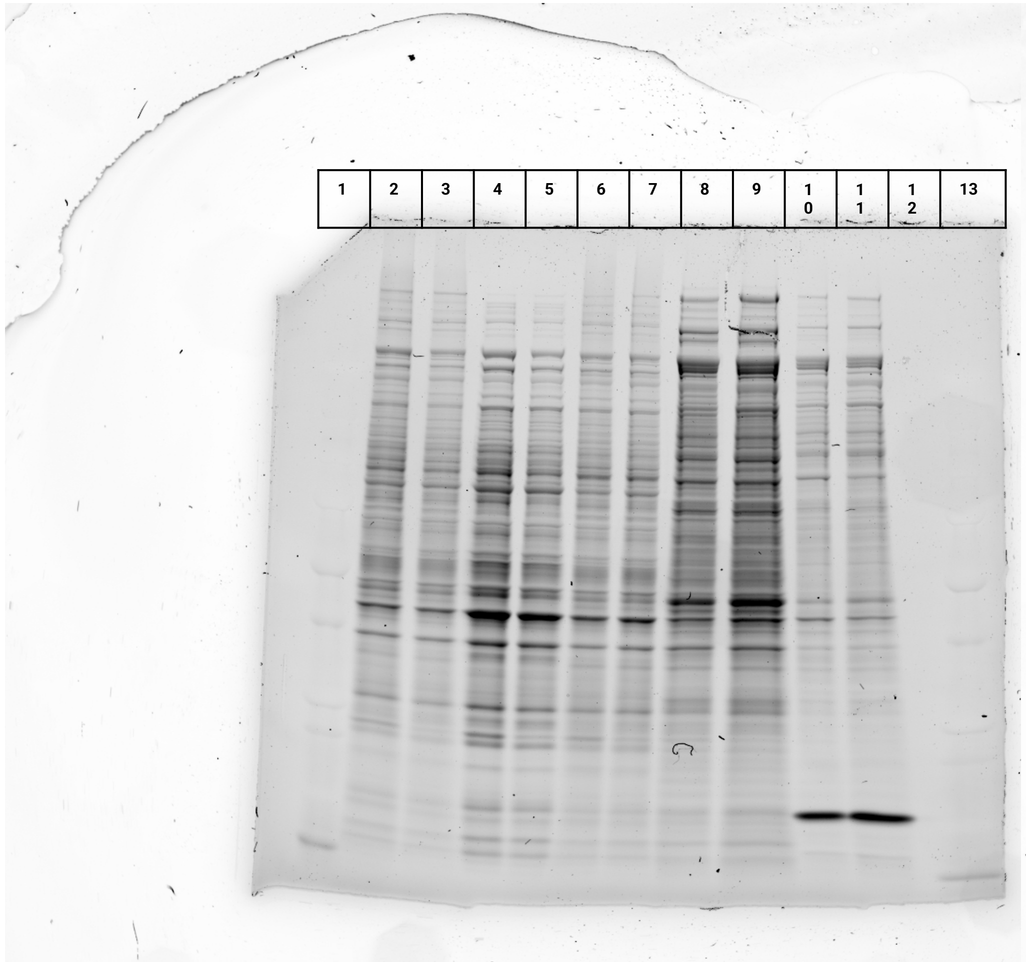

Imaged Myc, CD73, and Rab11a

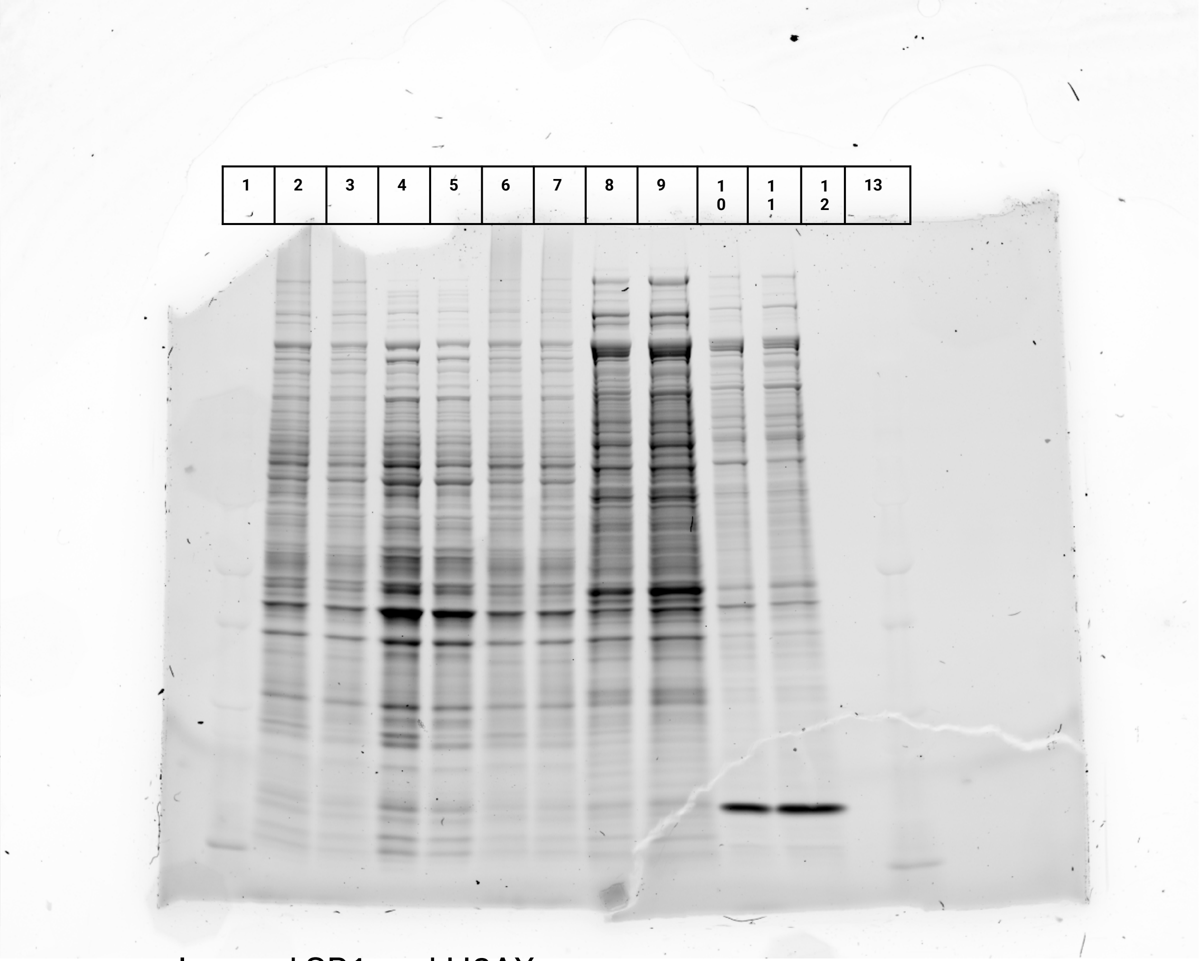

Imaged SP1 and H2AX

1	2	3	4	5	6	7	8	9	10	11	12	13
Ladder	NT5E $^{+/+}$ WCL	NT5E $^{-/-}$ WCL	NT5E $^{+/+}$ Cyto	NT5E $^{-/-}$ Cyto	NT5E $^{+/+}$ Mem	NT5E $^{-/-}$ Mem	NT5E $^{+/+}$ Nuc	NT5E $^{-/-}$ Nuc	NT5E $^{+/+}$ Chro	NT5E $^{-/-}$ Chro		Ladder

S4 C images - Myc

Myc tag, short exp,
105 kDa

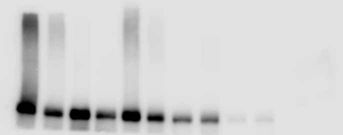

Myc tag, long exp,
105 kDa

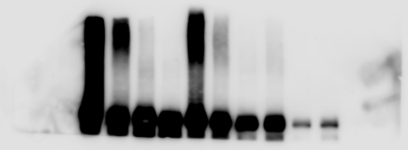

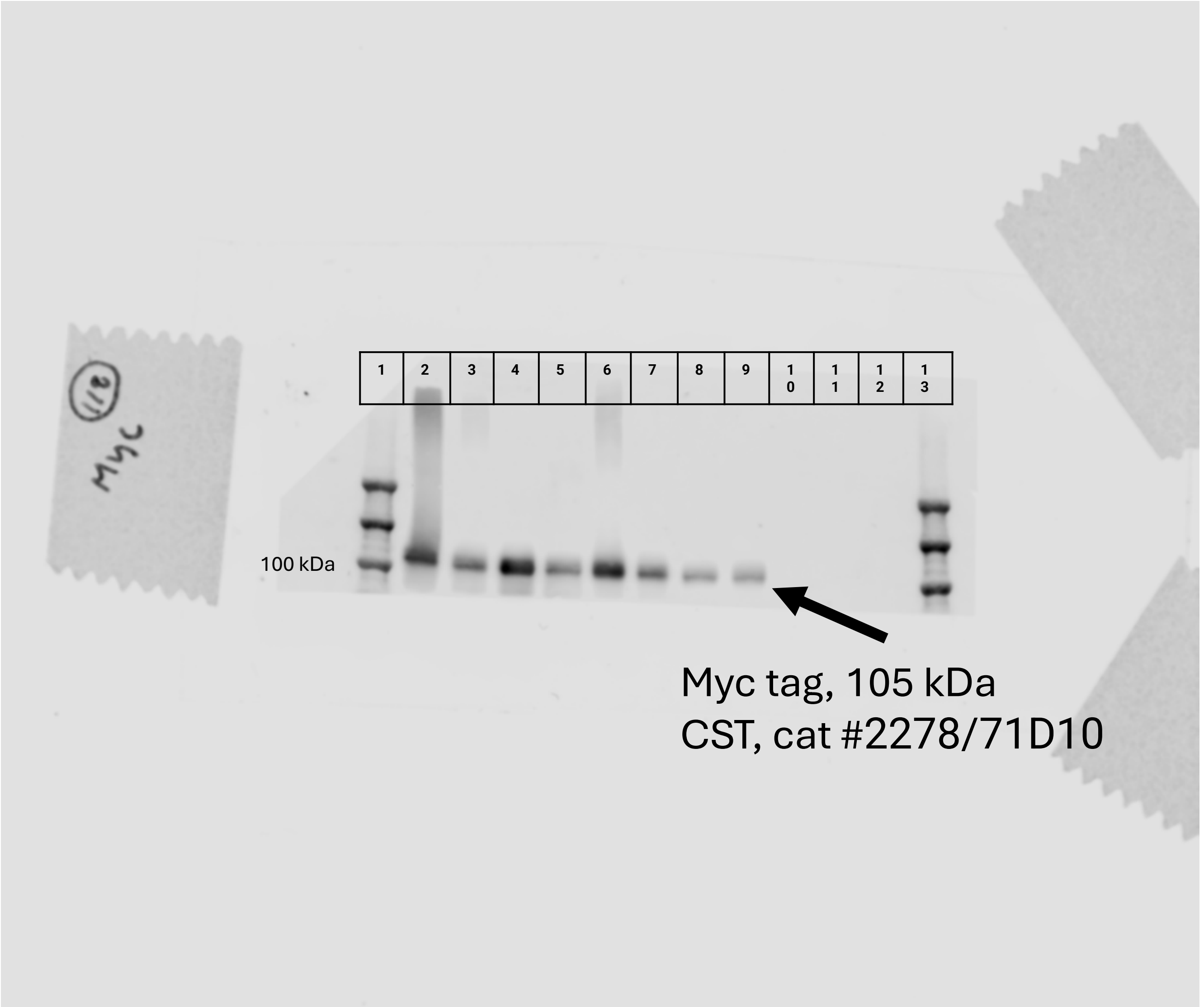

1	2	3	4	5	6	7	8	9	10	11	12	13
Ladder	NT5E +/+ WCL	NT5E -/- WCL	NT5E +/+ Cyto	NT5E -/- Cyto	NT5E +/+ Mem	NT5E -/- Mem	NT5E +/+ Nuc	NT5E -/- Nuc	NT5E +/+ Chro	NT5E -/- Chro		Ladder

S4 C images - Rab11

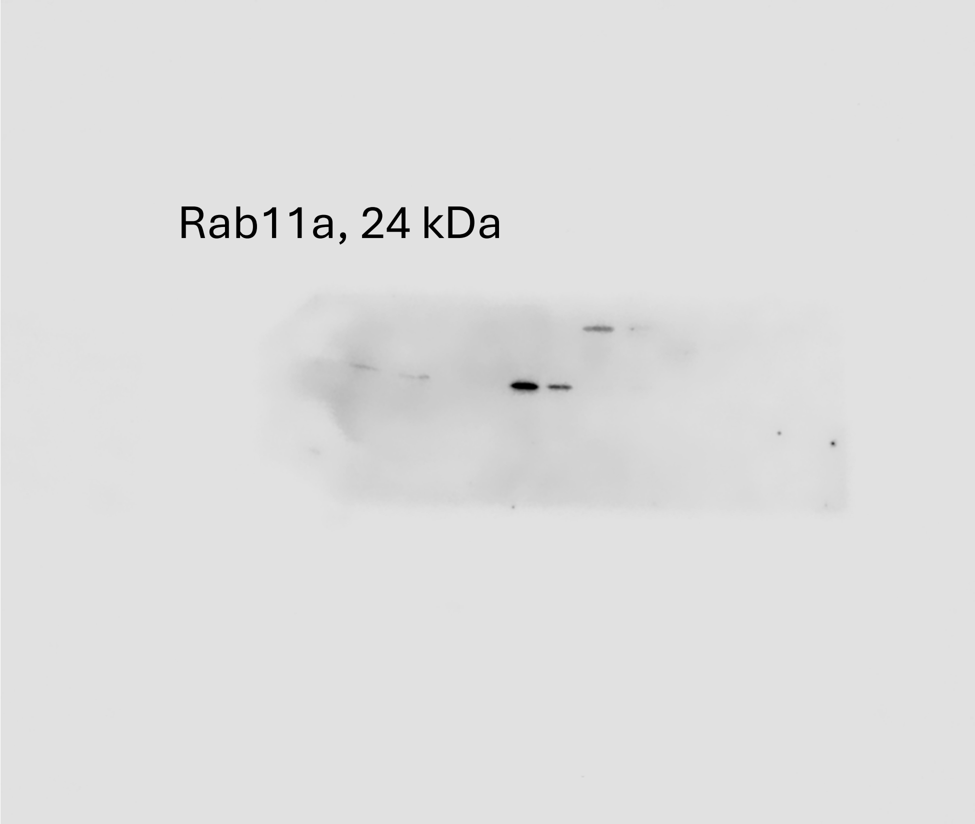

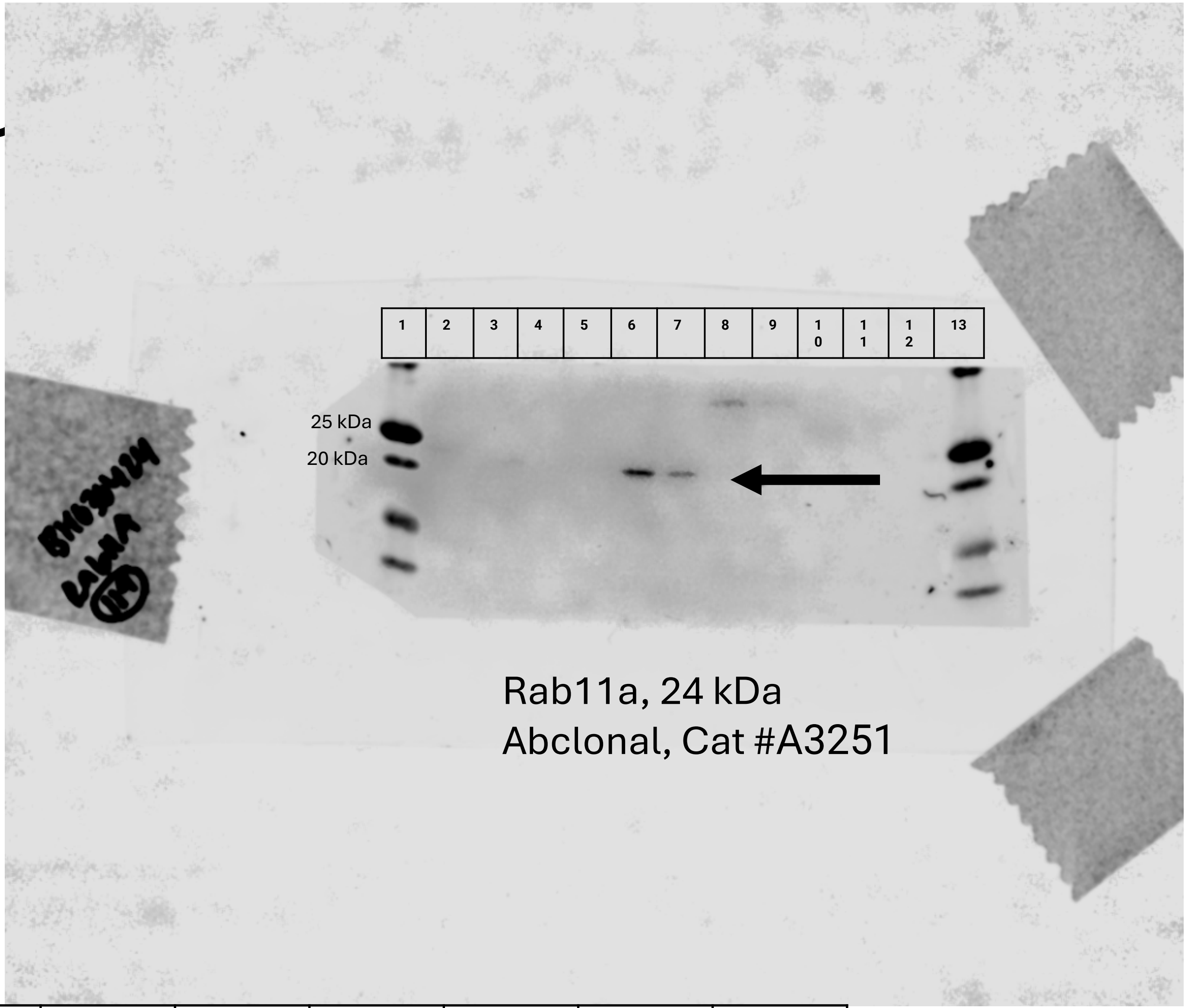

1	2	3	4	5	6	7	8	9	10	11	12	13
Ladder	NT5E $^{+/+}$ WCL	NT5E $^{-/-}$ WCL	NT5E $^{+/+}$ Cyto	NT5E $^{-/-}$ Cyto	NT5E $^{+/+}$ Mem	NT5E $^{-/-}$ Mem	NT5E $^{+/+}$ Nuc	NT5E $^{-/-}$ Nuc	NT5E $^{+/+}$ Chro	NT5E $^{-/-}$ Chro		Ladder

S4 C images - SP1

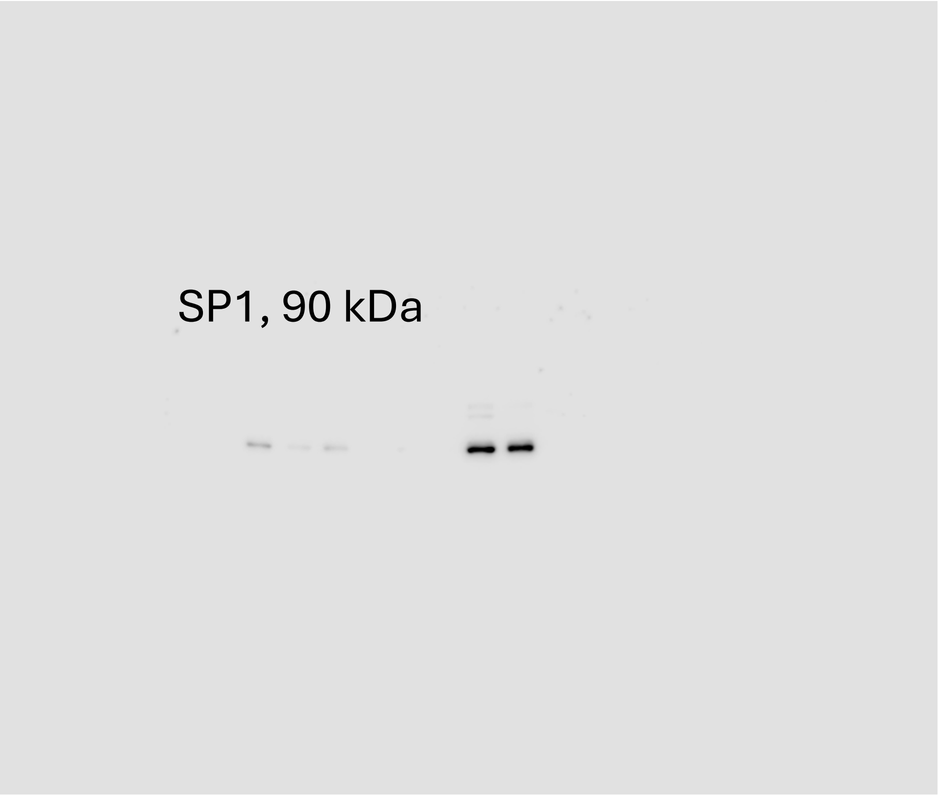

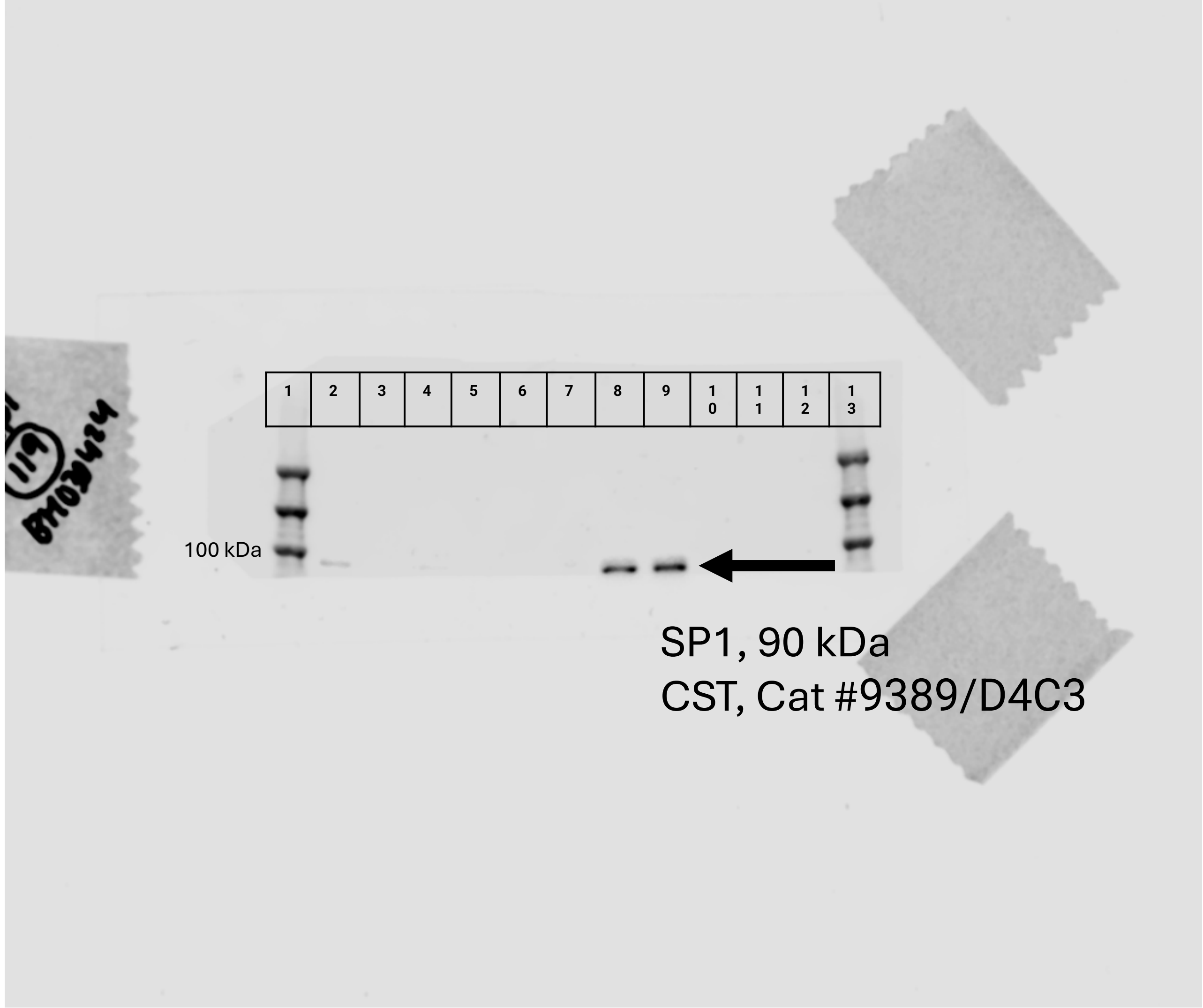

1	2	3	4	5	6	7	8	9	10	11	12	13
Ladder	NT5E $^{+/+}$ WCL	NT5E $^{-/-}$ WCL	NT5E $^{+/+}$ Cyto	NT5E $^{-/-}$ Cyto	NT5E $^{+/+}$ Mem	NT5E $^{-/-}$ Mem	NT5E $^{+/+}$ Nuc	NT5E $^{-/-}$ Nuc	NT5E $^{+/+}$ Chro	NT5E $^{-/-}$ Chro		Ladder

S4 C images - H2AX

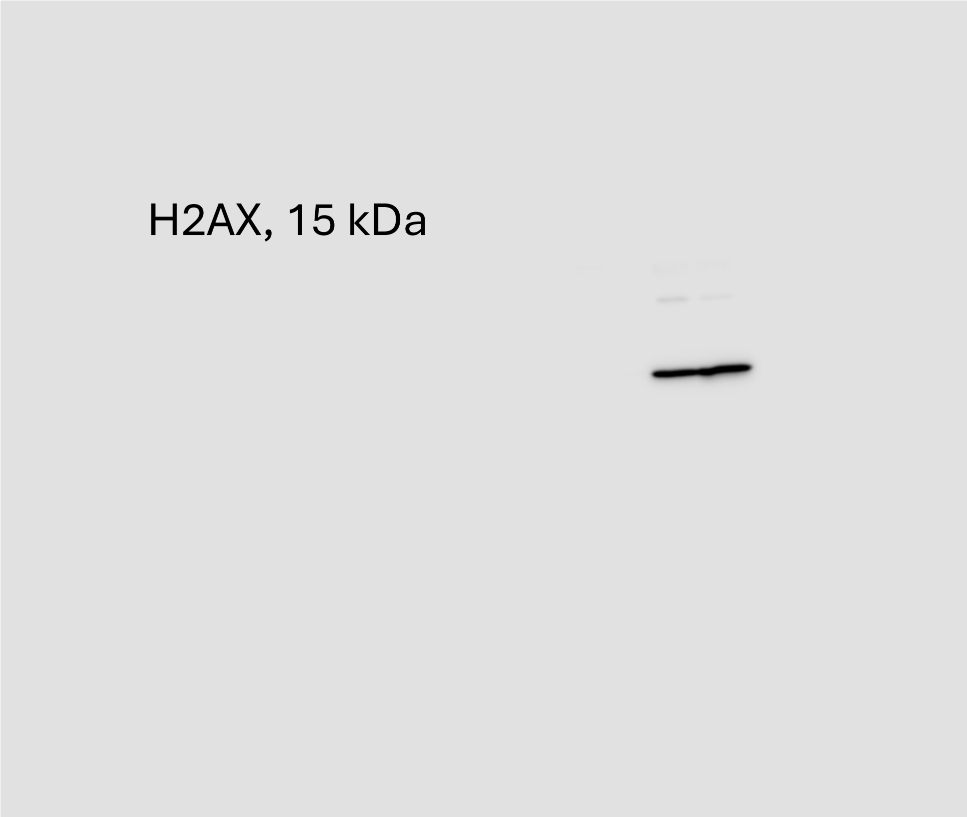

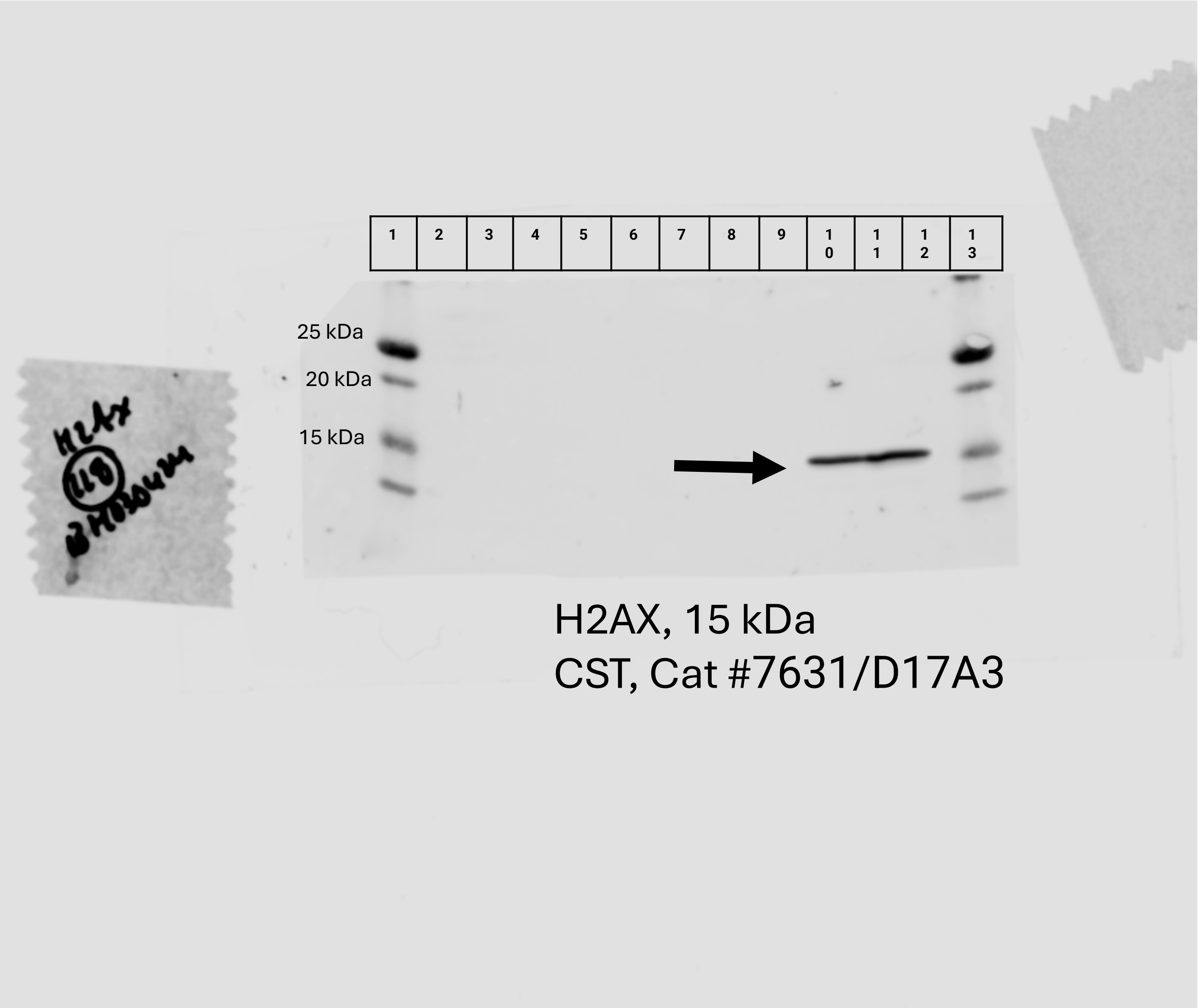

1	2	3	4	5	6	7	8	9	10	11	12	13
Ladder	NT5E $^{+/+}$ WCL	NT5E $^{-/-}$ WCL	NT5E $^{+/+}$ Cyto	NT5E $^{-/-}$ Cyto	NT5E $^{+/+}$ Mem	NT5E $^{-/-}$ Mem	NT5E $^{+/+}$ Nuc	NT5E $^{-/-}$ Nuc	NT5E $^{+/+}$ Chro	NT5E $^{-/-}$ Chro		Ladder

S4 C images

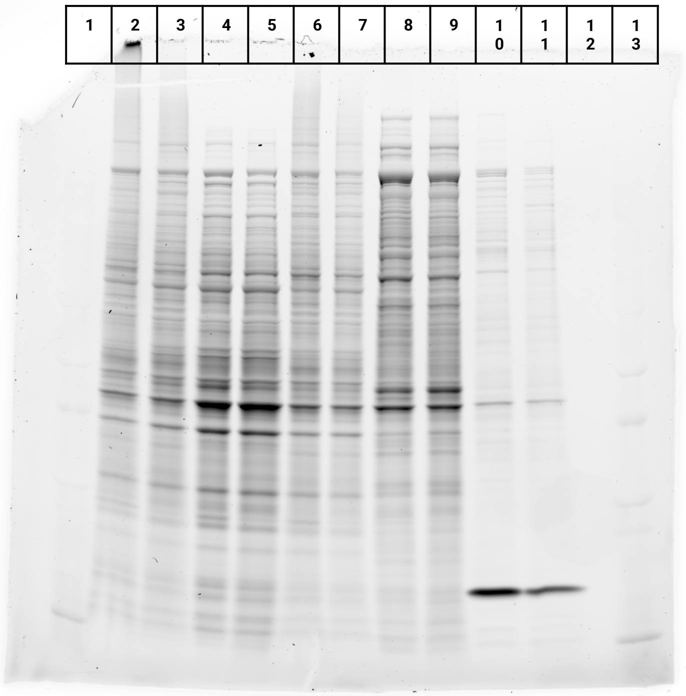

Imaged Myc, CD73, and Rab11a

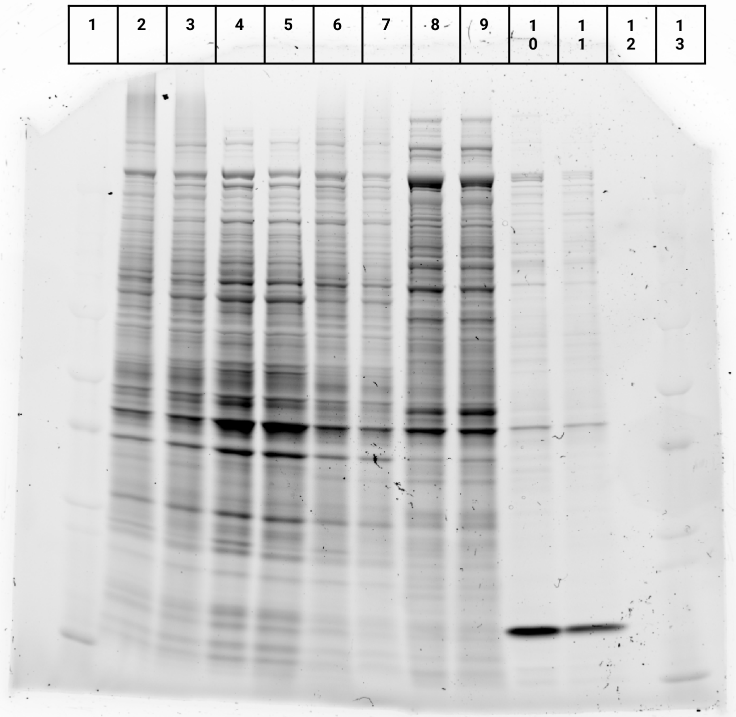

Imaged SP1, Akt, and H2AX

Total protein:
10% 2,2,2-trichloroethanol

1	2	3	4	5	6	7	8	9	10	11	12	13
Ladder	NT5E $^{+/+}$ WCL	NT5E $^{-/-}$ WCL	NT5E $^{+/+}$ Cyto	NT5E $^{-/-}$ Cyto	NT5E $^{+/+}$ Mem	NT5E $^{-/-}$ Mem	NT5E $^{+/+}$ Nuc	NT5E $^{-/-}$ Nuc	NT5E $^{+/+}$ Chro	NT5E $^{-/-}$ Chro		Ladder

S4 D Images - Myc

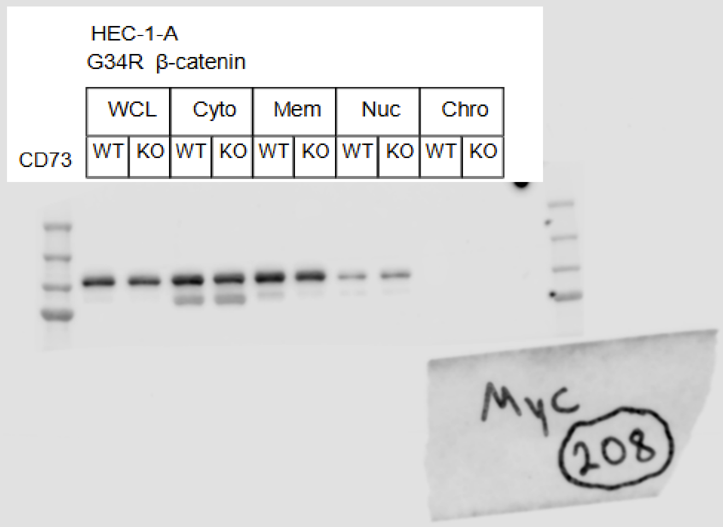

Short exposure

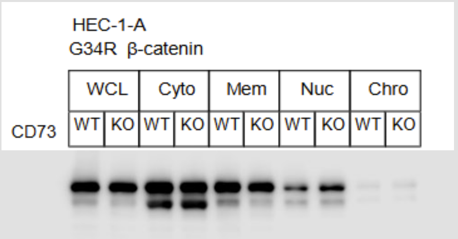

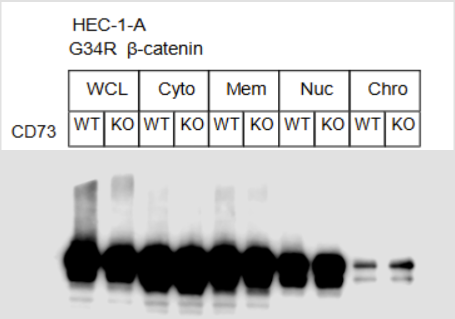

Long exposure

S4 D Images – Rab11

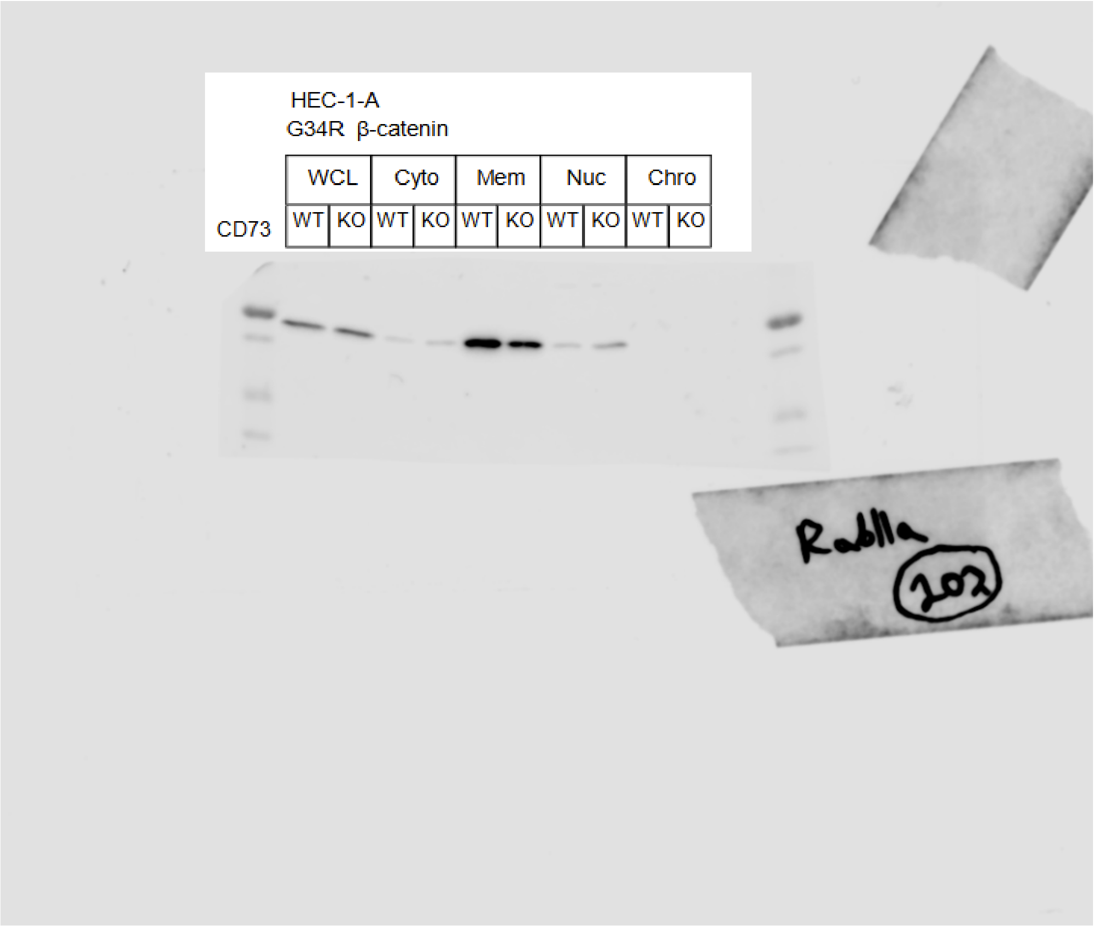

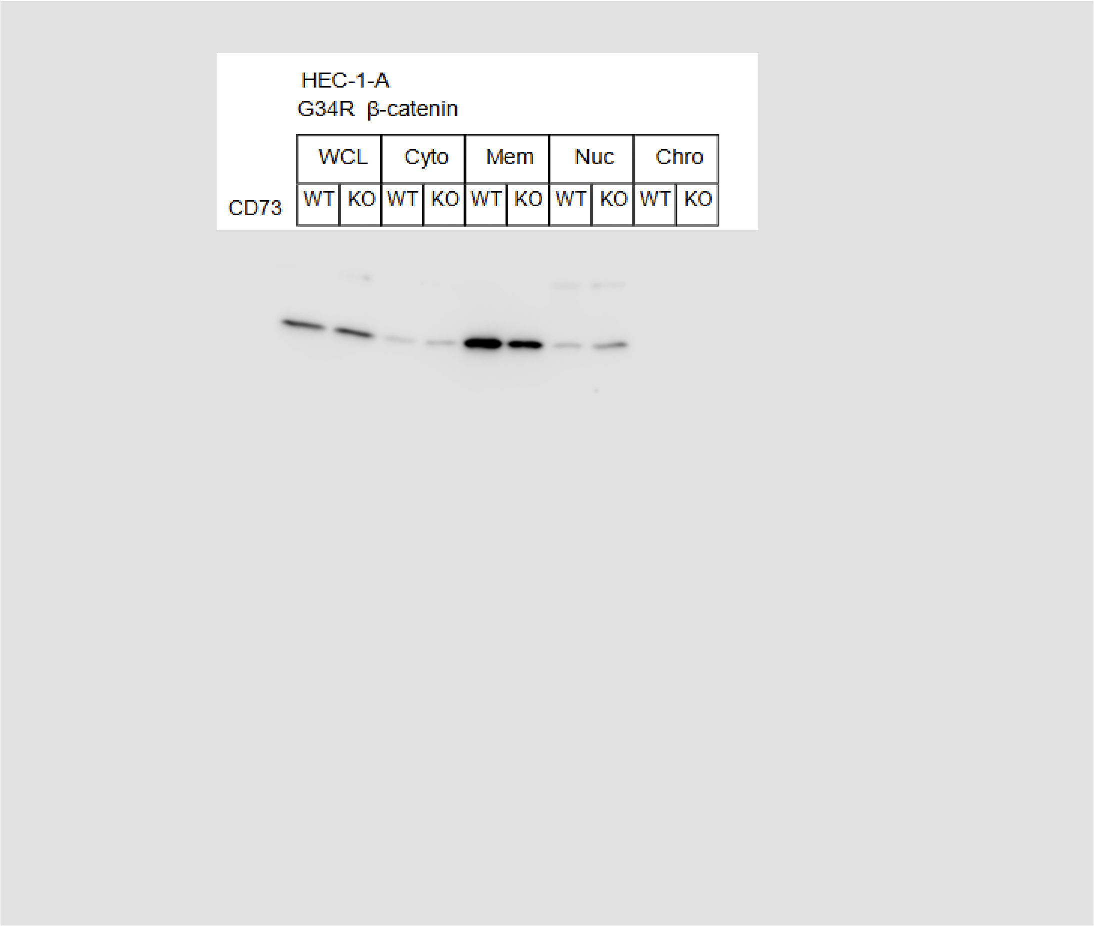

S4 D Images – SP1

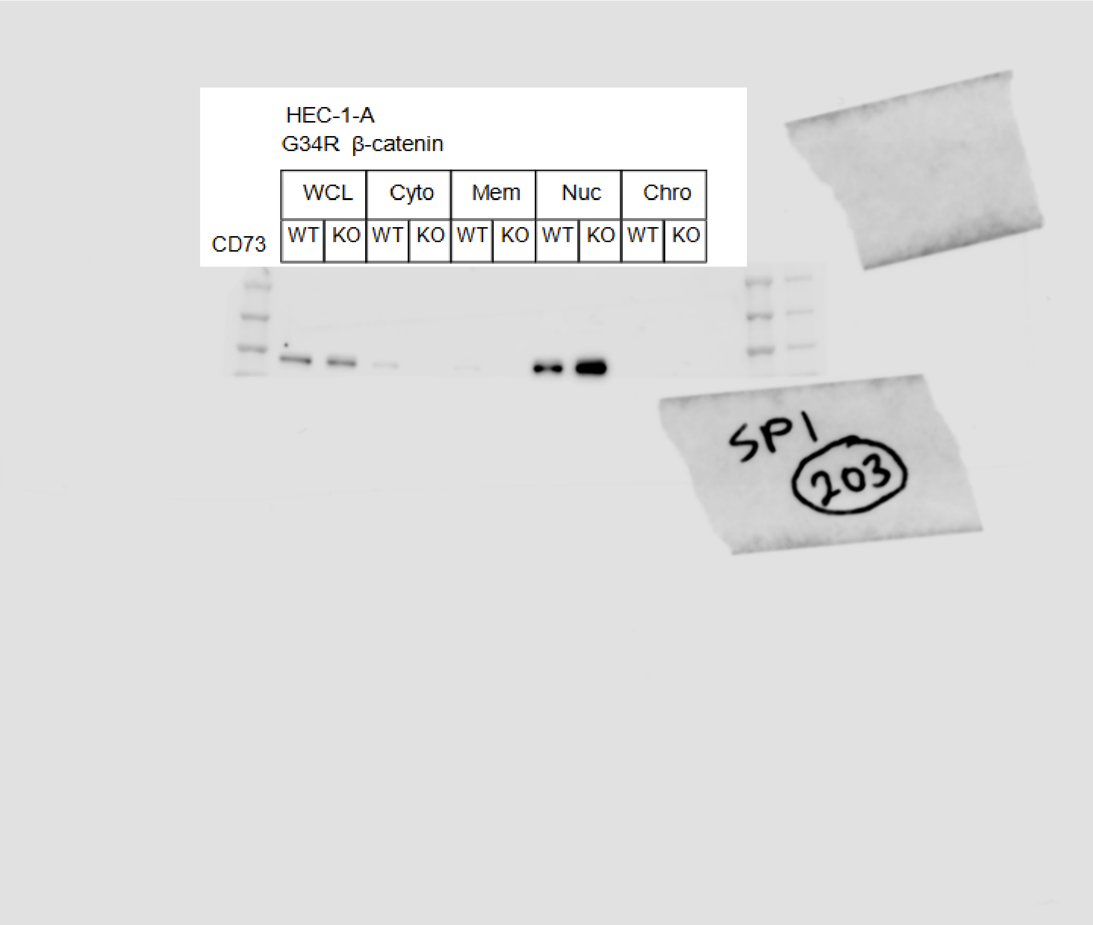

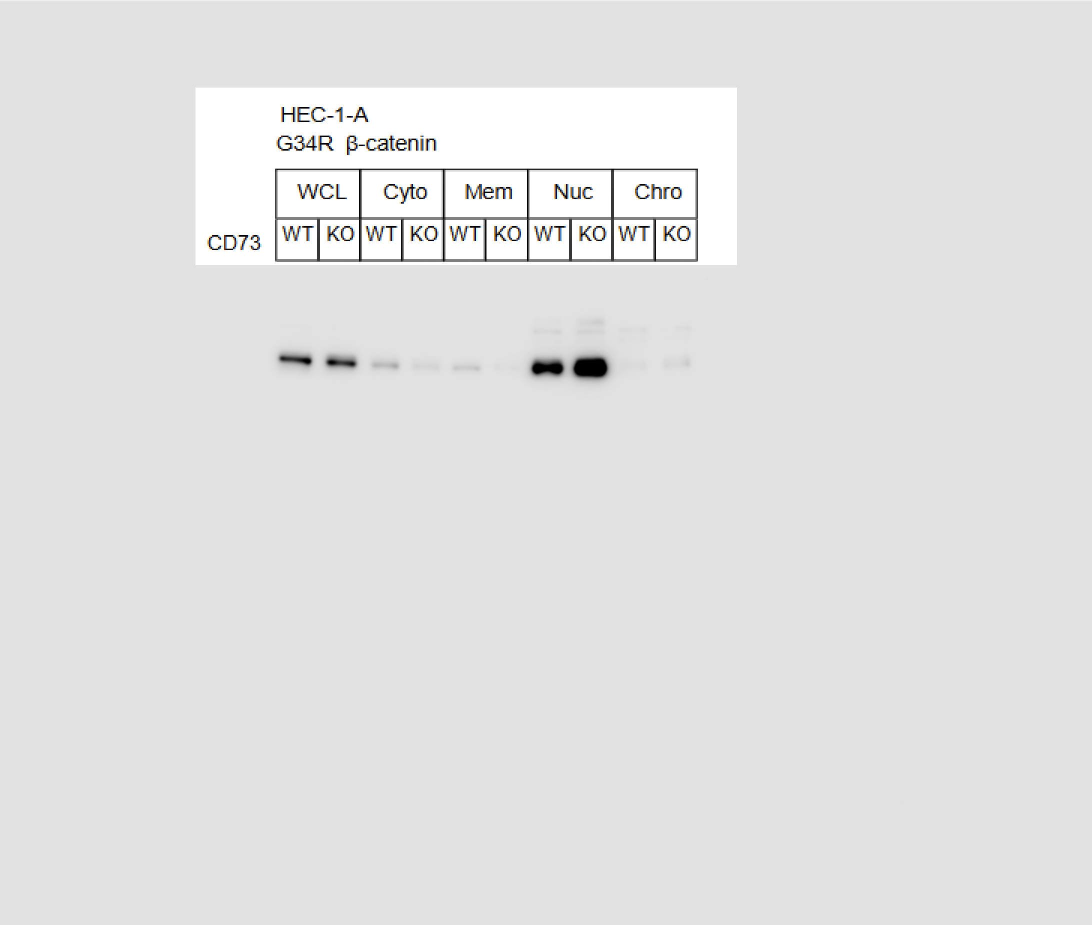

S4 D Images – H2AX

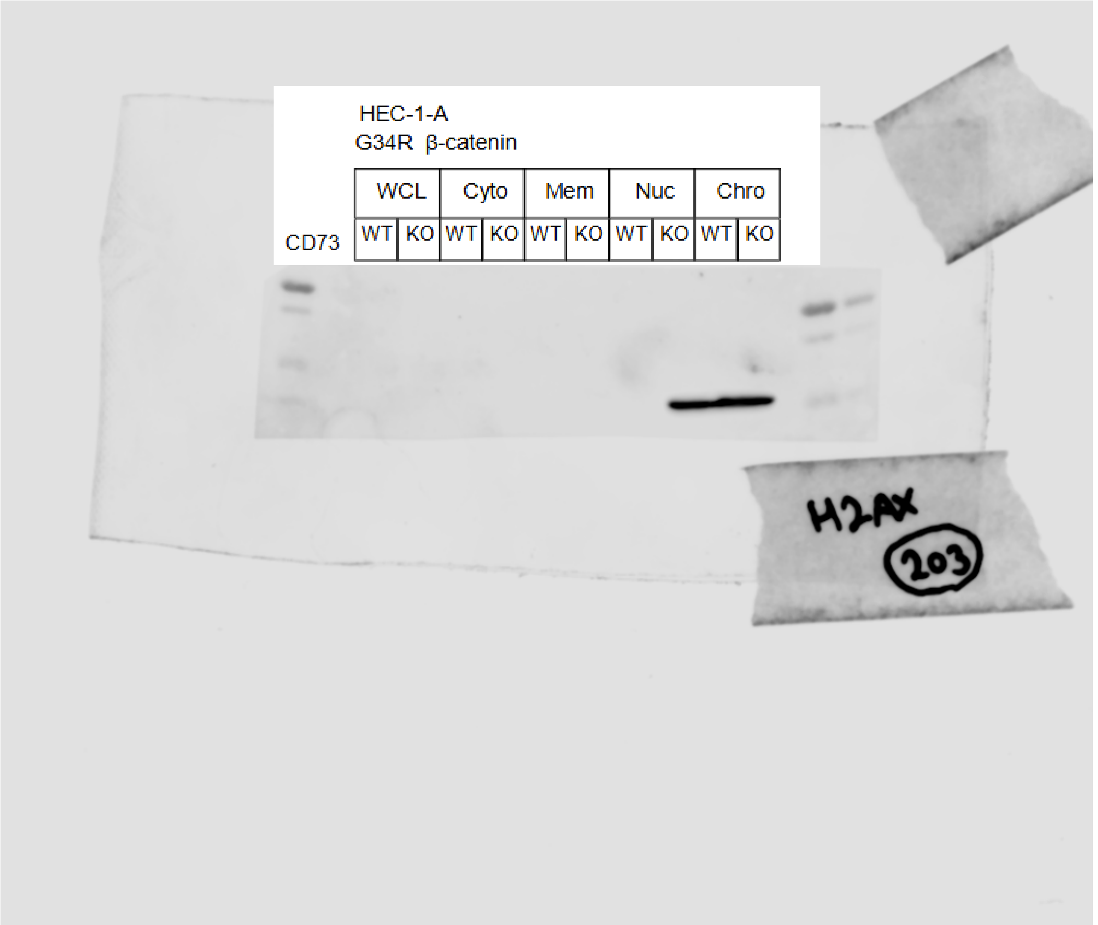

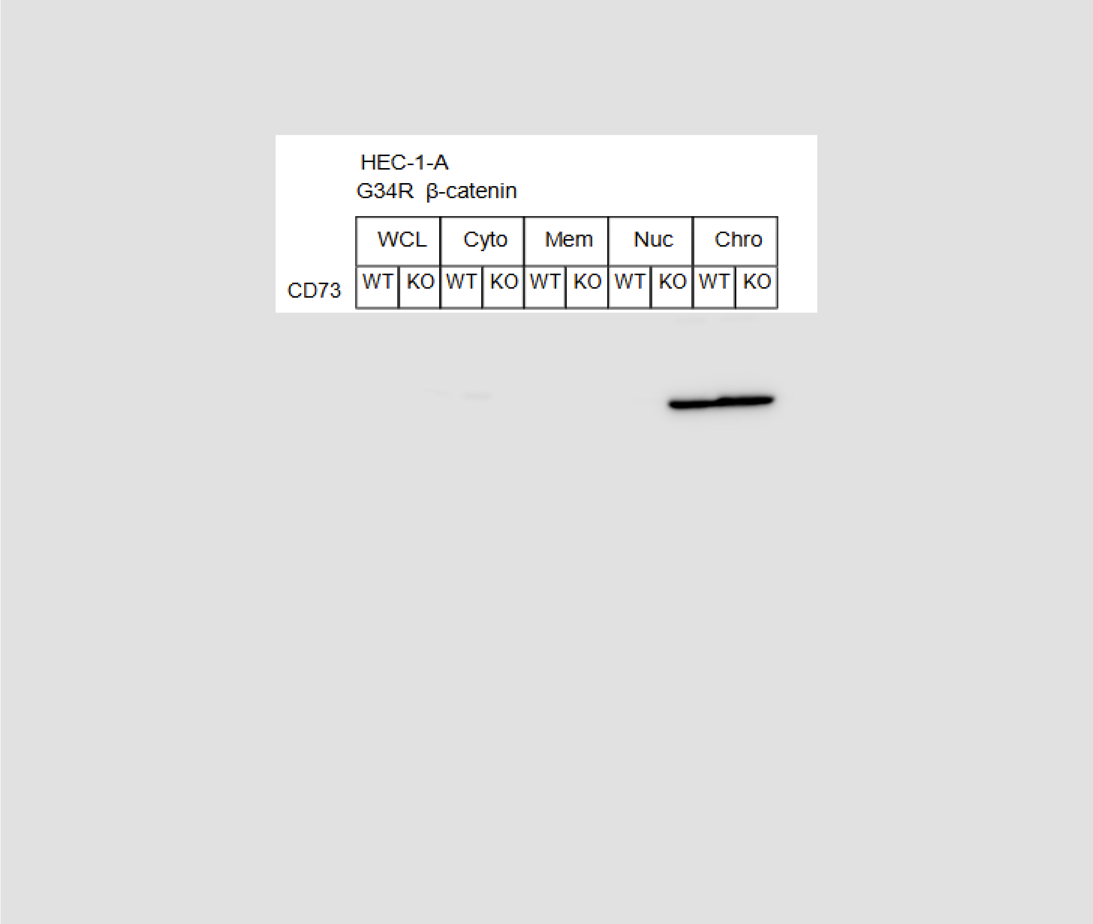

S4 D Images – Total Protein

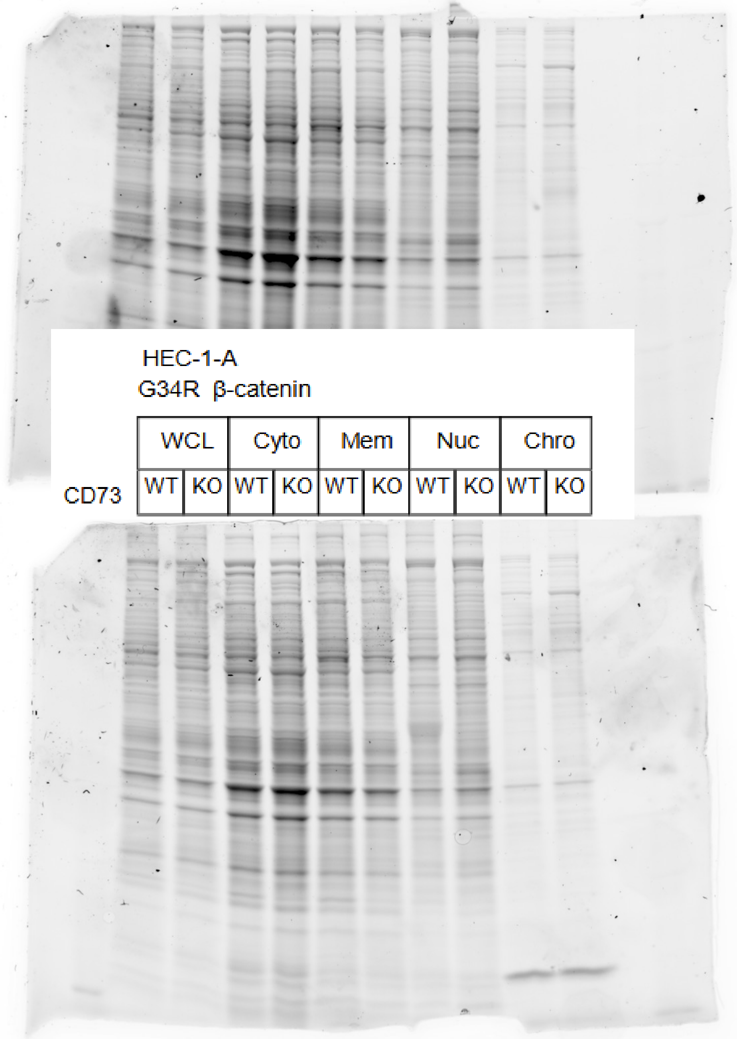

S4 E Images - Myc

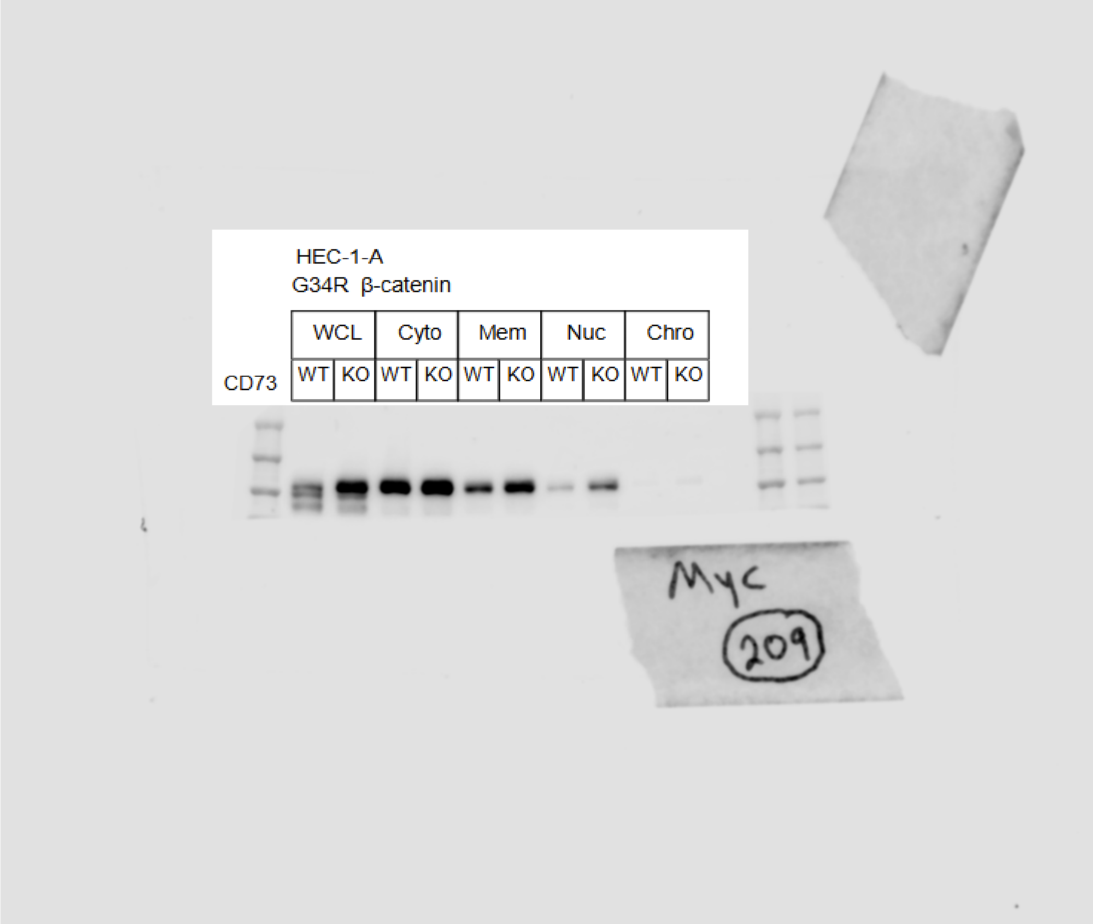

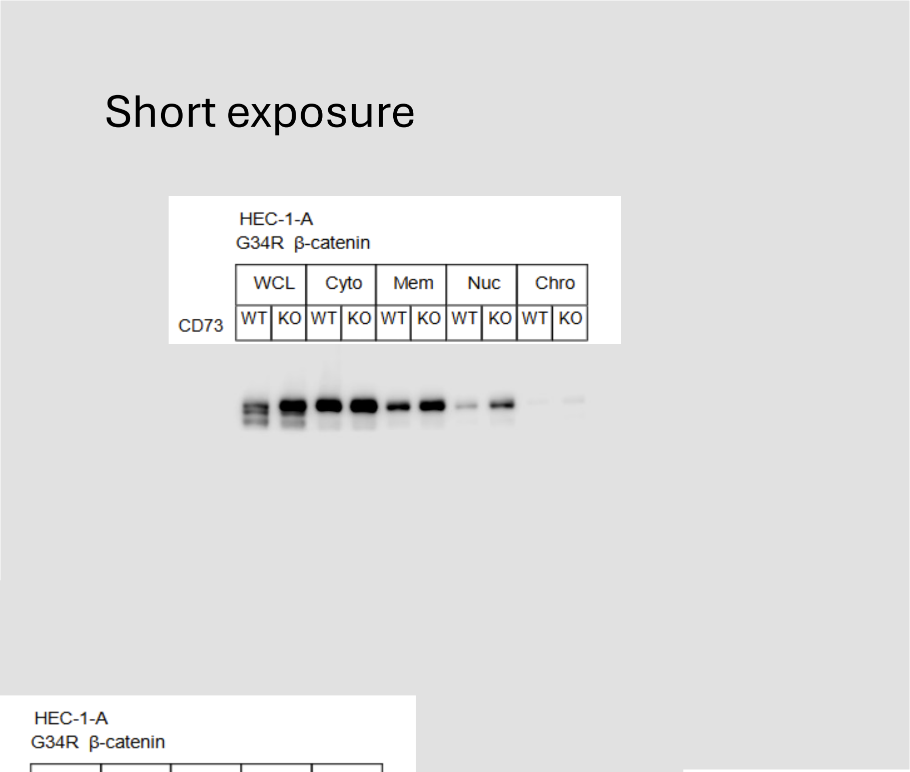

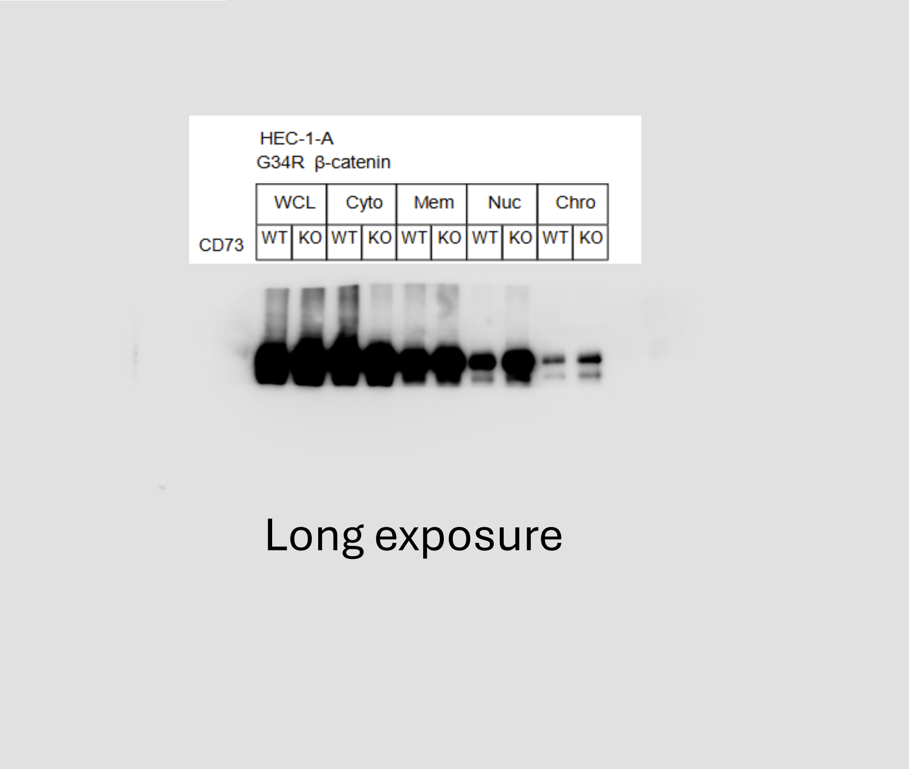

S4 E Images – Rab11

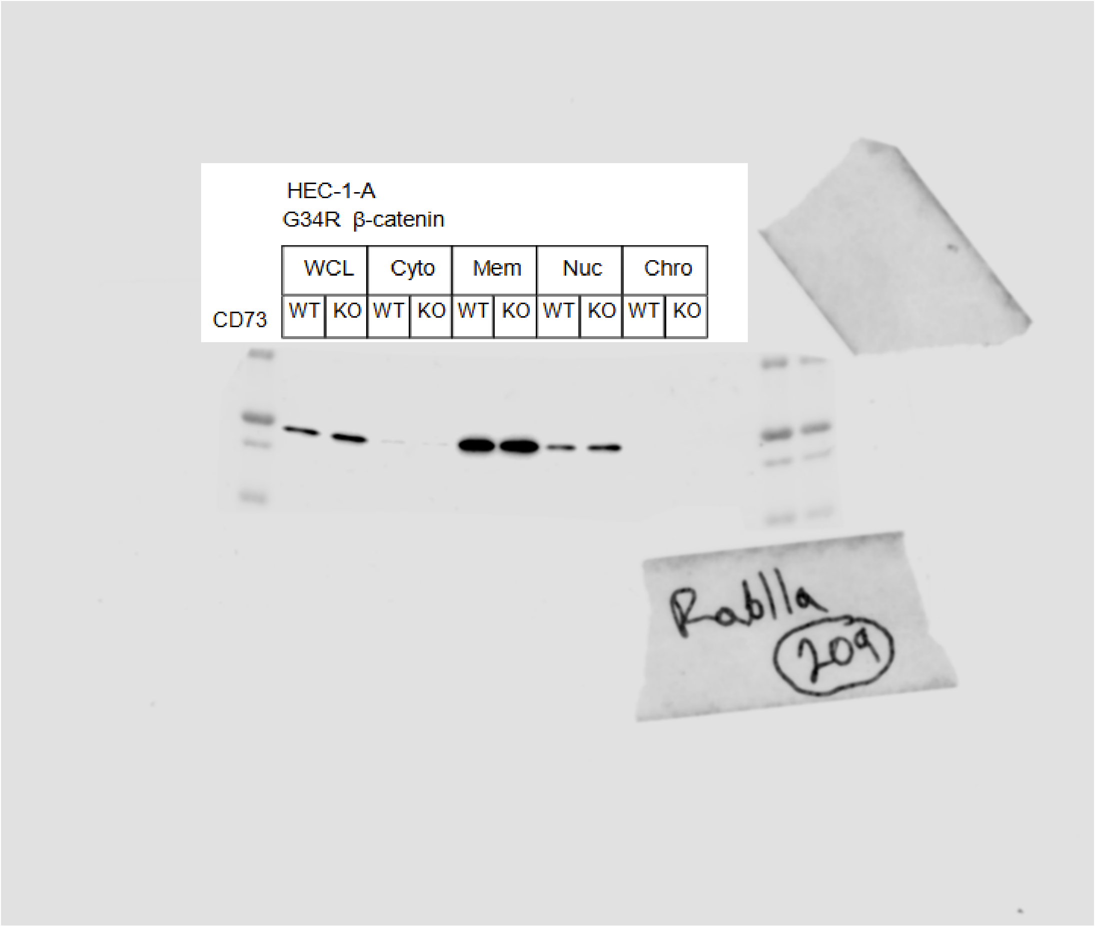

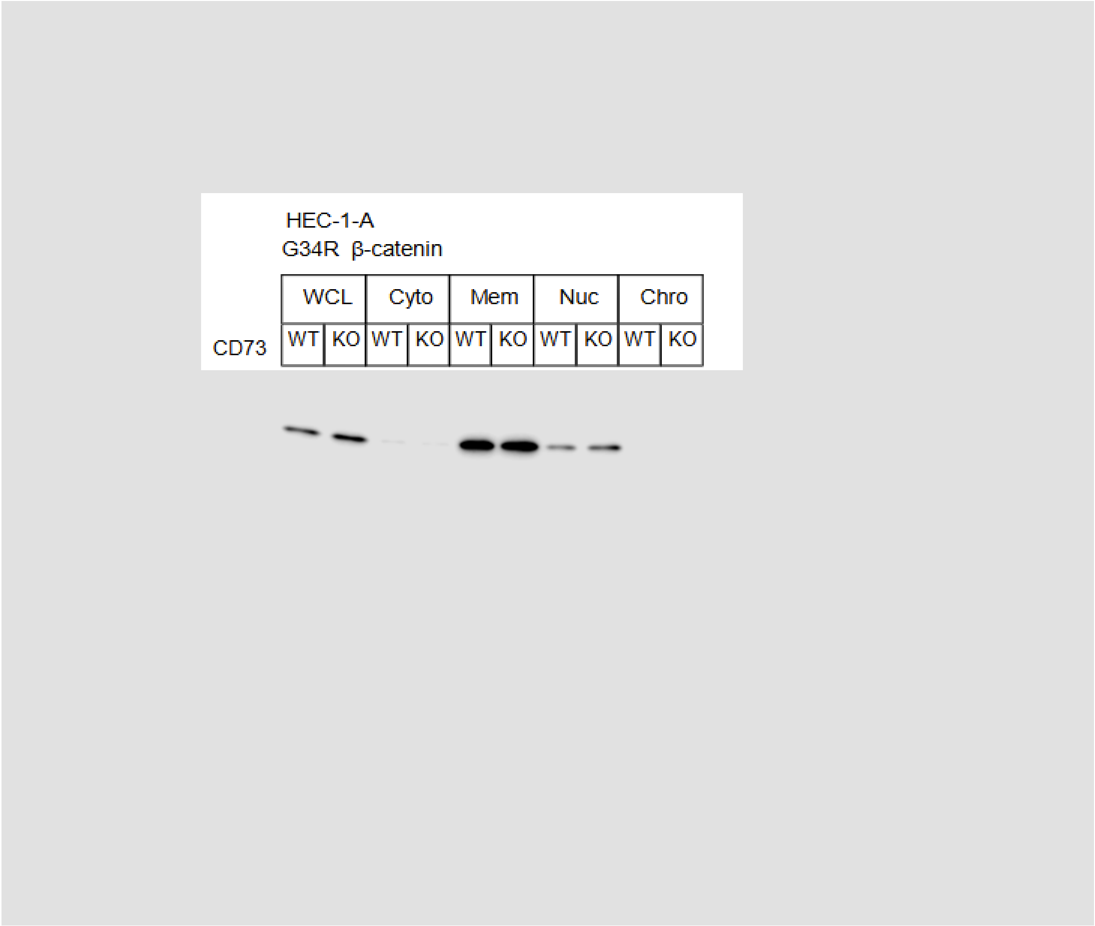

S4 E Images – SP1

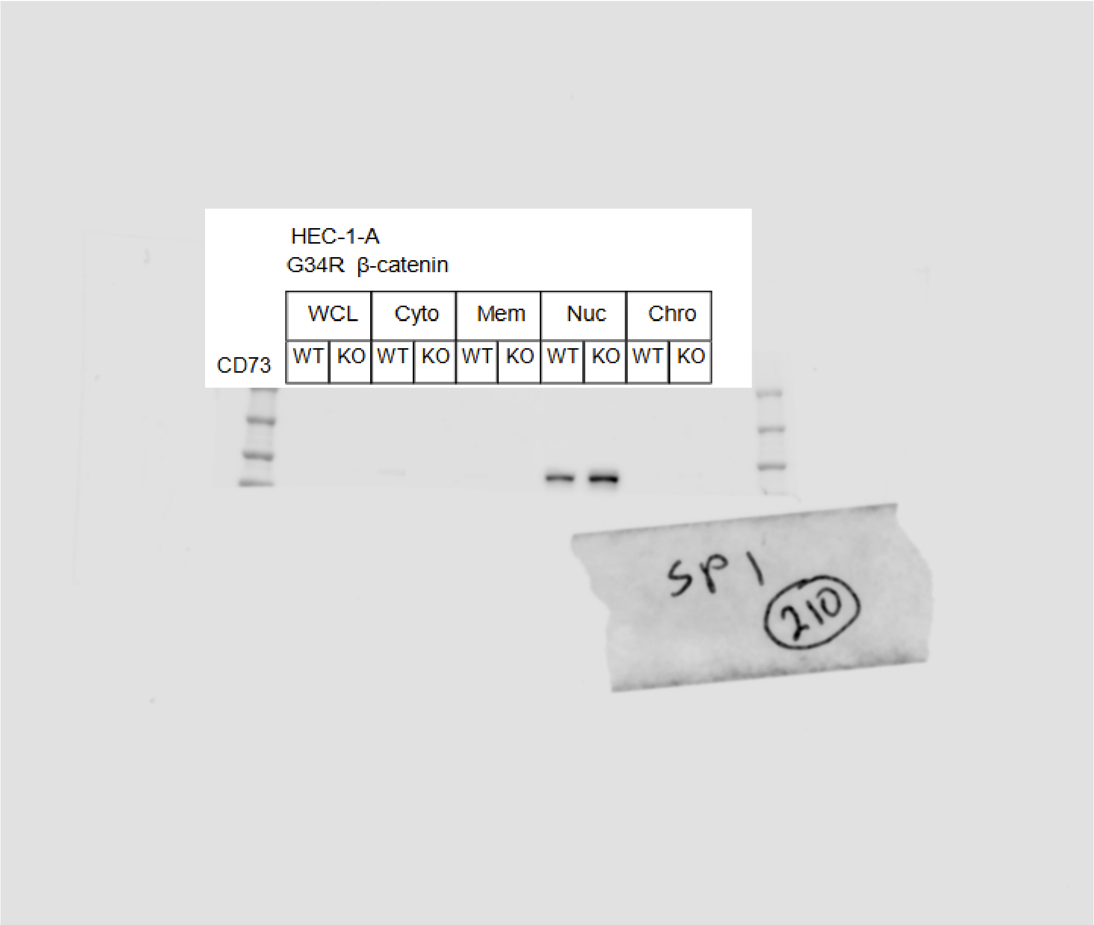

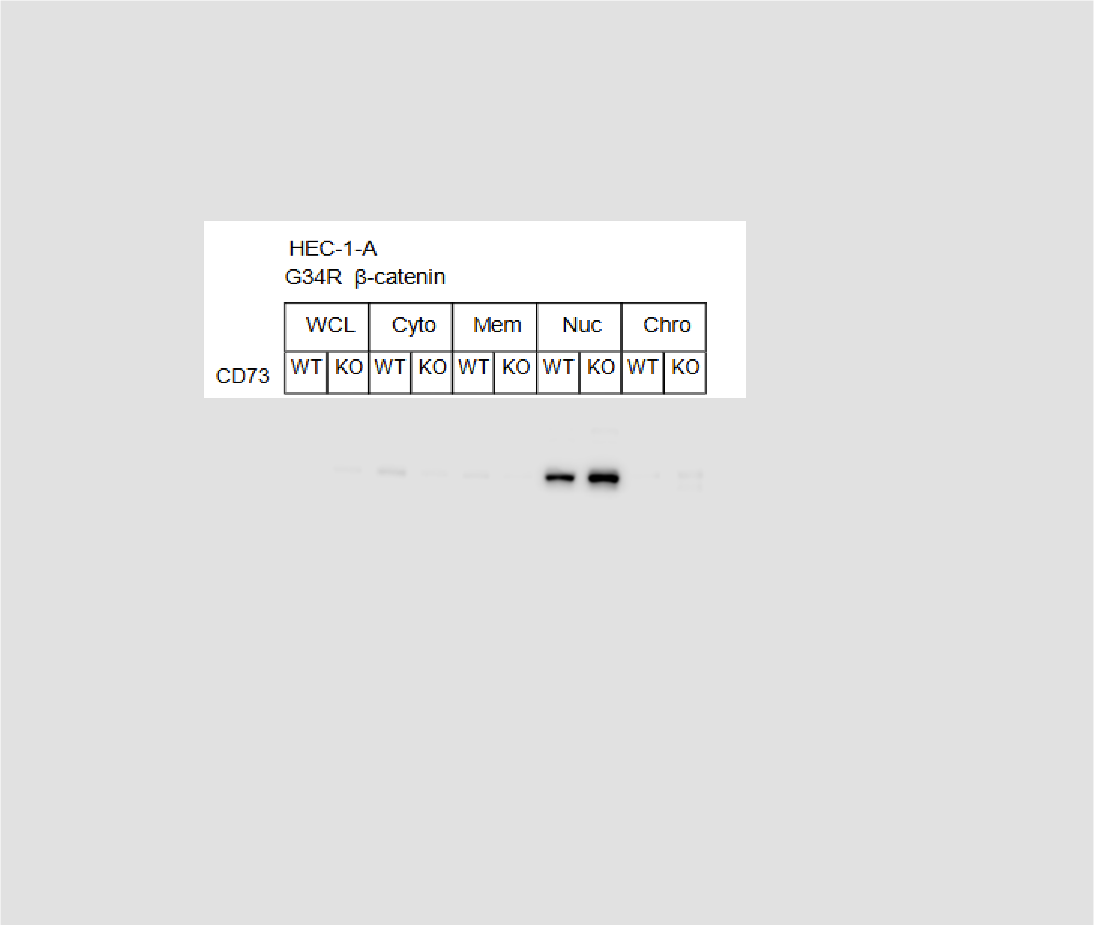

S4 E Images – H2AX

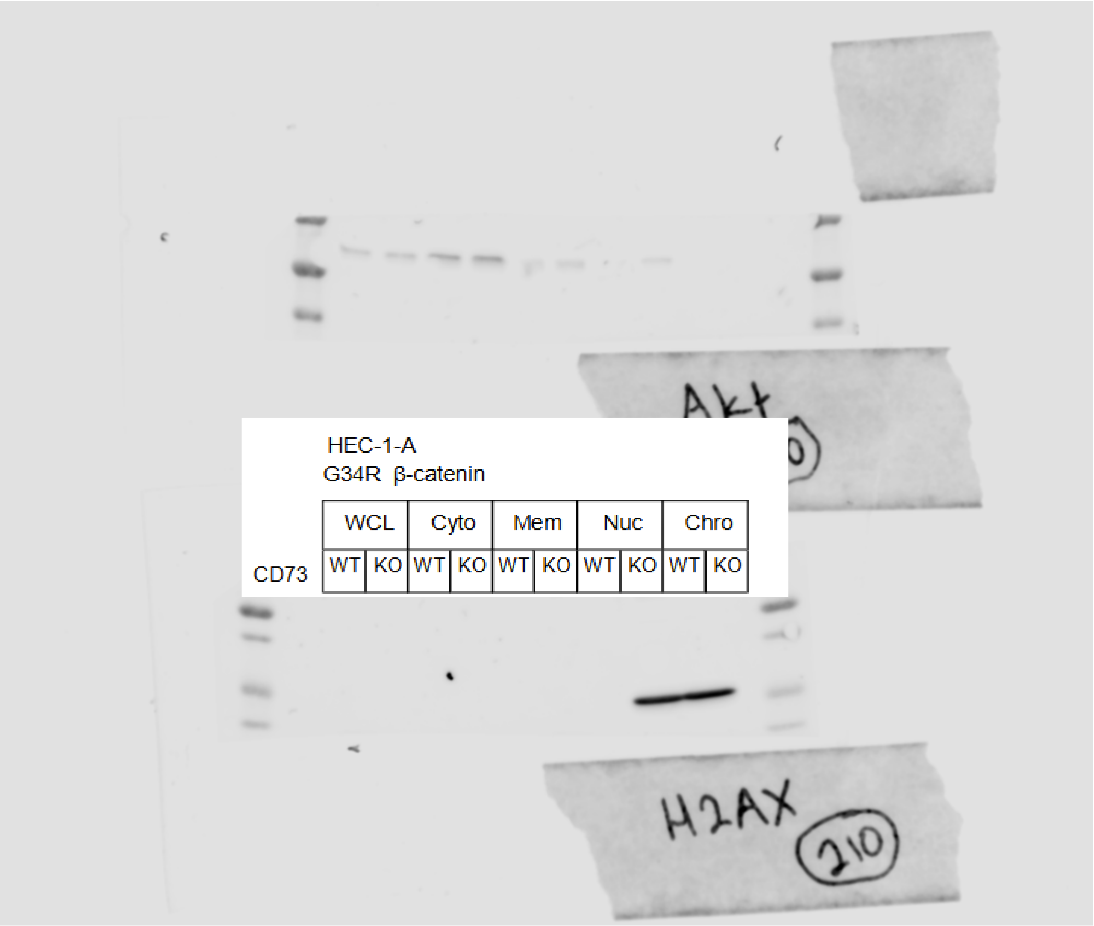

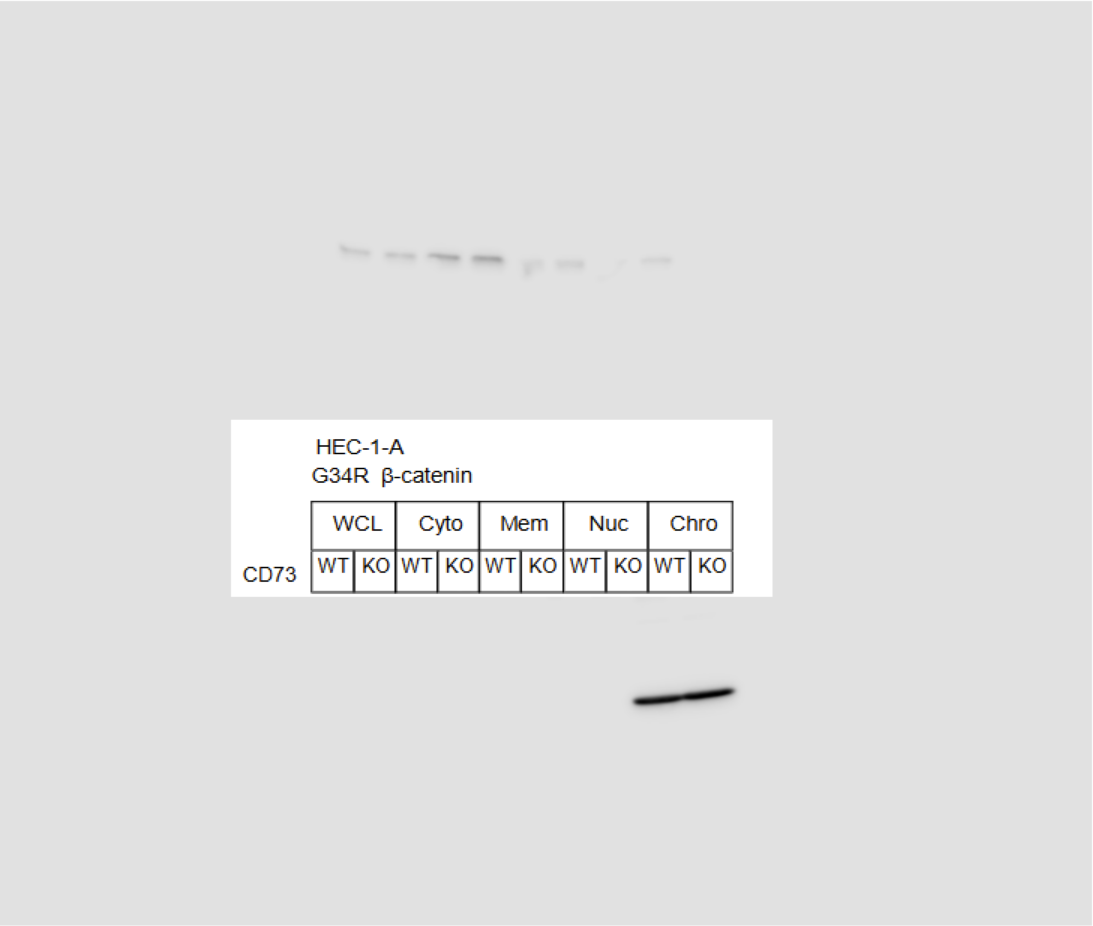

S4 E Images – Total Protein

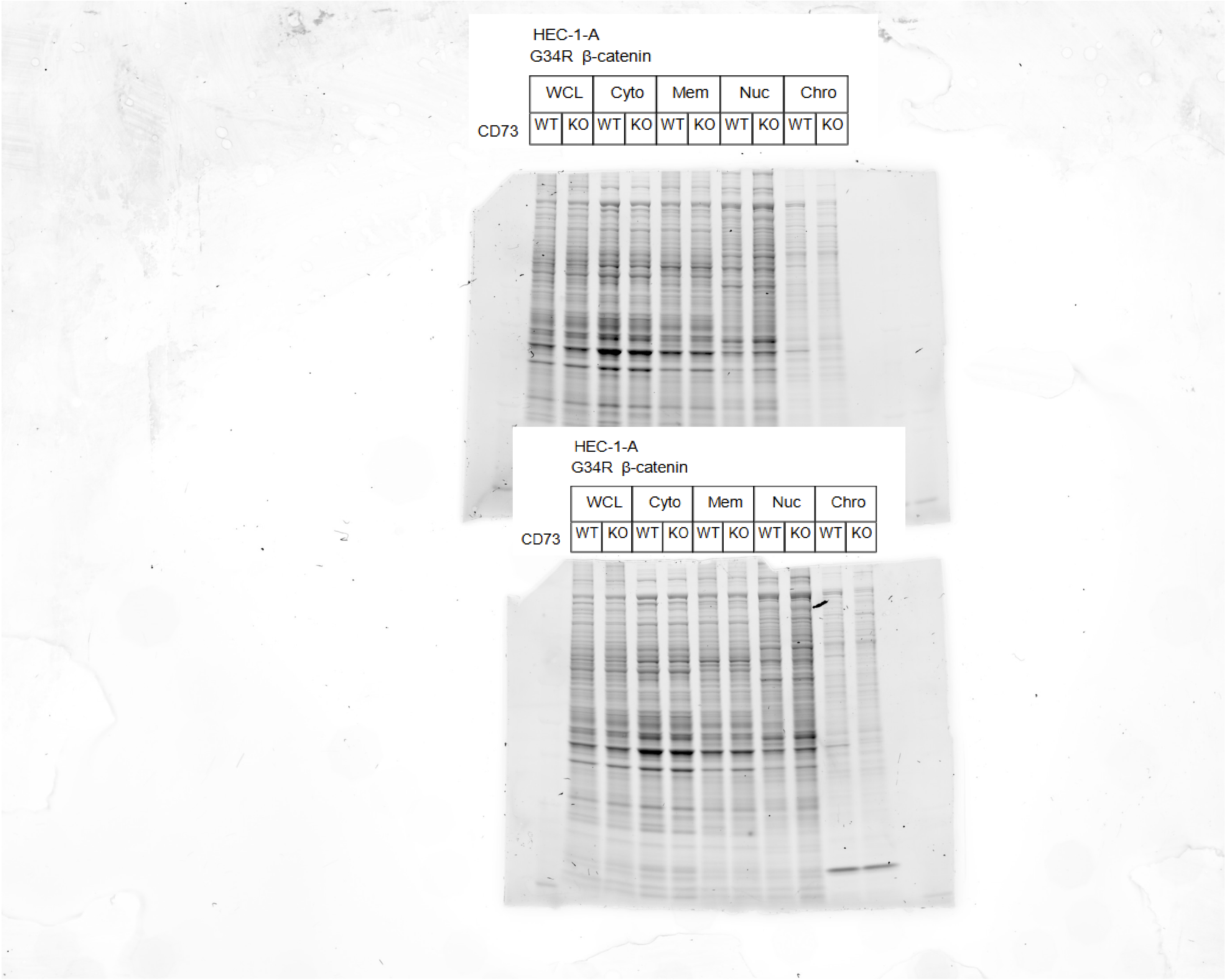

Supplemental Figure 5

S5 A

Total Protein

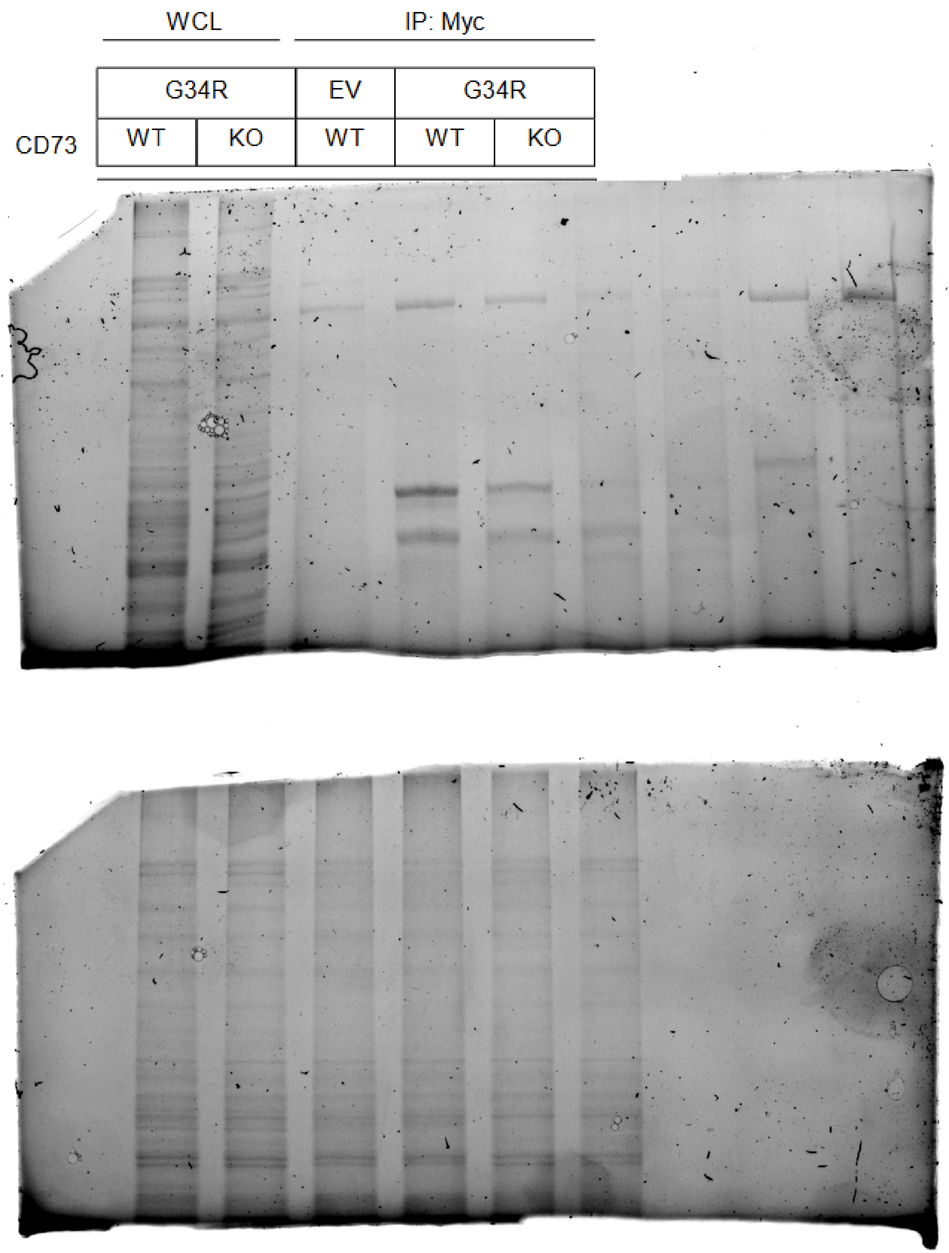

S5 A

E-cadherin

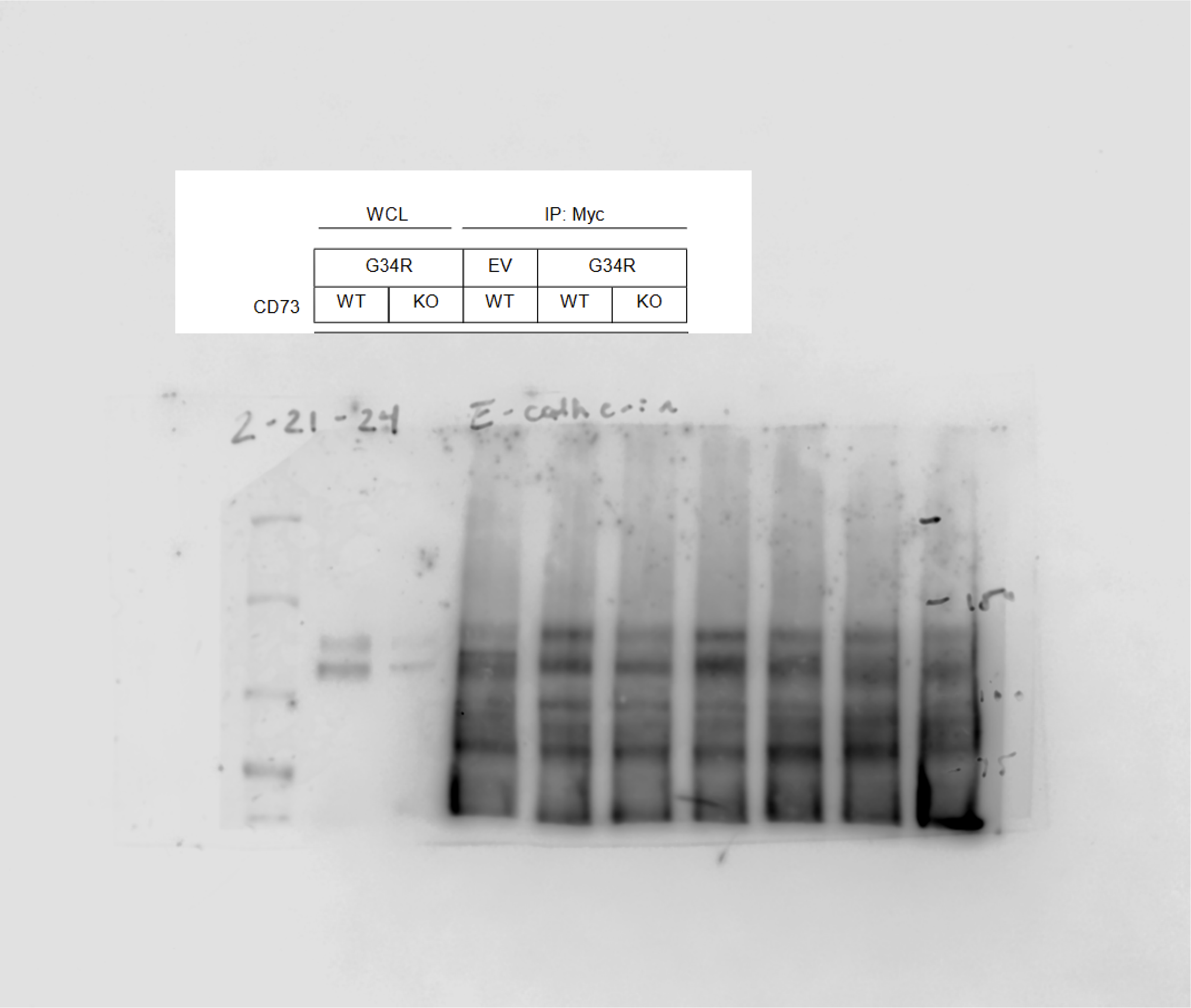

S5 A

E-cadherin

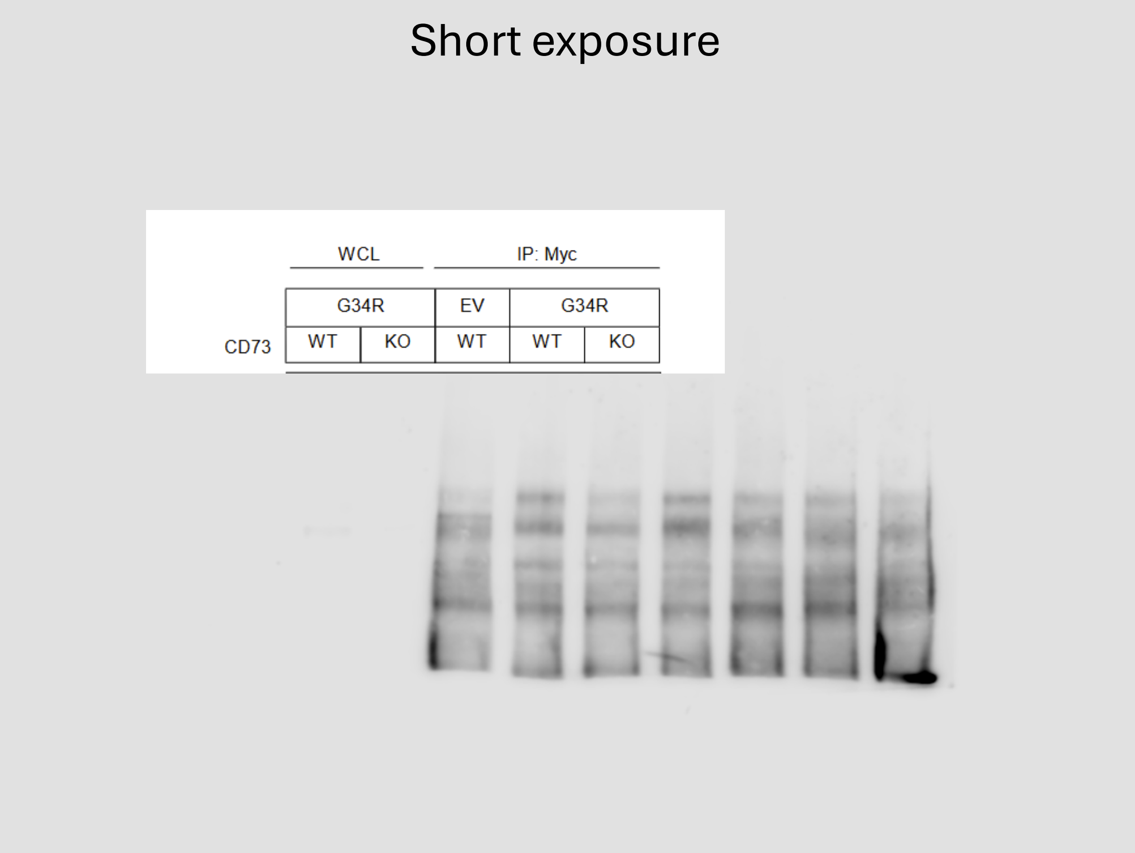

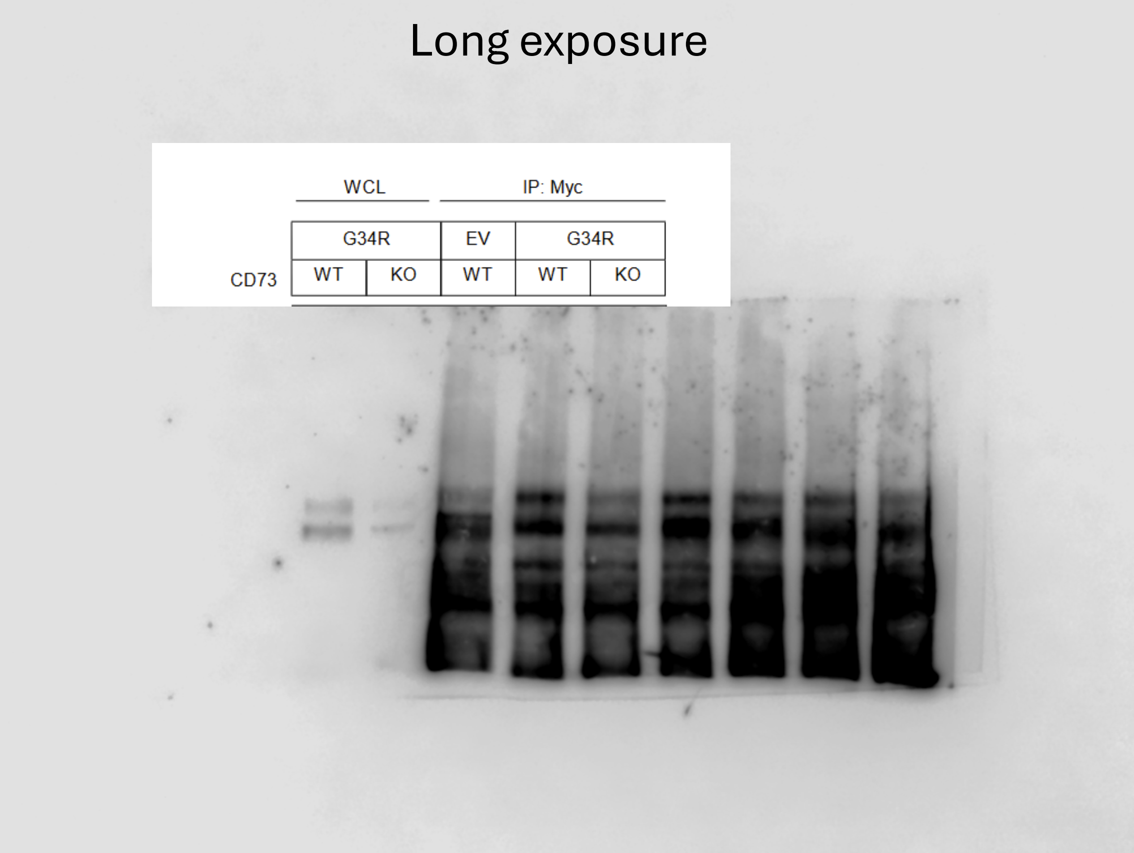

S5 A

Myc

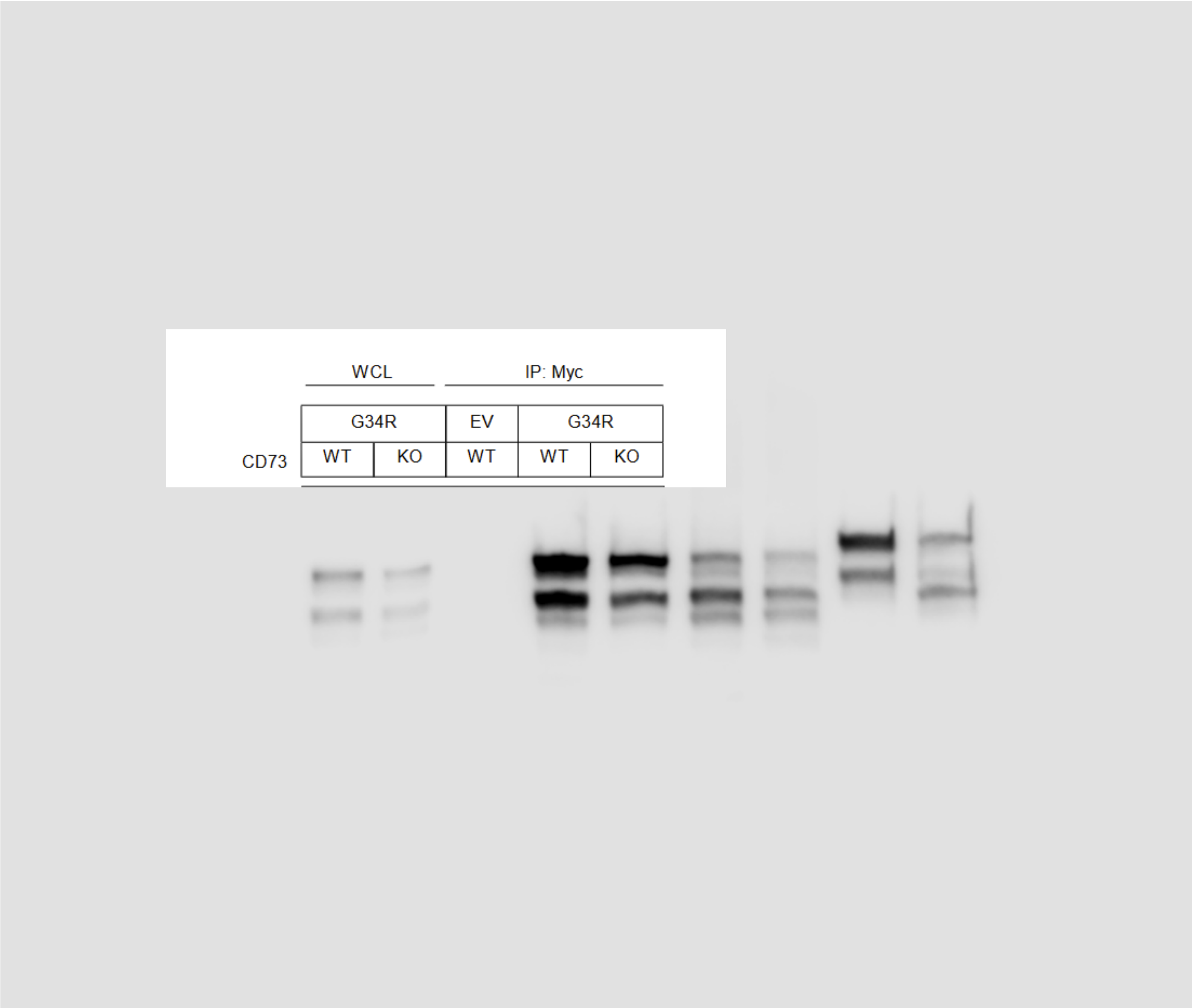

S5 B

Total Protein

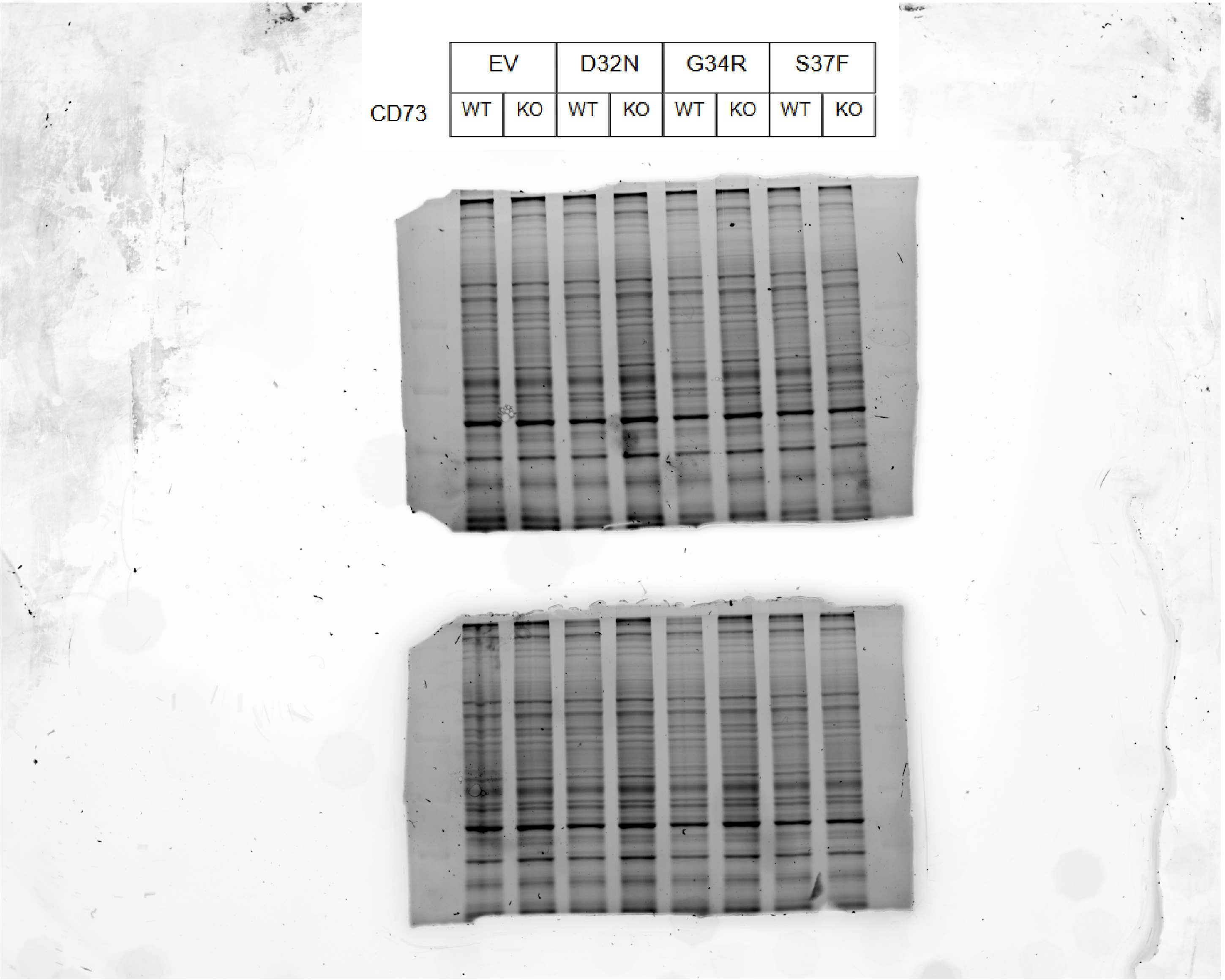

S5 B

a-catenin

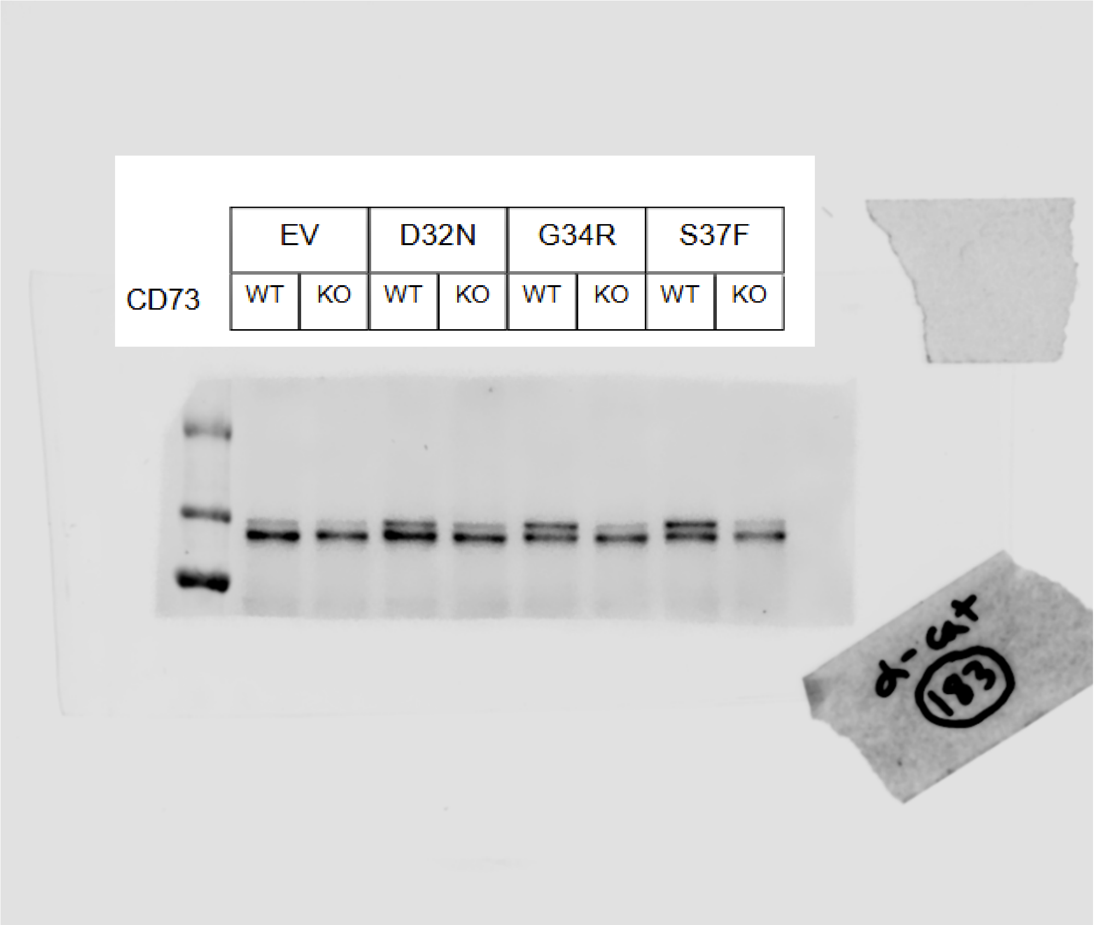

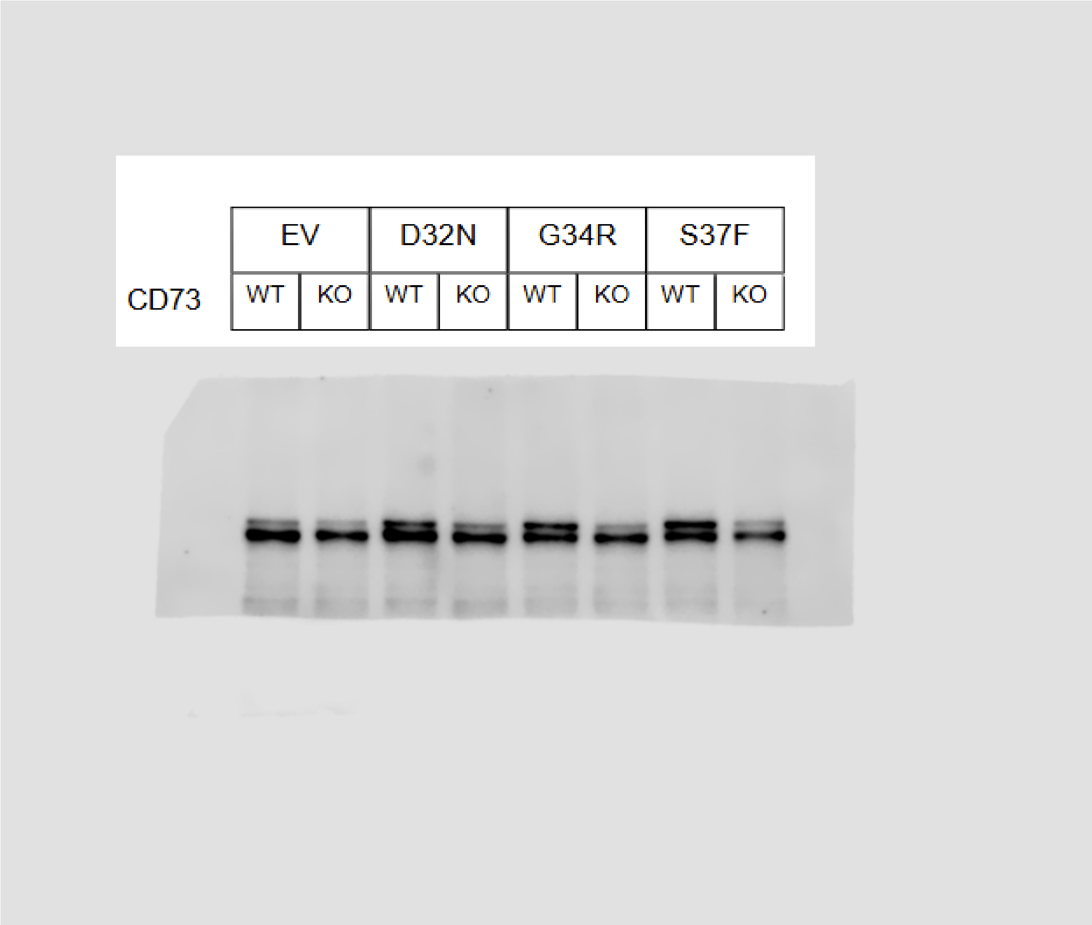

S5 B

CD73

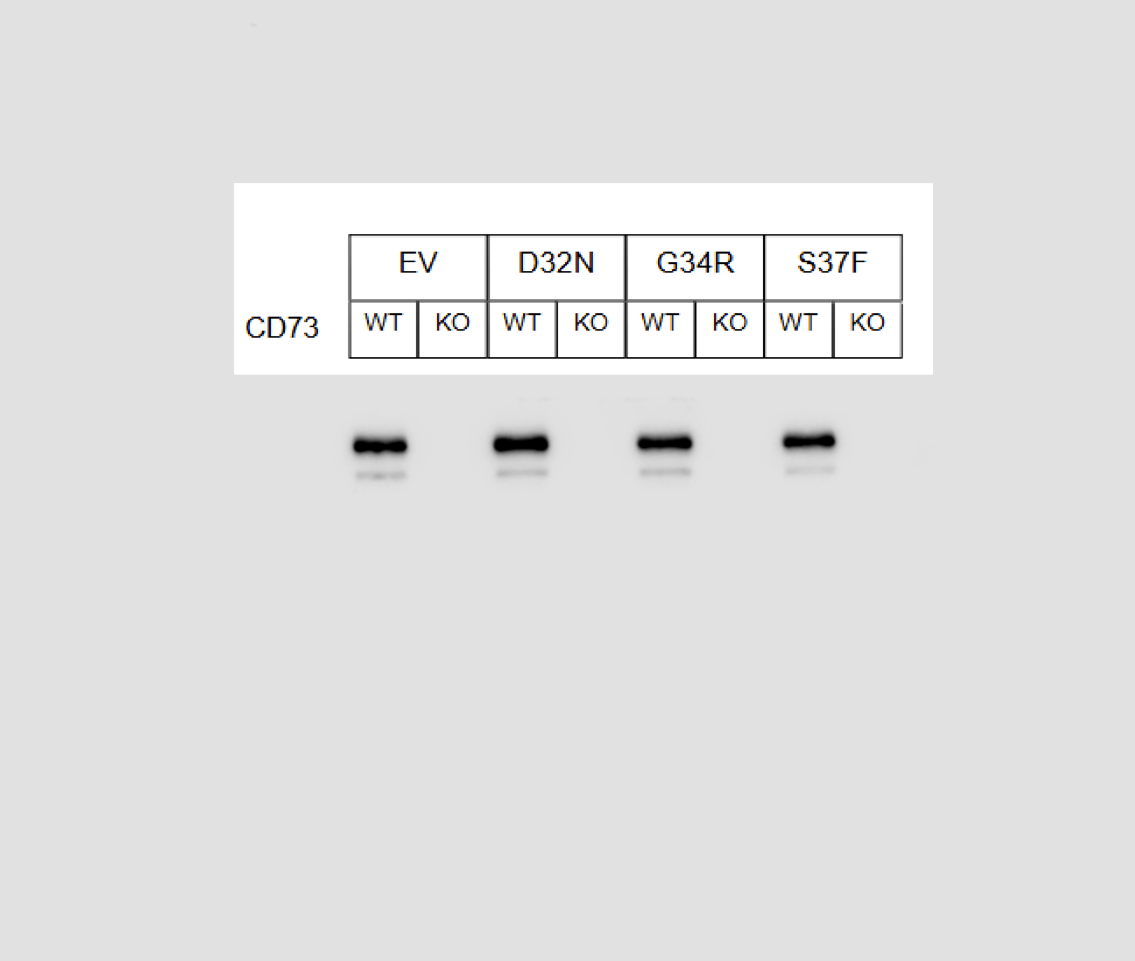

S5 B

E-cadherin

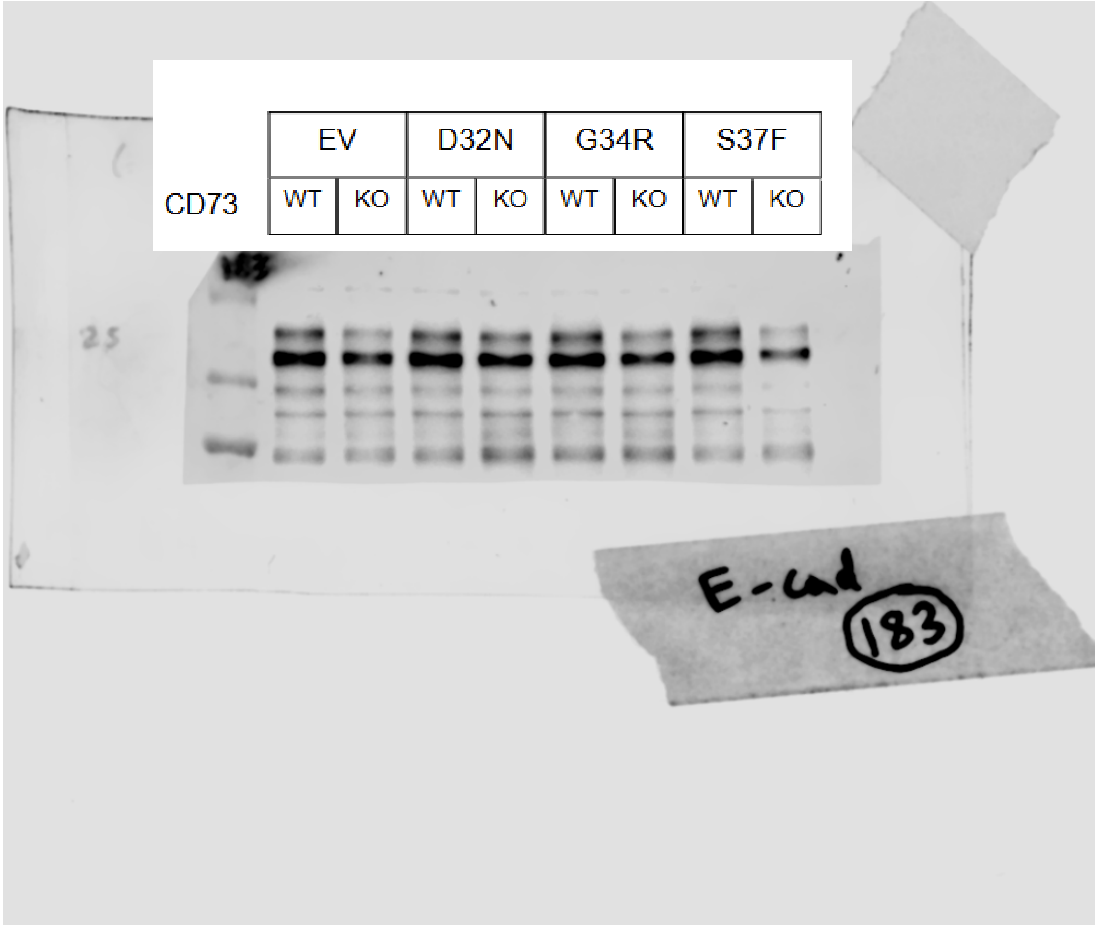

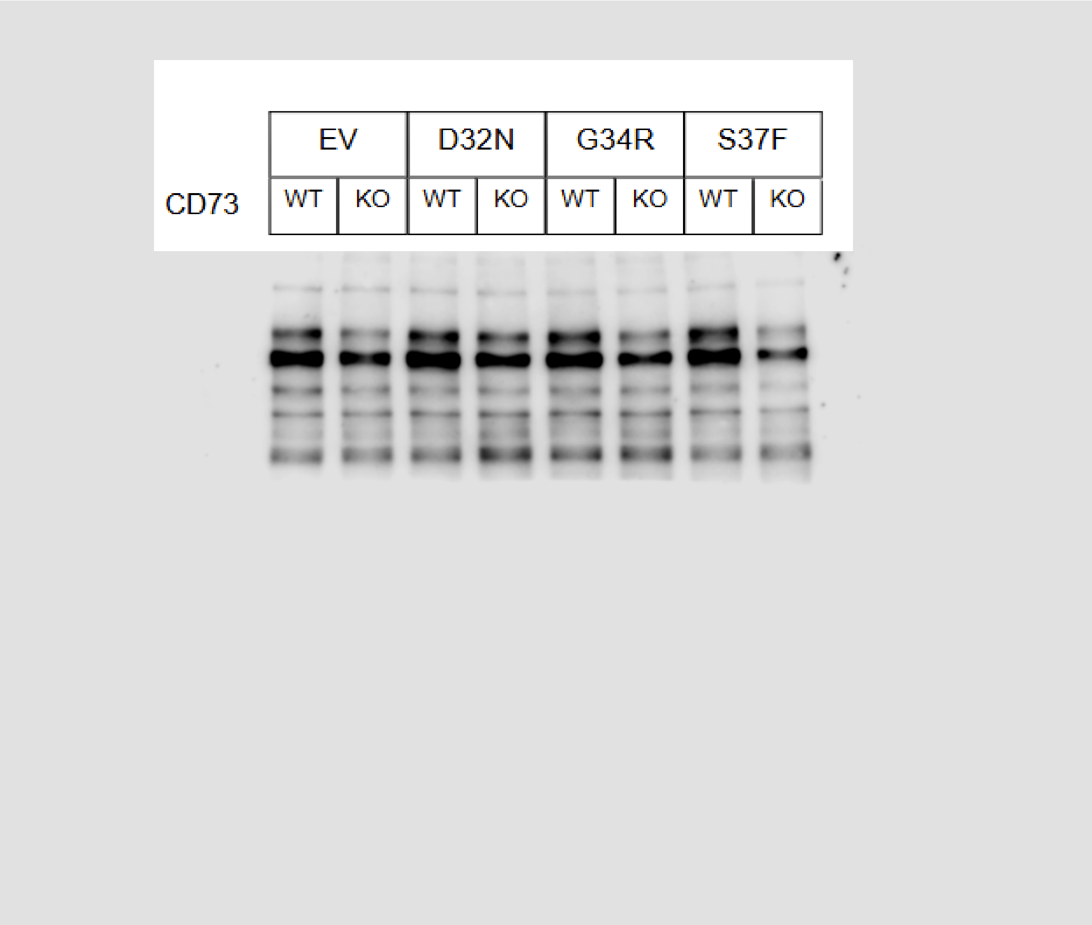

S5 B

Myc

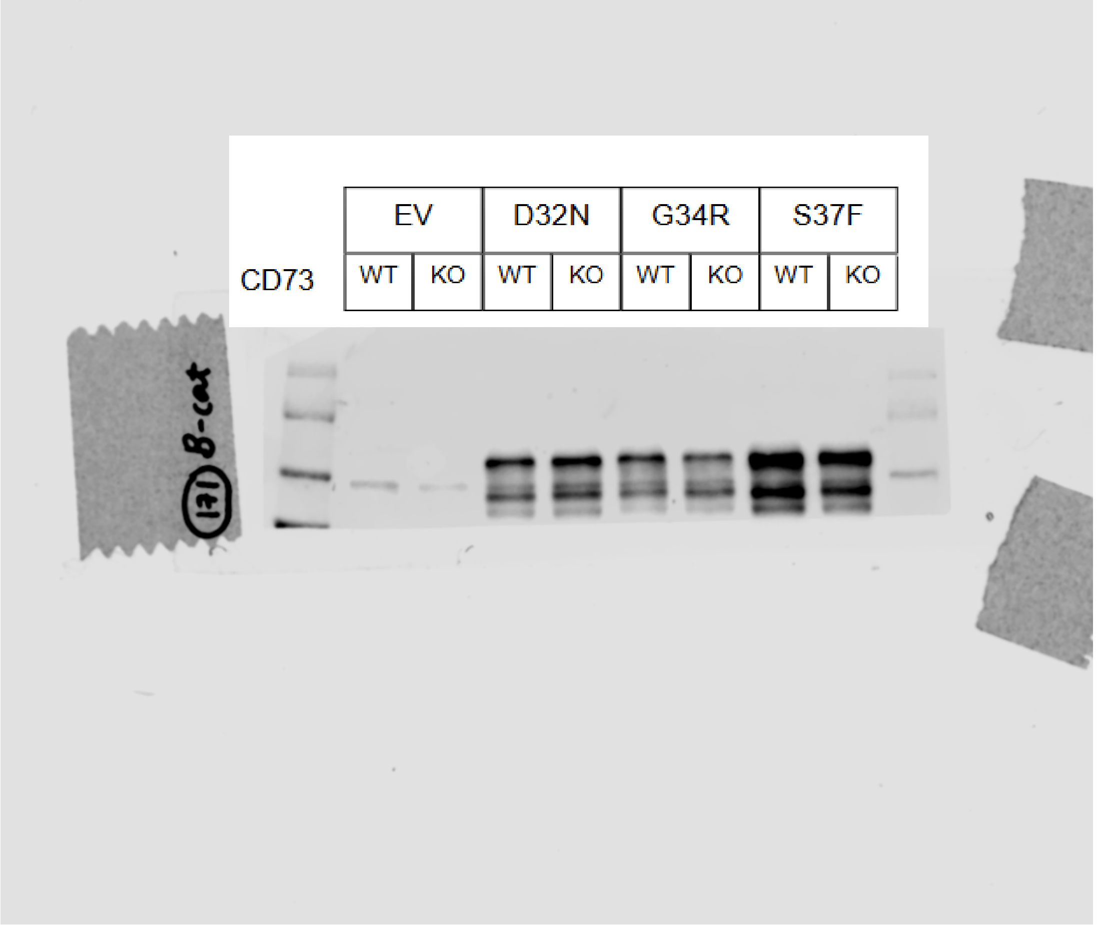
EV
D32N
G34R
S37F
CD73
WT
KO
WT
KO
WT
KO
WT
KO
B-cat
171

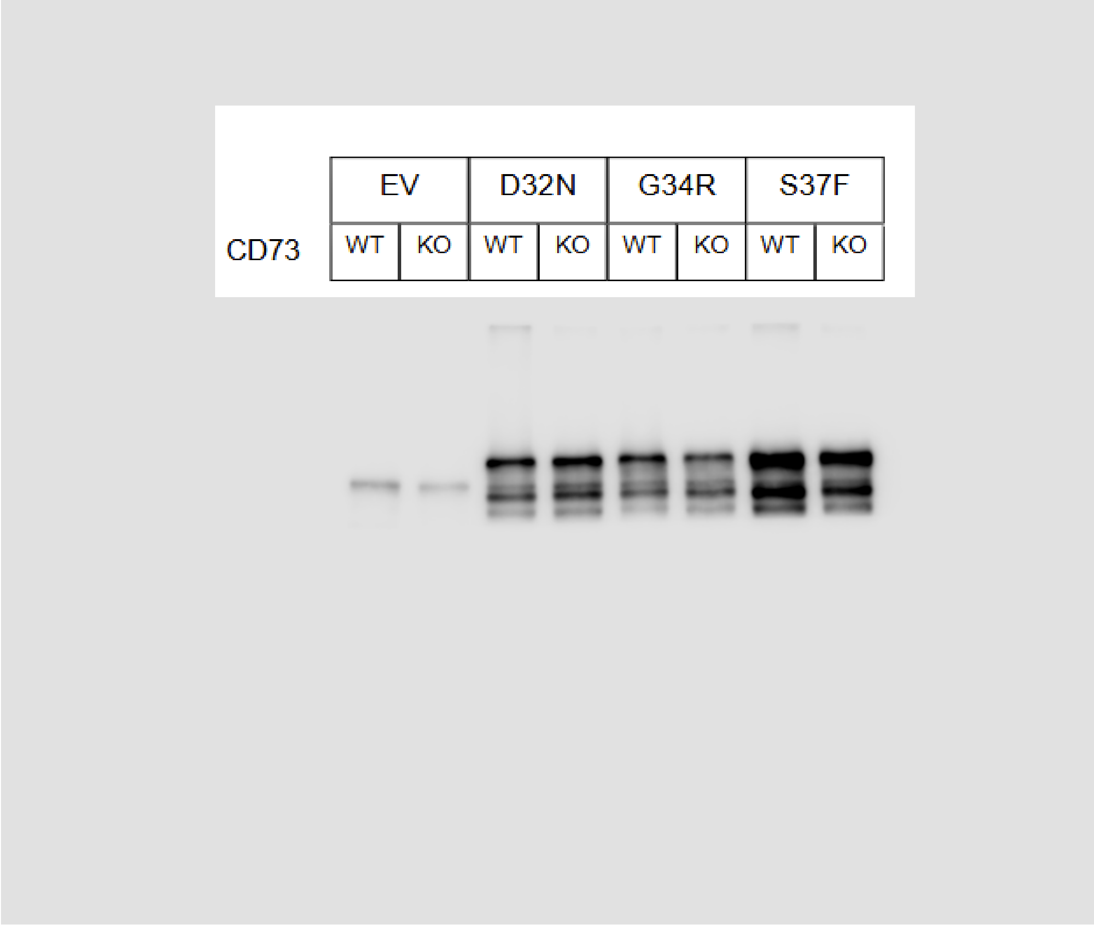
EV
D32N
G34R
S37F
CD73
WT
KO
WT
KO
WT
KO
WT
KO

Figure S6

S6 A

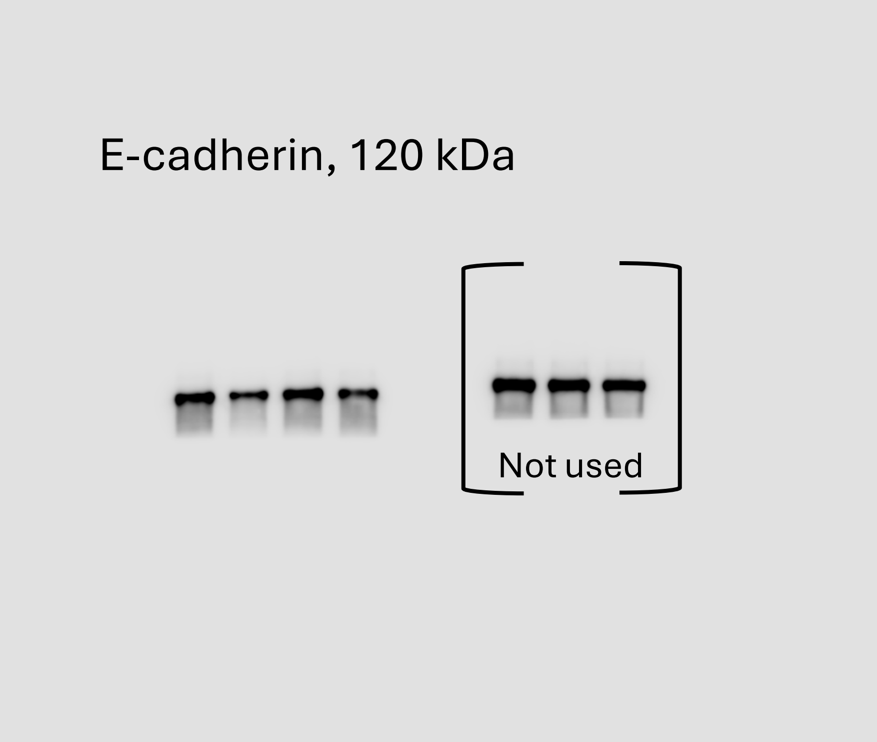

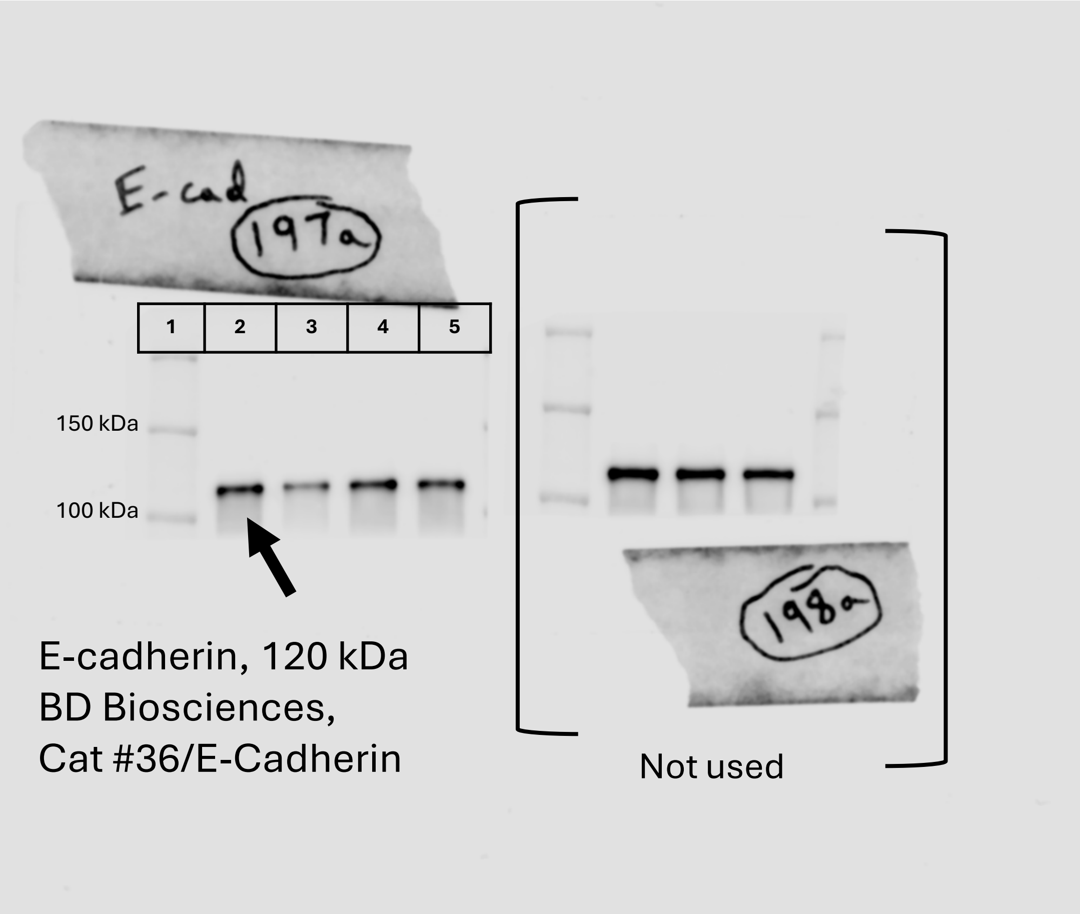

1	2	3	4	5
Ladder	HEC-1-A WT	HEC-1-A NT5E KO	HEC-1-A A1R KO	HEC-1-A A2BR KO

S6 A

1	2	3	4	5
Ladder	HEC-1-A WT	HEC-1-A NT5E KO	HEC-1-A A1R KO	HEC-1-A A2BR KO

Total Protein

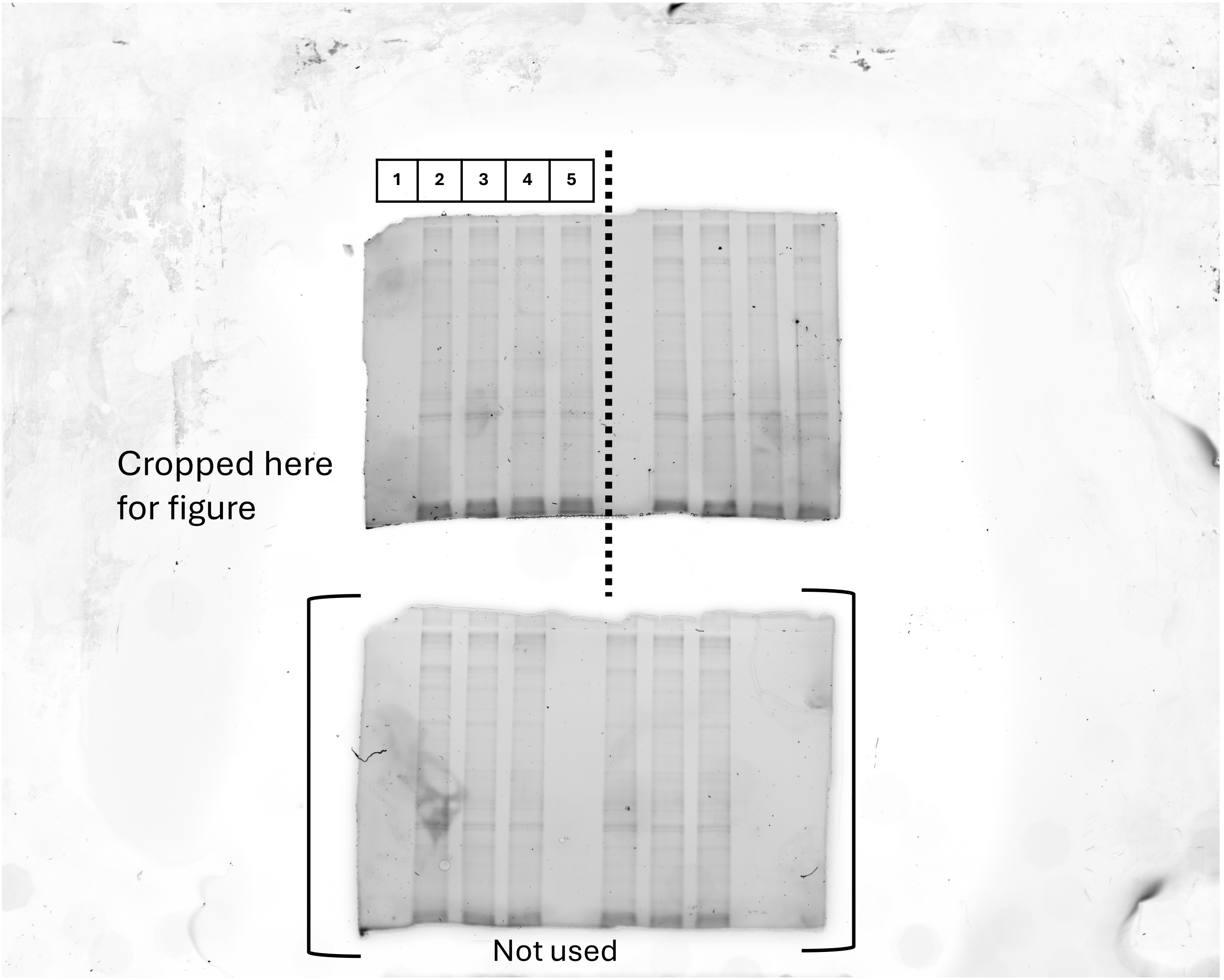

Figure S9

S9 A

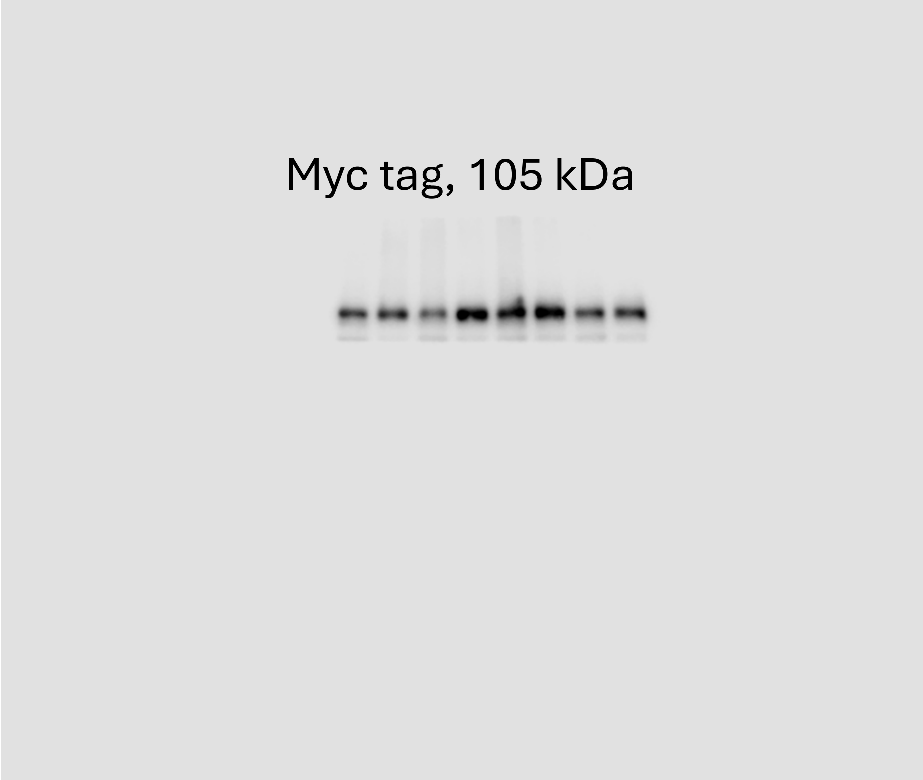

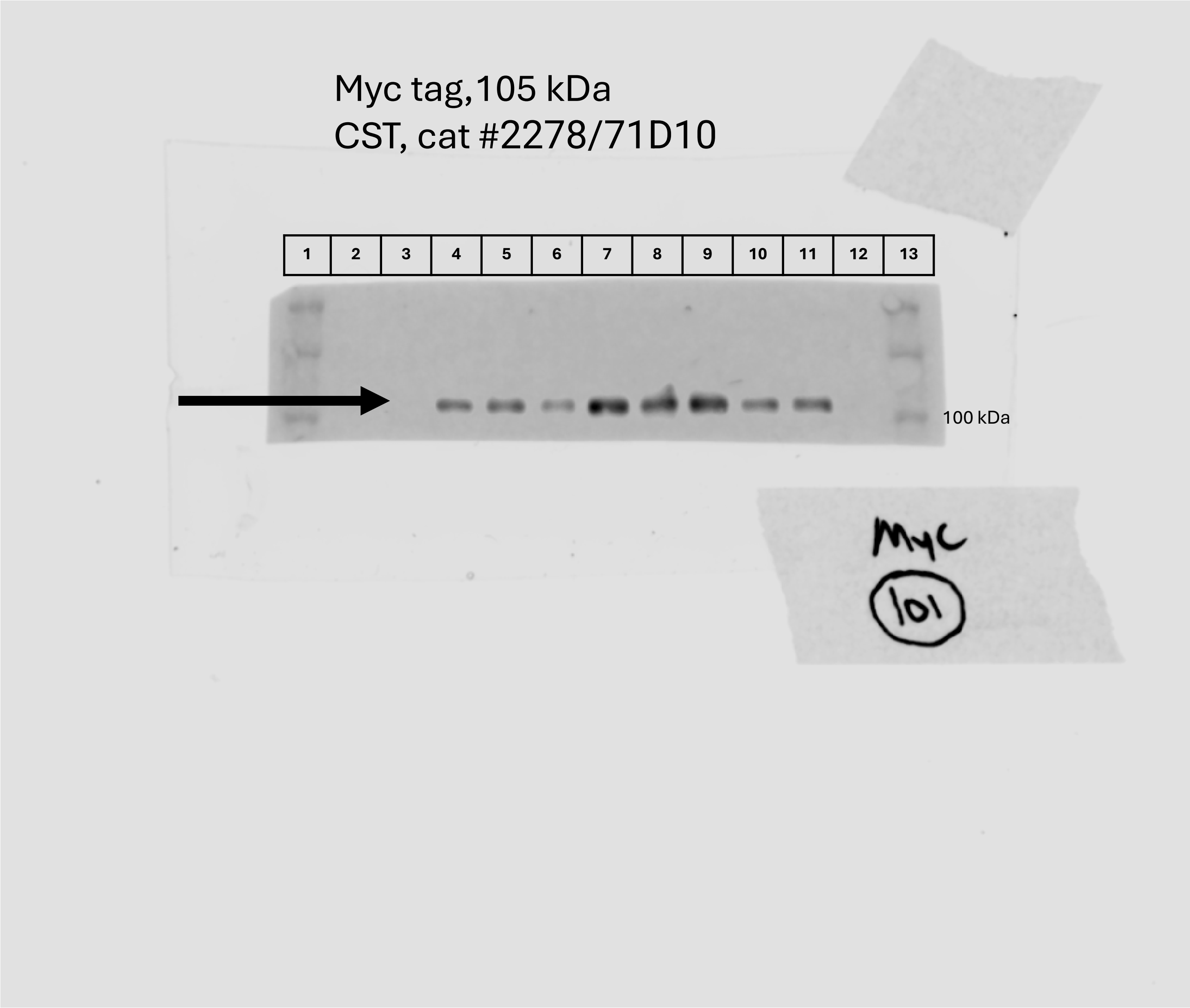

1	2	3	4	5	6	7	8	9	10	11	12	13
Ladder	NT5E WT, EV	NT5E KO, EV	NT5E WT, D32N	NT5E KO, D32N	NT5E WT, S33F	NT5E KO, S33F	NT5E WT, G34R	NT5E KO, G34R	NT5E WT, S37F	NT5E KO, S37F		Ladder

S9 A

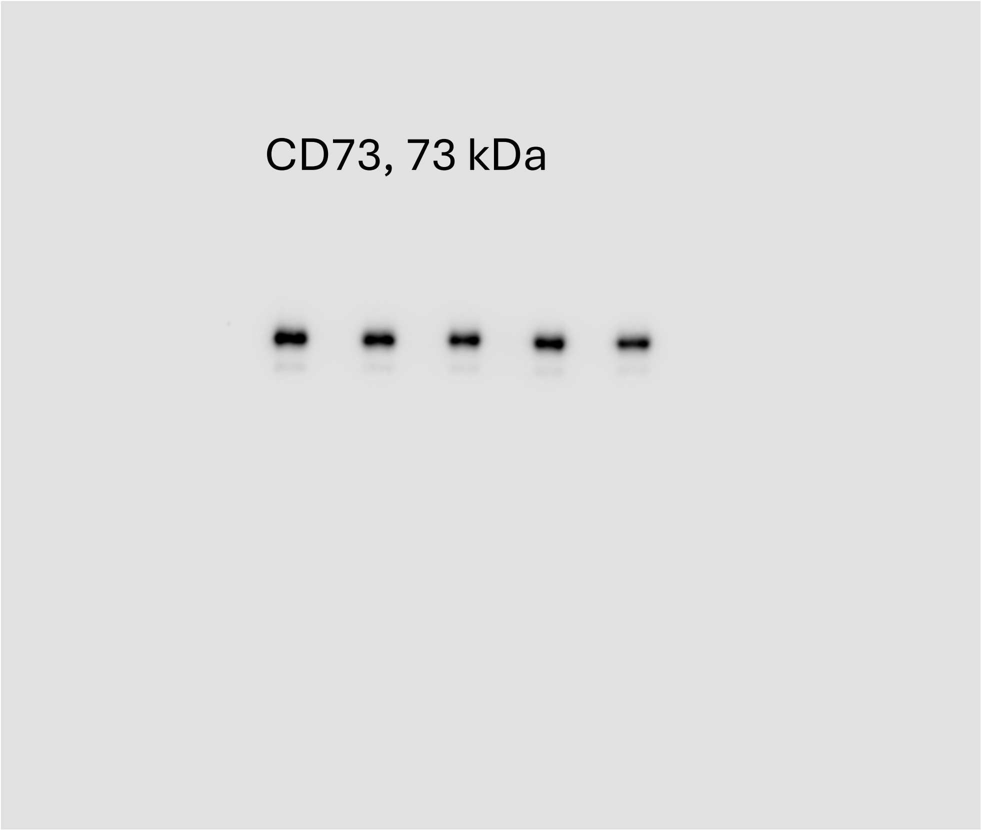

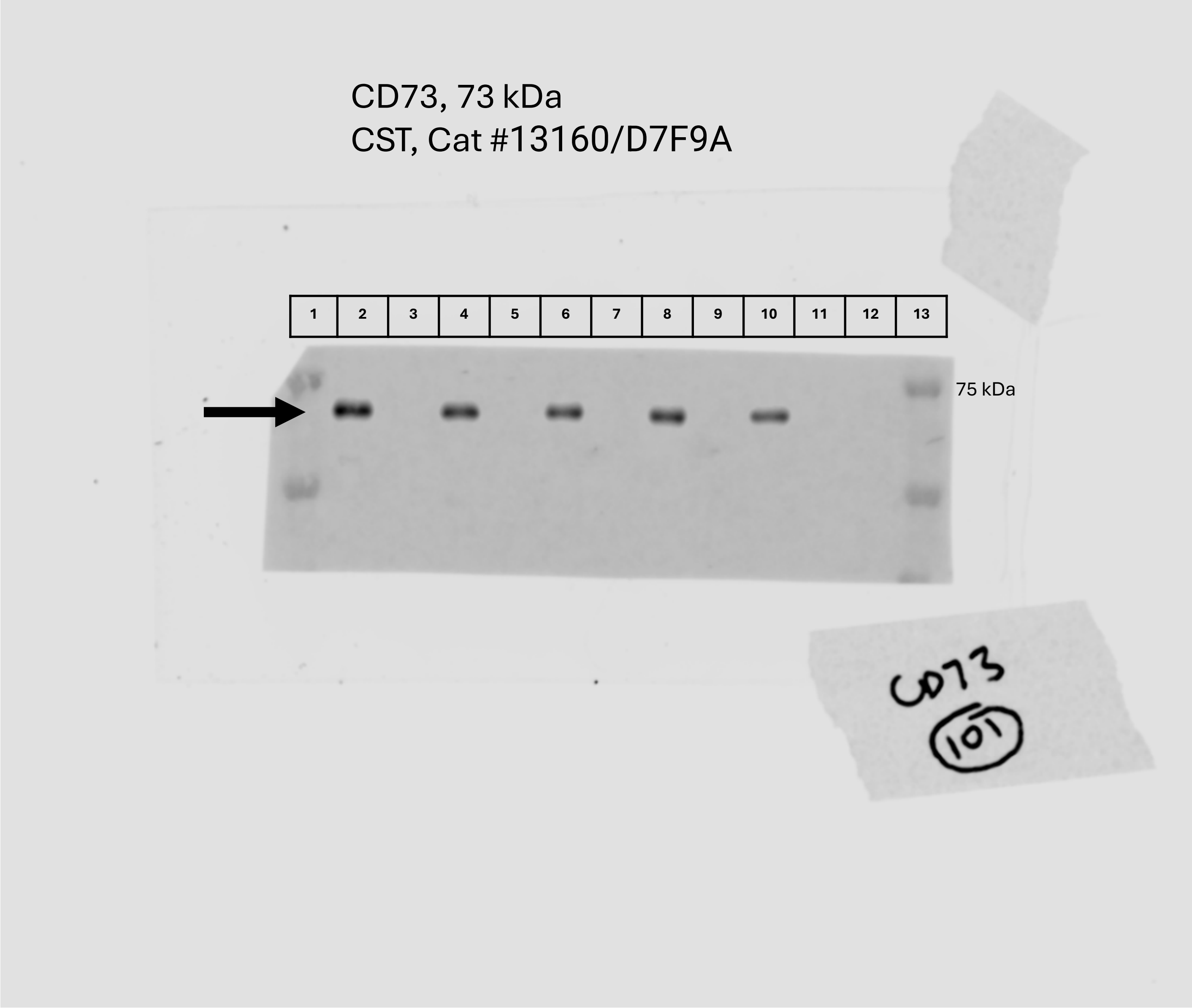

1	2	3	4	5	6	7	8	9	10	11	12	13
Ladder	NT5E WT, EV	NT5E KO, EV	NT5E WT, D32N	NT5E KO, D32N	NT5E WT, S33F	NT5E KO, S33F	NT5E WT, G34R	NT5E KO, G34R	NT5E WT, S37F	NT5E KO, S37F		Ladder

S9 A

Total Protein

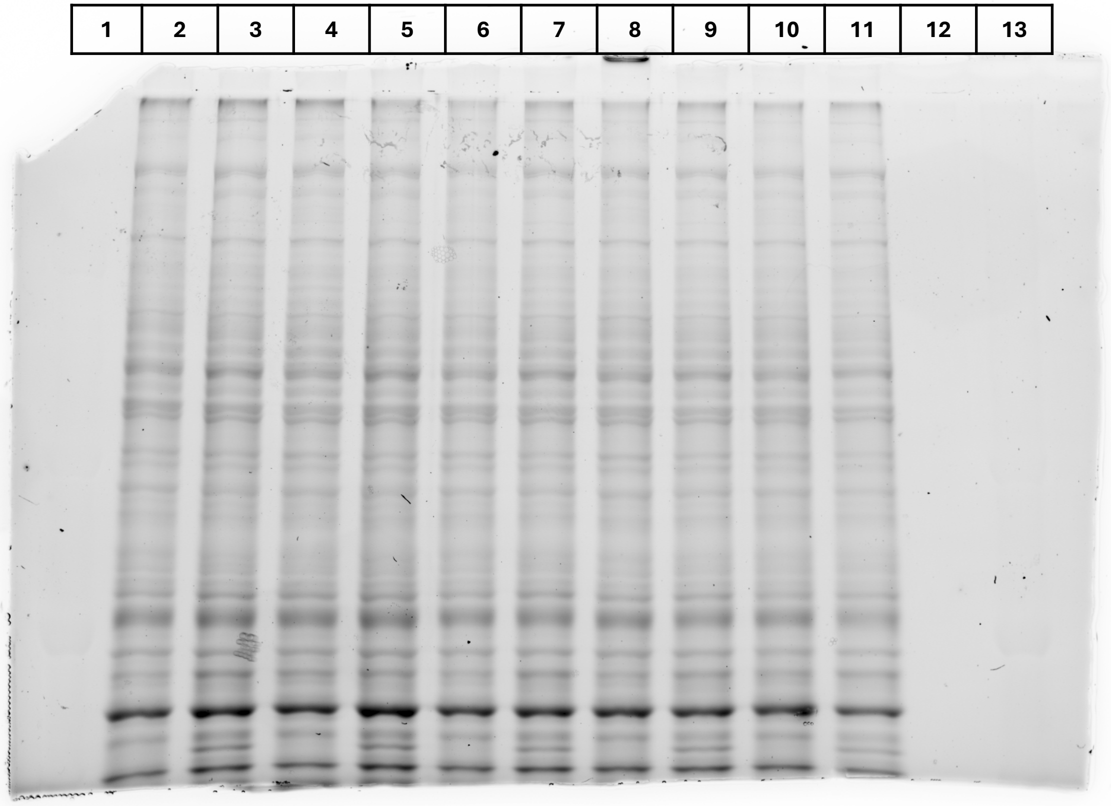

1	2	3	4	5	6	7	8	9	10	11	12	13
Ladder	NT5E WT, EV	NT5E KO, EV	NT5E WT, D32N	NT5E KO, D32N	NT5E WT, S33F	NT5E KO, S33F	NT5E WT, G34R	NT5E KO, G34R	NT5E WT, S37F	NT5E KO, S37F		Ladder
